# Supplementary figures and images for: Fibroblasts as an in vitro model of circadian genetic and genomic studies
Source: Mamm Genome. 2024 Jul 3;35(3):432–44. doi: 10.1007/s00335-024-10050-7 (PMC11329553; doi:10.1007/s00335-024-10050-7)

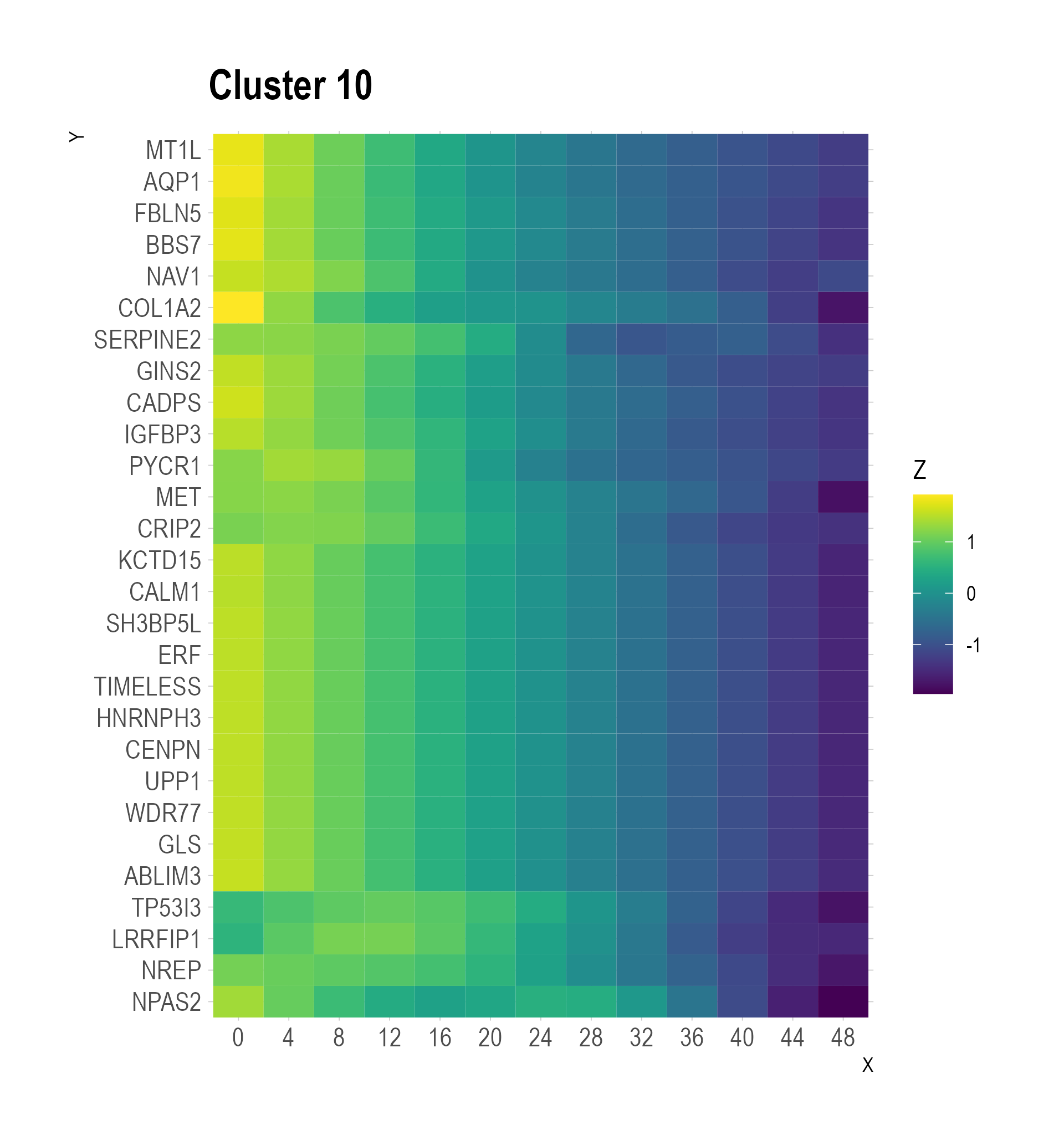

Supplement: Supplementary file 1 — Supplementary file1 (ZIP 1292 kb) [file 335_2024_10050_MOESM1_ESM.zip › All_Circadian_Genes_Heatmap_Clusters/heatmap_cluster 10 .png]

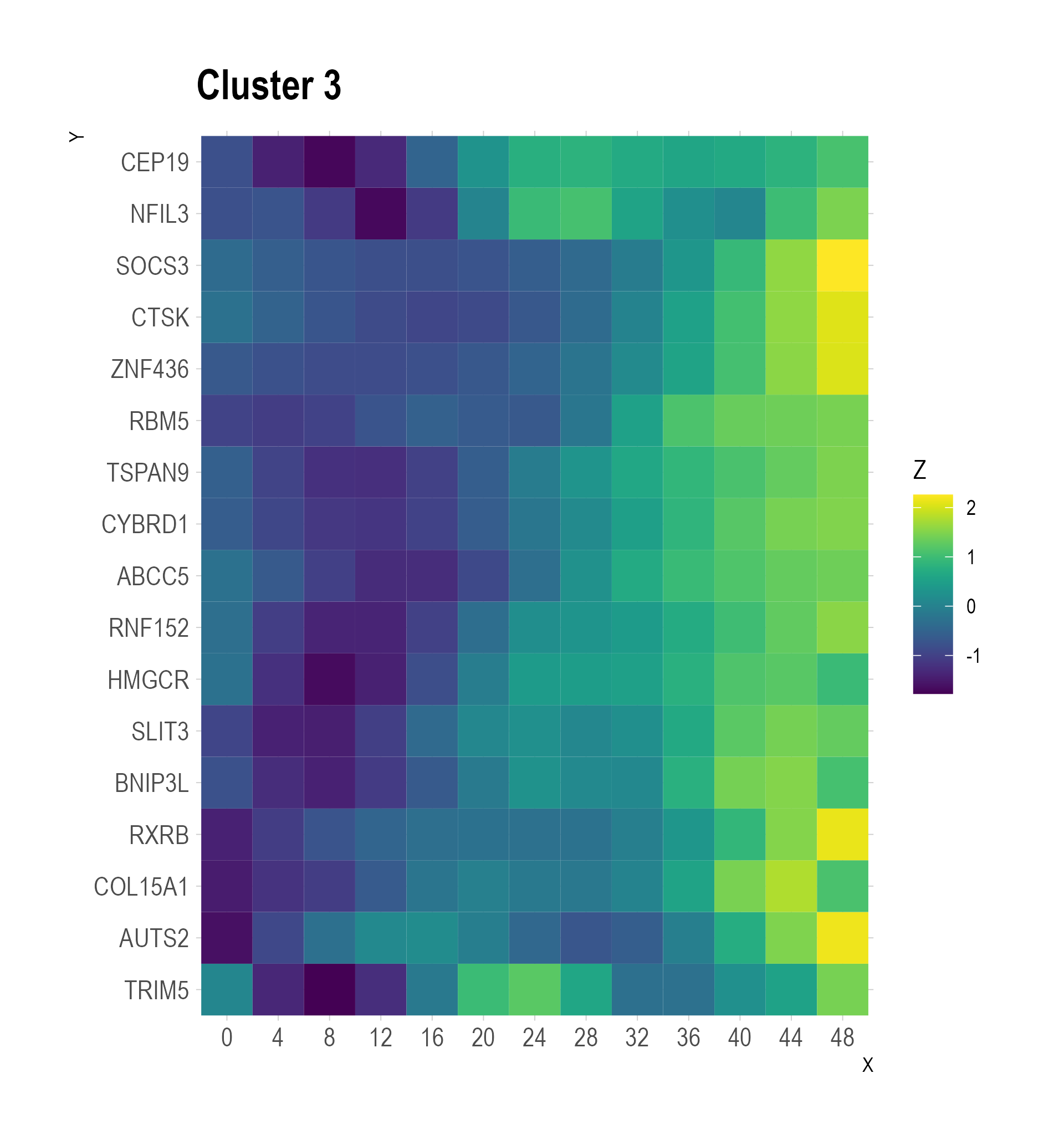

Supplement: Supplementary file 1 — Supplementary file1 (ZIP 1292 kb) [file 335_2024_10050_MOESM1_ESM.zip › All_Circadian_Genes_Heatmap_Clusters/heatmap_cluster 3 .png]

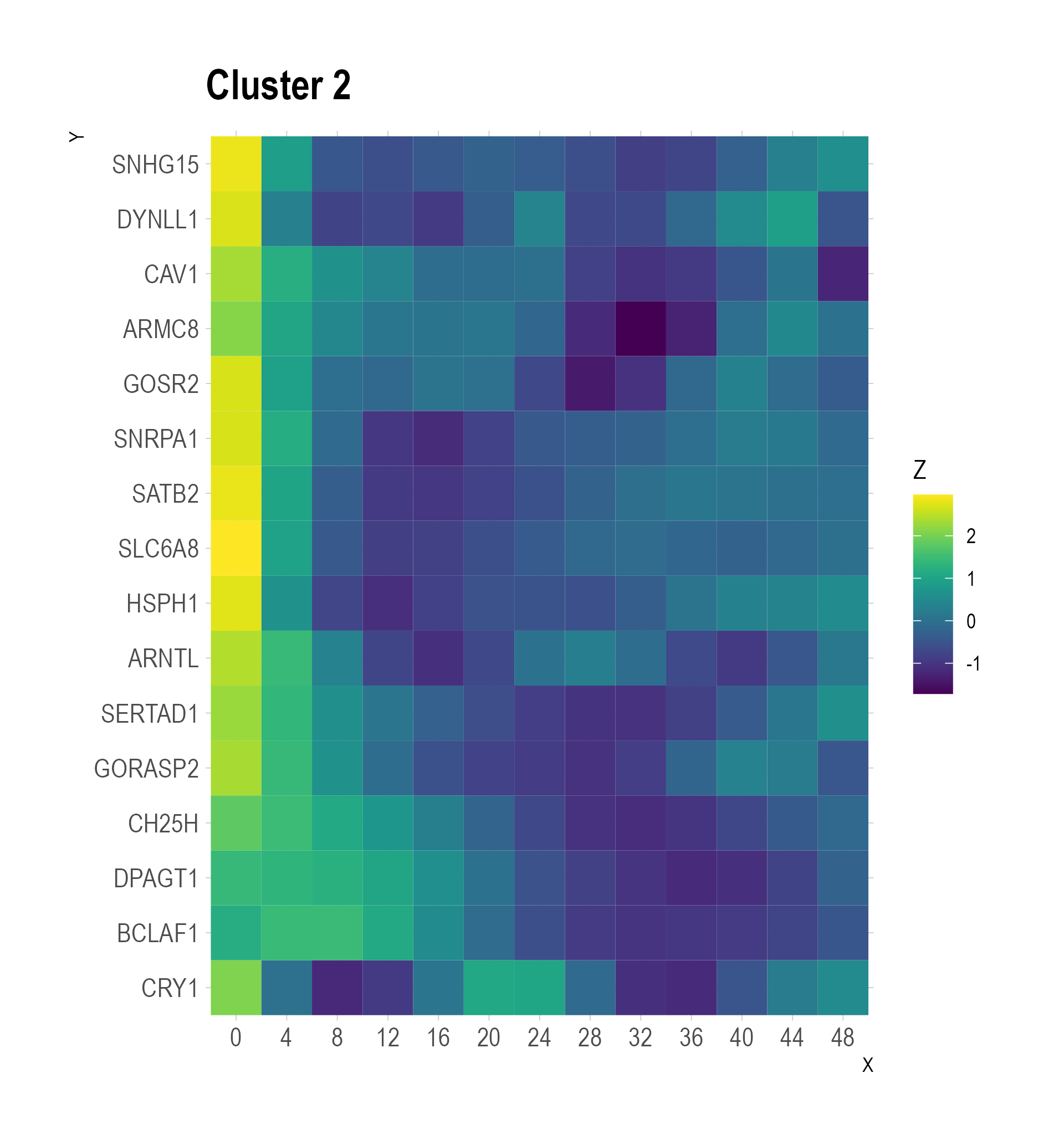

Supplement: Supplementary file 1 — Supplementary file1 (ZIP 1292 kb) [file 335_2024_10050_MOESM1_ESM.zip › All_Circadian_Genes_Heatmap_Clusters/heatmap_cluster 2 .png]

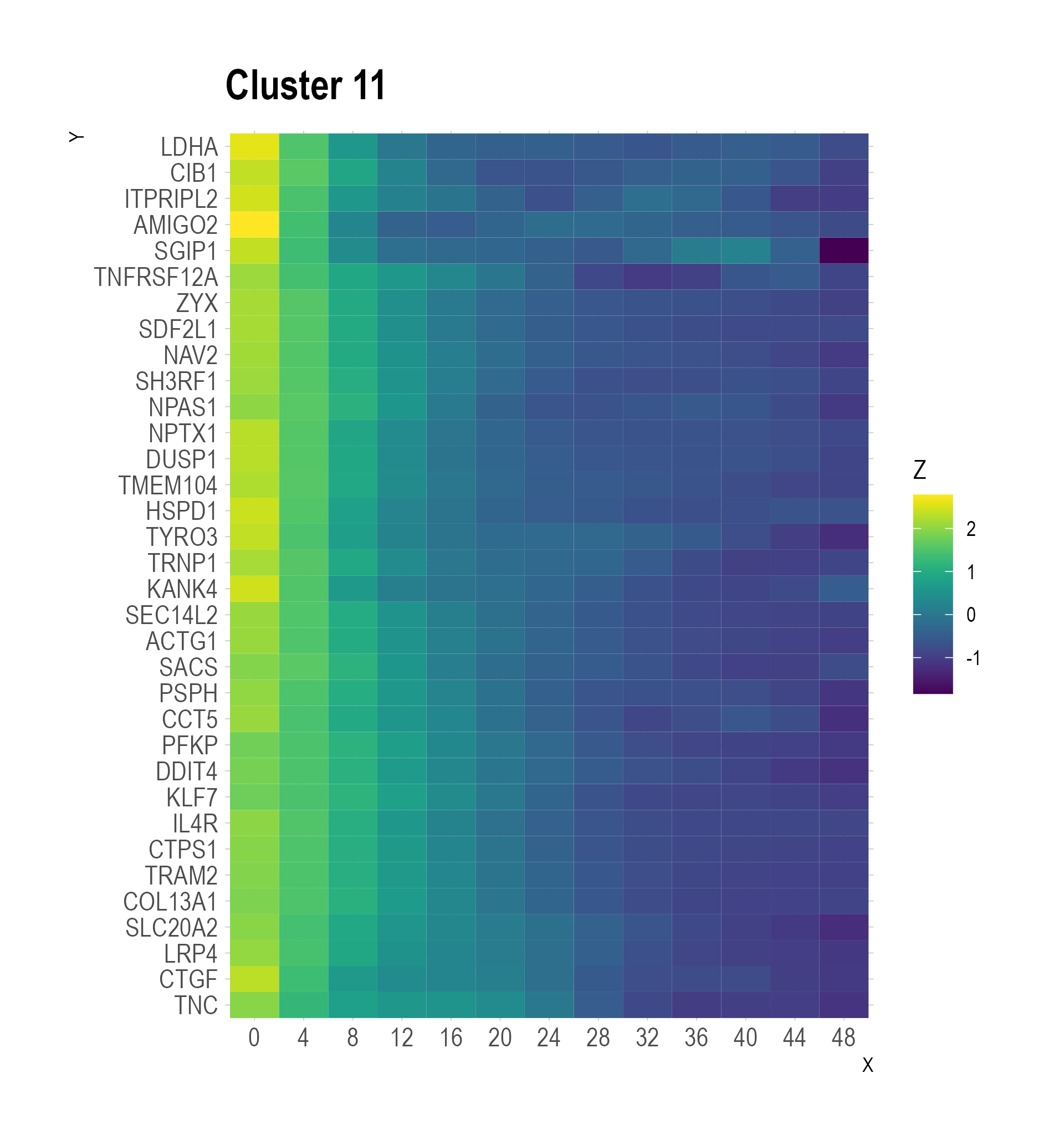

Supplement: Supplementary file 1 — Supplementary file1 (ZIP 1292 kb) [file 335_2024_10050_MOESM1_ESM.zip › All_Circadian_Genes_Heatmap_Clusters/heatmap_cluster 11 .png]

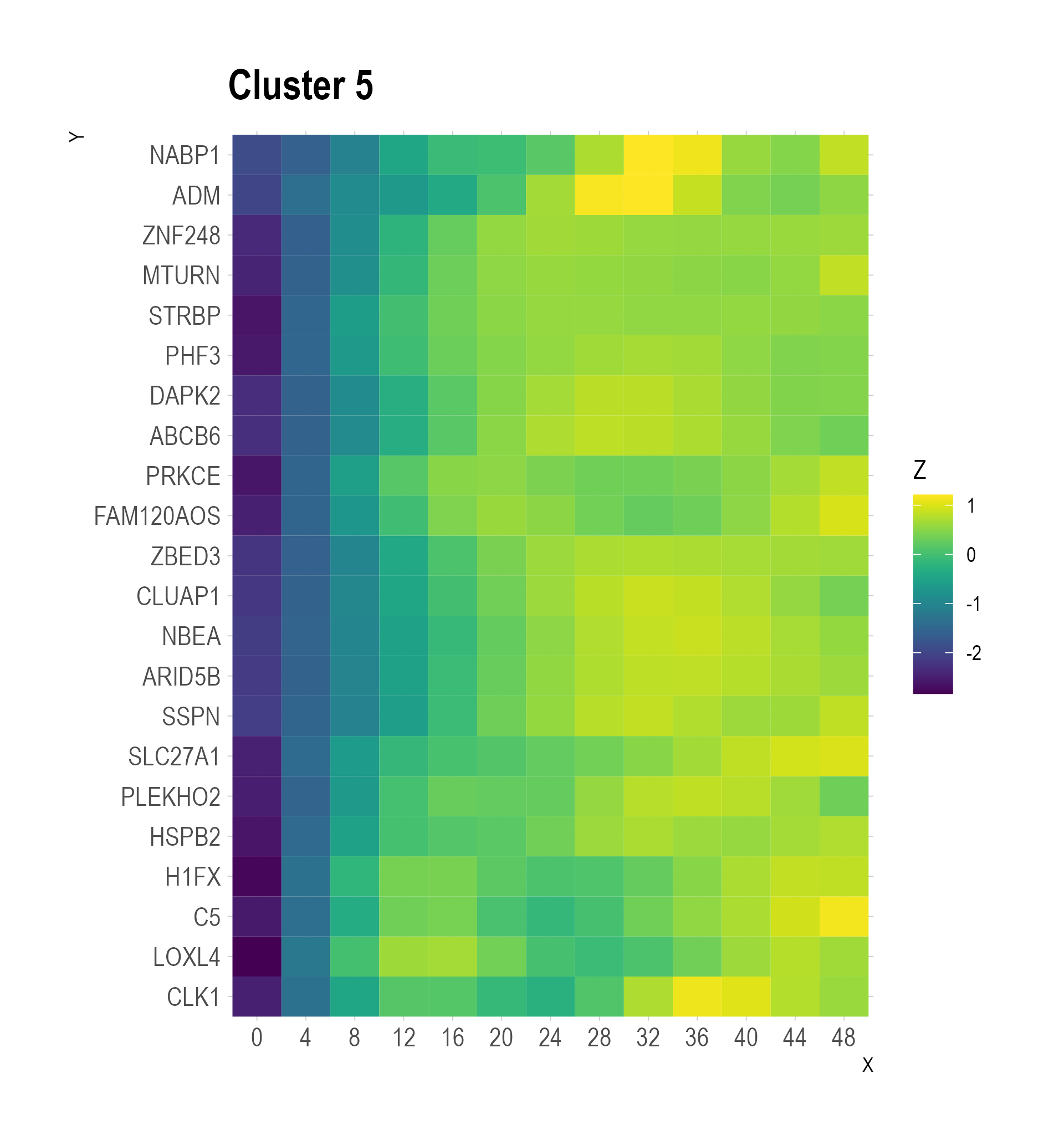

Supplement: Supplementary file 1 — Supplementary file1 (ZIP 1292 kb) [file 335_2024_10050_MOESM1_ESM.zip › All_Circadian_Genes_Heatmap_Clusters/heatmap_cluster 5 .png]

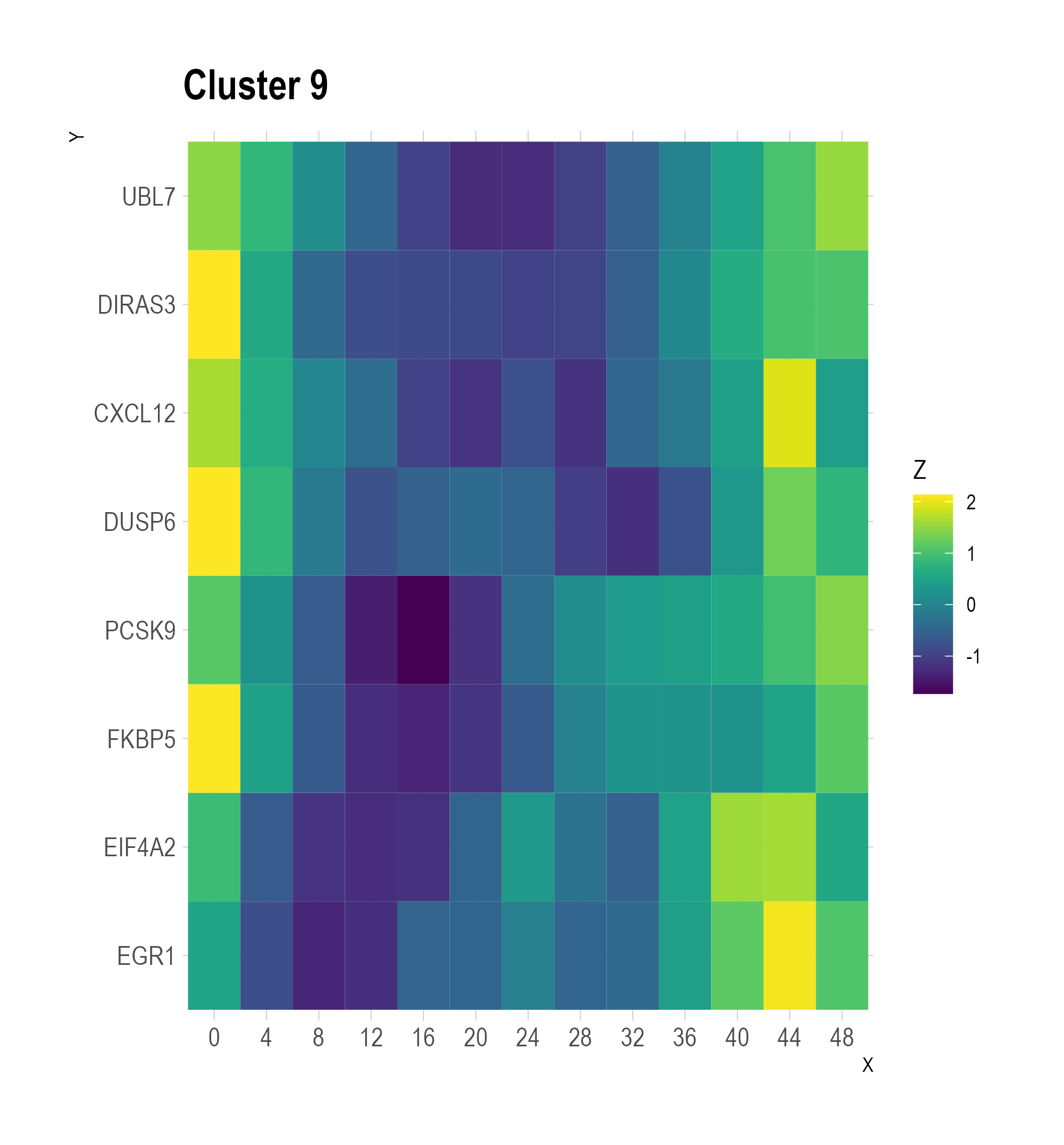

Supplement: Supplementary file 1 — Supplementary file1 (ZIP 1292 kb) [file 335_2024_10050_MOESM1_ESM.zip › All_Circadian_Genes_Heatmap_Clusters/heatmap_cluster 9 .png]

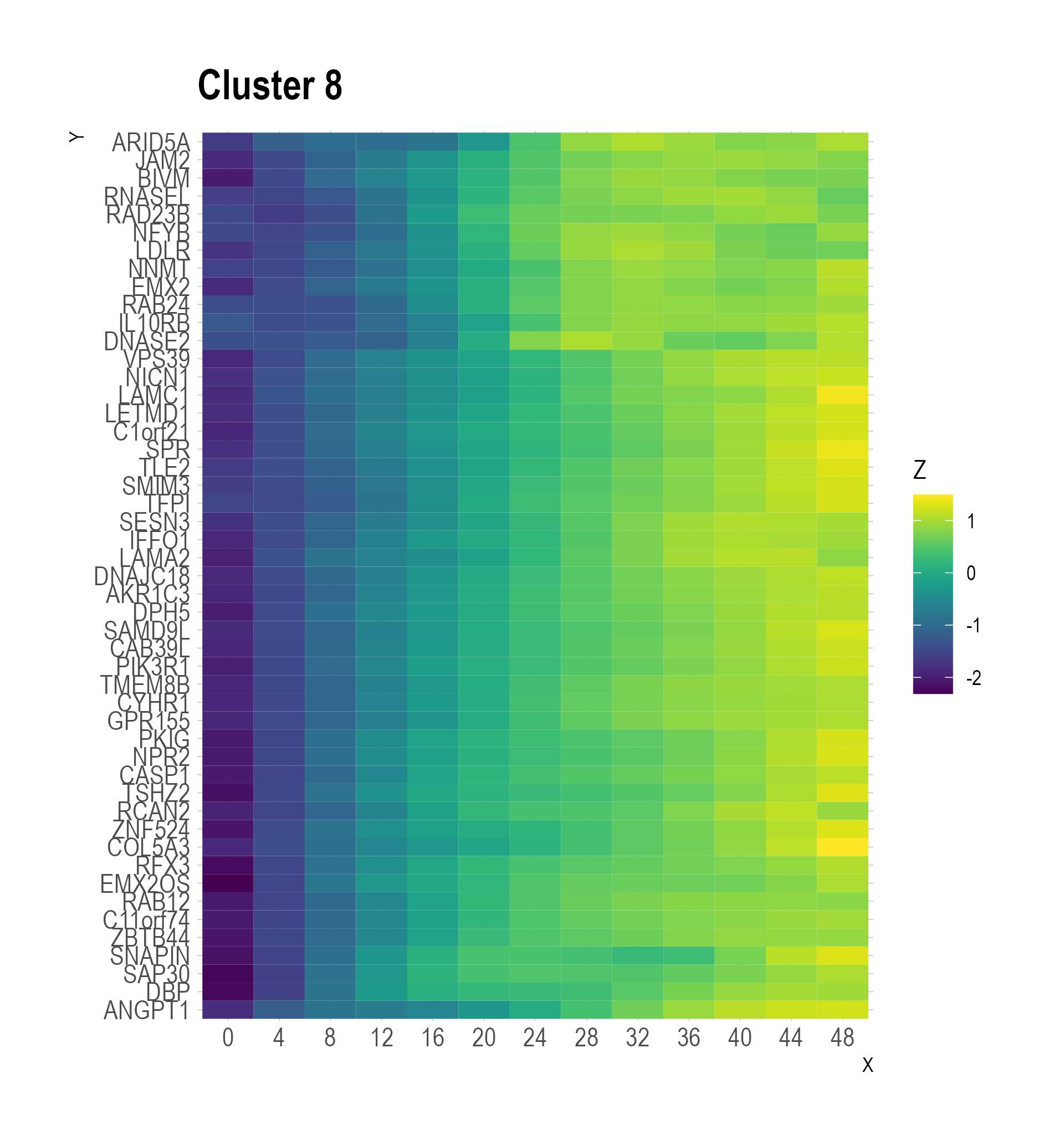

Supplement: Supplementary file 1 — Supplementary file1 (ZIP 1292 kb) [file 335_2024_10050_MOESM1_ESM.zip › All_Circadian_Genes_Heatmap_Clusters/heatmap_cluster 8 .png]

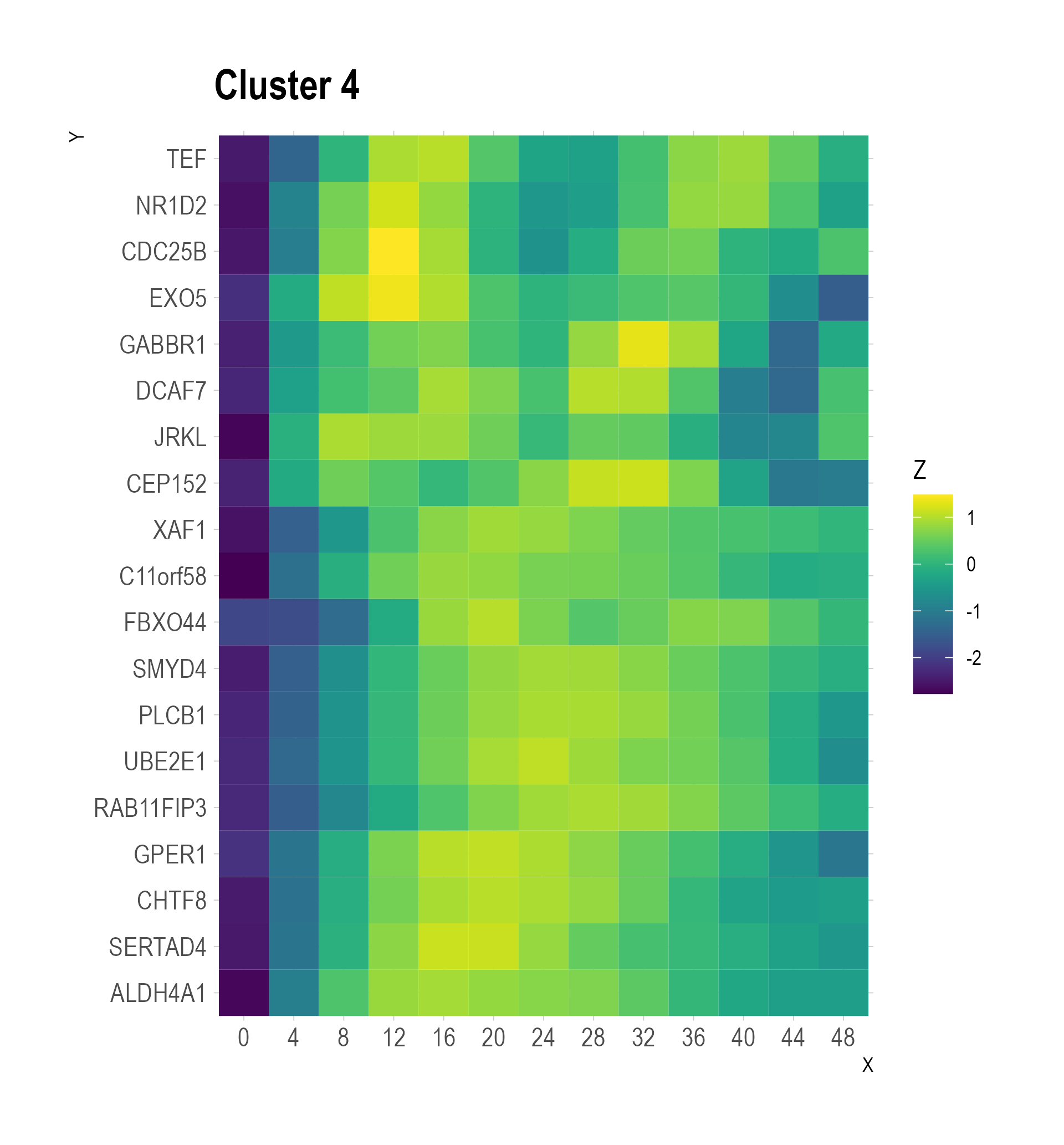

Supplement: Supplementary file 1 — Supplementary file1 (ZIP 1292 kb) [file 335_2024_10050_MOESM1_ESM.zip › All_Circadian_Genes_Heatmap_Clusters/heatmap_cluster 4 .png]

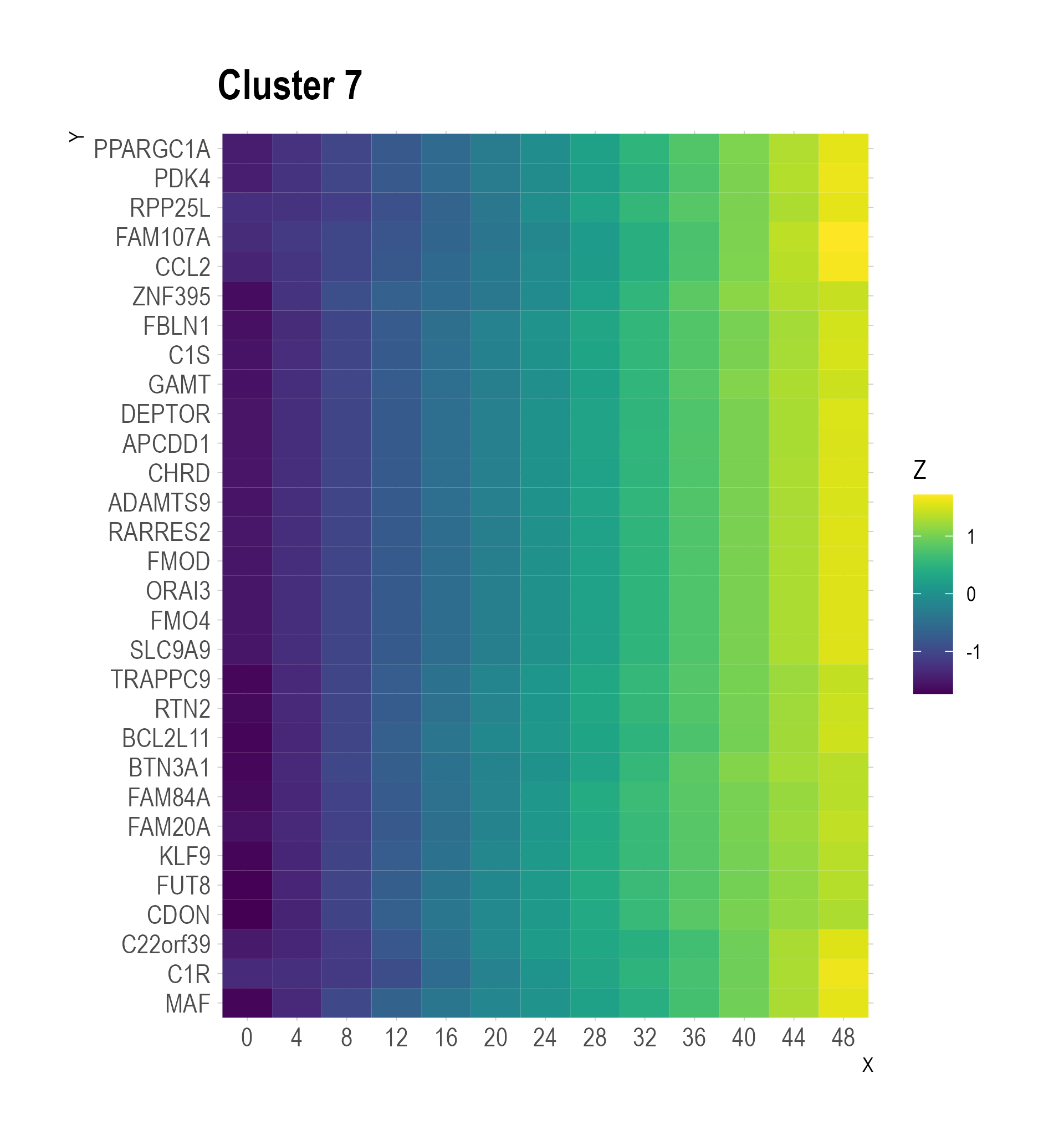

Supplement: Supplementary file 1 — Supplementary file1 (ZIP 1292 kb) [file 335_2024_10050_MOESM1_ESM.zip › All_Circadian_Genes_Heatmap_Clusters/heatmap_cluster 7 .png]

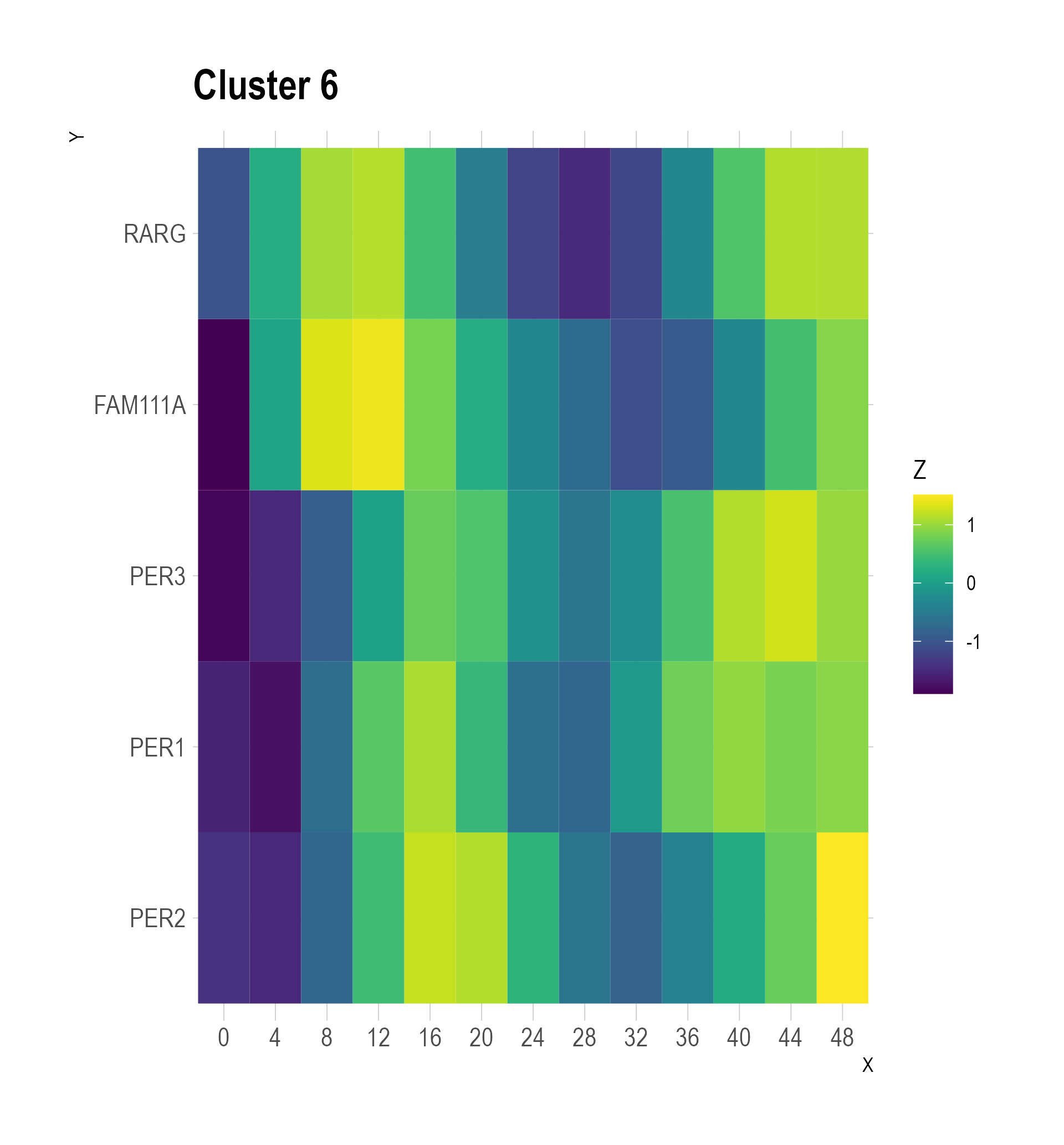

Supplement: Supplementary file 1 — Supplementary file1 (ZIP 1292 kb) [file 335_2024_10050_MOESM1_ESM.zip › All_Circadian_Genes_Heatmap_Clusters/heatmap_cluster 6 .png]

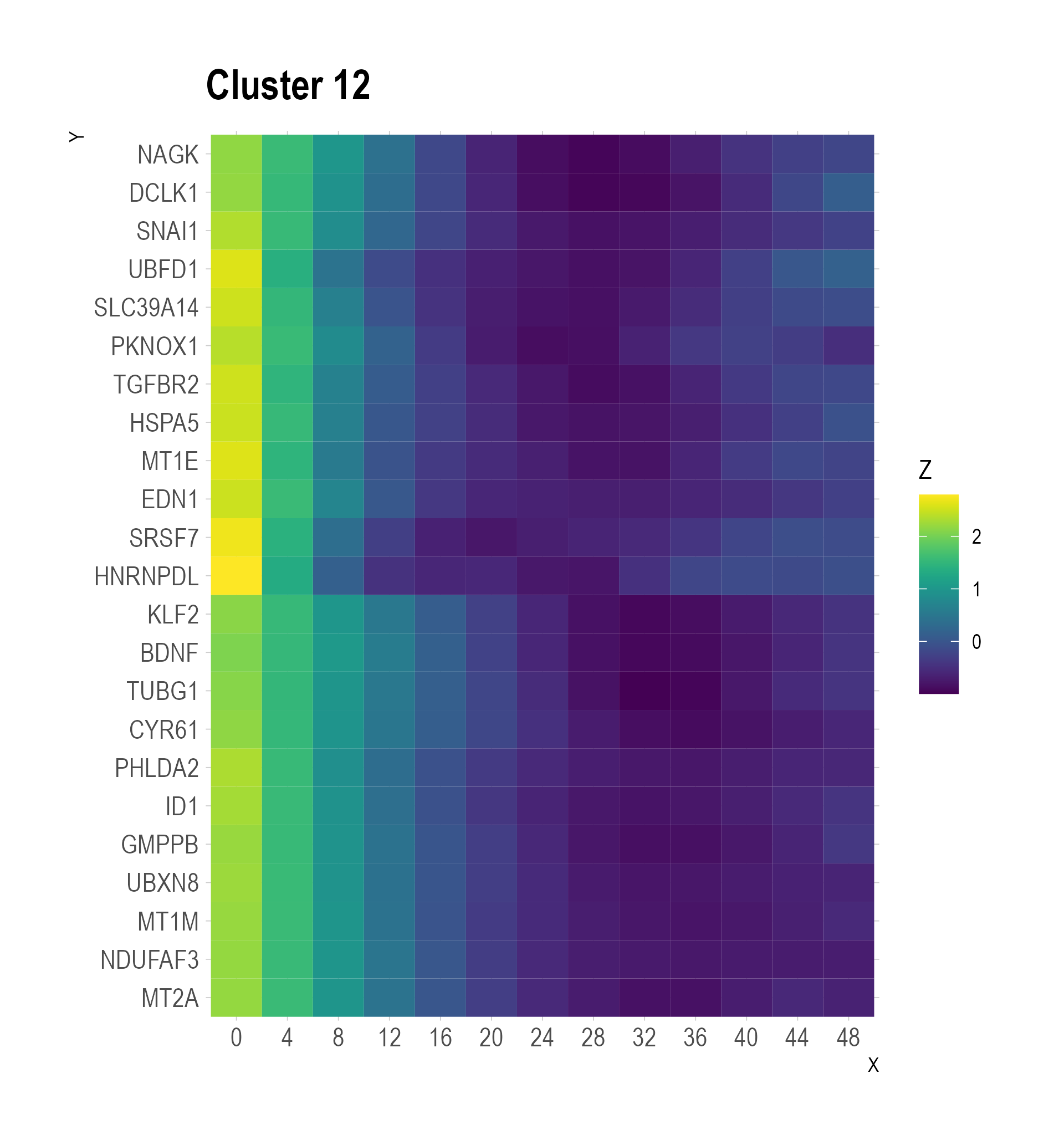

Supplement: Supplementary file 1 — Supplementary file1 (ZIP 1292 kb) [file 335_2024_10050_MOESM1_ESM.zip › All_Circadian_Genes_Heatmap_Clusters/heatmap_cluster 12 .png]

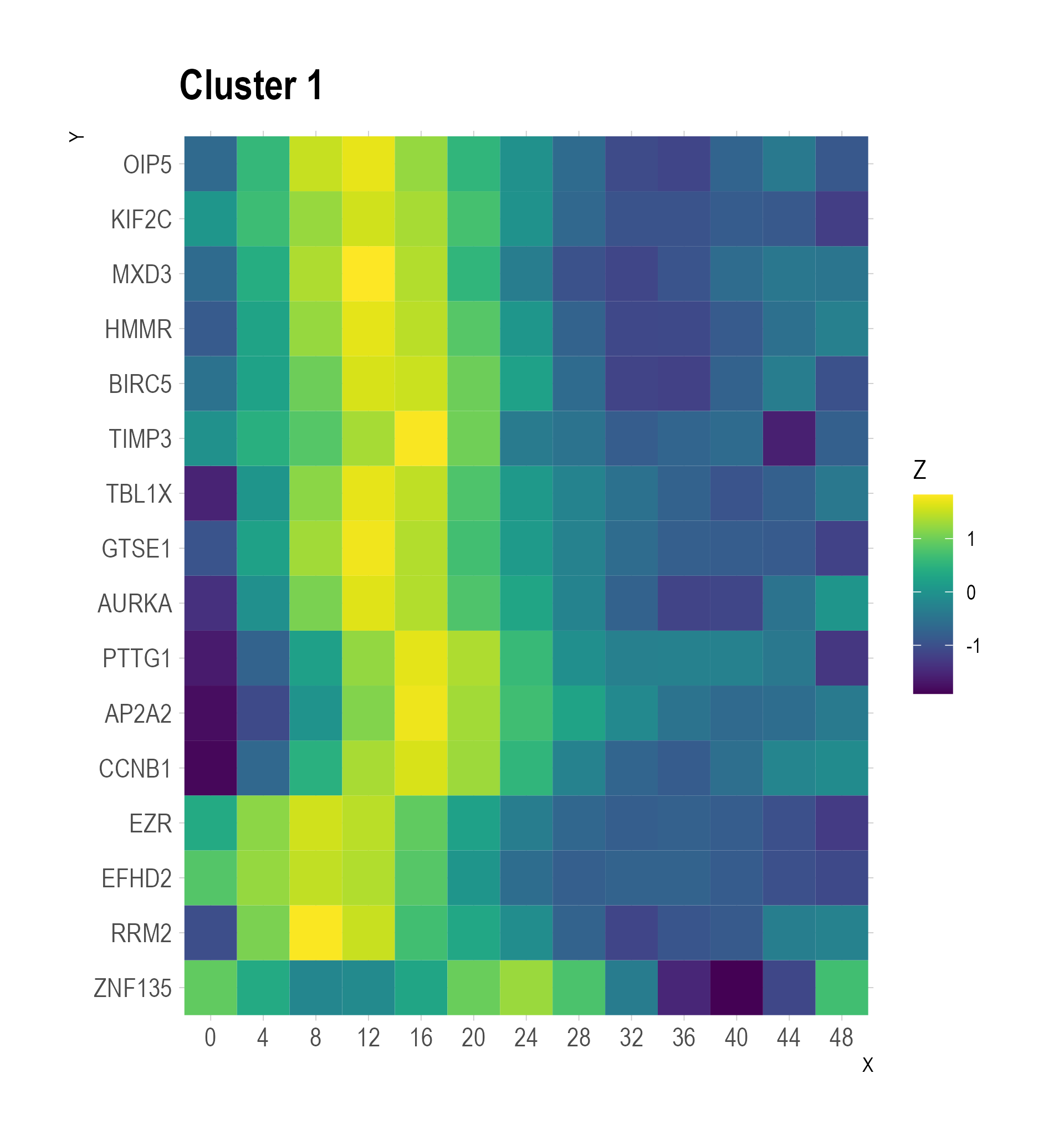

Supplement: Supplementary file 1 — Supplementary file1 (ZIP 1292 kb) [file 335_2024_10050_MOESM1_ESM.zip › All_Circadian_Genes_Heatmap_Clusters/heatmap_cluster 1 .png]

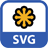

Supplement: Supplementary file 4 — Supplementary file4 (ZIP 16237 kb) [file 335_2024_10050_MOESM4_ESM.zip › icon/SVG48.png]

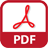

Supplement: Supplementary file 4 — Supplementary file4 (ZIP 16237 kb) [file 335_2024_10050_MOESM4_ESM.zip › icon/PDF48.png]

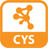

Supplement: Supplementary file 4 — Supplementary file4 (ZIP 16237 kb) [file 335_2024_10050_MOESM4_ESM.zip › icon/CYS48.png]

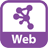

Supplement: Supplementary file 4 — Supplementary file4 (ZIP 16237 kb) [file 335_2024_10050_MOESM4_ESM.zip › icon/WEB_CYS48.png]

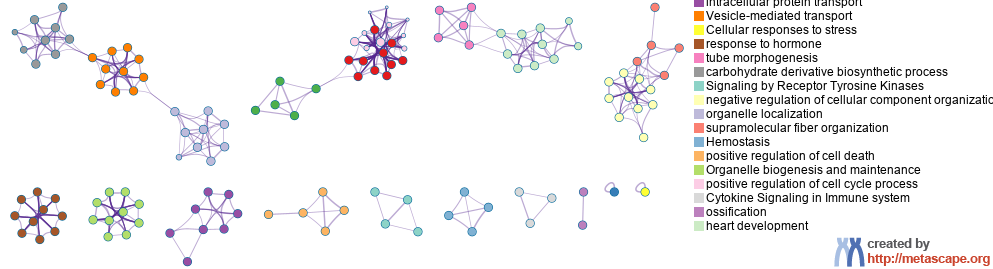

Supplement: Supplementary file 4 — Supplementary file4 (ZIP 16237 kb) [file 335_2024_10050_MOESM4_ESM.zip › Enrichment_GO/ColorByCluster.png]

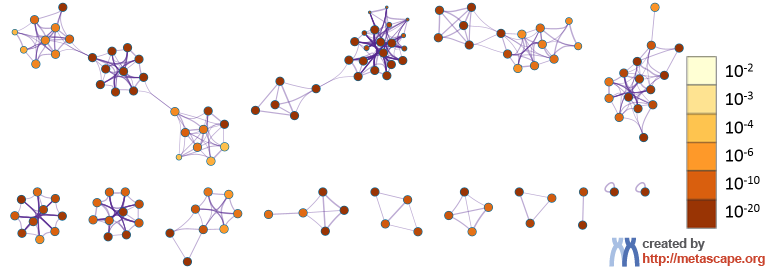

Supplement: Supplementary file 4 — Supplementary file4 (ZIP 16237 kb) [file 335_2024_10050_MOESM4_ESM.zip › Enrichment_GO/ColorByPValue.png]

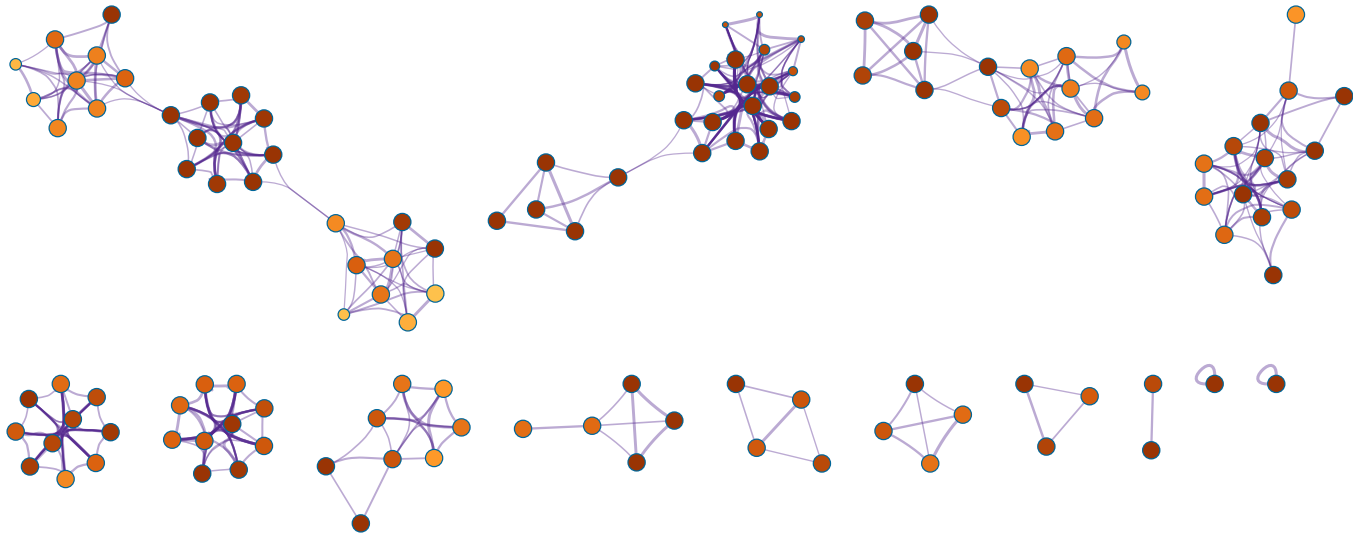

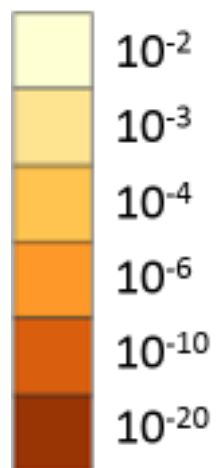

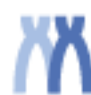 created by  
<http://metascape.org>

Supplement: Supplementary file 4 — Supplementary file4 (ZIP 16237 kb) [file 335_2024_10050_MOESM4_ESM.zip › Enrichment_GO/ColorByPValue.pdf]

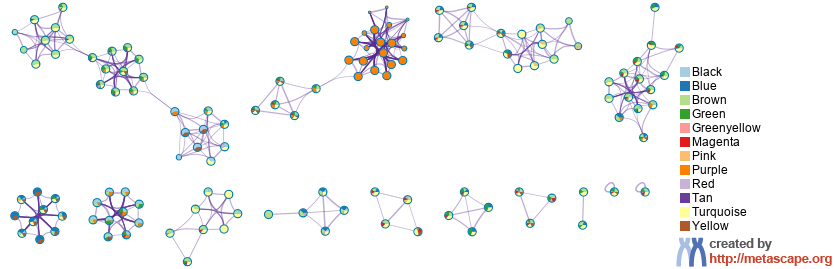

Supplement: Supplementary file 4 — Supplementary file4 (ZIP 16237 kb) [file 335_2024_10050_MOESM4_ESM.zip › Enrichment_GO/ColorByCounts.png]

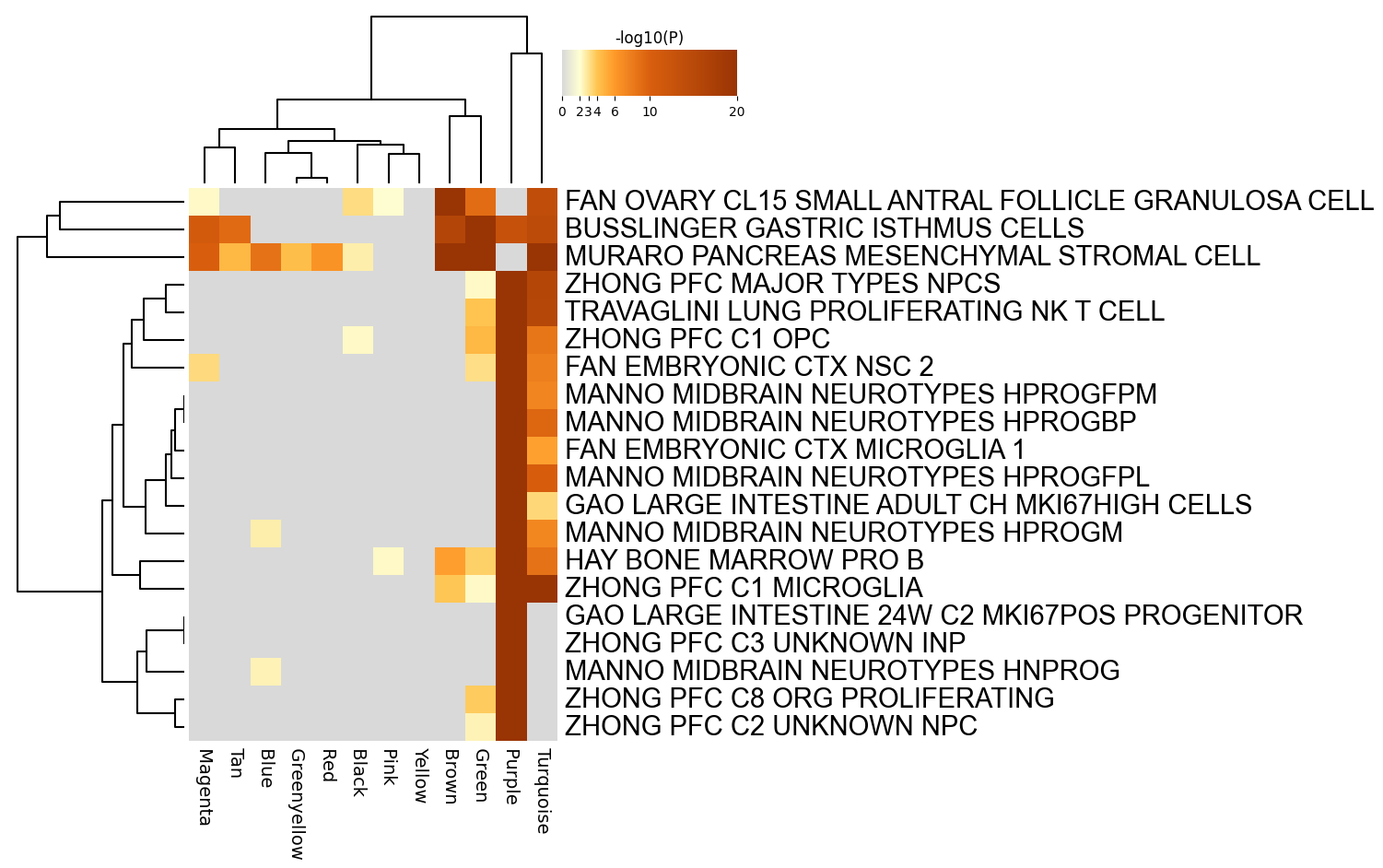

Supplement: Supplementary file 4 — Supplementary file4 (ZIP 16237 kb) [file 335_2024_10050_MOESM4_ESM.zip › Enrichment_QC/HeatmapSelectedGO_Cell_Type_Signatures.png]

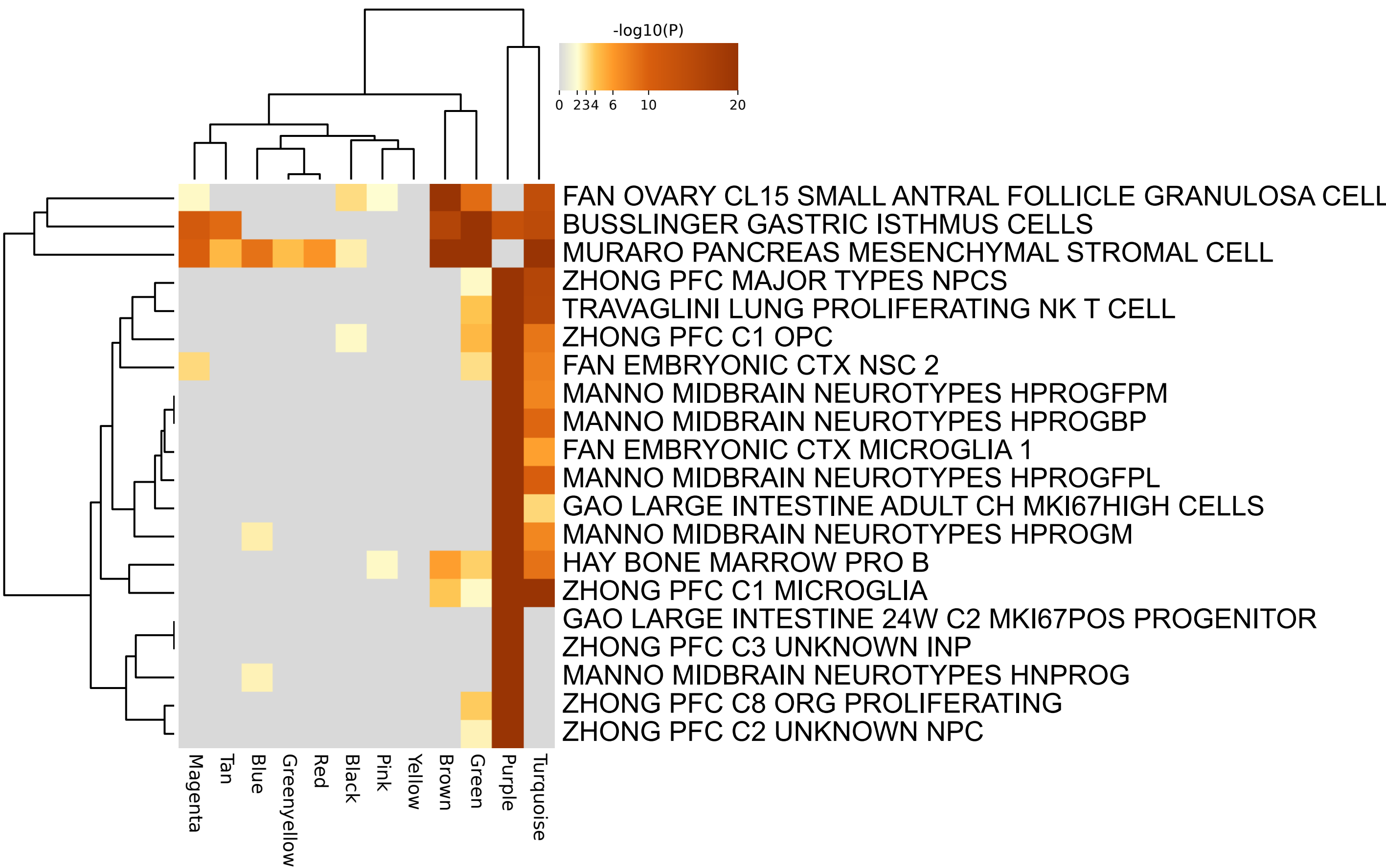

Supplement: Supplementary file 4 — Supplementary file4 (ZIP 16237 kb) [file 335_2024_10050_MOESM4_ESM.zip › Enrichment_QC/HeatmapSelectedGO_Cell_Type_Signatures.pdf]

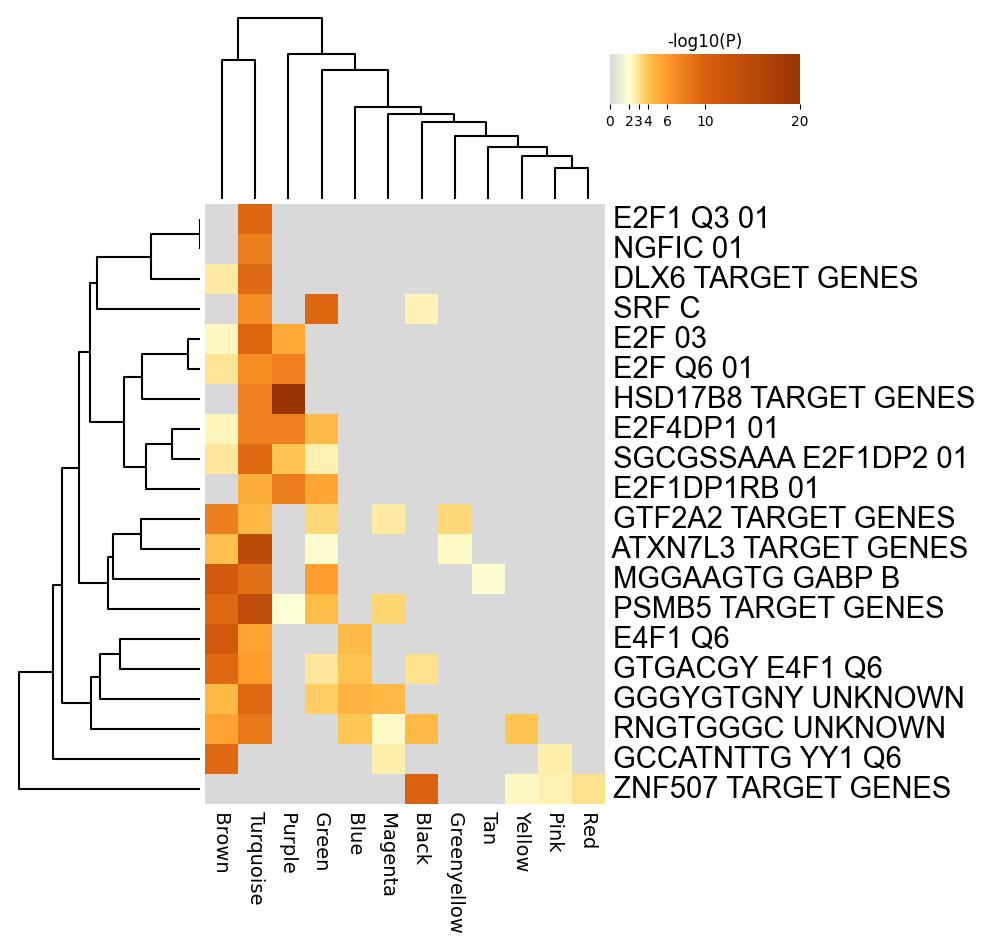

Supplement: Supplementary file 4 — Supplementary file4 (ZIP 16237 kb) [file 335_2024_10050_MOESM4_ESM.zip › Enrichment_QC/HeatmapSelectedGO_Transcription_Factor_Targets.png]

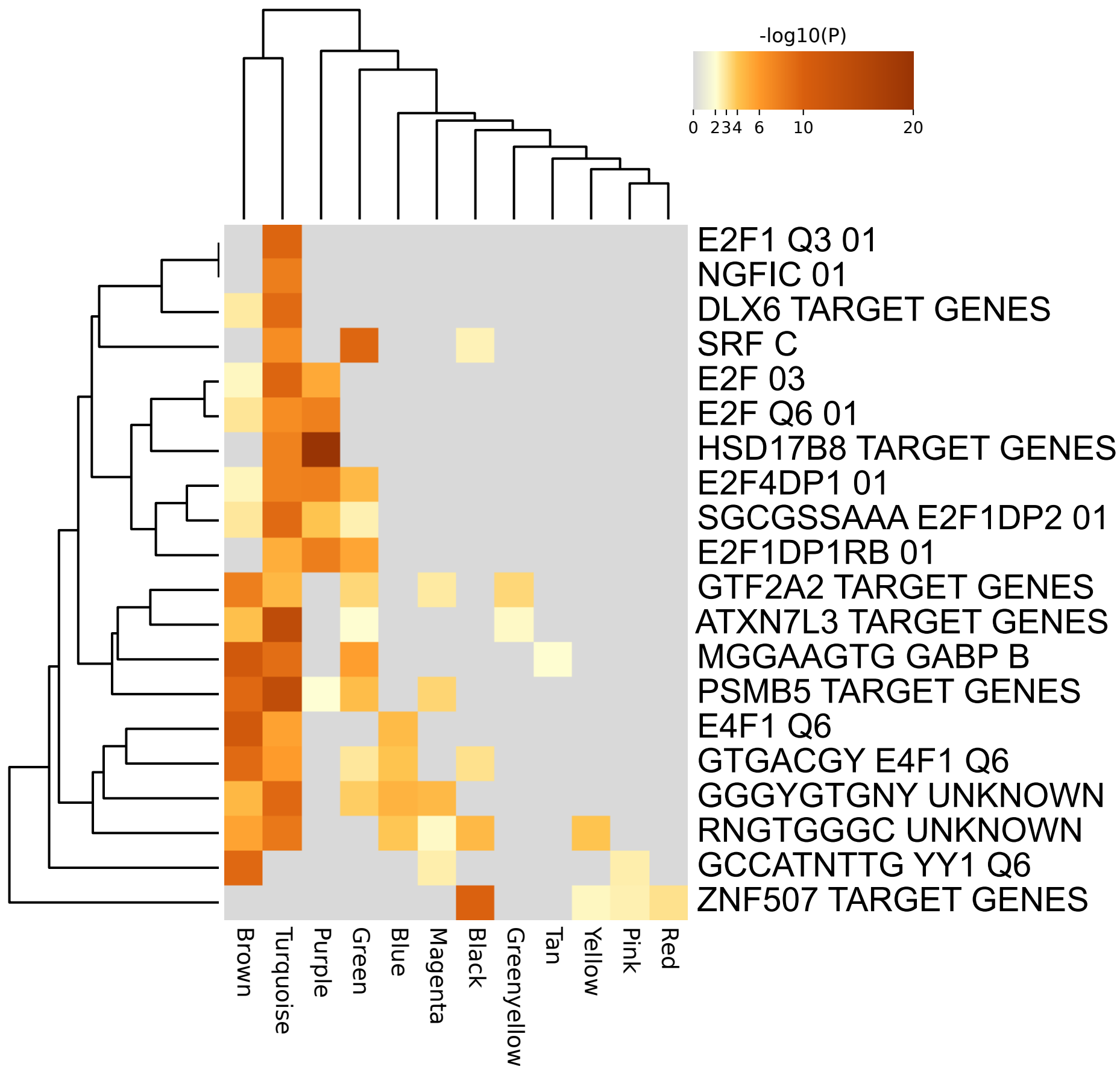

Supplement: Supplementary file 4 — Supplementary file4 (ZIP 16237 kb) [file 335_2024_10050_MOESM4_ESM.zip › Enrichment_QC/HeatmapSelectedGO_Transcription_Factor_Targets.pdf]

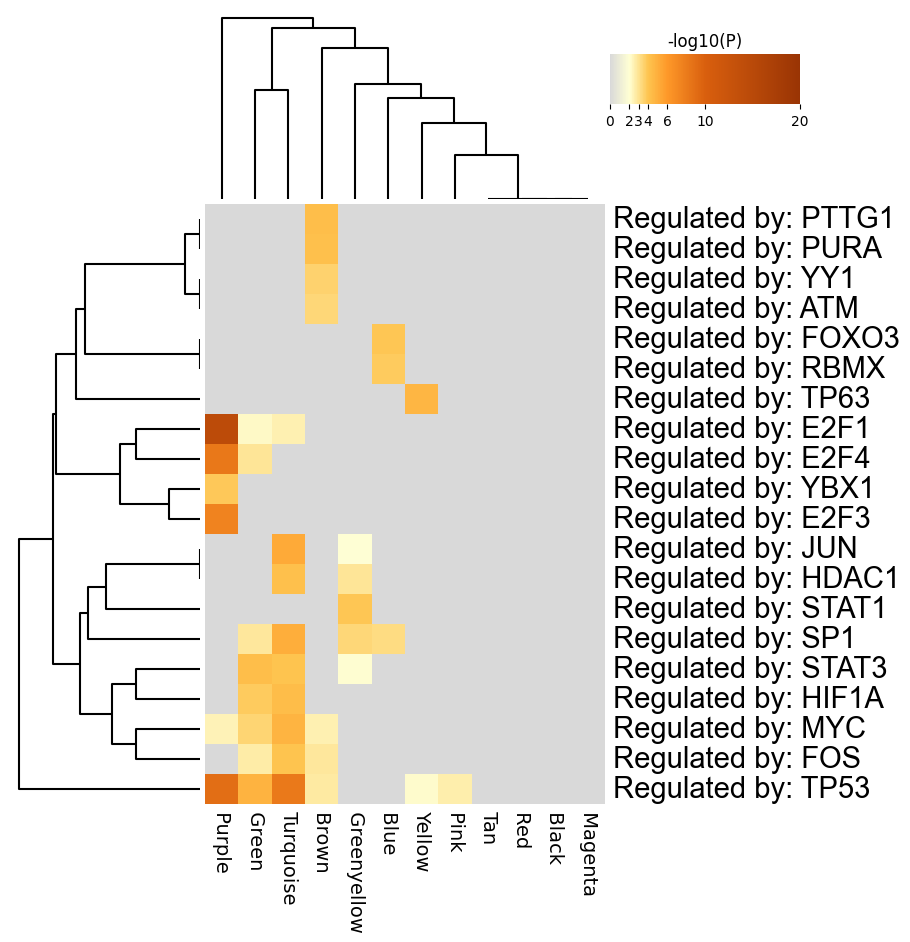

Supplement: Supplementary file 4 — Supplementary file4 (ZIP 16237 kb) [file 335_2024_10050_MOESM4_ESM.zip › Enrichment_QC/HeatmapSelectedGO_TRRUST.png]

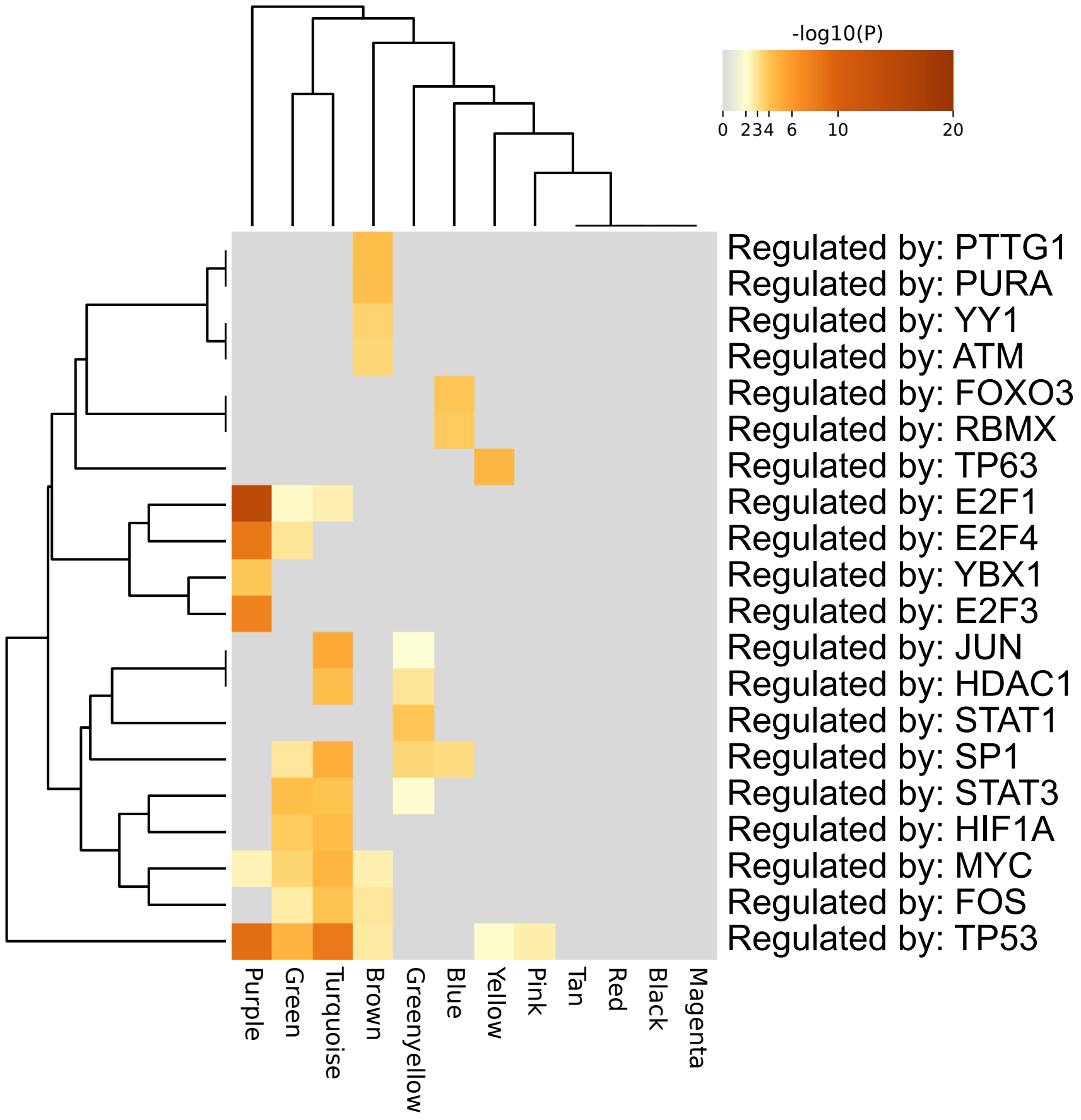

Supplement: Supplementary file 4 — Supplementary file4 (ZIP 16237 kb) [file 335_2024_10050_MOESM4_ESM.zip › Enrichment_QC/HeatmapSelectedGO_TRRUST.pdf]

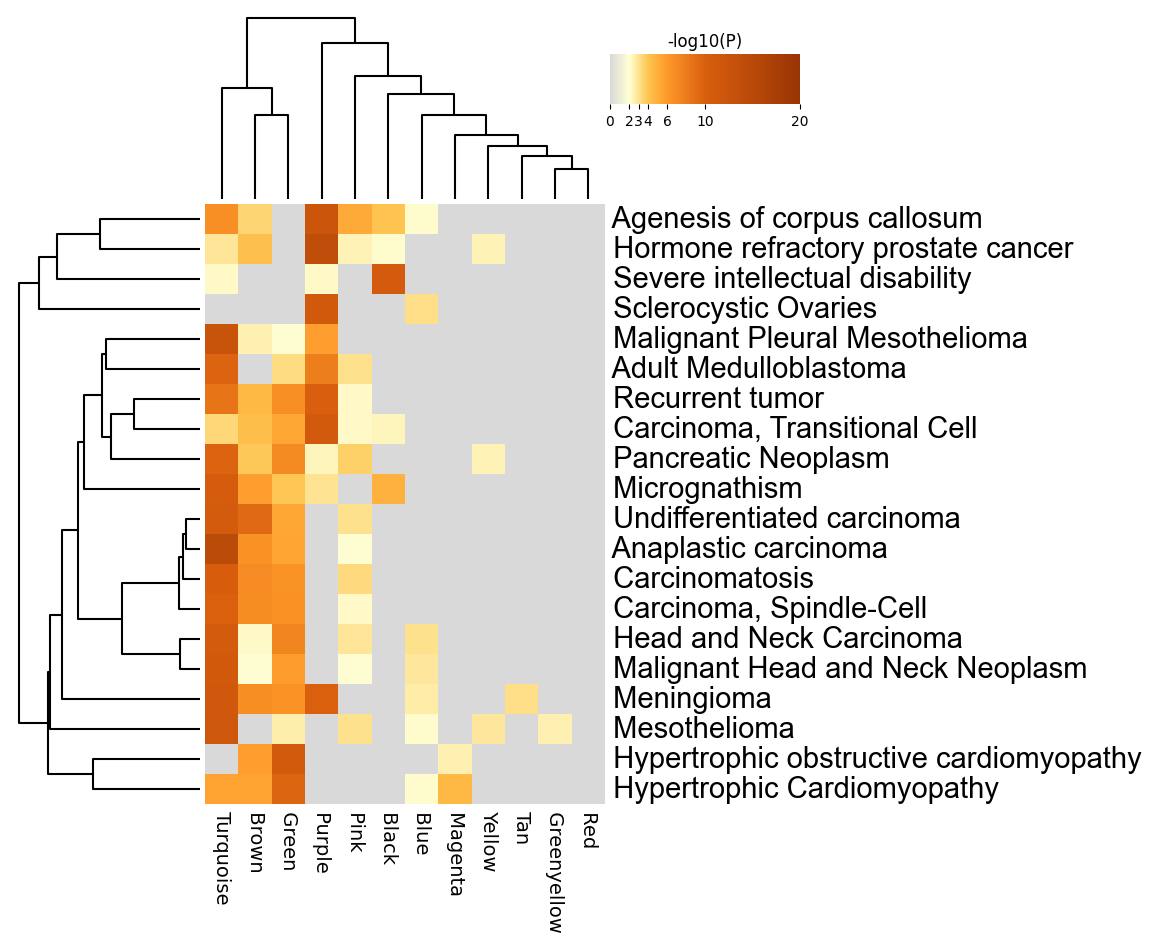

Supplement: Supplementary file 4 — Supplementary file4 (ZIP 16237 kb) [file 335_2024_10050_MOESM4_ESM.zip › Enrichment_QC/HeatmapSelectedGO_DisGeNET.png]

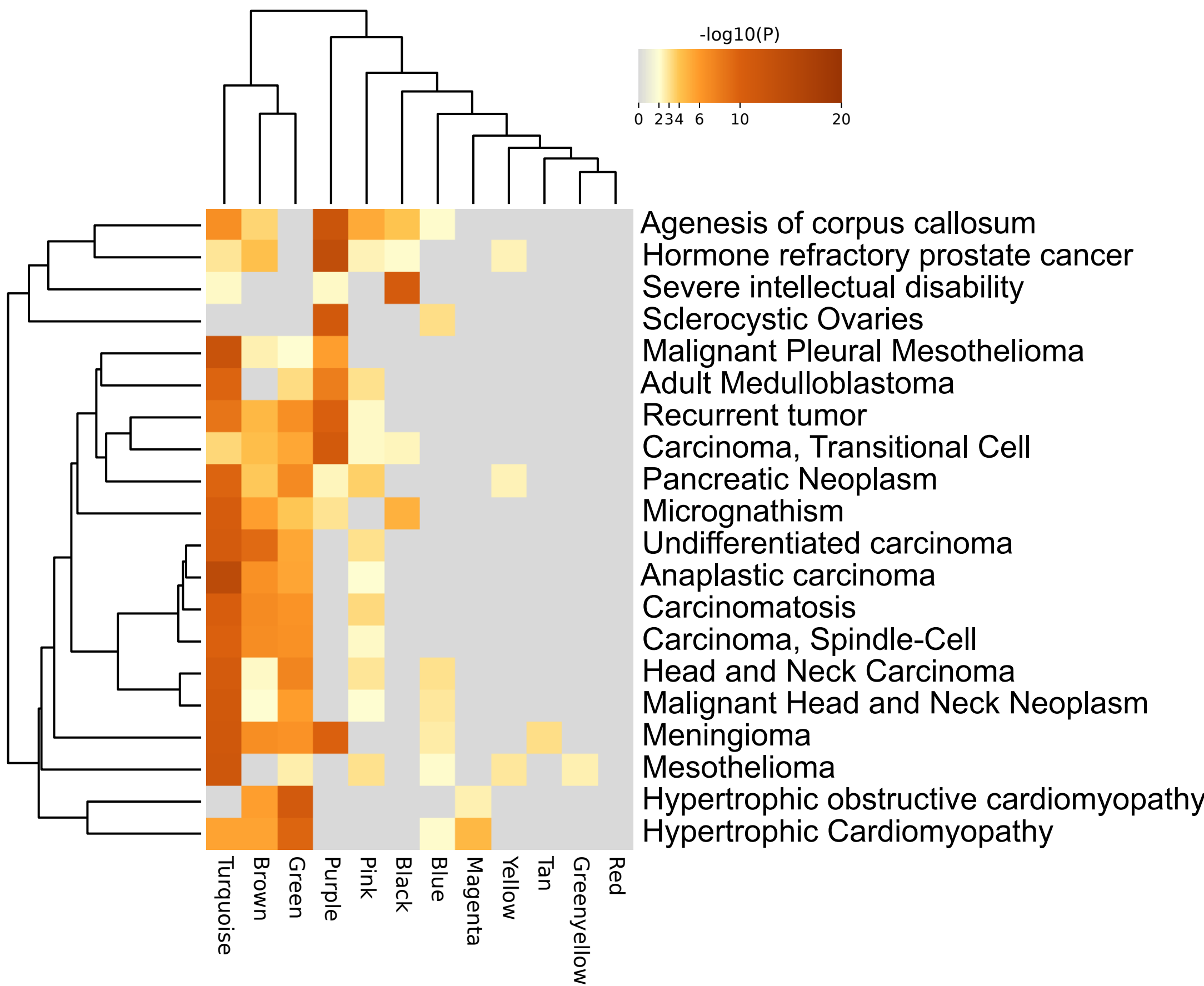

Supplement: Supplementary file 4 — Supplementary file4 (ZIP 16237 kb) [file 335_2024_10050_MOESM4_ESM.zip › Enrichment_QC/HeatmapSelectedGO_DisGeNET.pdf]

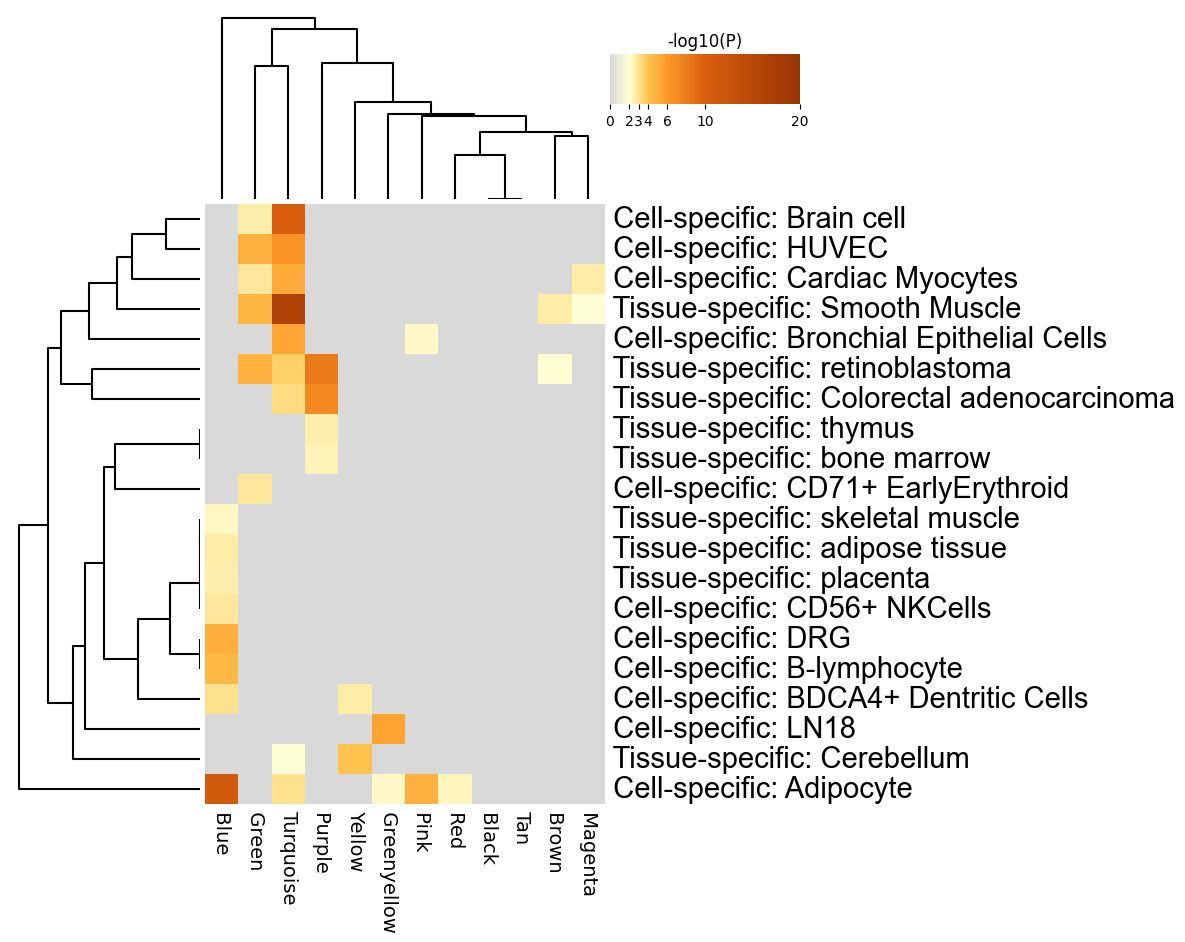

Supplement: Supplementary file 4 — Supplementary file4 (ZIP 16237 kb) [file 335_2024_10050_MOESM4_ESM.zip › Enrichment_QC/HeatmapSelectedGO_PaGenBase.png]

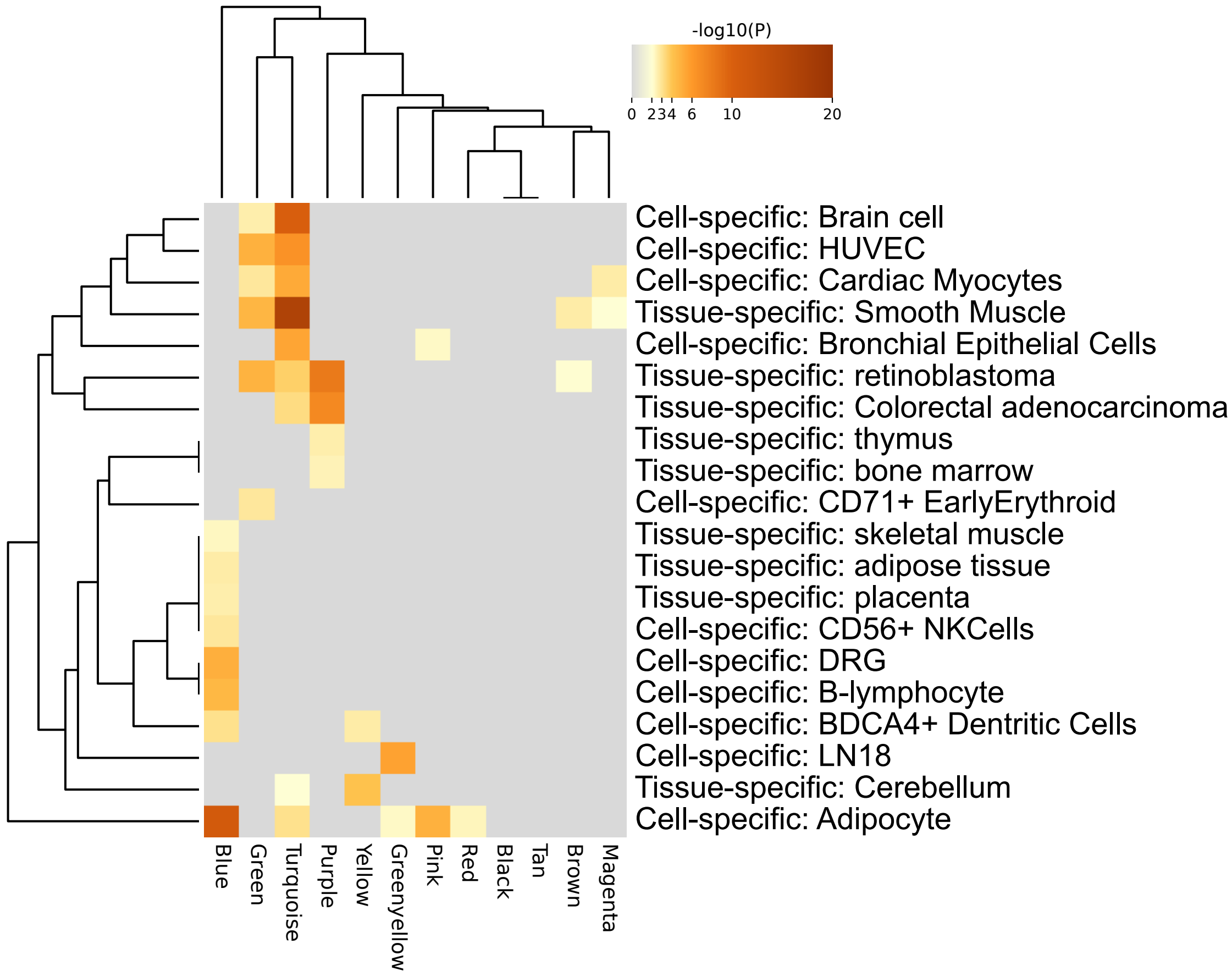

Supplement: Supplementary file 4 — Supplementary file4 (ZIP 16237 kb) [file 335_2024_10050_MOESM4_ESM.zip › Enrichment_QC/HeatmapSelectedGO_PaGenBase.pdf]

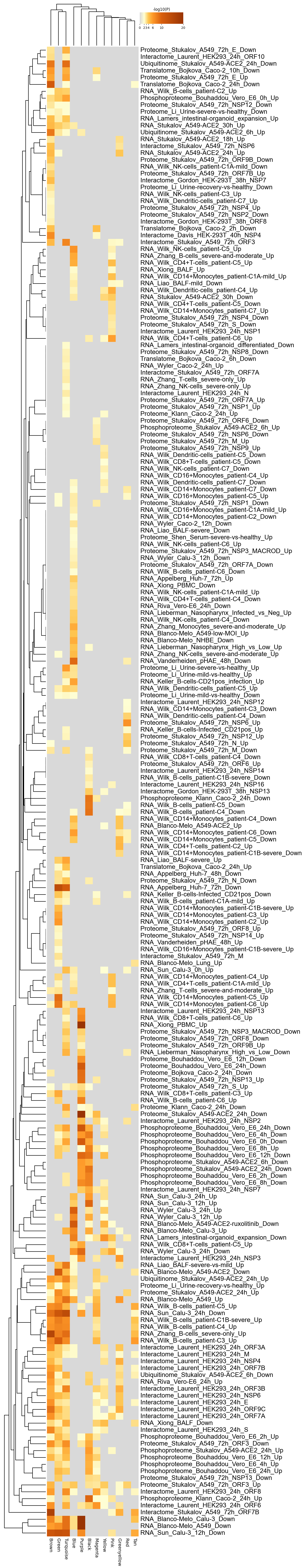

Supplement: Supplementary file 4 — Supplementary file4 (ZIP 16237 kb) [file 335_2024_10050_MOESM4_ESM.zip › Enrichment_QC/HeatmapSelectedGO_COVID.png]

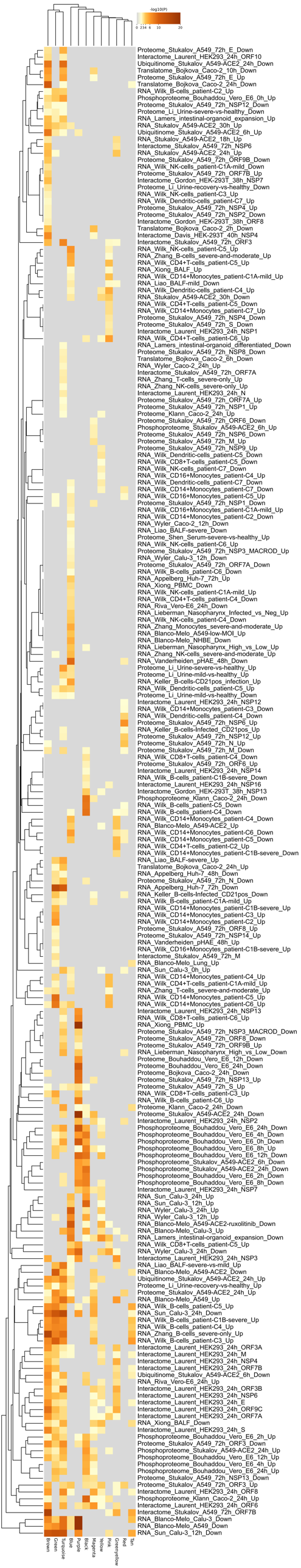

Supplement: Supplementary file 4 — Supplementary file4 (ZIP 16237 kb) [file 335_2024_10050_MOESM4_ESM.zip › Enrichment_QC/HeatmapSelectedGO_COVID.pdf]

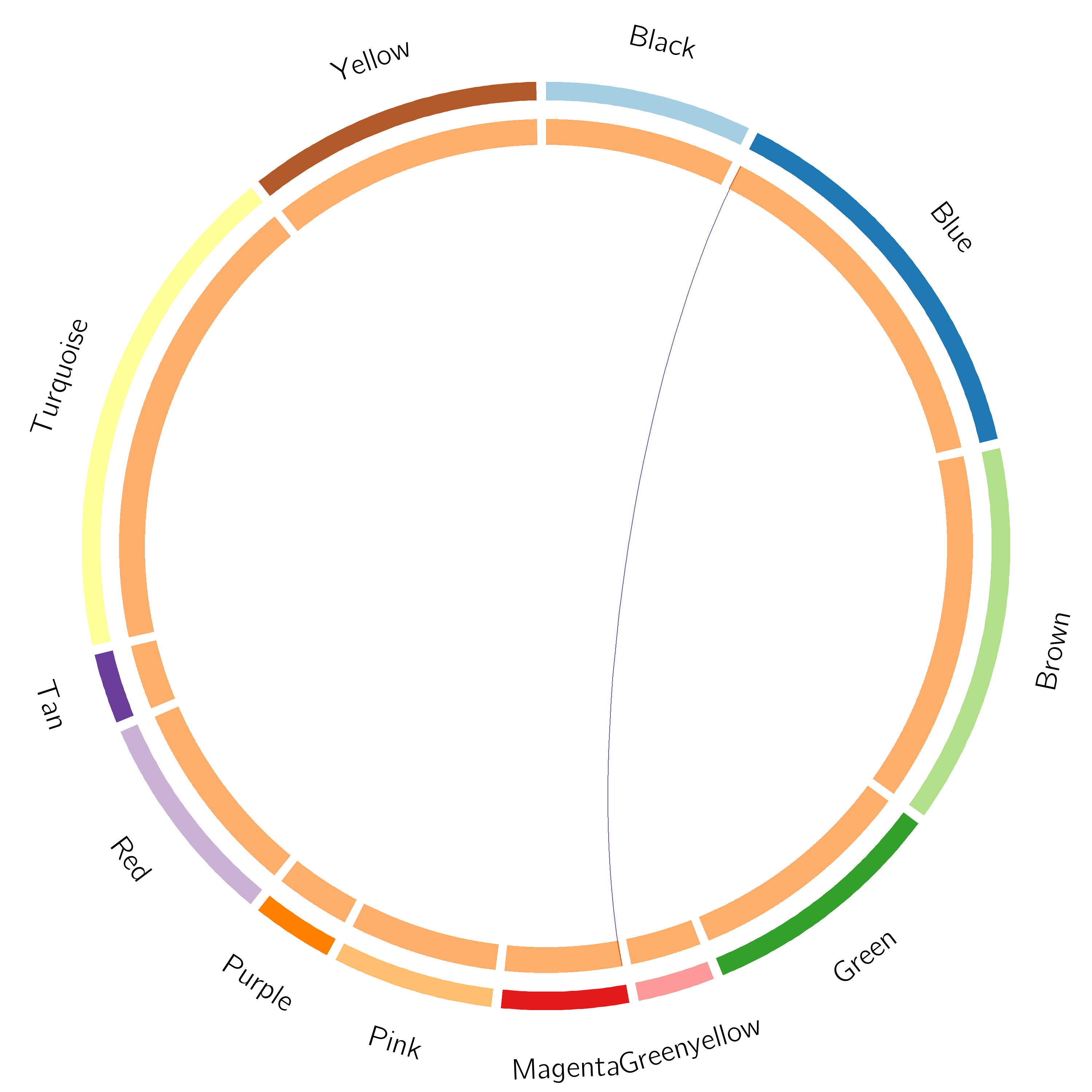

Supplement: Supplementary file 4 — Supplementary file4 (ZIP 16237 kb) [file 335_2024_10050_MOESM4_ESM.zip › Overlap_circos/CircosOverlapByGene.png]

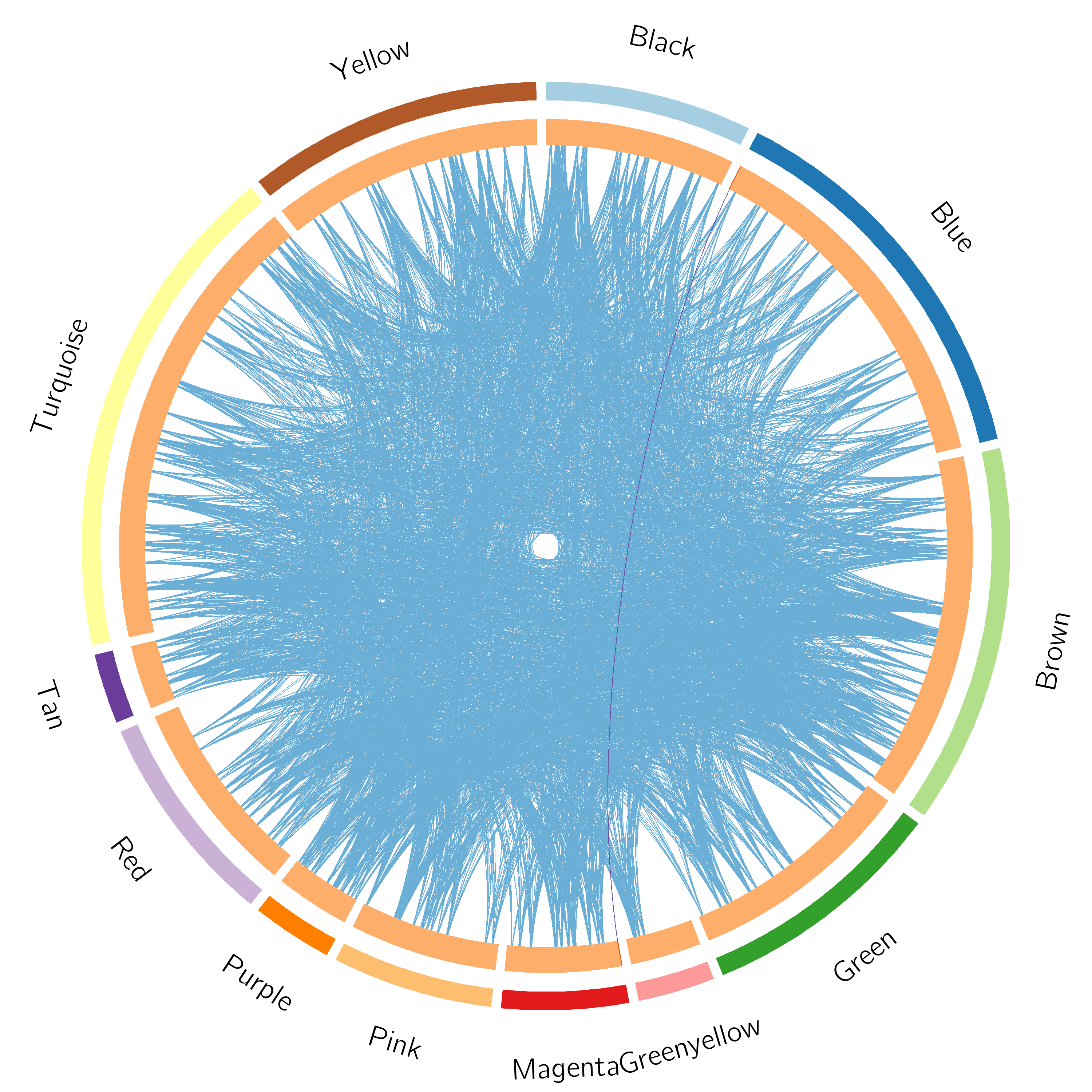

Supplement: Supplementary file 4 — Supplementary file4 (ZIP 16237 kb) [file 335_2024_10050_MOESM4_ESM.zip › Overlap_circos/CircosOverlapByGO.png]

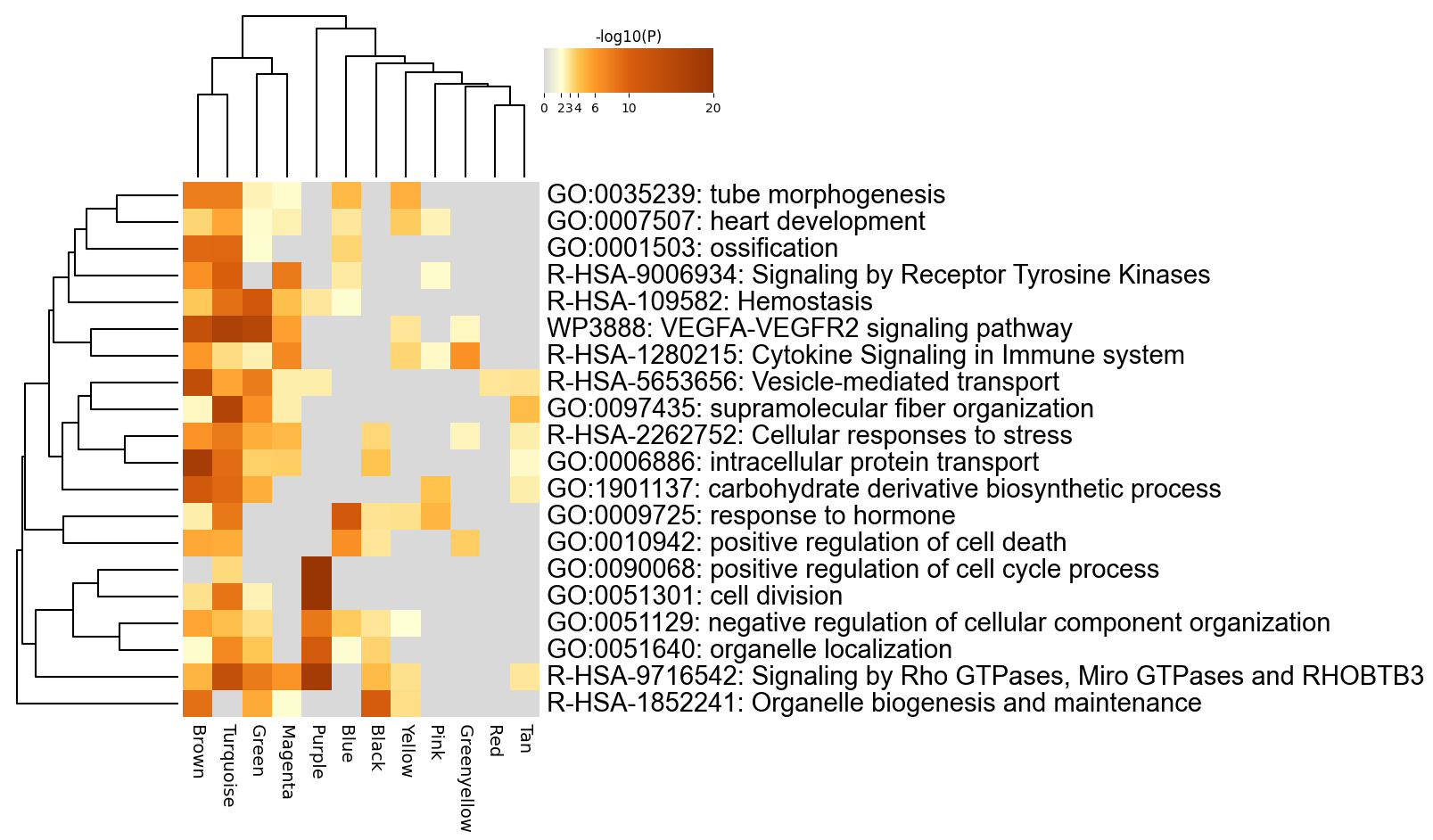

Supplement: Supplementary file 4 — Supplementary file4 (ZIP 16237 kb) [file 335_2024_10050_MOESM4_ESM.zip › Enrichment_heatmap/HeatmapSelectedGO.png]

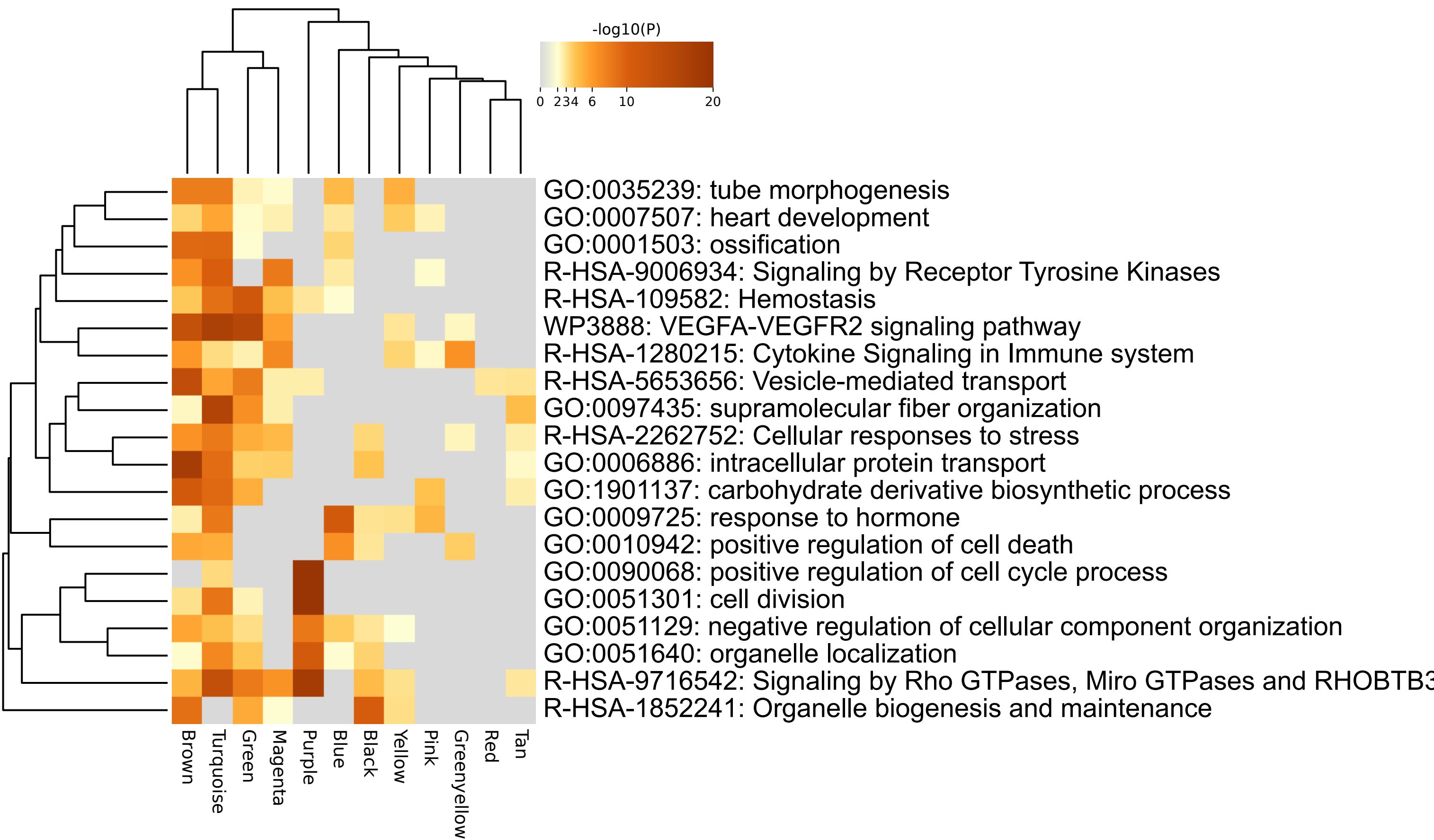

Supplement: Supplementary file 4 — Supplementary file4 (ZIP 16237 kb) [file 335_2024_10050_MOESM4_ESM.zip › Enrichment_heatmap/HeatmapSelectedGO.pdf]

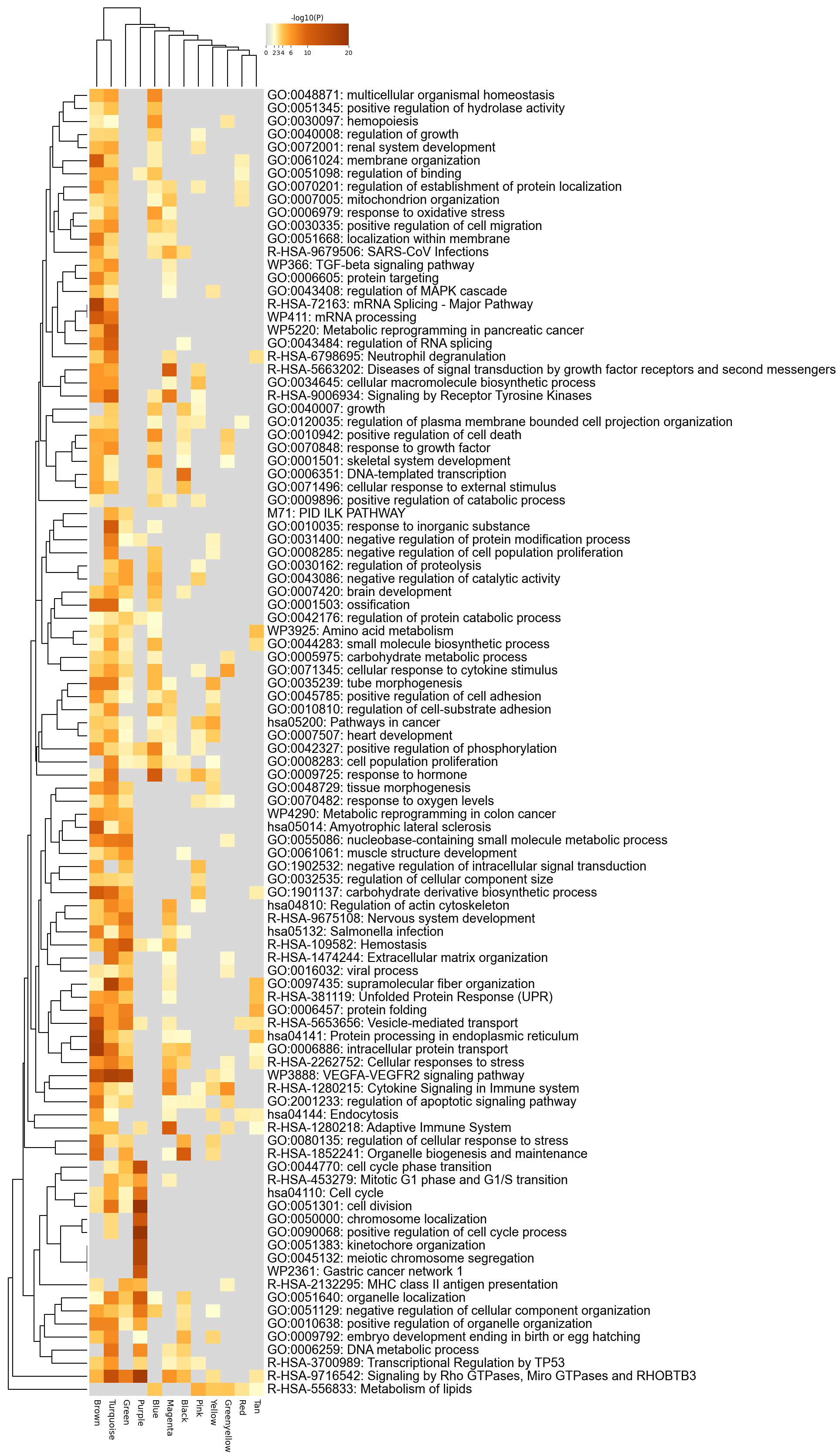

Supplement: Supplementary file 4 — Supplementary file4 (ZIP 16237 kb) [file 335_2024_10050_MOESM4_ESM.zip › Enrichment_heatmap/HeatmapSelectedGOTop100.png]

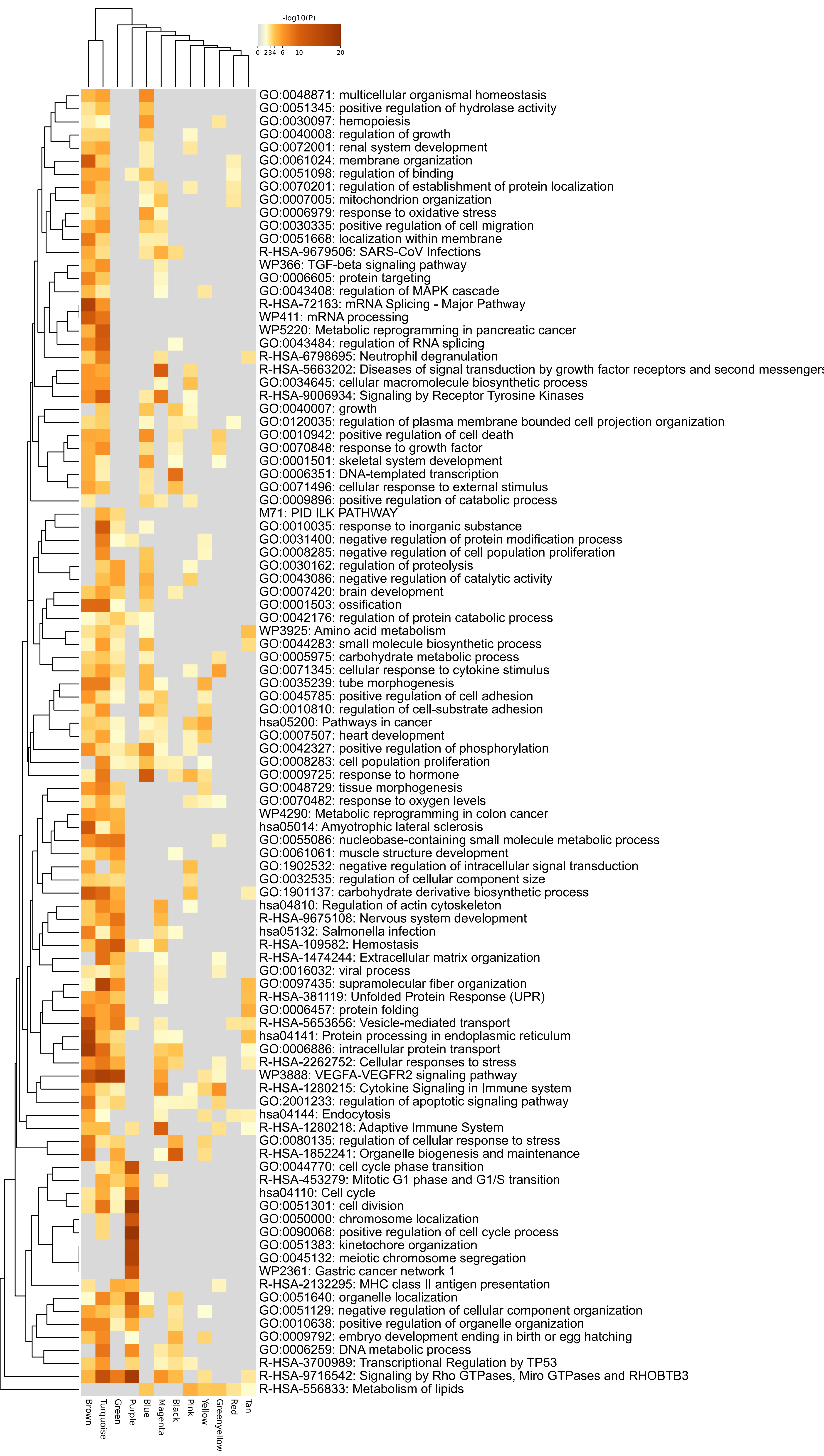

Supplement: Supplementary file 4 — Supplementary file4 (ZIP 16237 kb) [file 335_2024_10050_MOESM4_ESM.zip › Enrichment_heatmap/HeatmapSelectedGOTop100.pdf]

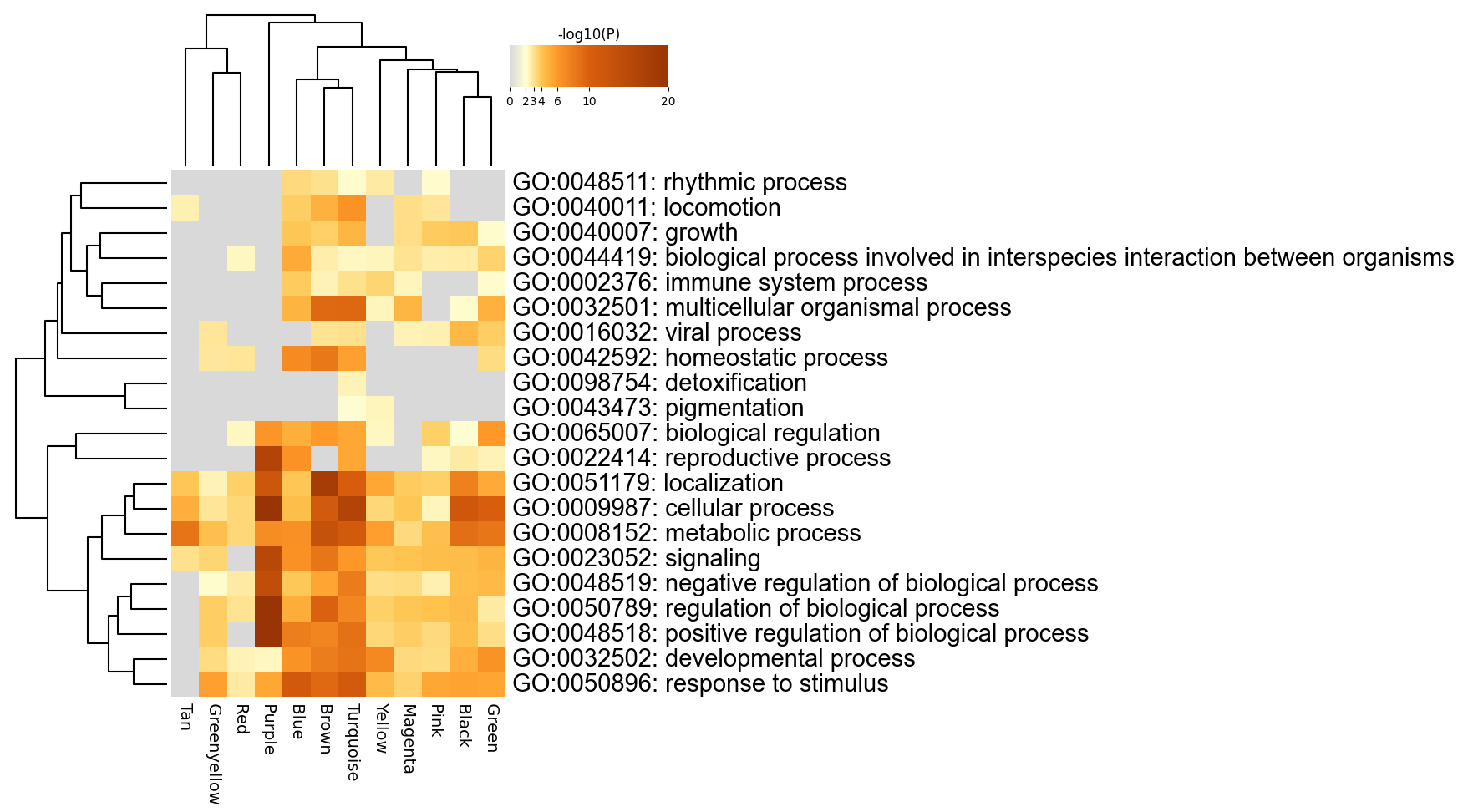

Supplement: Supplementary file 4 — Supplementary file4 (ZIP 16237 kb) [file 335_2024_10050_MOESM4_ESM.zip › Enrichment_heatmap/HeatmapSelectedGOParent.png]

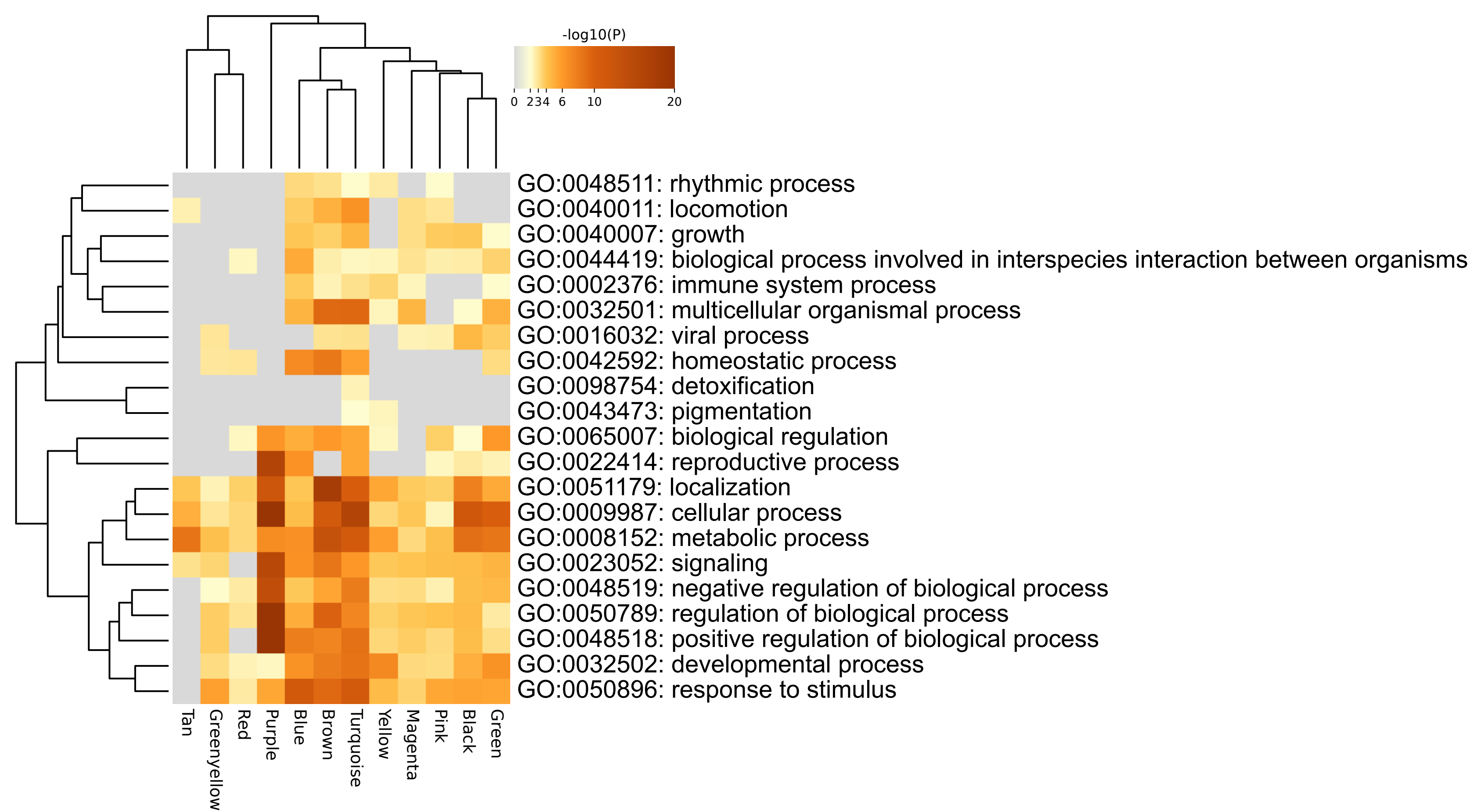

Supplement: Supplementary file 4 — Supplementary file4 (ZIP 16237 kb) [file 335_2024_10050_MOESM4_ESM.zip › Enrichment_heatmap/HeatmapSelectedGOParent.pdf]

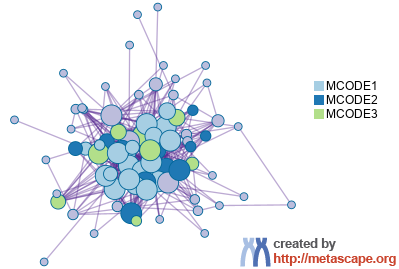

Supplement: Supplementary file 4 — Supplementary file4 (ZIP 16237 kb) [file 335_2024_10050_MOESM4_ESM.zip › Enrichment_PPI/Purple_PPIColorByCluster.png]

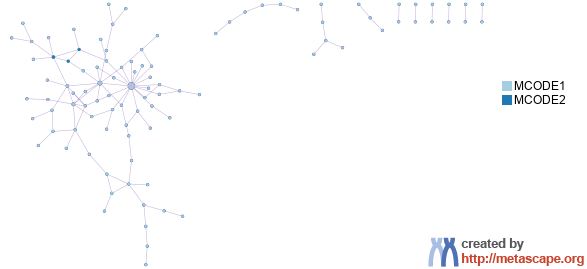

Supplement: Supplementary file 4 — Supplementary file4 (ZIP 16237 kb) [file 335_2024_10050_MOESM4_ESM.zip › Enrichment_PPI/Red_PPIColorByCluster.png]

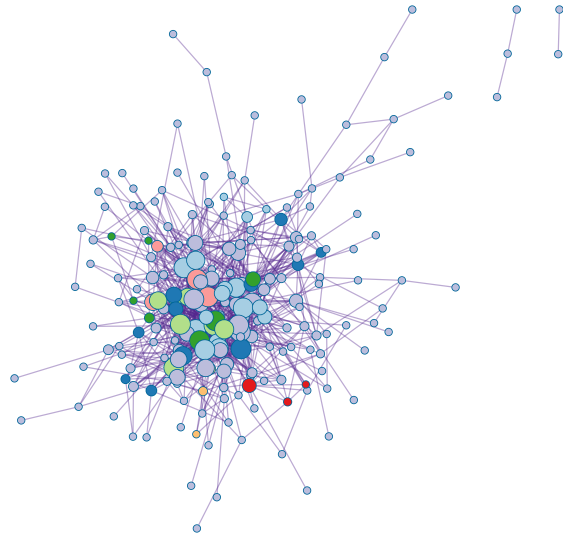

■ MCODE1  
■ MCODE2  
■ MCODE3  
■ MCODE4  
■ MCODE5  
■ MCODE6  
■ MCODE7

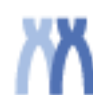 created by  
<http://metascape.org>

Supplement: Supplementary file 4 — Supplementary file4 (ZIP 16237 kb) [file 335_2024_10050_MOESM4_ESM.zip › Enrichment_PPI/Green_PPIColorByCluster.pdf]

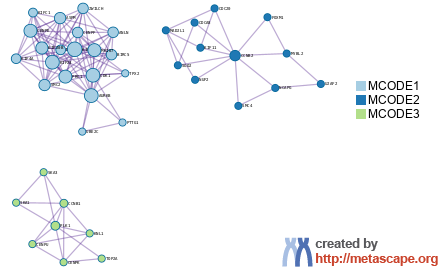

Supplement: Supplementary file 4 — Supplementary file4 (ZIP 16237 kb) [file 335_2024_10050_MOESM4_ESM.zip › Enrichment_PPI/Purple_MCODE_ALL_PPIColorByCluster.png]

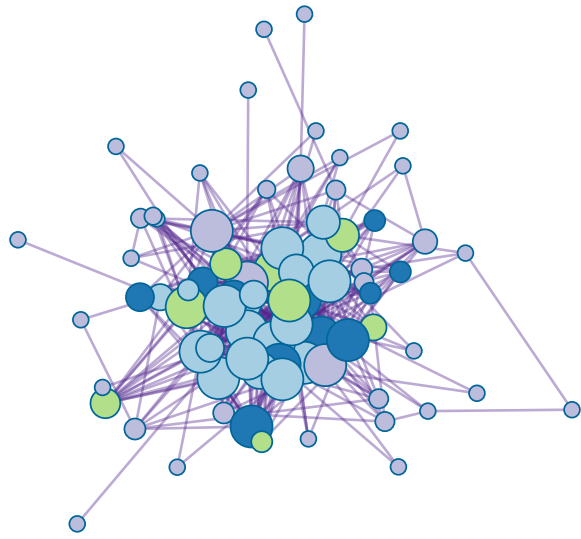

■ MCODE1  
■ MCODE2  
■ MCODE3

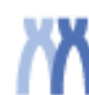 created by  
<http://metascape.org>

Supplement: Supplementary file 4 — Supplementary file4 (ZIP 16237 kb) [file 335_2024_10050_MOESM4_ESM.zip › Enrichment_PPI/Purple_PPIColorByCluster.pdf]

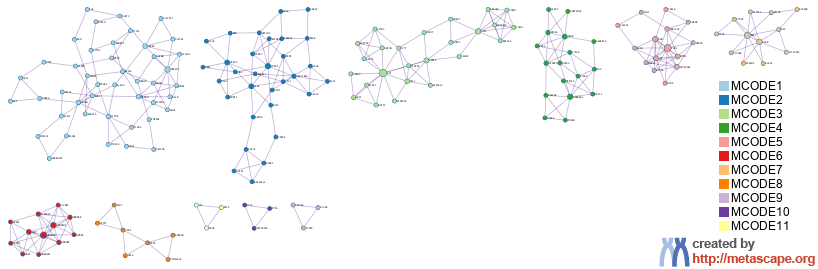

Supplement: Supplementary file 4 — Supplementary file4 (ZIP 16237 kb) [file 335_2024_10050_MOESM4_ESM.zip › Enrichment_PPI/Turquoise_MCODE_ALL_PPIColorByCluster.png]

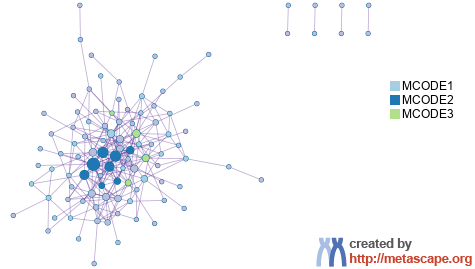

Supplement: Supplementary file 4 — Supplementary file4 (ZIP 16237 kb) [file 335_2024_10050_MOESM4_ESM.zip › Enrichment_PPI/Magenta_PPIColorByCluster.png]

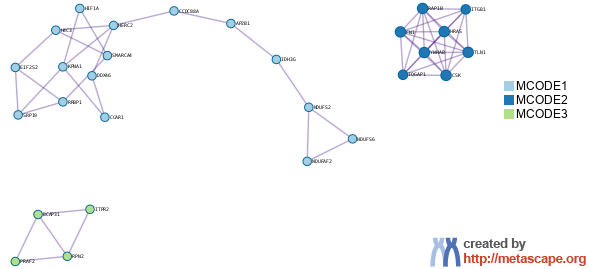

Supplement: Supplementary file 4 — Supplementary file4 (ZIP 16237 kb) [file 335_2024_10050_MOESM4_ESM.zip › Enrichment_PPI/Magenta_MCODE_ALL_PPIColorByCluster.png]

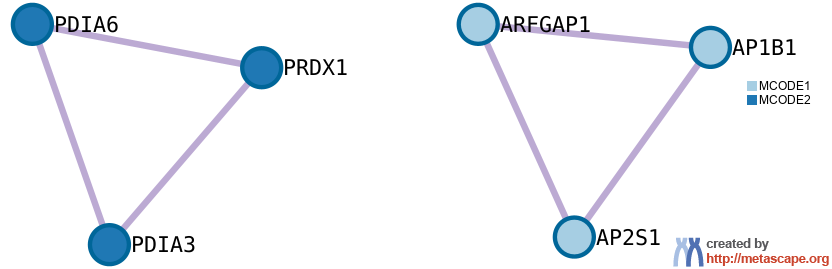

Supplement: Supplementary file 4 — Supplementary file4 (ZIP 16237 kb) [file 335_2024_10050_MOESM4_ESM.zip › Enrichment_PPI/Tan_MCODE_ALL_PPIColorByCluster.png]

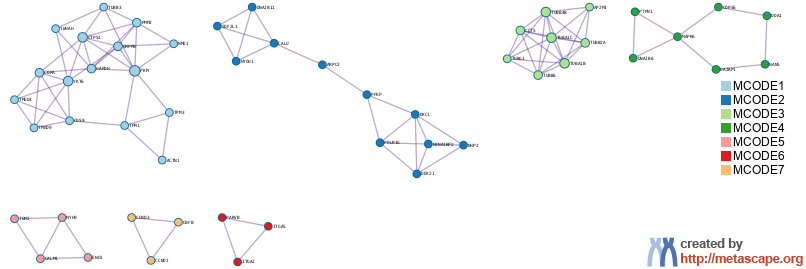

Supplement: Supplementary file 4 — Supplementary file4 (ZIP 16237 kb) [file 335_2024_10050_MOESM4_ESM.zip › Enrichment_PPI/Green_MCODE_ALL_PPIColorByCluster.png]

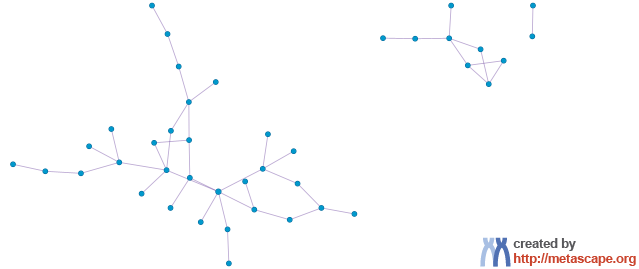

Supplement: Supplementary file 4 — Supplementary file4 (ZIP 16237 kb) [file 335_2024_10050_MOESM4_ESM.zip › Enrichment_PPI/Greenyellow_PPIColorByCluster.png]

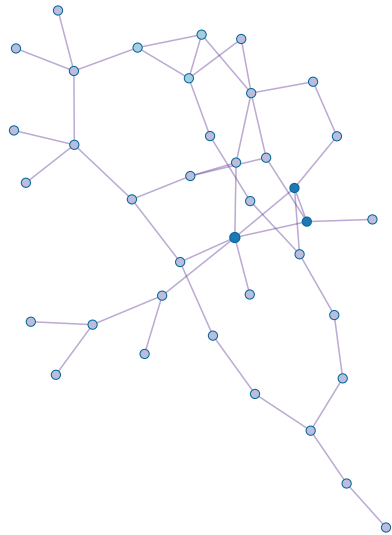

■ MCODE1  
■ MCODE2

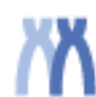 created by  
<http://metascape.org>

Supplement: Supplementary file 4 — Supplementary file4 (ZIP 16237 kb) [file 335_2024_10050_MOESM4_ESM.zip › Enrichment_PPI/Tan_PPIColorByCluster.pdf]

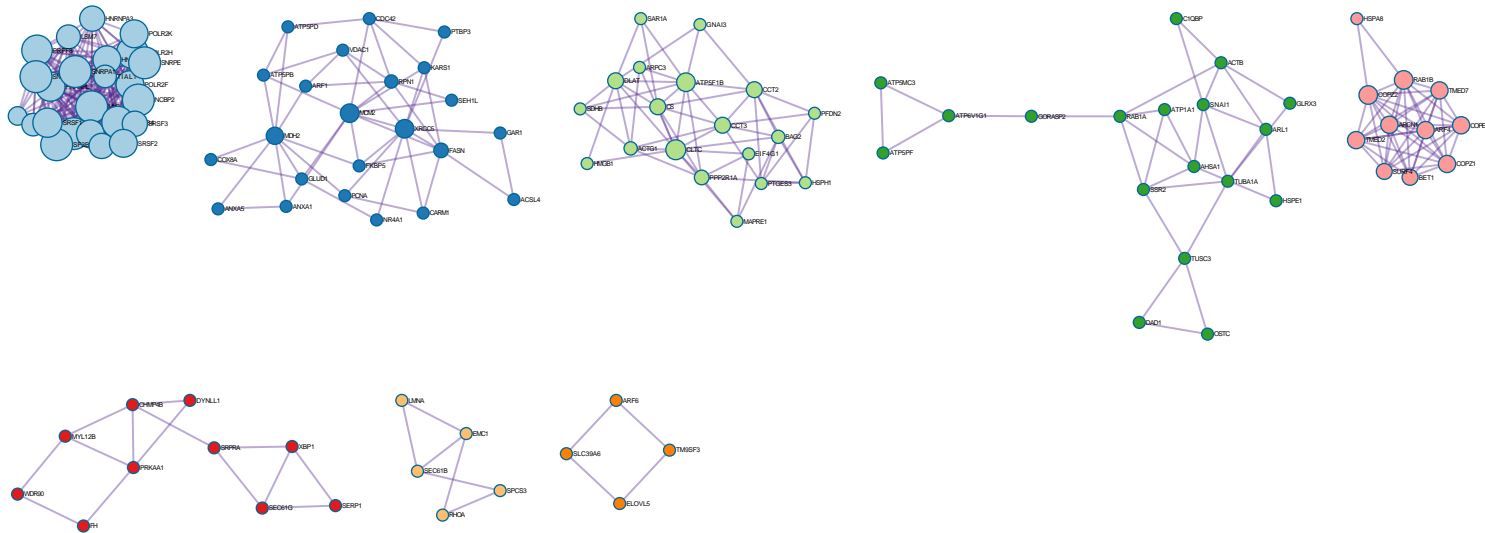

■ MCODE1  
■ MCODE2  
■ MCODE3  
■ MCODE4  
■ MCODE5  
■ MCODE6  
■ MCODE7  
■ MCODE8

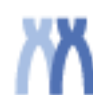 created by  
<http://metascape.org>

Supplement: Supplementary file 4 — Supplementary file4 (ZIP 16237 kb) [file 335_2024_10050_MOESM4_ESM.zip › Enrichment_PPI/Brown_MCODE_ALL_PPIColorByCluster.pdf]

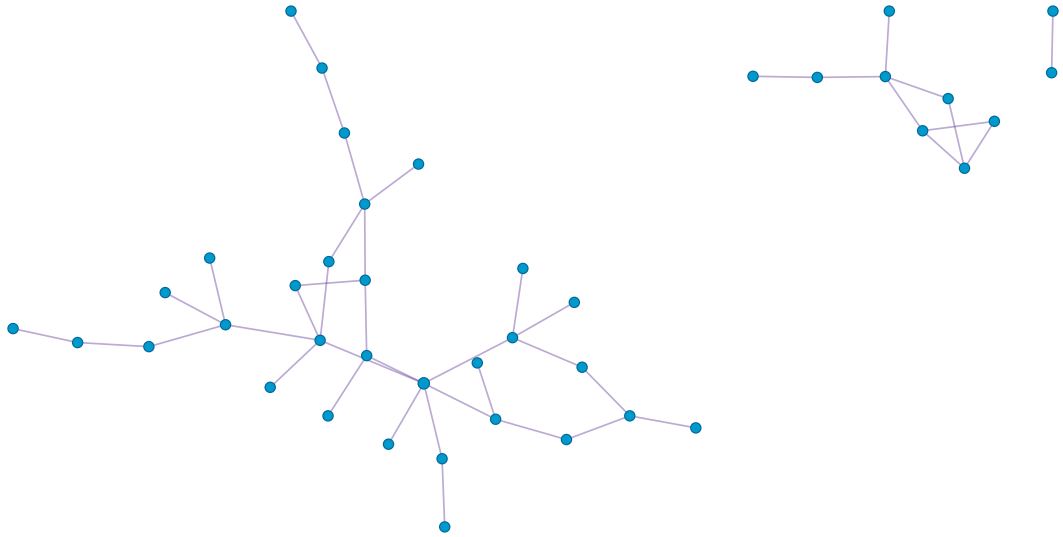

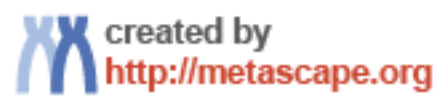

Supplement: Supplementary file 4 — Supplementary file4 (ZIP 16237 kb) [file 335_2024_10050_MOESM4_ESM.zip › Enrichment_PPI/Greenyellow_PPIColorByCluster.pdf]

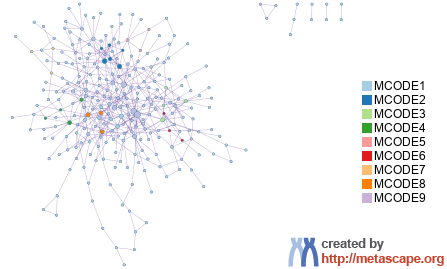

Supplement: Supplementary file 4 — Supplementary file4 (ZIP 16237 kb) [file 335_2024_10050_MOESM4_ESM.zip › Enrichment_PPI/Blue_PPIColorByCluster.png]

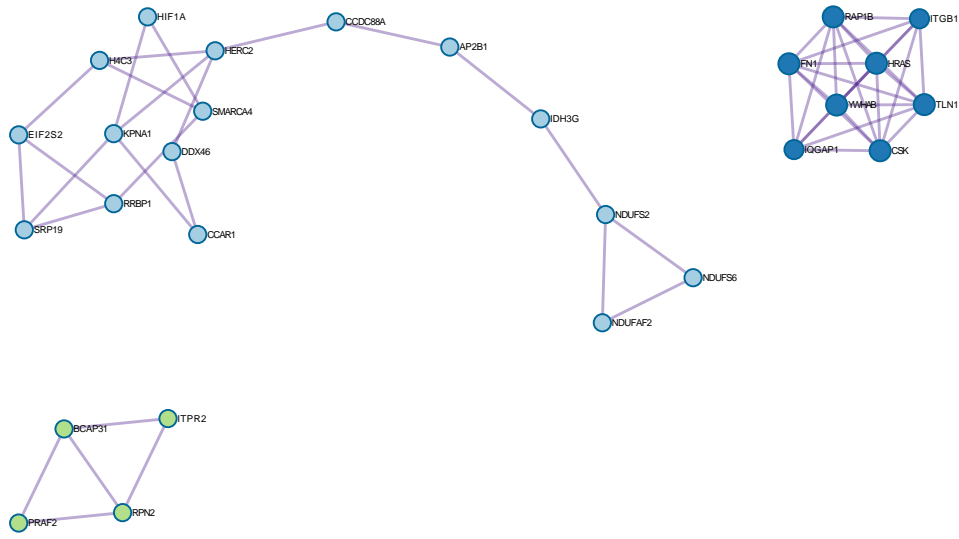

■ MCODE1  
■ MCODE2  
■ MCODE3

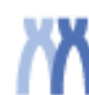 created by  
<http://metascape.org>

Supplement: Supplementary file 4 — Supplementary file4 (ZIP 16237 kb) [file 335_2024_10050_MOESM4_ESM.zip › Enrichment_PPI/Magenta_MCODE_ALL_PPIColorByCluster.pdf]

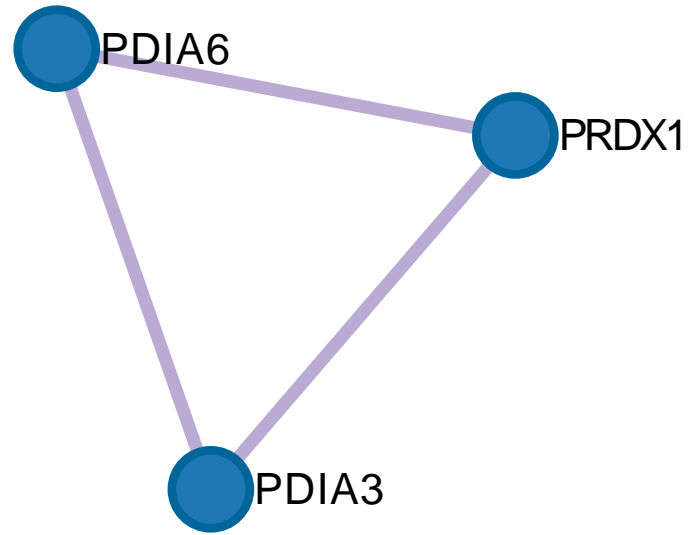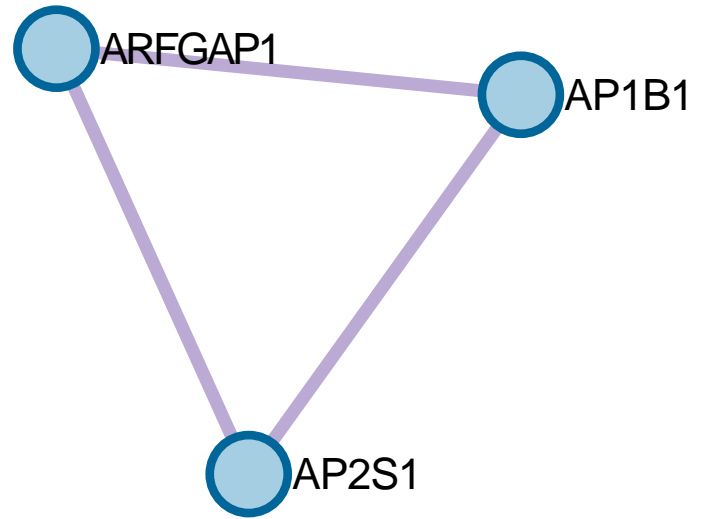

■ MCODE1  
■ MCODE2

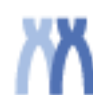 created by  
<http://metascape.org>

Supplement: Supplementary file 4 — Supplementary file4 (ZIP 16237 kb) [file 335_2024_10050_MOESM4_ESM.zip › Enrichment_PPI/Tan_MCODE_ALL_PPIColorByCluster.pdf]

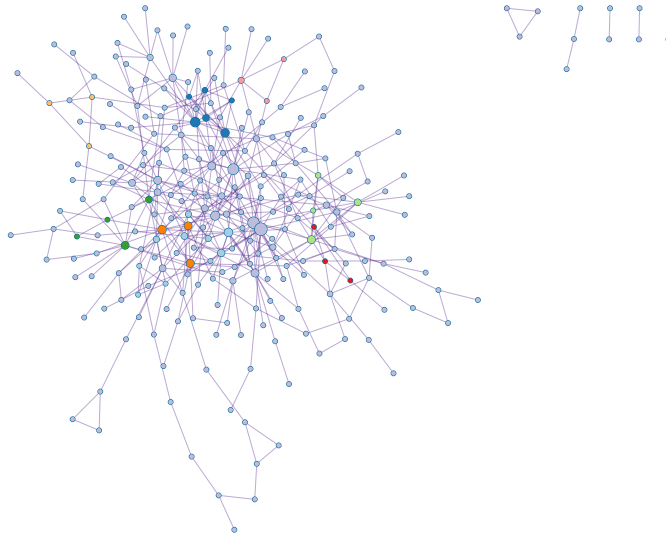

■ MCODE1  
■ MCODE2  
■ MCODE3  
■ MCODE4  
■ MCODE5  
■ MCODE6  
■ MCODE7  
■ MCODE8  
■ MCODE9

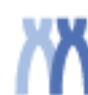 created by  
<http://metascape.org>

Supplement: Supplementary file 4 — Supplementary file4 (ZIP 16237 kb) [file 335_2024_10050_MOESM4_ESM.zip › Enrichment_PPI/Blue_PPIColorByCluster.pdf]

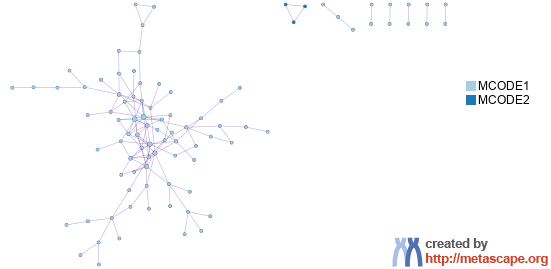

Supplement: Supplementary file 4 — Supplementary file4 (ZIP 16237 kb) [file 335_2024_10050_MOESM4_ESM.zip › Enrichment_PPI/Pink_PPIColorByCluster.png]

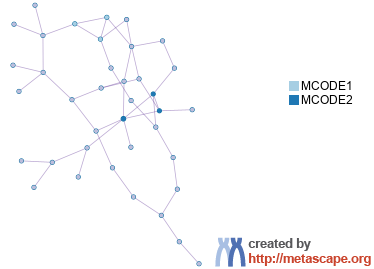

Supplement: Supplementary file 4 — Supplementary file4 (ZIP 16237 kb) [file 335_2024_10050_MOESM4_ESM.zip › Enrichment_PPI/Tan_PPIColorByCluster.png]

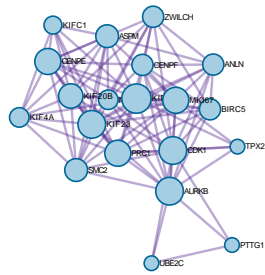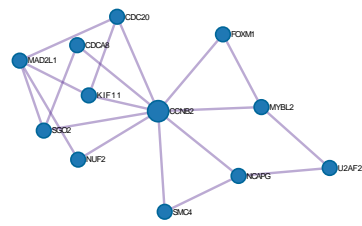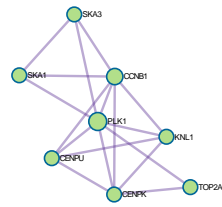

■ MCODE1  
■ MCODE2  
■ MCODE3

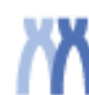 created by  
<http://metascape.org>

Supplement: Supplementary file 4 — Supplementary file4 (ZIP 16237 kb) [file 335_2024_10050_MOESM4_ESM.zip › Enrichment_PPI/Purple_MCODE_ALL_PPIColorByCluster.pdf]

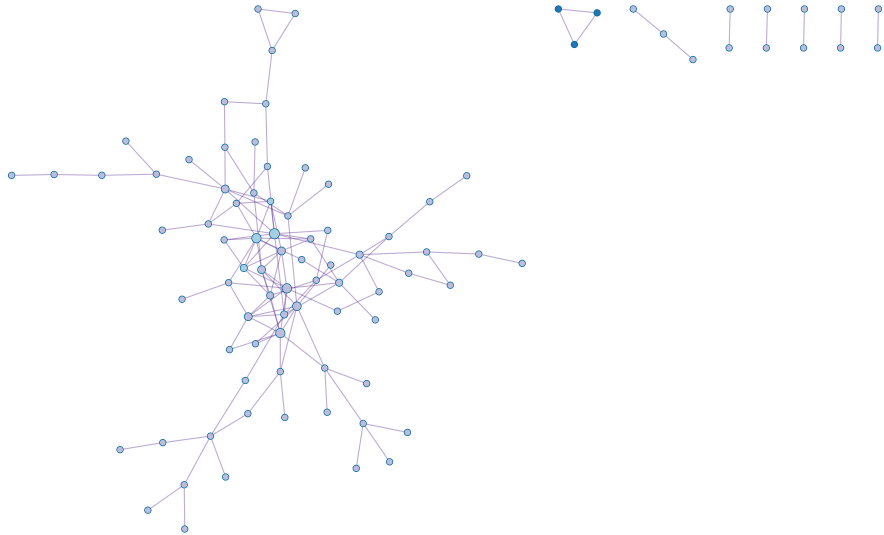

■ MCODE1  
■ MCODE2

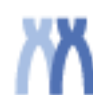 created by  
<http://metascape.org>

Supplement: Supplementary file 4 — Supplementary file4 (ZIP 16237 kb) [file 335_2024_10050_MOESM4_ESM.zip › Enrichment_PPI/Pink_PPIColorByCluster.pdf]

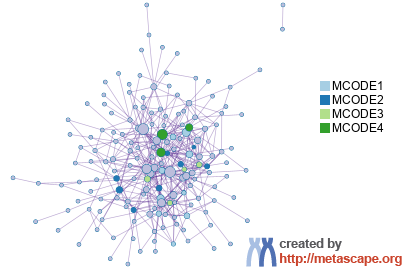

Supplement: Supplementary file 4 — Supplementary file4 (ZIP 16237 kb) [file 335_2024_10050_MOESM4_ESM.zip › Enrichment_PPI/Black_PPIColorByCluster.png]

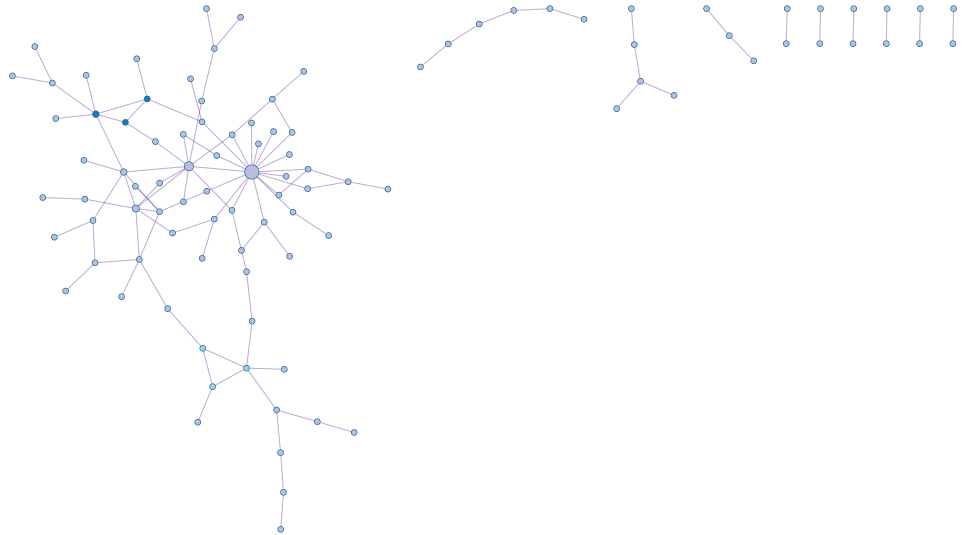

■ MCODE1  
■ MCODE2

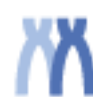 created by  
<http://metascape.org>

Supplement: Supplementary file 4 — Supplementary file4 (ZIP 16237 kb) [file 335_2024_10050_MOESM4_ESM.zip › Enrichment_PPI/Red_PPIColorByCluster.pdf]

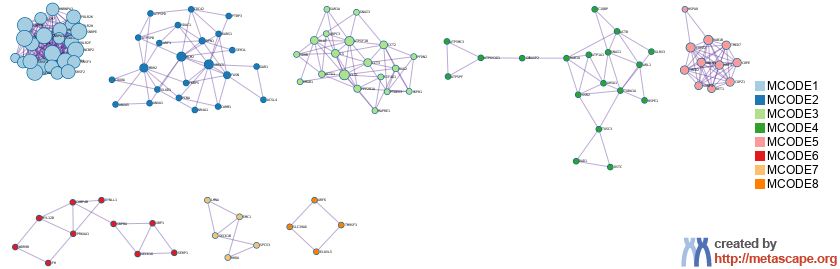

Supplement: Supplementary file 4 — Supplementary file4 (ZIP 16237 kb) [file 335_2024_10050_MOESM4_ESM.zip › Enrichment_PPI/Brown_MCODE_ALL_PPIColorByCluster.png]

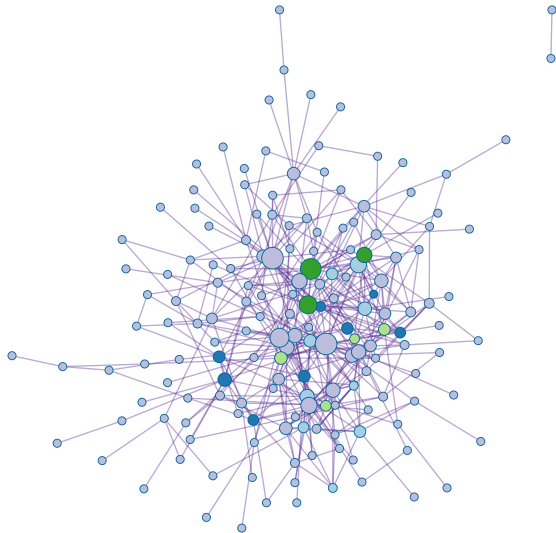

■ MCODE1  
■ MCODE2  
■ MCODE3  
■ MCODE4

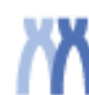 created by  
<http://metascape.org>

Supplement: Supplementary file 4 — Supplementary file4 (ZIP 16237 kb) [file 335_2024_10050_MOESM4_ESM.zip › Enrichment_PPI/Black_PPIColorByCluster.pdf]

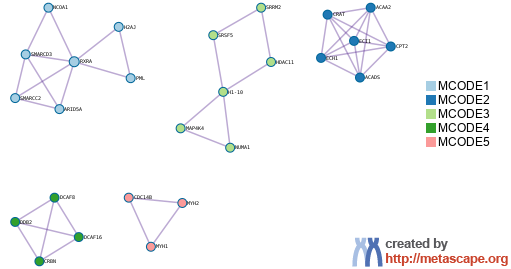

Supplement: Supplementary file 4 — Supplementary file4 (ZIP 16237 kb) [file 335_2024_10050_MOESM4_ESM.zip › Enrichment_PPI/Yellow_MCODE_ALL_PPIColorByCluster.png]

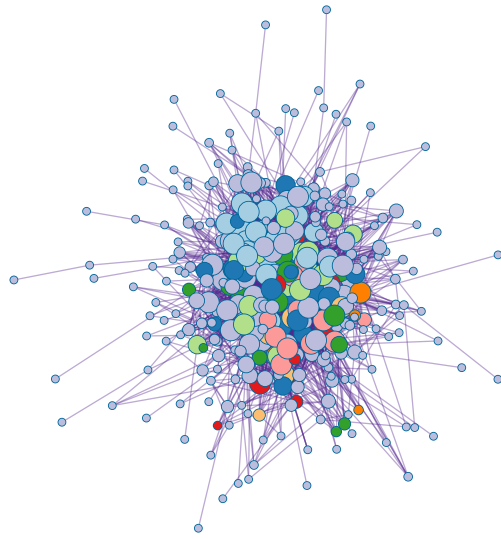

■ MCODE1  
■ MCODE2  
■ MCODE3  
■ MCODE4  
■ MCODE5  
■ MCODE6  
■ MCODE7  
■ MCODE8

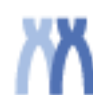 created by  
<http://metascape.org>

Supplement: Supplementary file 4 — Supplementary file4 (ZIP 16237 kb) [file 335_2024_10050_MOESM4_ESM.zip › Enrichment_PPI/Brown_PPIColorByCluster.pdf]

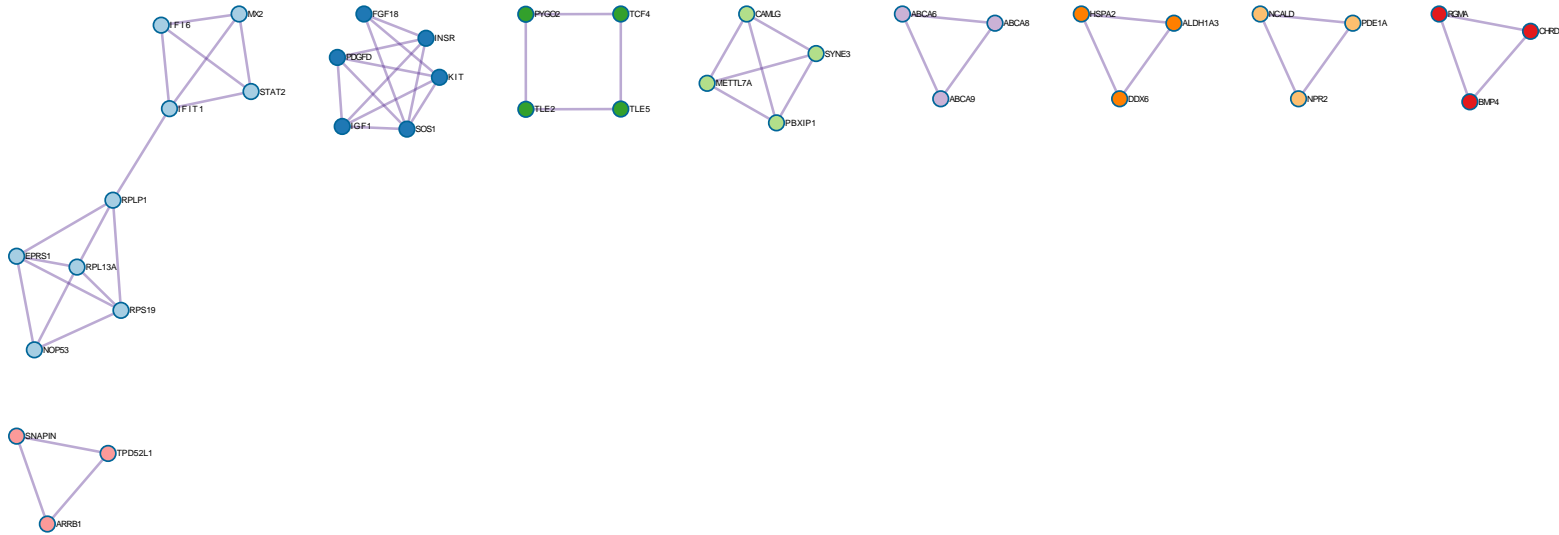

■ MCODE1  
■ MCODE2  
■ MCODE3  
■ MCODE4  
■ MCODE5  
■ MCODE6  
■ MCODE7  
■ MCODE8  
■ MCODE9

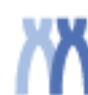 created by  
<http://metascape.org>

Supplement: Supplementary file 4 — Supplementary file4 (ZIP 16237 kb) [file 335_2024_10050_MOESM4_ESM.zip › Enrichment_PPI/Blue_MCODE_ALL_PPIColorByCluster.pdf]

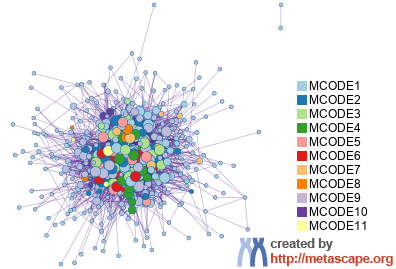

Supplement: Supplementary file 4 — Supplementary file4 (ZIP 16237 kb) [file 335_2024_10050_MOESM4_ESM.zip › Enrichment_PPI/Turquoise_PPIColorByCluster.png]

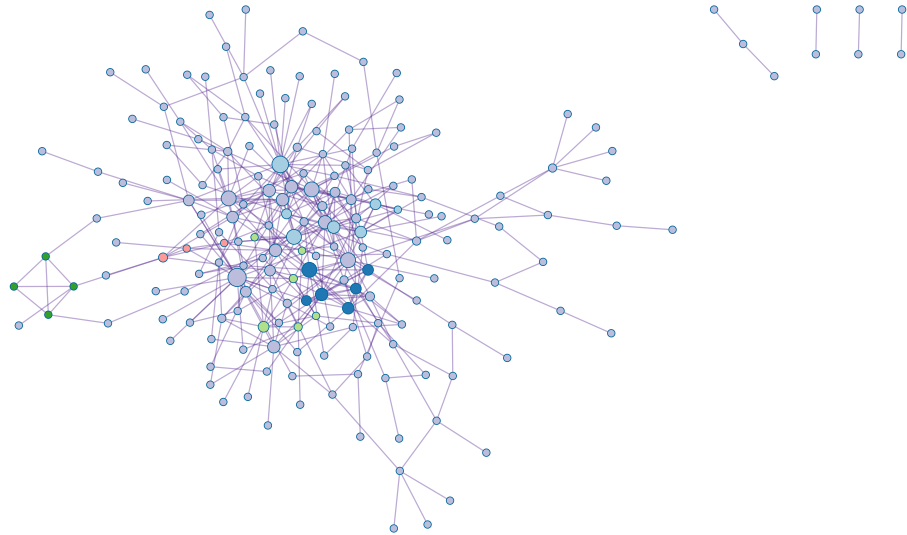

■ MCODE1  
■ MCODE2  
■ MCODE3  
■ MCODE4  
■ MCODE5

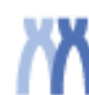 created by  
<http://metascape.org>

Supplement: Supplementary file 4 — Supplementary file4 (ZIP 16237 kb) [file 335_2024_10050_MOESM4_ESM.zip › Enrichment_PPI/Yellow_PPIColorByCluster.pdf]

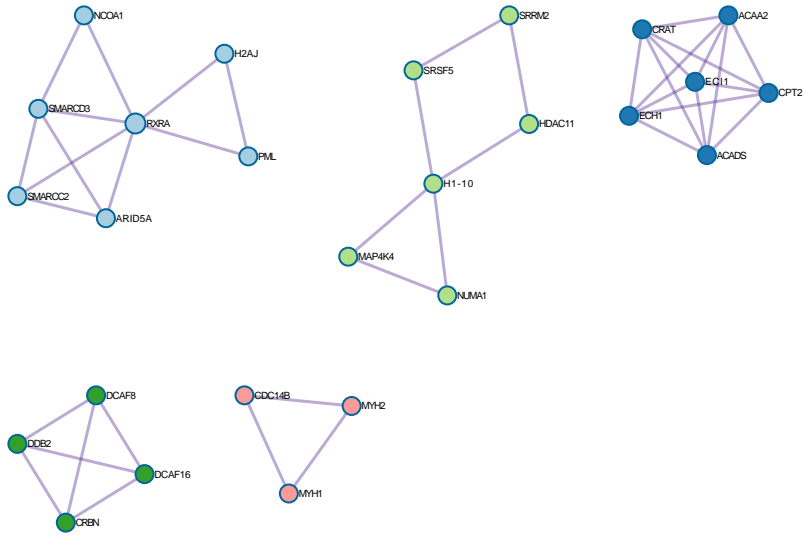

■ MCODE1  
■ MCODE2  
■ MCODE3  
■ MCODE4  
■ MCODE5

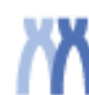 created by  
<http://metascape.org>

Supplement: Supplementary file 4 — Supplementary file4 (ZIP 16237 kb) [file 335_2024_10050_MOESM4_ESM.zip › Enrichment_PPI/Yellow_MCODE_ALL_PPIColorByCluster.pdf]

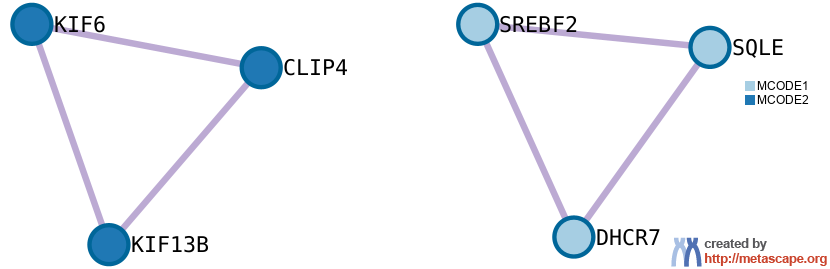

Supplement: Supplementary file 4 — Supplementary file4 (ZIP 16237 kb) [file 335_2024_10050_MOESM4_ESM.zip › Enrichment_PPI/Red_MCODE_ALL_PPIColorByCluster.png]

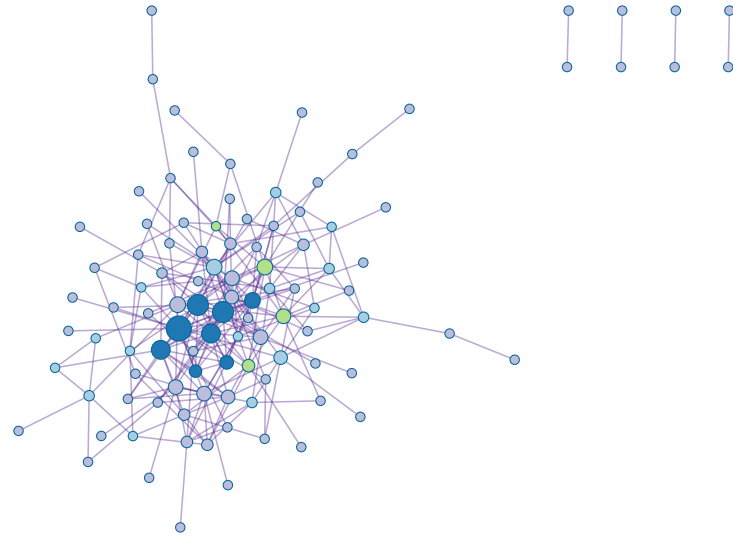

■ MCODE1  
■ MCODE2  
■ MCODE3

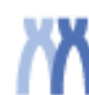 created by  
<http://metascape.org>

Supplement: Supplementary file 4 — Supplementary file4 (ZIP 16237 kb) [file 335_2024_10050_MOESM4_ESM.zip › Enrichment_PPI/Magenta_PPIColorByCluster.pdf]

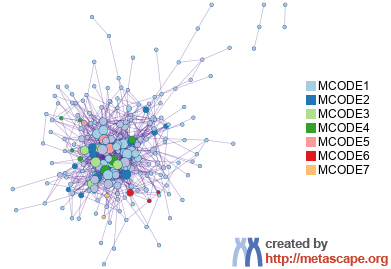

Supplement: Supplementary file 4 — Supplementary file4 (ZIP 16237 kb) [file 335_2024_10050_MOESM4_ESM.zip › Enrichment_PPI/Green_PPIColorByCluster.png]

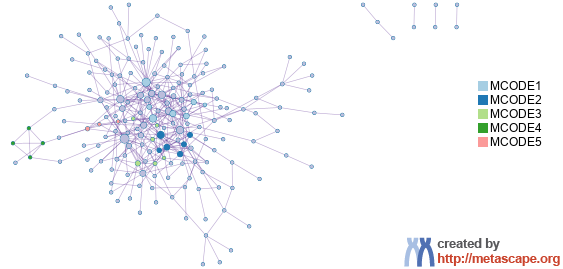

Supplement: Supplementary file 4 — Supplementary file4 (ZIP 16237 kb) [file 335_2024_10050_MOESM4_ESM.zip › Enrichment_PPI/Yellow_PPIColorByCluster.png]

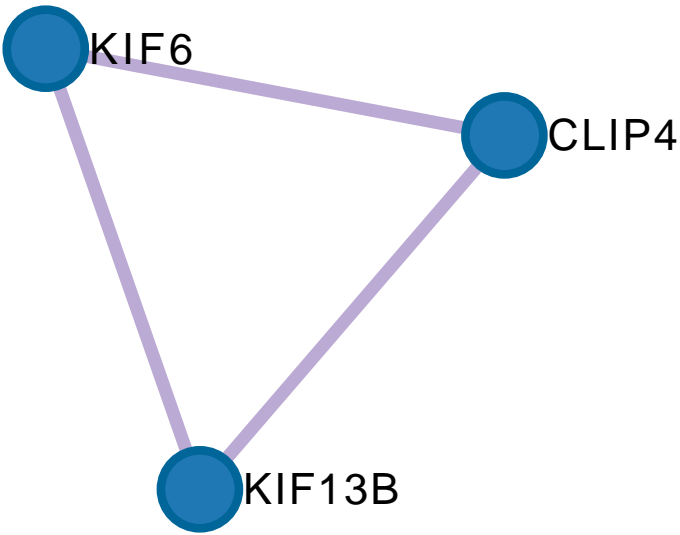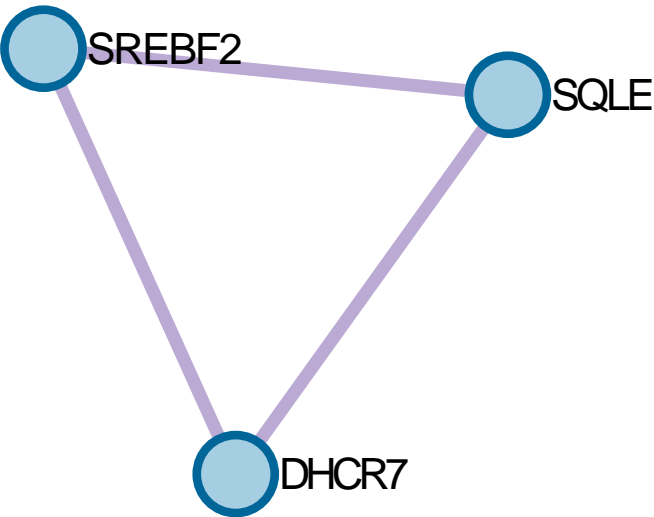

■ MCODE1  
■ MCODE2

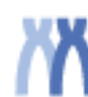 created by  
<http://metascape.org>

Supplement: Supplementary file 4 — Supplementary file4 (ZIP 16237 kb) [file 335_2024_10050_MOESM4_ESM.zip › Enrichment_PPI/Red_MCODE_ALL_PPIColorByCluster.pdf]

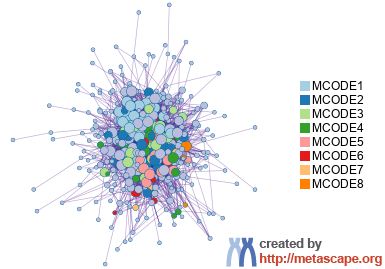

Supplement: Supplementary file 4 — Supplementary file4 (ZIP 16237 kb) [file 335_2024_10050_MOESM4_ESM.zip › Enrichment_PPI/Brown_PPIColorByCluster.png]

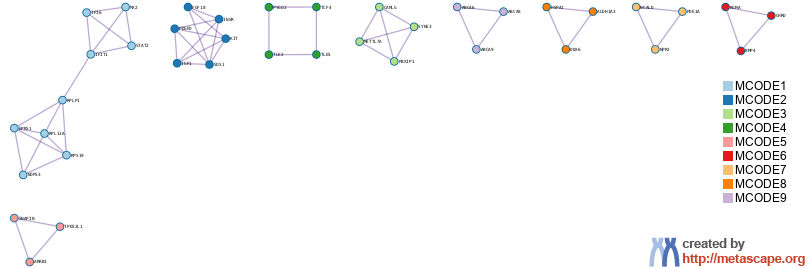

Supplement: Supplementary file 4 — Supplementary file4 (ZIP 16237 kb) [file 335_2024_10050_MOESM4_ESM.zip › Enrichment_PPI/Blue_MCODE_ALL_PPIColorByCluster.png]

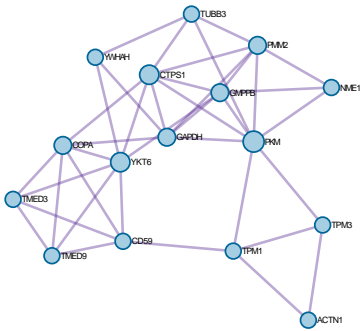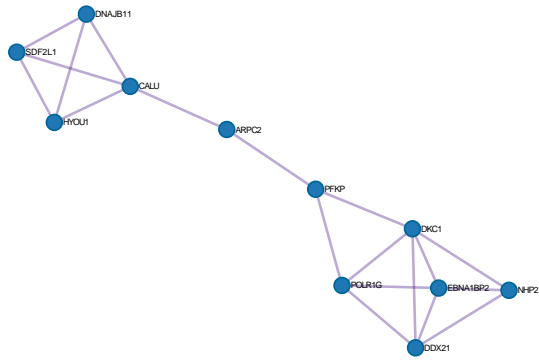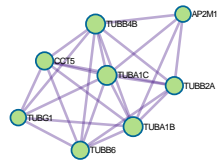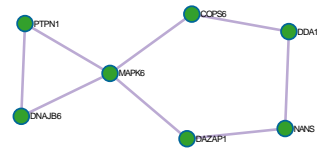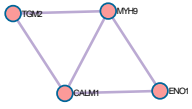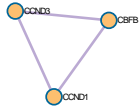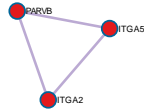

■ MCODE1  
■ MCODE2  
■ MCODE3  
■ MCODE4  
■ MCODE5  
■ MCODE6  
■ MCODE7

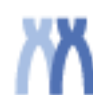 created by  
<http://metascape.org>

Supplement: Supplementary file 4 — Supplementary file4 (ZIP 16237 kb) [file 335_2024_10050_MOESM4_ESM.zip › Enrichment_PPI/Green_MCODE_ALL_PPIColorByCluster.pdf]

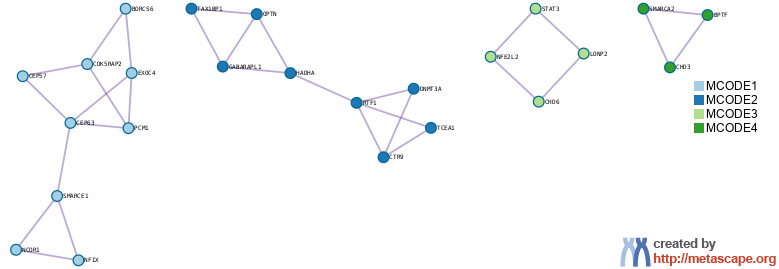

Supplement: Supplementary file 4 — Supplementary file4 (ZIP 16237 kb) [file 335_2024_10050_MOESM4_ESM.zip › Enrichment_PPI/Black_MCODE_ALL_PPIColorByCluster.png]

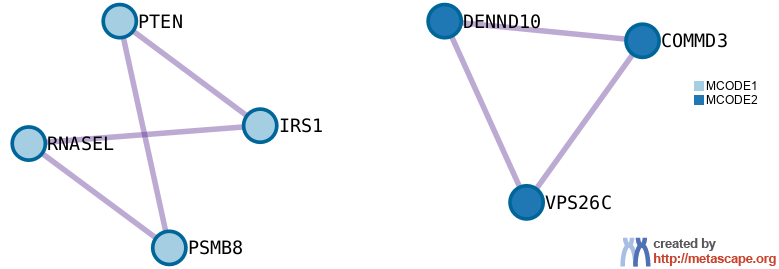

Supplement: Supplementary file 4 — Supplementary file4 (ZIP 16237 kb) [file 335_2024_10050_MOESM4_ESM.zip › Enrichment_PPI/Pink_MCODE_ALL_PPIColorByCluster.png]

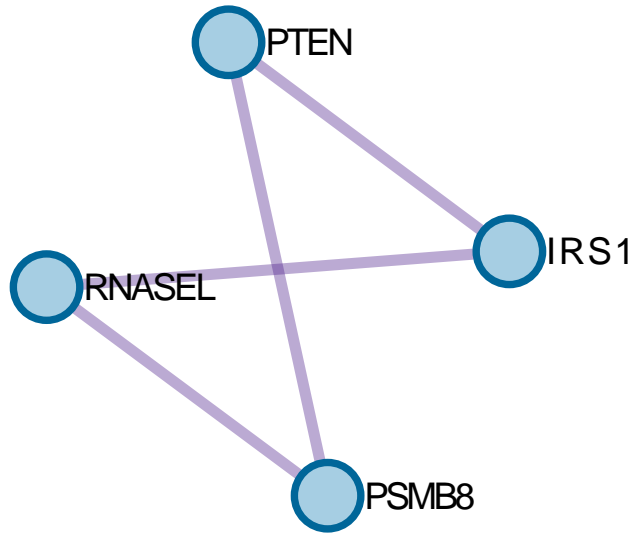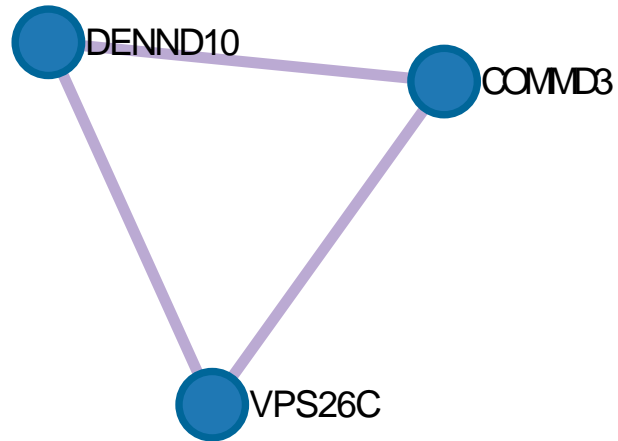

■ MCODE1  
■ MCODE2

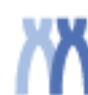 created by  
<http://metascape.org>

Supplement: Supplementary file 4 — Supplementary file4 (ZIP 16237 kb) [file 335_2024_10050_MOESM4_ESM.zip › Enrichment_PPI/Pink_MCODE_ALL_PPIColorByCluster.pdf]

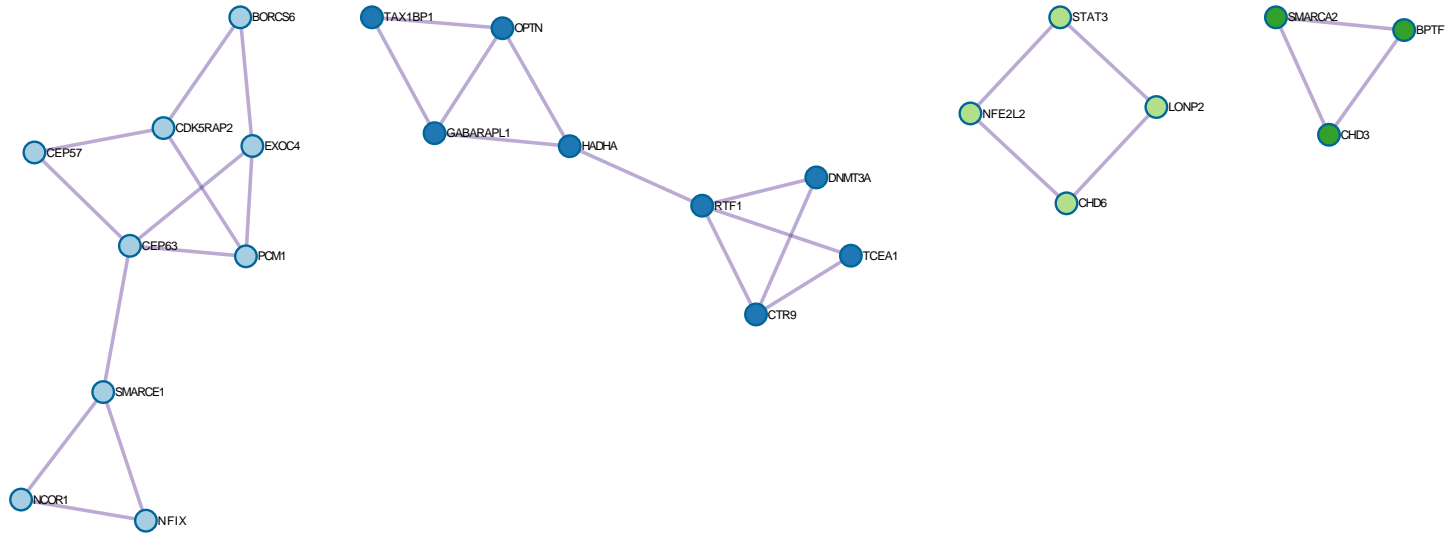

■ MCODE1  
■ MCODE2  
■ MCODE3  
■ MCODE4

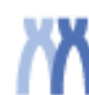 created by  
<http://metascape.org>

Supplement: Supplementary file 4 — Supplementary file4 (ZIP 16237 kb) [file 335_2024_10050_MOESM4_ESM.zip › Enrichment_PPI/Black_MCODE_ALL_PPIColorByCluster.pdf]
