# Supplementary material for: Fibroblasts as an in vitro model of circadian genetic and genomic studies
Source: Mamm Genome. 2024 Jul 3;35(3):432–44. doi: 10.1007/s00335-024-10050-7 (PMC11329553; doi:10.1007/s00335-024-10050-7)
Supplement: Supplementary file 4 — Supplementary file4 (ZIP 16237 kb) [file 335_2024_10050_MOESM4_ESM.zip › AnalysisReport.pptx]

## Slide 1
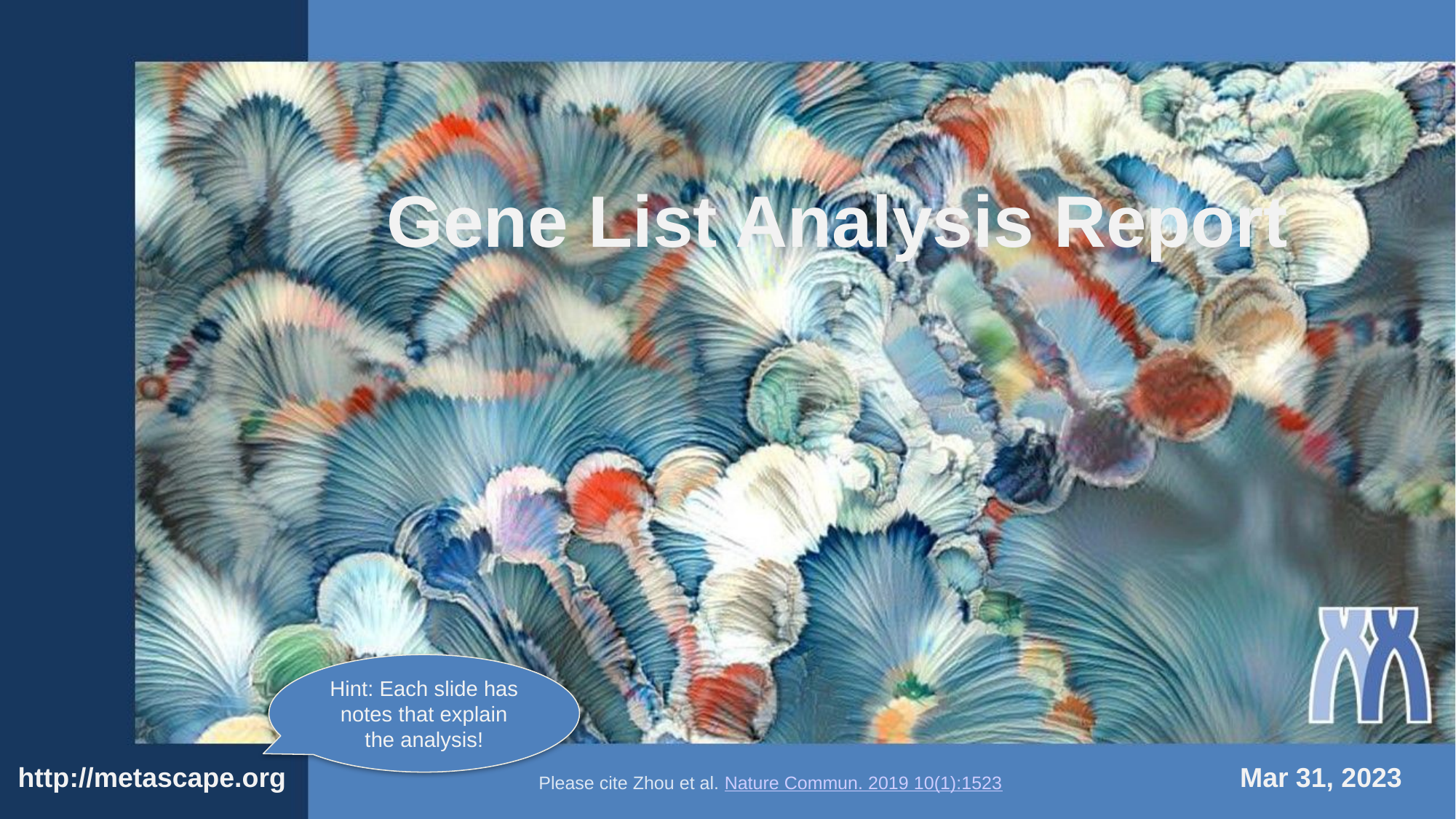

Gene List Analysis Report
Hint: Each slide has notes that explain the analysis!
http://metascape.org
Mar 31, 2023
Please cite Zhou et al. Nature Commun. 2019 10(1):1523

## Slide 2
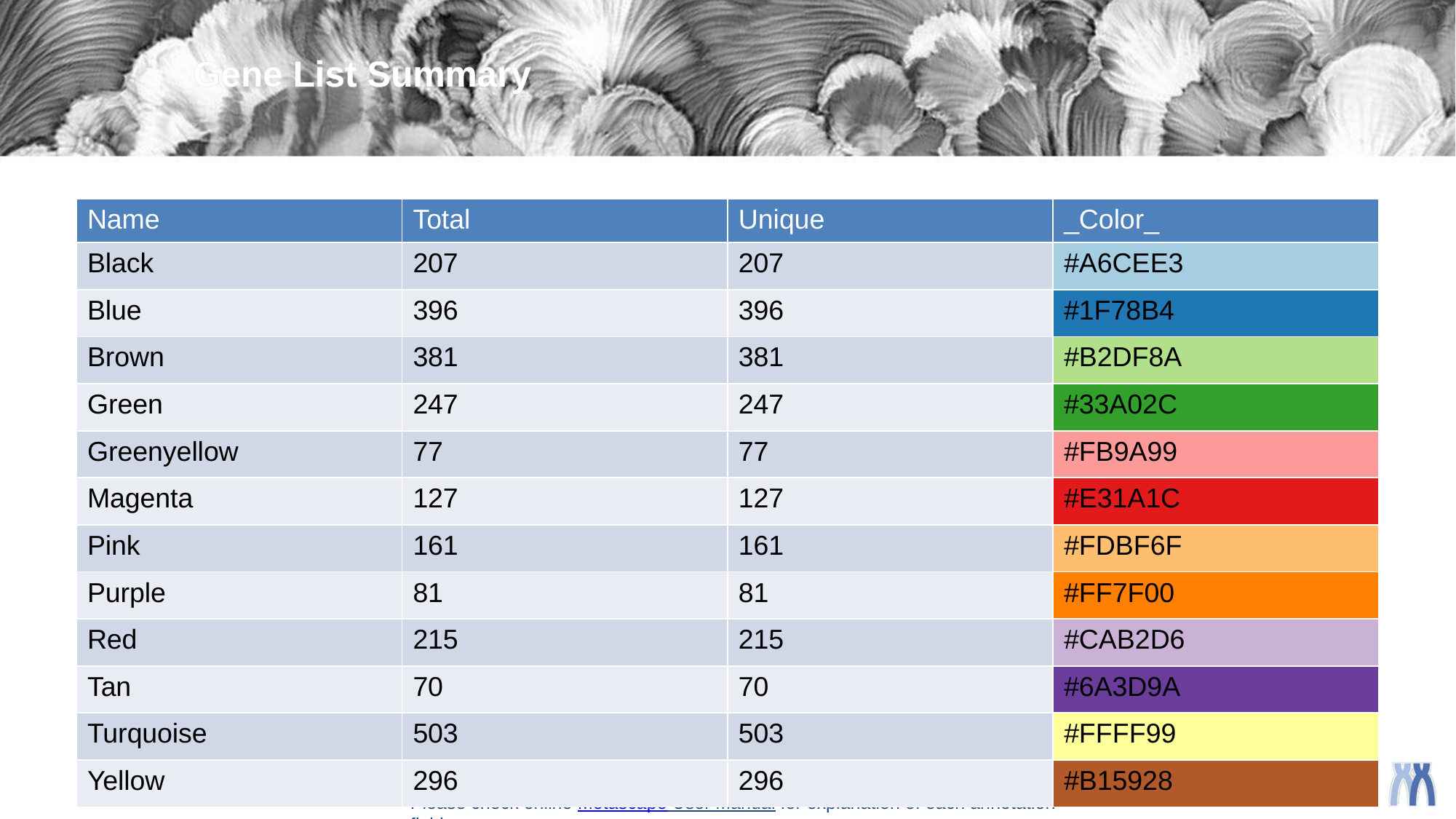

Gene List Summary
| Name | Total | Unique | \_Color\_ |
| --- | --- | --- | --- |
| Black | 207 | 207 | #A6CEE3 |
| Blue | 396 | 396 | #1F78B4 |
| Brown | 381 | 381 | #B2DF8A |
| Green | 247 | 247 | #33A02C |
| Greenyellow | 77 | 77 | #FB9A99 |
| Magenta | 127 | 127 | #E31A1C |
| Pink | 161 | 161 | #FDBF6F |
| Purple | 81 | 81 | #FF7F00 |
| Red | 215 | 215 | #CAB2D6 |
| Tan | 70 | 70 | #6A3D9A |
| Turquoise | 503 | 503 | #FFFF99 |
| Yellow | 296 | 296 | #B15928 |
Please check online Metascape User Manual for explanation of each annotation field.

## Slide 3
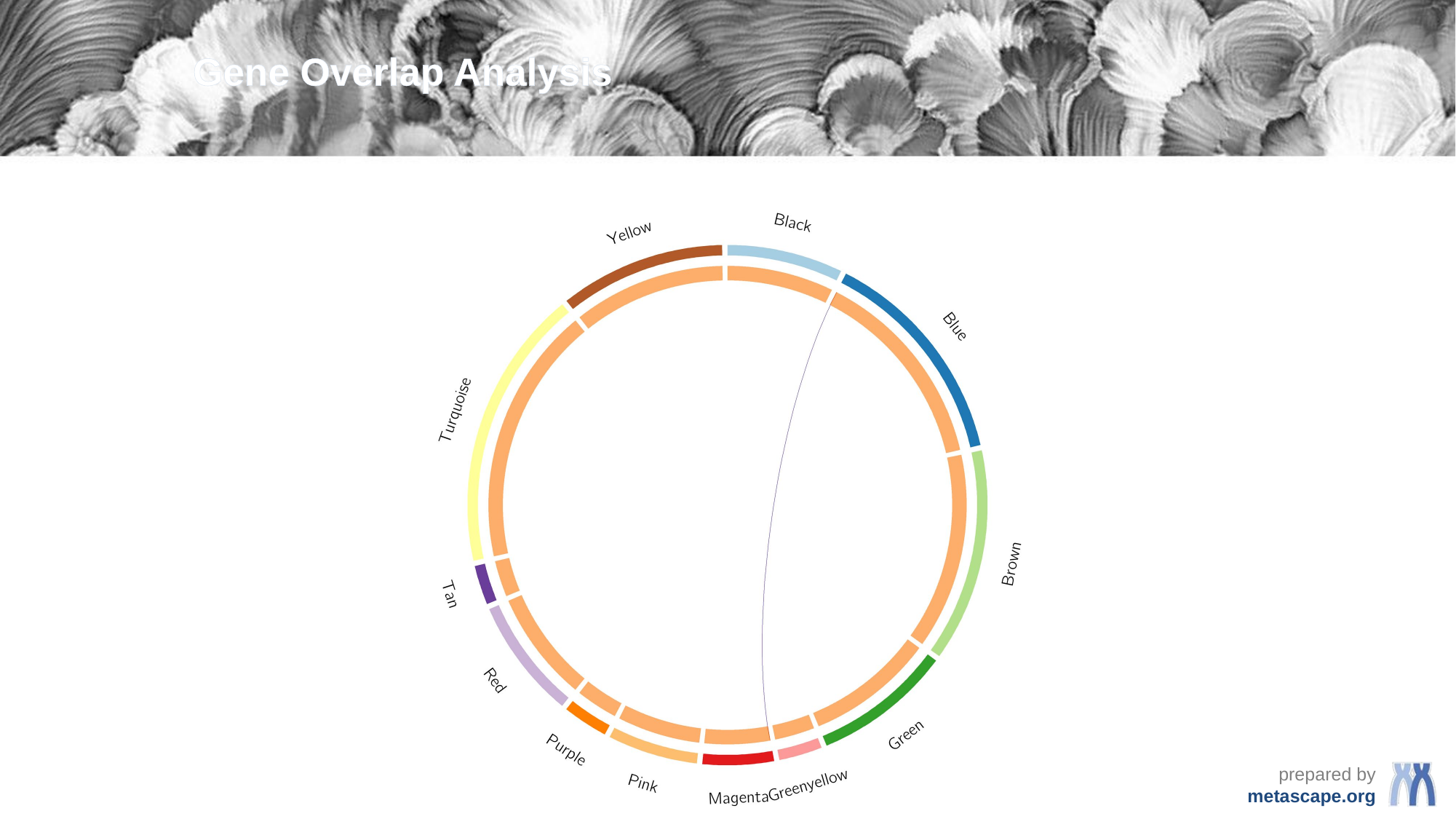

Gene Overlap Analysis

## Slide 4
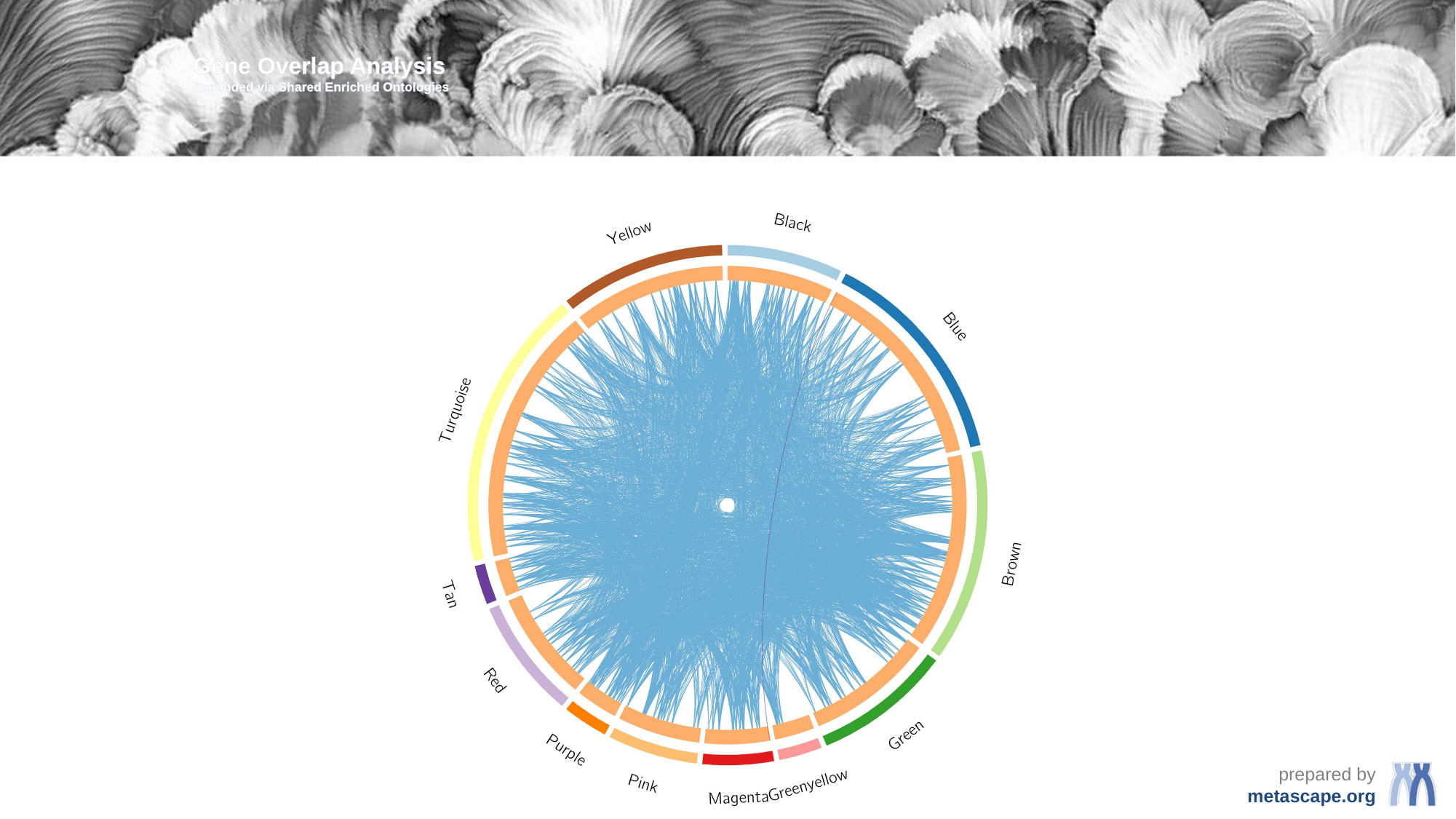

Gene Overlap Analysis
Expanded via Shared Enriched Ontologies

## Slide 5
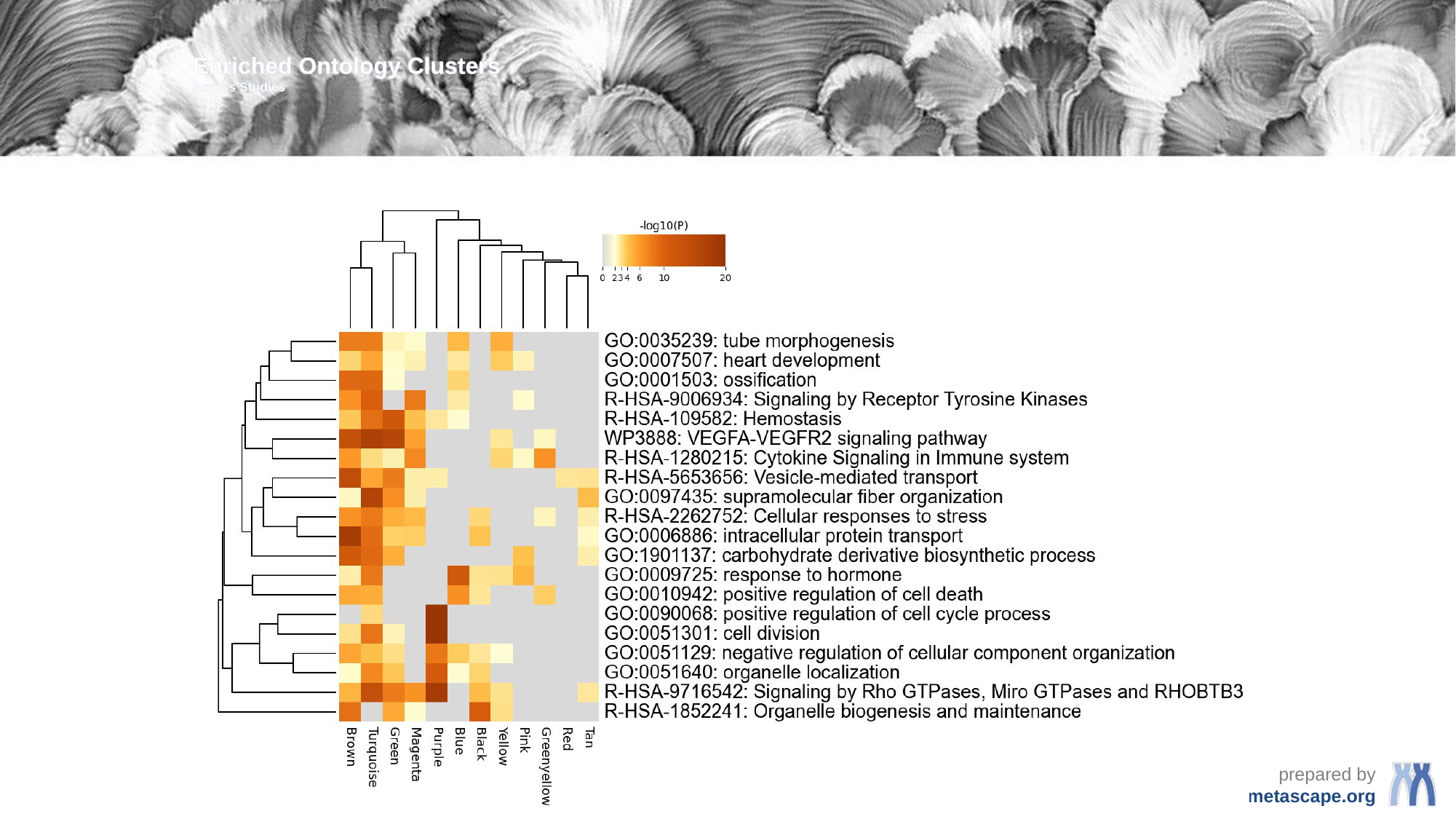

Enriched Ontology Clusters
Across Studies

## Slide 6
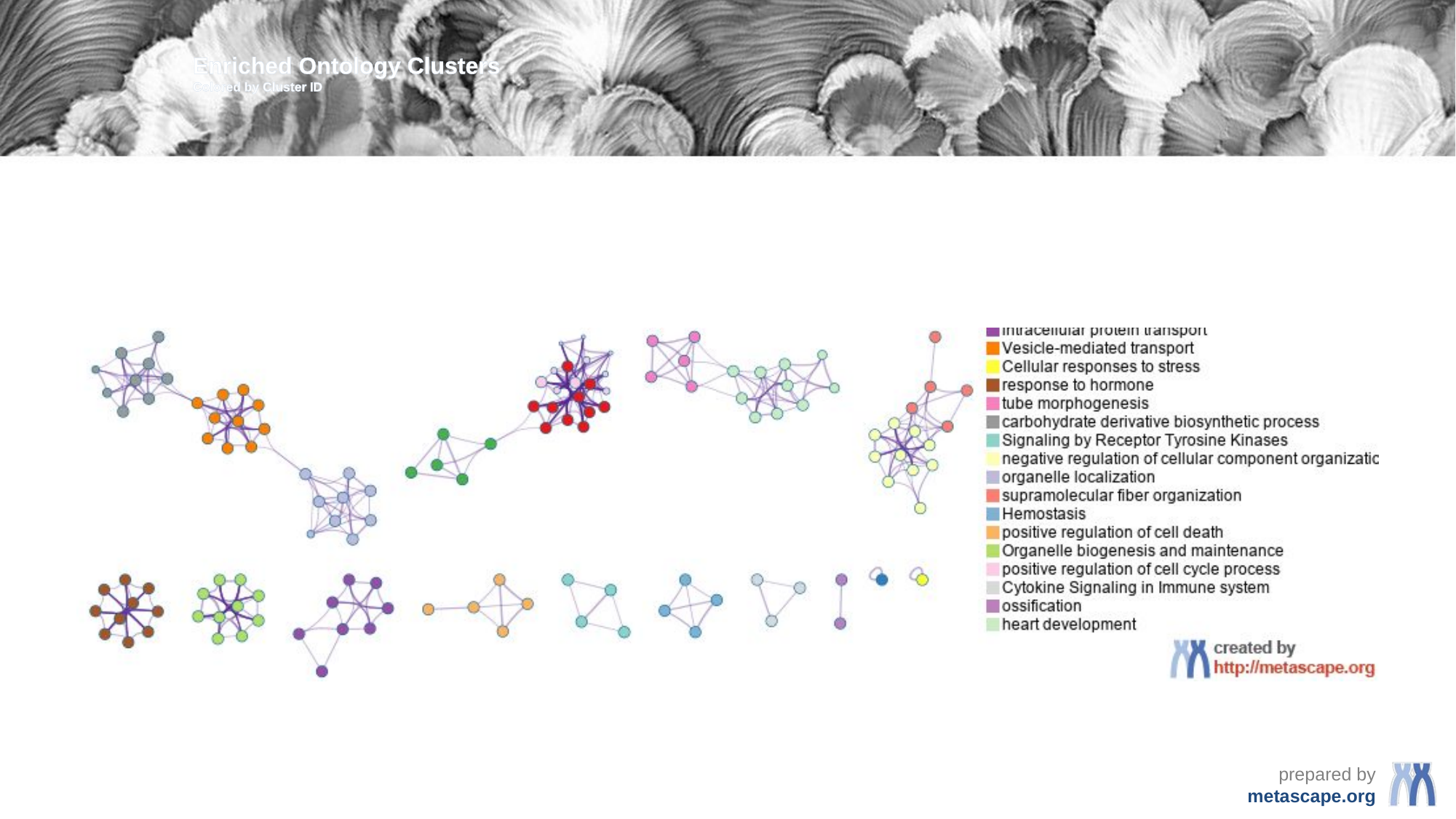

Enriched Ontology Clusters
Colored by Cluster ID

## Slide 7
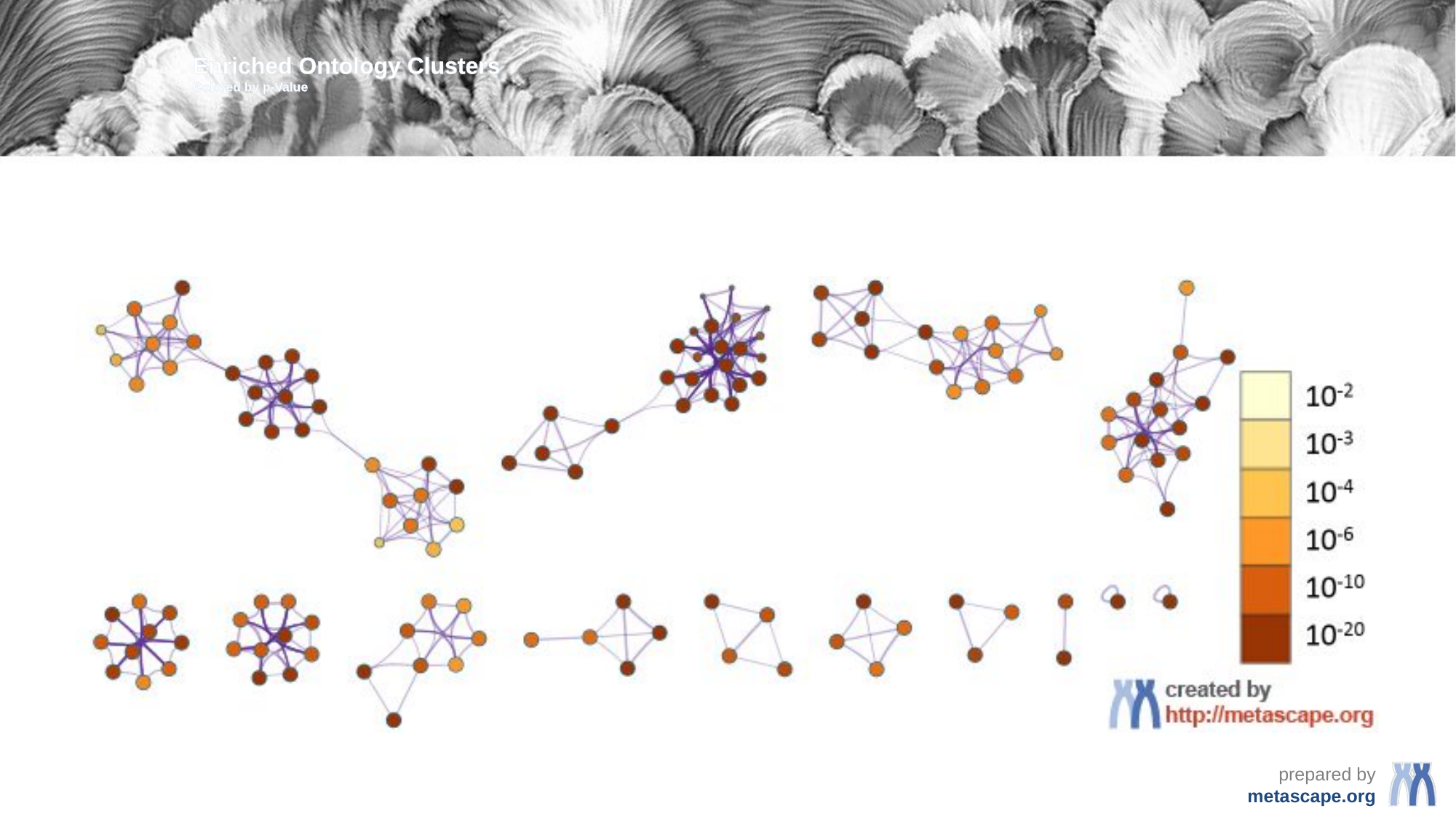

Enriched Ontology Clusters
Colored by p-Value

## Slide 8
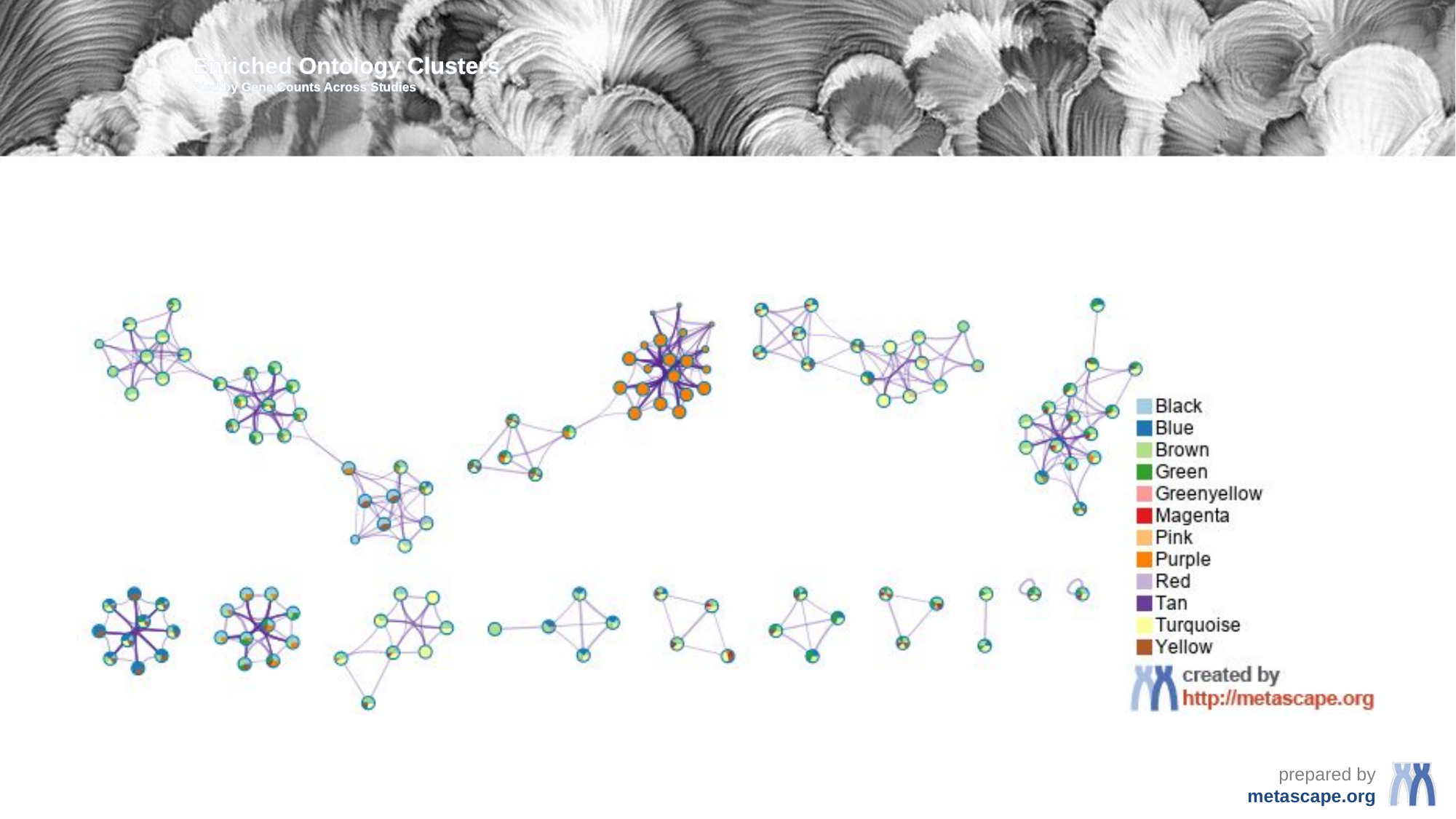

Enriched Ontology Clusters
Pied by Gene Counts Across Studies

## Slide 9
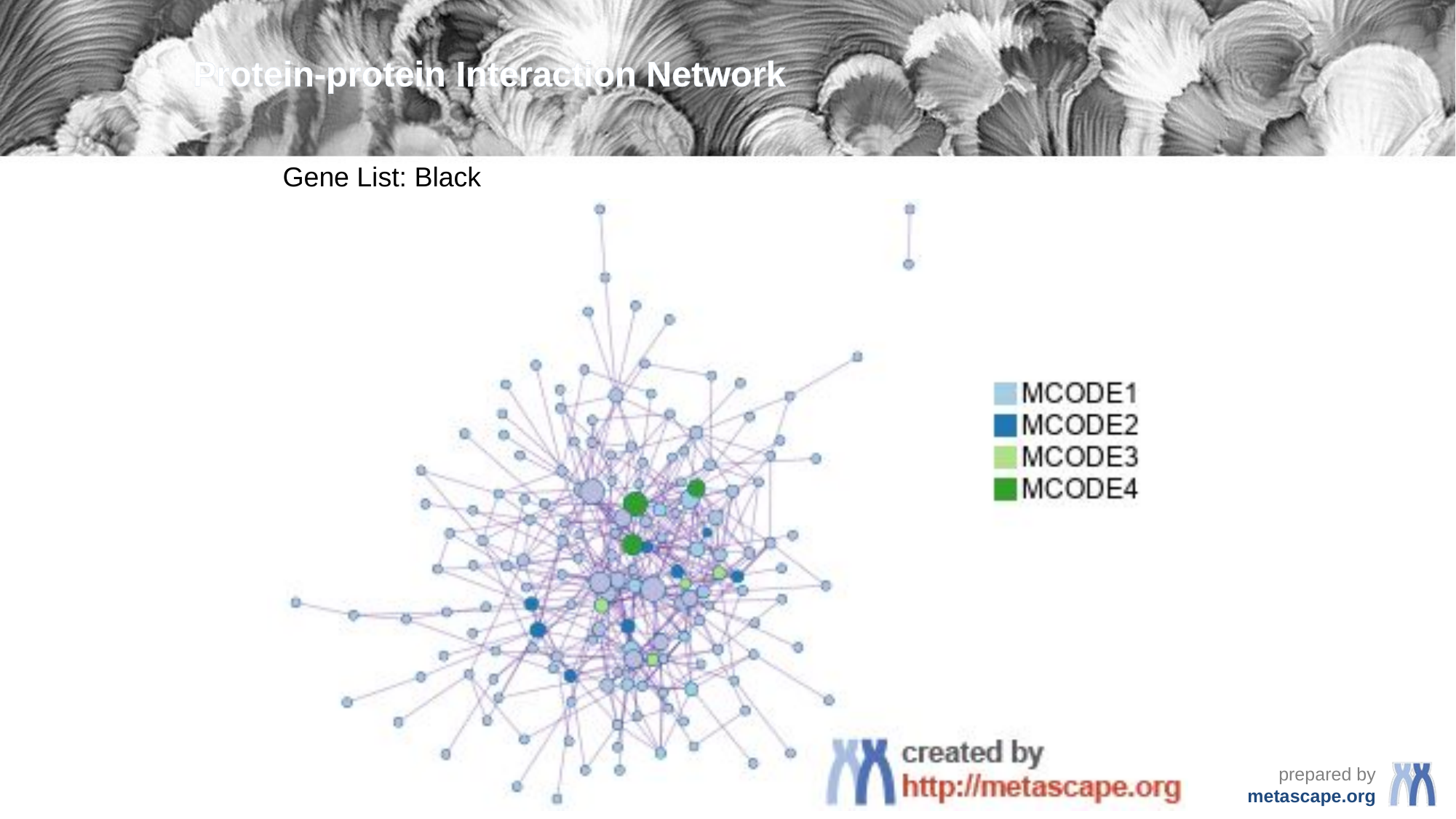

Protein-protein Interaction Network
Gene List: Black

## Slide 10
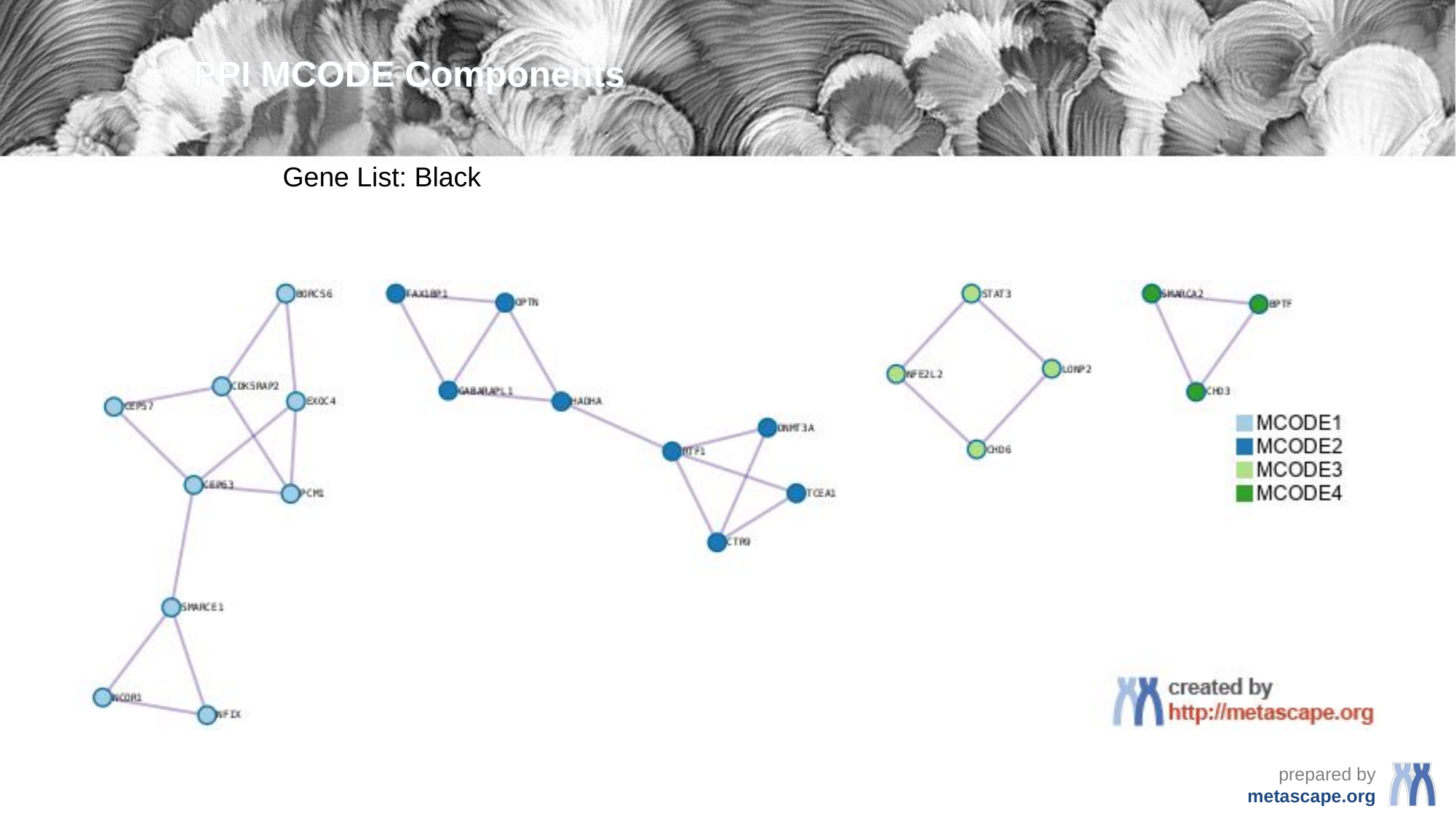

PPI MCODE Components
Gene List: Black

## Slide 11
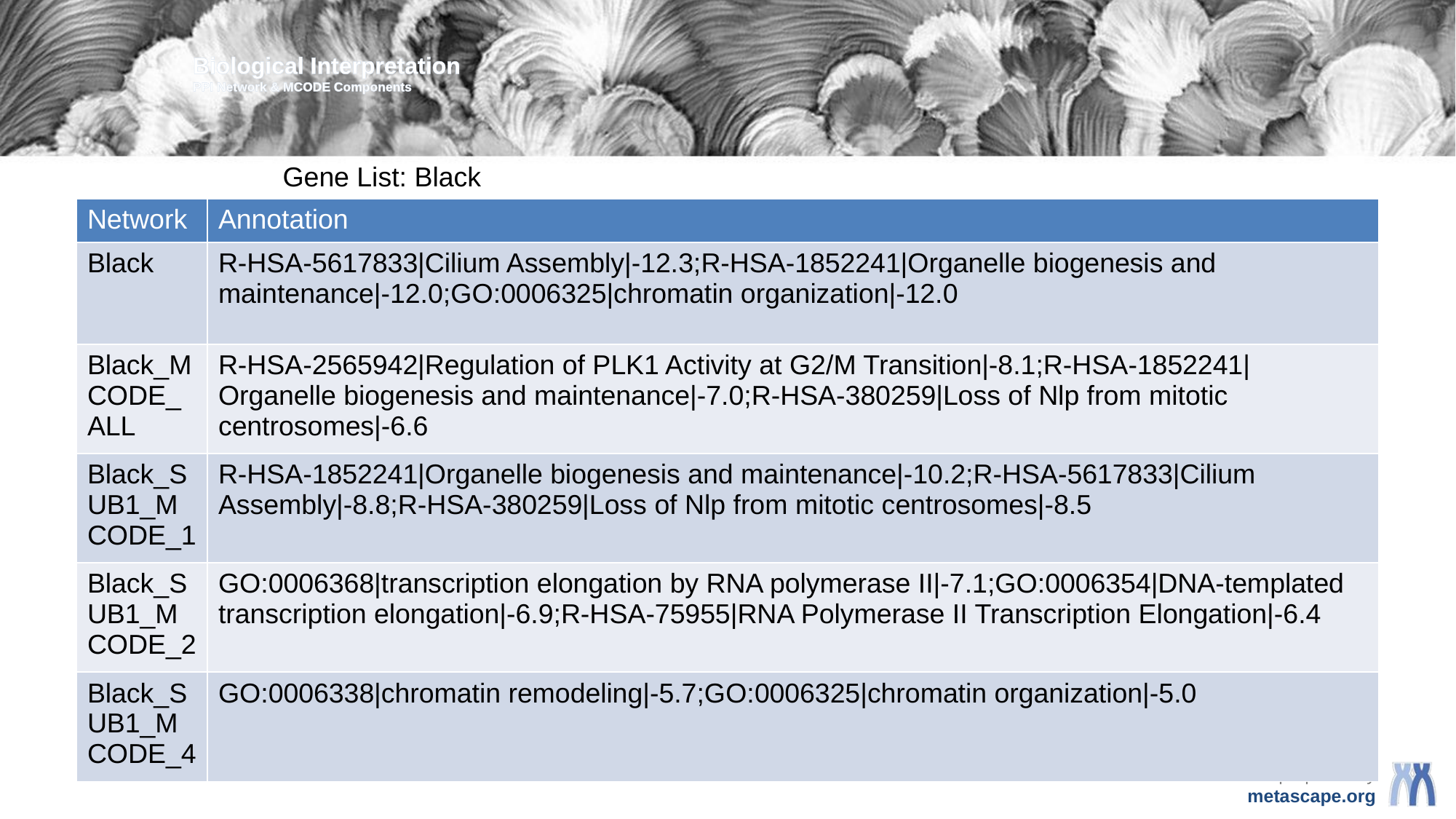

Biological Interpretation
PPI Network & MCODE Components
Gene List: Black
| Network | Annotation |
| --- | --- |
| Black | R-HSA-5617833|Cilium Assembly|-12.3;R-HSA-1852241|Organelle biogenesis and maintenance|-12.0;GO:0006325|chromatin organization|-12.0 |
| Black\_MCODE\_ALL | R-HSA-2565942|Regulation of PLK1 Activity at G2/M Transition|-8.1;R-HSA-1852241|Organelle biogenesis and maintenance|-7.0;R-HSA-380259|Loss of Nlp from mitotic centrosomes|-6.6 |
| Black\_SUB1\_MCODE\_1 | R-HSA-1852241|Organelle biogenesis and maintenance|-10.2;R-HSA-5617833|Cilium Assembly|-8.8;R-HSA-380259|Loss of Nlp from mitotic centrosomes|-8.5 |
| Black\_SUB1\_MCODE\_2 | GO:0006368|transcription elongation by RNA polymerase II|-7.1;GO:0006354|DNA-templated transcription elongation|-6.9;R-HSA-75955|RNA Polymerase II Transcription Elongation|-6.4 |
| Black\_SUB1\_MCODE\_4 | GO:0006338|chromatin remodeling|-5.7;GO:0006325|chromatin organization|-5.0 |

## Slide 12
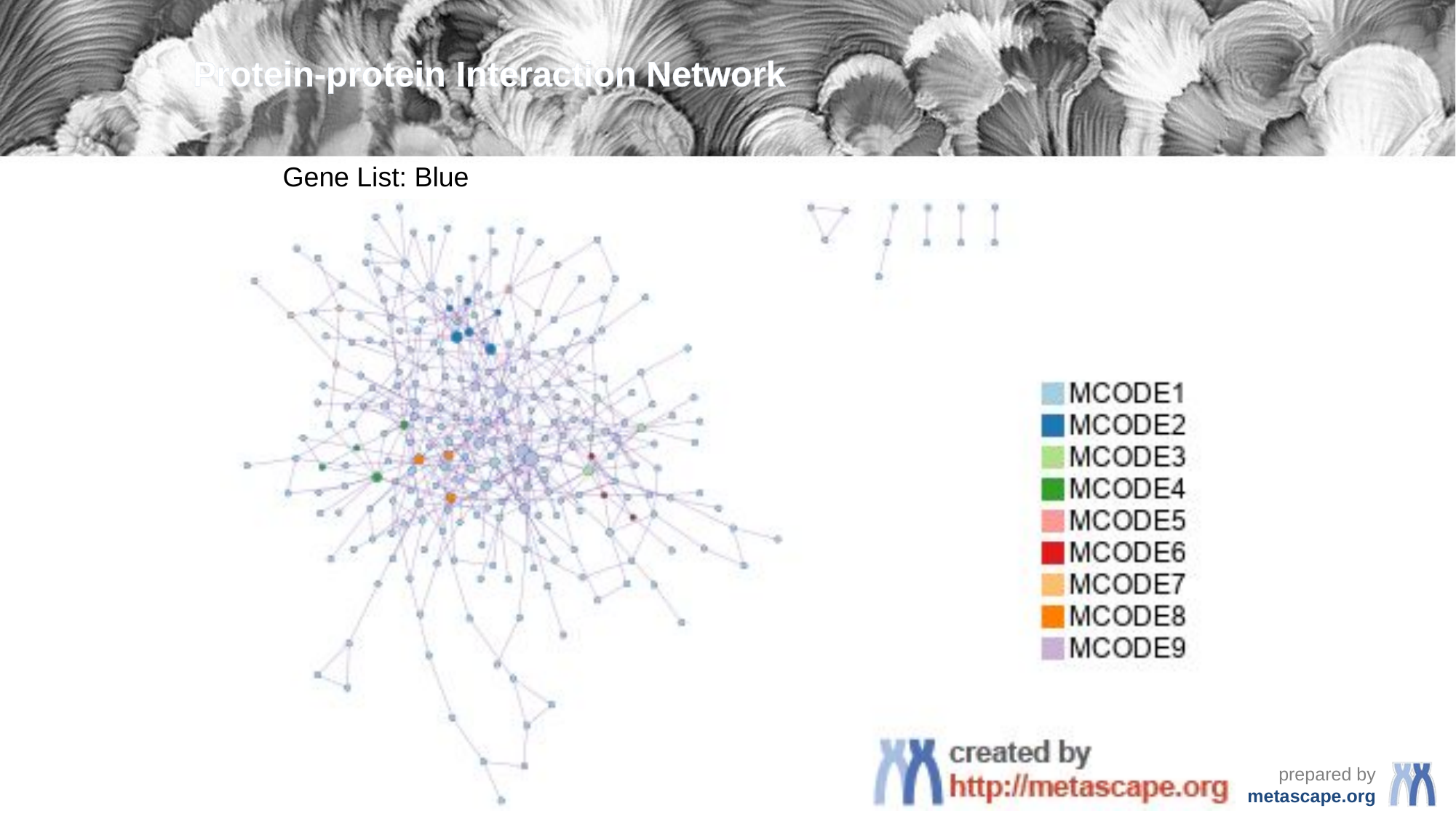

Protein-protein Interaction Network
Gene List: Blue

## Slide 13
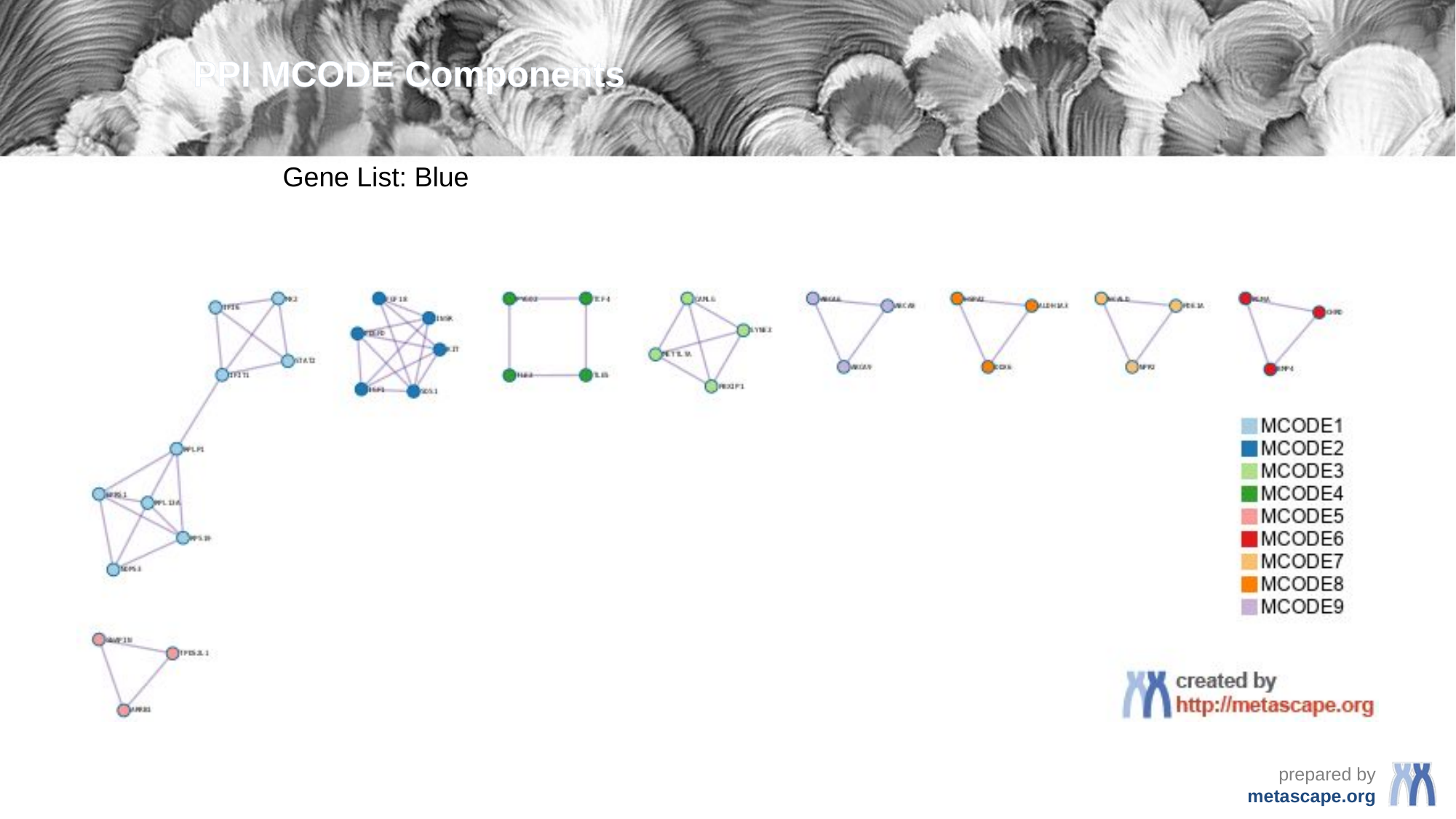

PPI MCODE Components
Gene List: Blue

## Slide 14
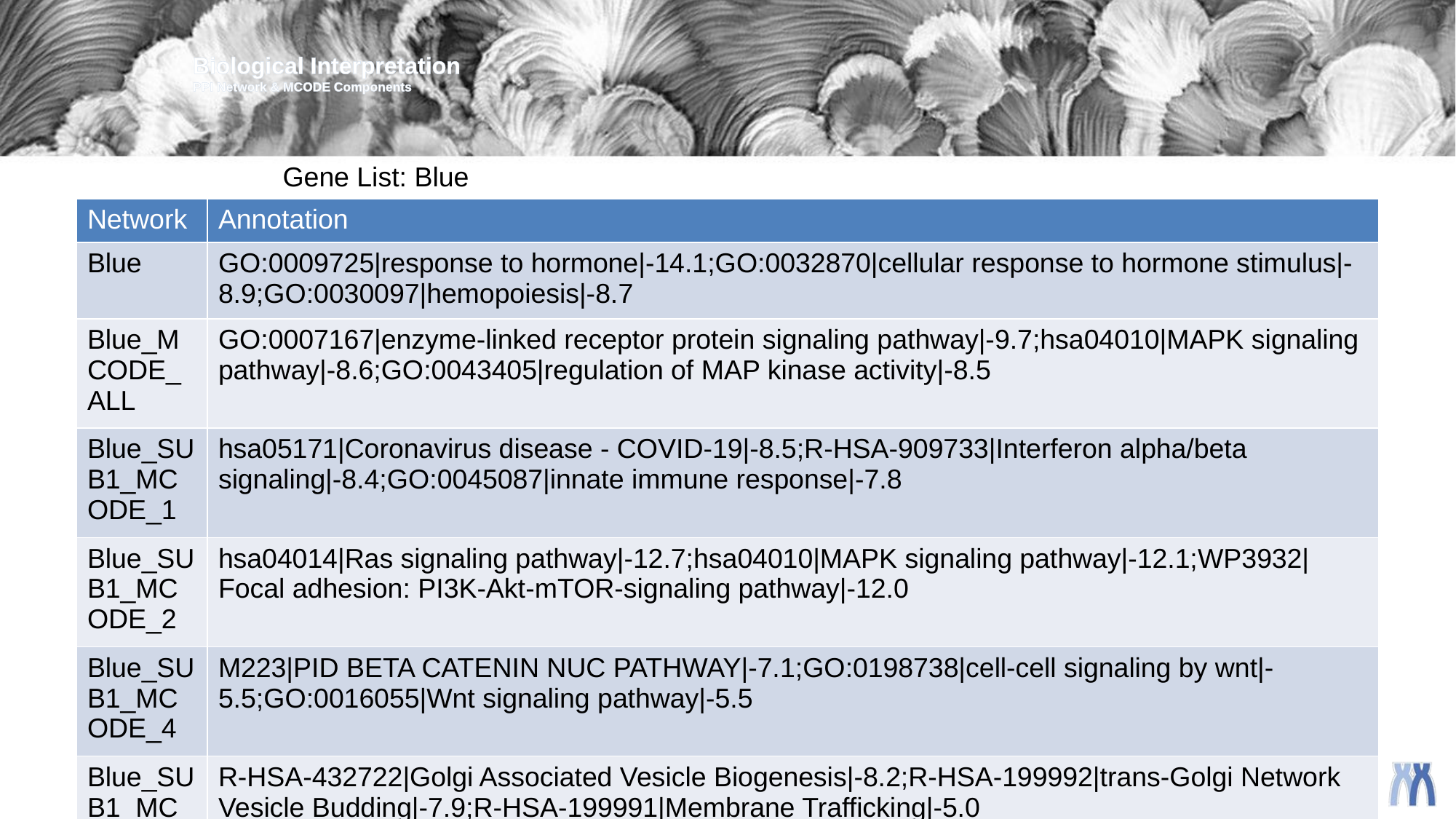

Biological Interpretation
PPI Network & MCODE Components
Gene List: Blue
| Network | Annotation |
| --- | --- |
| Blue | GO:0009725|response to hormone|-14.1;GO:0032870|cellular response to hormone stimulus|-8.9;GO:0030097|hemopoiesis|-8.7 |
| Blue\_MCODE\_ALL | GO:0007167|enzyme-linked receptor protein signaling pathway|-9.7;hsa04010|MAPK signaling pathway|-8.6;GO:0043405|regulation of MAP kinase activity|-8.5 |
| Blue\_SUB1\_MCODE\_1 | hsa05171|Coronavirus disease - COVID-19|-8.5;R-HSA-909733|Interferon alpha/beta signaling|-8.4;GO:0045087|innate immune response|-7.8 |
| Blue\_SUB1\_MCODE\_2 | hsa04014|Ras signaling pathway|-12.7;hsa04010|MAPK signaling pathway|-12.1;WP3932|Focal adhesion: PI3K-Akt-mTOR-signaling pathway|-12.0 |
| Blue\_SUB1\_MCODE\_4 | M223|PID BETA CATENIN NUC PATHWAY|-7.1;GO:0198738|cell-cell signaling by wnt|-5.5;GO:0016055|Wnt signaling pathway|-5.5 |
| Blue\_SUB1\_MCODE\_5 | R-HSA-432722|Golgi Associated Vesicle Biogenesis|-8.2;R-HSA-199992|trans-Golgi Network Vesicle Budding|-7.9;R-HSA-199991|Membrane Trafficking|-5.0 |
| Blue\_SUB1\_MCODE\_6 | M181|PID BMP PATHWAY|-8.6;GO:0030509|BMP signaling pathway|-7.6;hsa04350|TGF-beta signaling pathway|-7.5 |
| Blue\_SUB2\_MCODE\_9 | hsa02010|ABC transporters|-8.5;R-HSA-382556|ABC-family proteins mediated transport|-7.4;GO:0006869|lipid transport|-5.8 |

## Slide 15
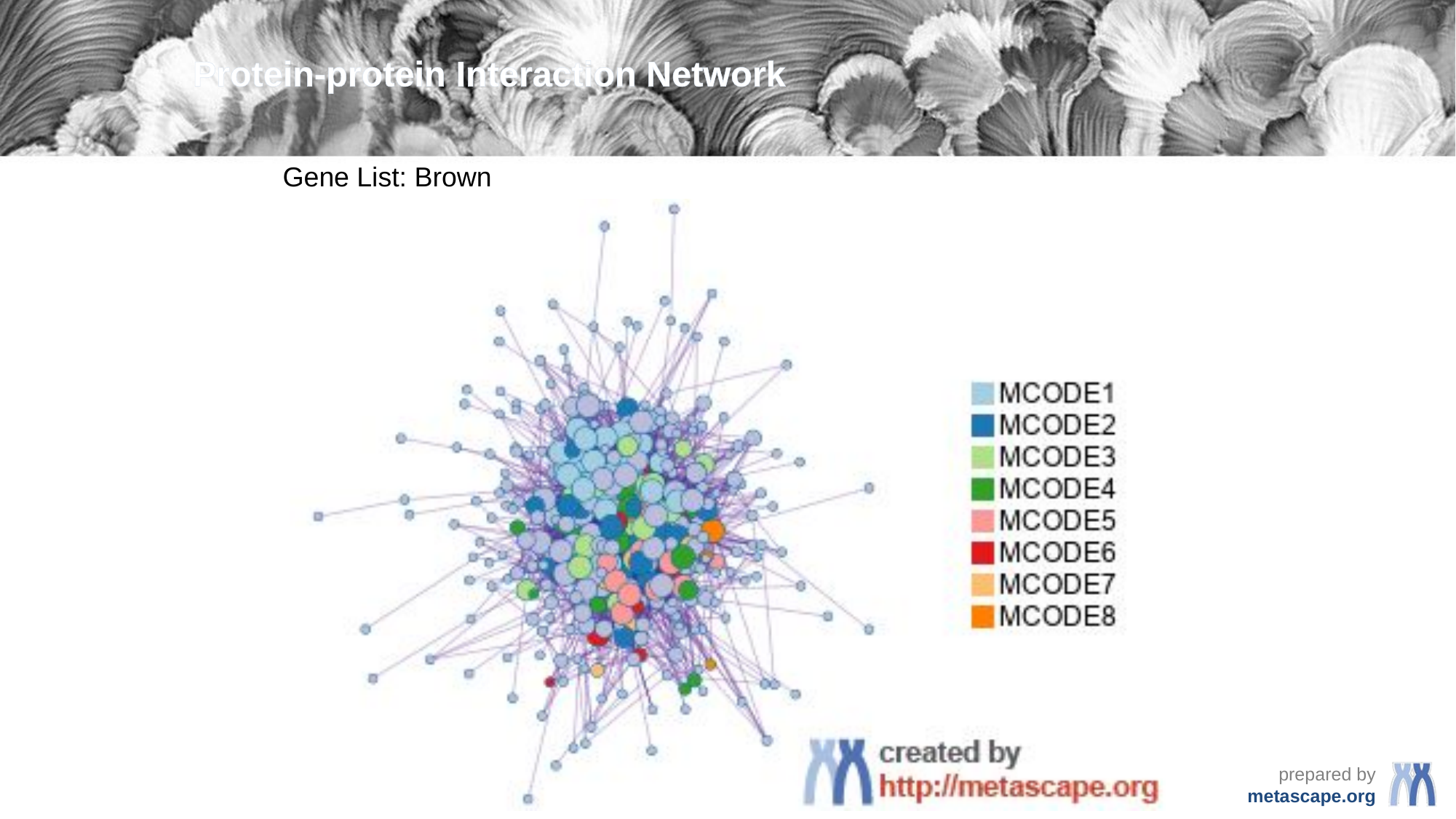

Protein-protein Interaction Network
Gene List: Brown

## Slide 16
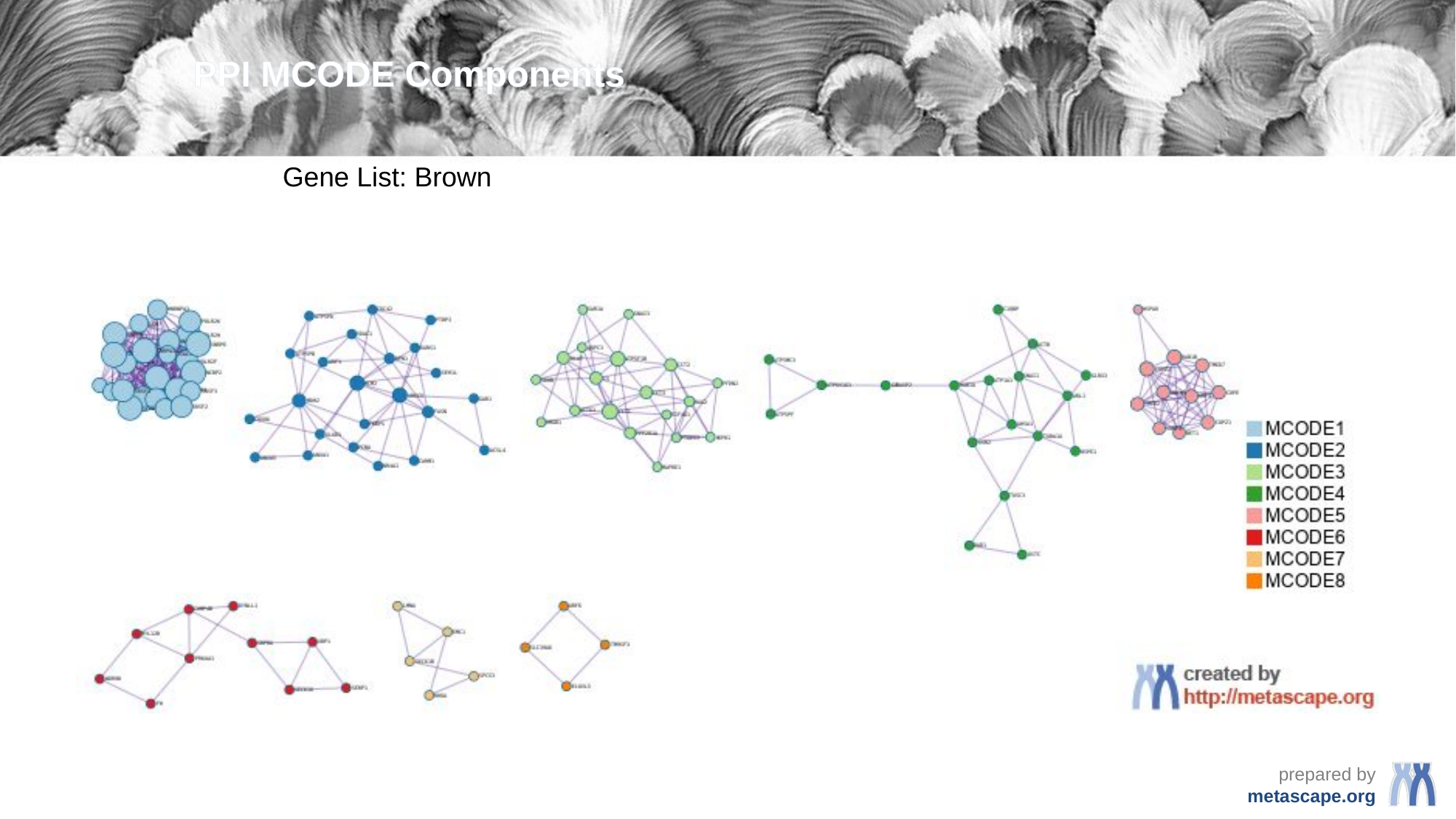

PPI MCODE Components
Gene List: Brown

## Slide 17
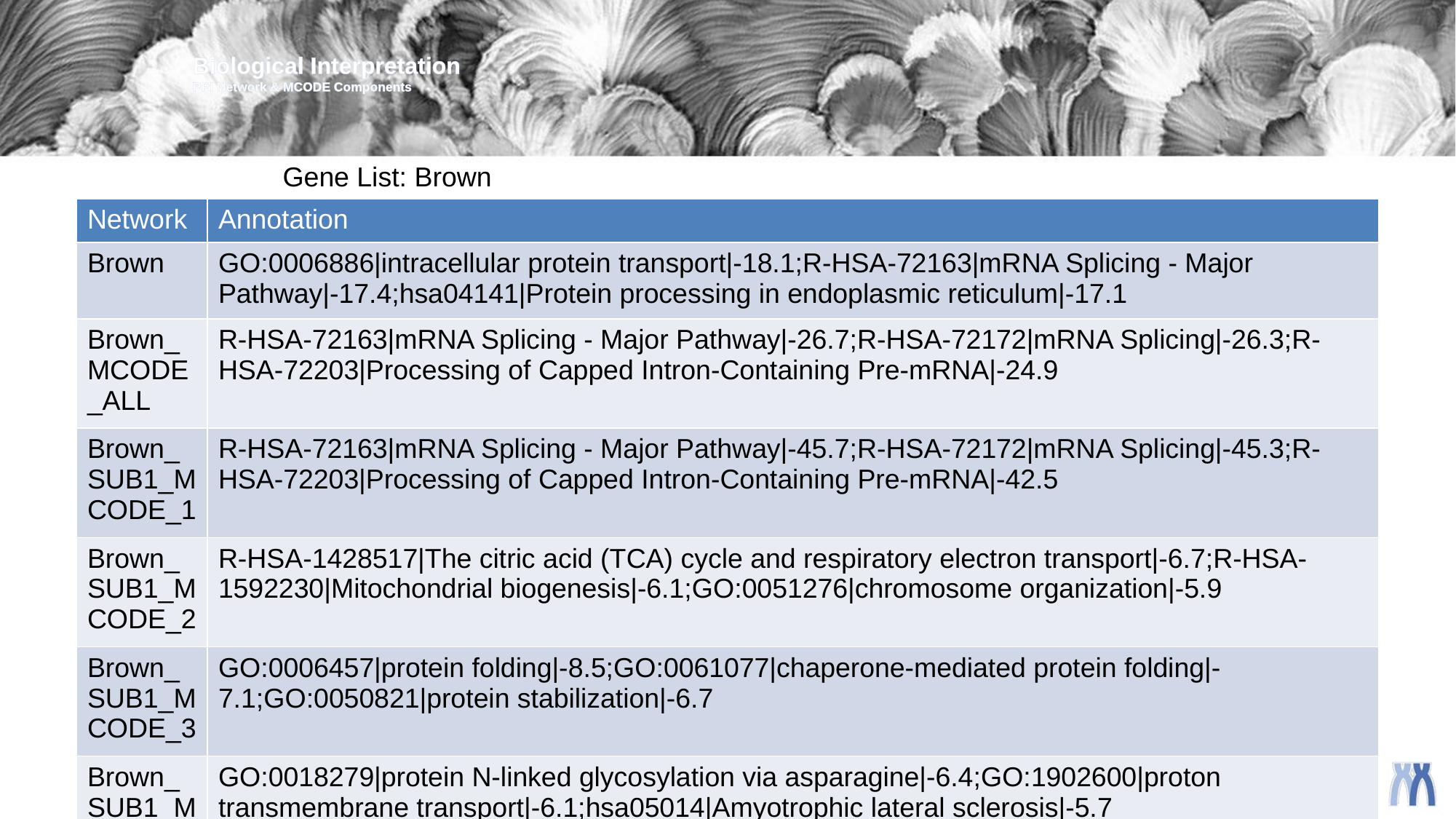

Biological Interpretation
PPI Network & MCODE Components
Gene List: Brown
| Network | Annotation |
| --- | --- |
| Brown | GO:0006886|intracellular protein transport|-18.1;R-HSA-72163|mRNA Splicing - Major Pathway|-17.4;hsa04141|Protein processing in endoplasmic reticulum|-17.1 |
| Brown\_MCODE\_ALL | R-HSA-72163|mRNA Splicing - Major Pathway|-26.7;R-HSA-72172|mRNA Splicing|-26.3;R-HSA-72203|Processing of Capped Intron-Containing Pre-mRNA|-24.9 |
| Brown\_SUB1\_MCODE\_1 | R-HSA-72163|mRNA Splicing - Major Pathway|-45.7;R-HSA-72172|mRNA Splicing|-45.3;R-HSA-72203|Processing of Capped Intron-Containing Pre-mRNA|-42.5 |
| Brown\_SUB1\_MCODE\_2 | R-HSA-1428517|The citric acid (TCA) cycle and respiratory electron transport|-6.7;R-HSA-1592230|Mitochondrial biogenesis|-6.1;GO:0051276|chromosome organization|-5.9 |
| Brown\_SUB1\_MCODE\_3 | GO:0006457|protein folding|-8.5;GO:0061077|chaperone-mediated protein folding|-7.1;GO:0050821|protein stabilization|-6.7 |
| Brown\_SUB1\_MCODE\_4 | GO:0018279|protein N-linked glycosylation via asparagine|-6.4;GO:1902600|proton transmembrane transport|-6.1;hsa05014|Amyotrophic lateral sclerosis|-5.7 |
| Brown\_SUB1\_MCODE\_5 | R-HSA-6811434|COPI-dependent Golgi-to-ER retrograde traffic|-20.8;R-HSA-6807878|COPI-mediated anterograde transport|-20.7;R-HSA-8856688|Golgi-to-ER retrograde transport|-19.6 |
| Brown\_SUB1\_MCODE\_6 | GO:0045047|protein targeting to ER|-6.3;R-HSA-381038|XBP1(S) activates chaperone genes|-6.3;R-HSA-381070|IRE1alpha activates chaperones|-6.2 |
| Brown\_SUB1\_MCODE\_7 | GO:0033365|protein localization to organelle|-5.8;GO:0072594|establishment of protein localization to organelle|-4.8;GO:0051668|localization within membrane|-4.2 |

## Slide 18
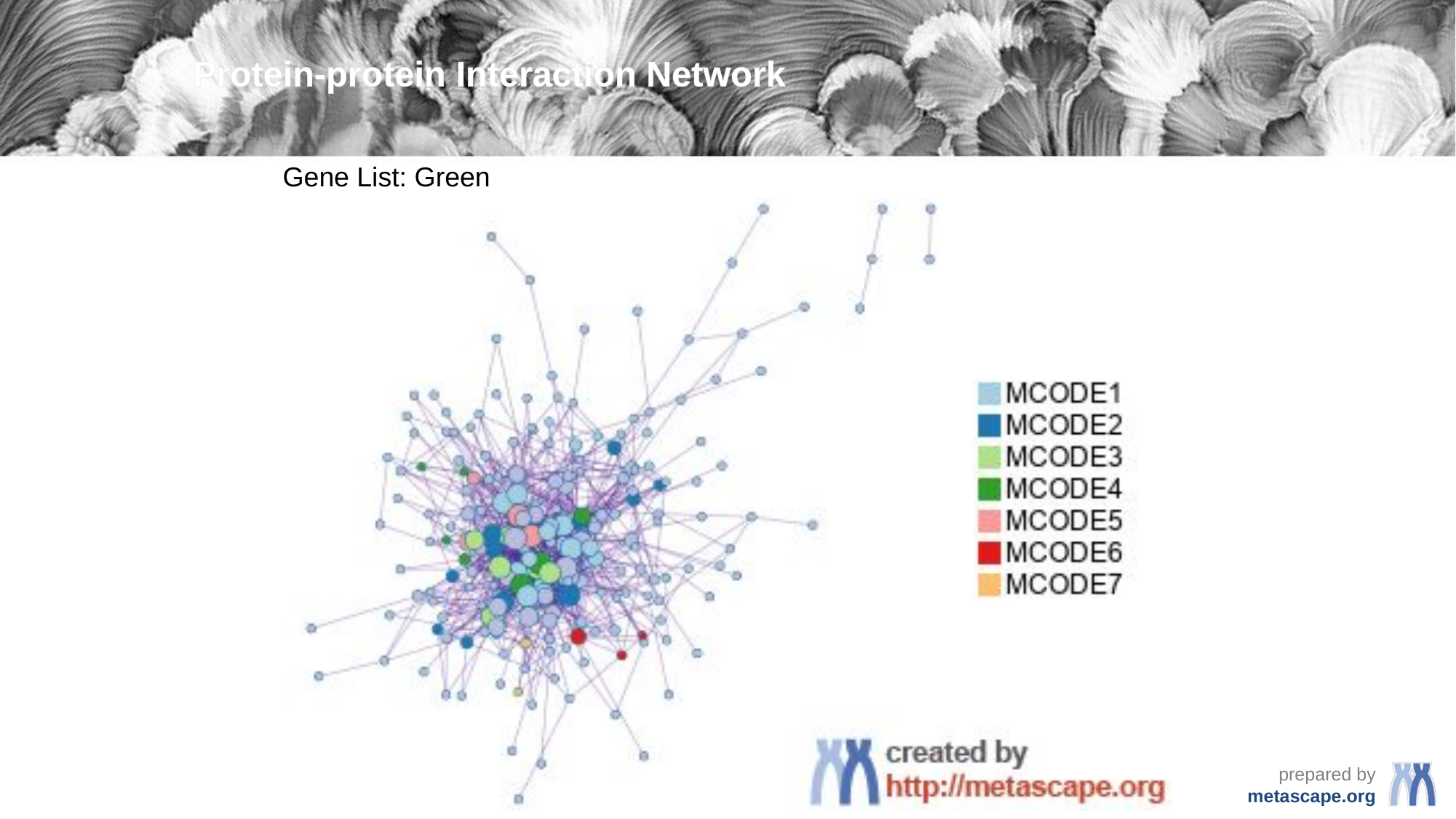

Protein-protein Interaction Network
Gene List: Green

## Slide 19
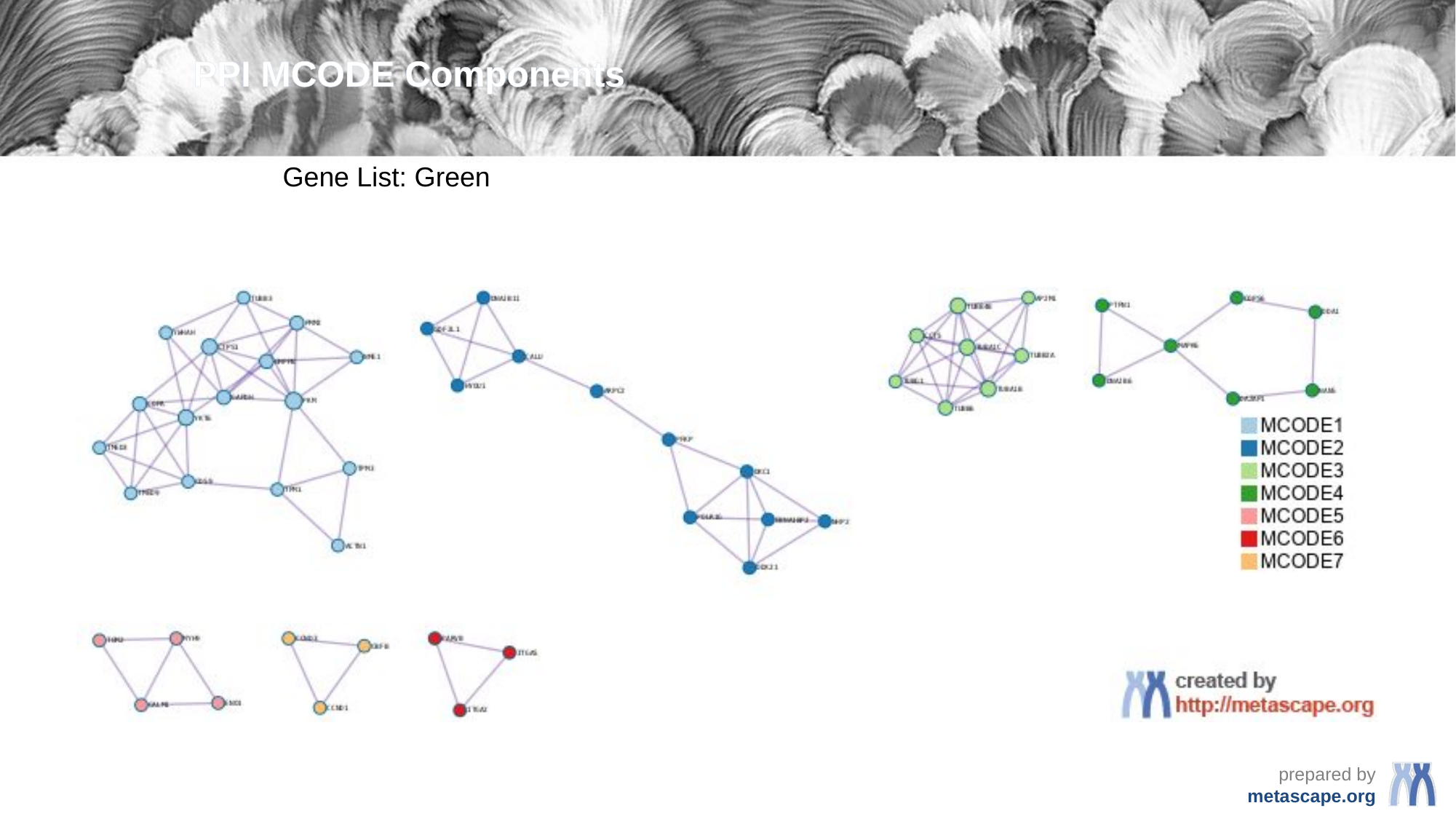

PPI MCODE Components
Gene List: Green

## Slide 20
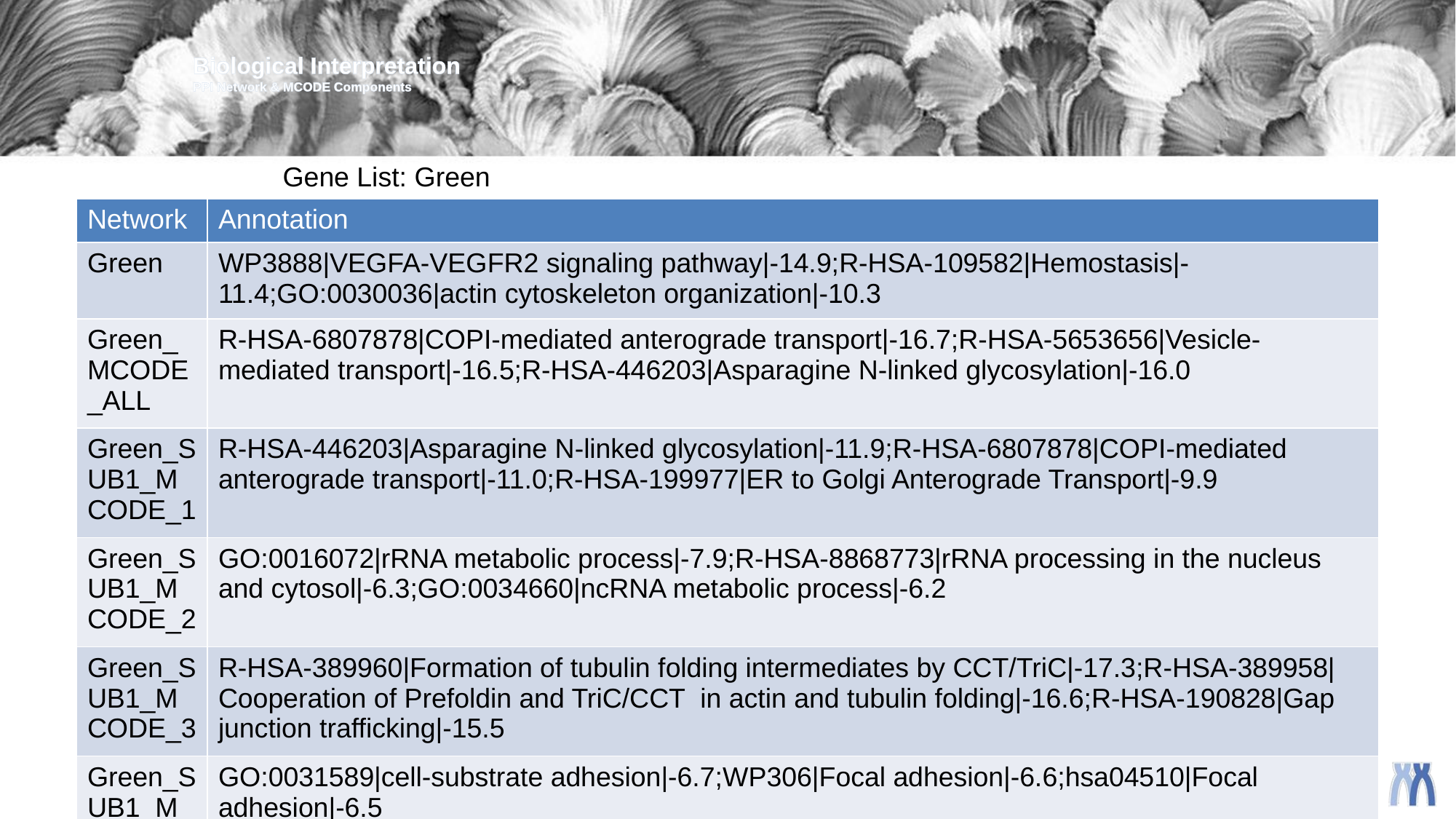

Biological Interpretation
PPI Network & MCODE Components
Gene List: Green
| Network | Annotation |
| --- | --- |
| Green | WP3888|VEGFA-VEGFR2 signaling pathway|-14.9;R-HSA-109582|Hemostasis|-11.4;GO:0030036|actin cytoskeleton organization|-10.3 |
| Green\_MCODE\_ALL | R-HSA-6807878|COPI-mediated anterograde transport|-16.7;R-HSA-5653656|Vesicle-mediated transport|-16.5;R-HSA-446203|Asparagine N-linked glycosylation|-16.0 |
| Green\_SUB1\_MCODE\_1 | R-HSA-446203|Asparagine N-linked glycosylation|-11.9;R-HSA-6807878|COPI-mediated anterograde transport|-11.0;R-HSA-199977|ER to Golgi Anterograde Transport|-9.9 |
| Green\_SUB1\_MCODE\_2 | GO:0016072|rRNA metabolic process|-7.9;R-HSA-8868773|rRNA processing in the nucleus and cytosol|-6.3;GO:0034660|ncRNA metabolic process|-6.2 |
| Green\_SUB1\_MCODE\_3 | R-HSA-389960|Formation of tubulin folding intermediates by CCT/TriC|-17.3;R-HSA-389958|Cooperation of Prefoldin and TriC/CCT in actin and tubulin folding|-16.6;R-HSA-190828|Gap junction trafficking|-15.5 |
| Green\_SUB1\_MCODE\_6 | GO:0031589|cell-substrate adhesion|-6.7;WP306|Focal adhesion|-6.6;hsa04510|Focal adhesion|-6.5 |
| Green\_SUB1\_MCODE\_7 | R-HSA-8934593|Regulation of RUNX1 Expression and Activity|-9.8;R-HSA-8878171|Transcriptional regulation by RUNX1|-6.3 |

## Slide 21
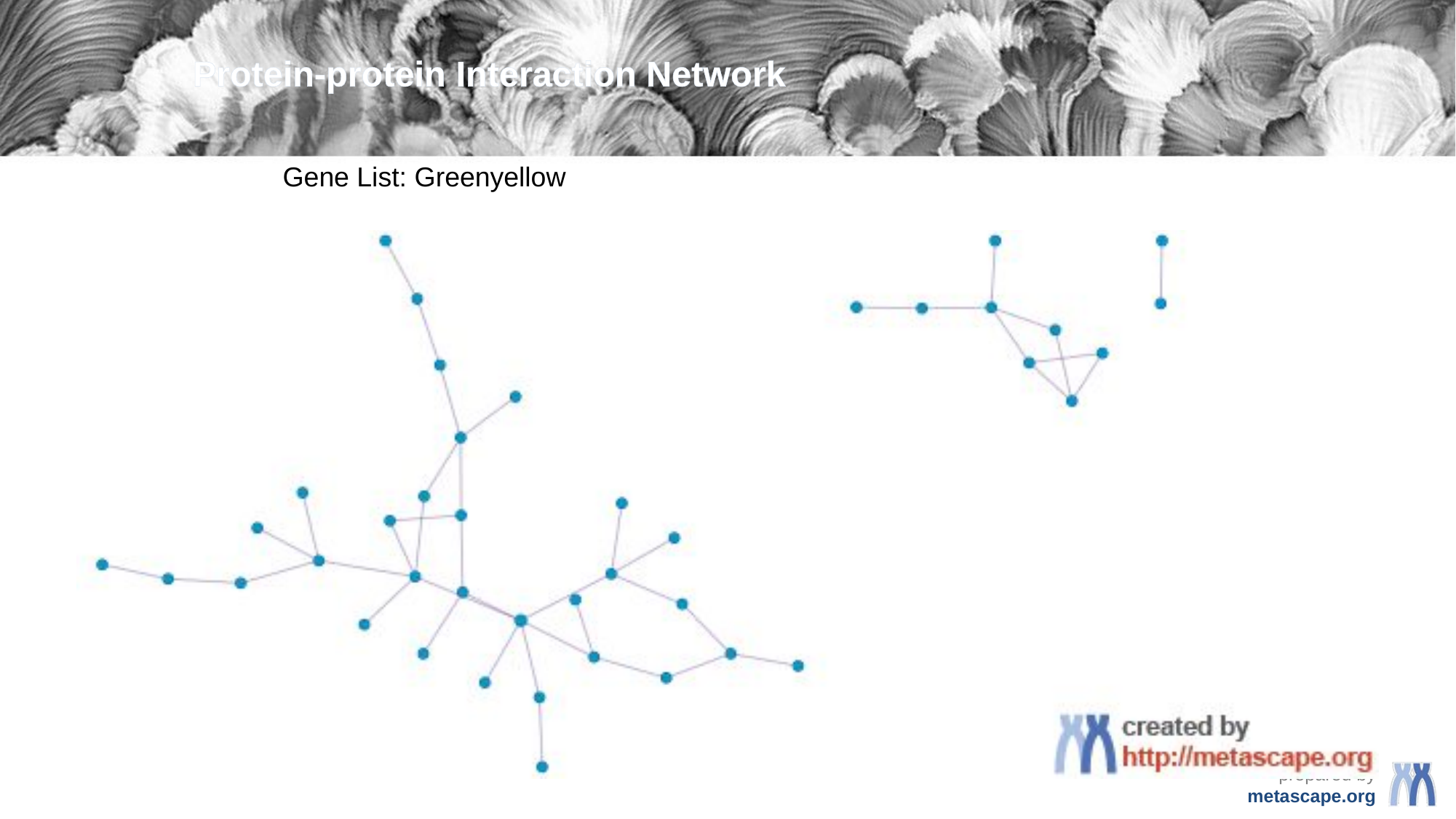

Protein-protein Interaction Network
Gene List: Greenyellow

## Slide 22
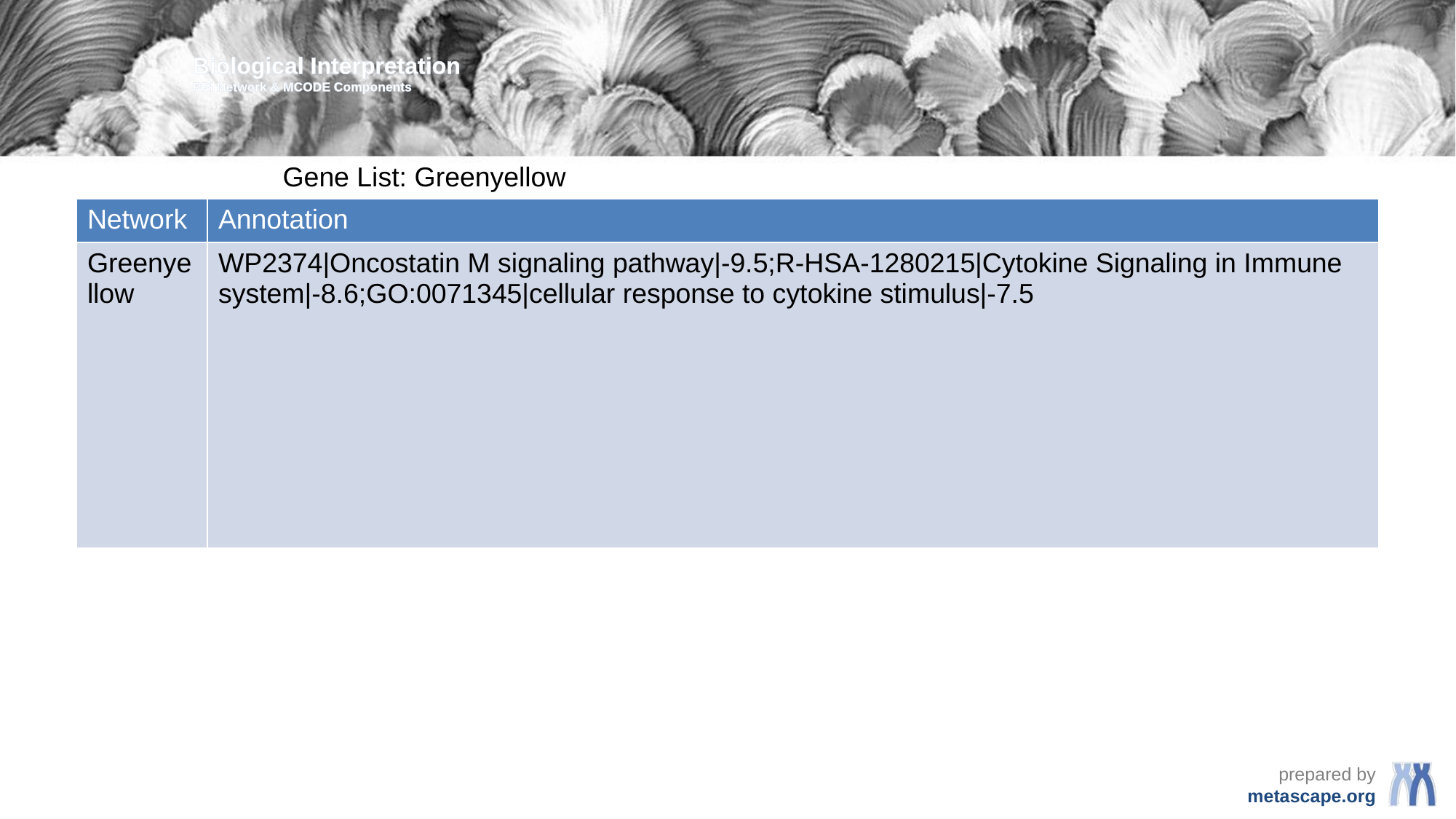

Biological Interpretation
PPI Network & MCODE Components
Gene List: Greenyellow
| Network | Annotation |
| --- | --- |
| Greenyellow | WP2374|Oncostatin M signaling pathway|-9.5;R-HSA-1280215|Cytokine Signaling in Immune system|-8.6;GO:0071345|cellular response to cytokine stimulus|-7.5 |

## Slide 23
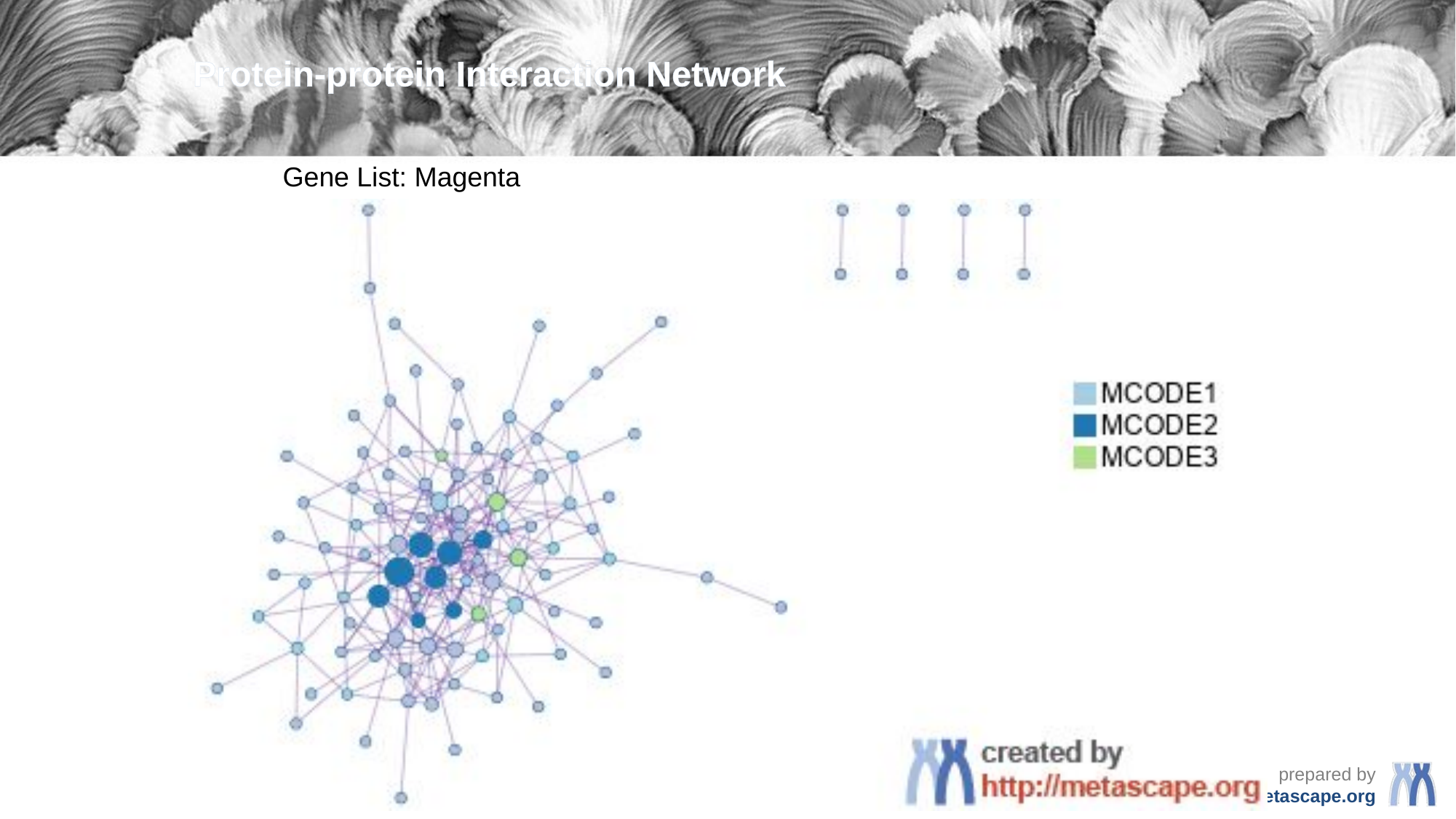

Protein-protein Interaction Network
Gene List: Magenta

## Slide 24
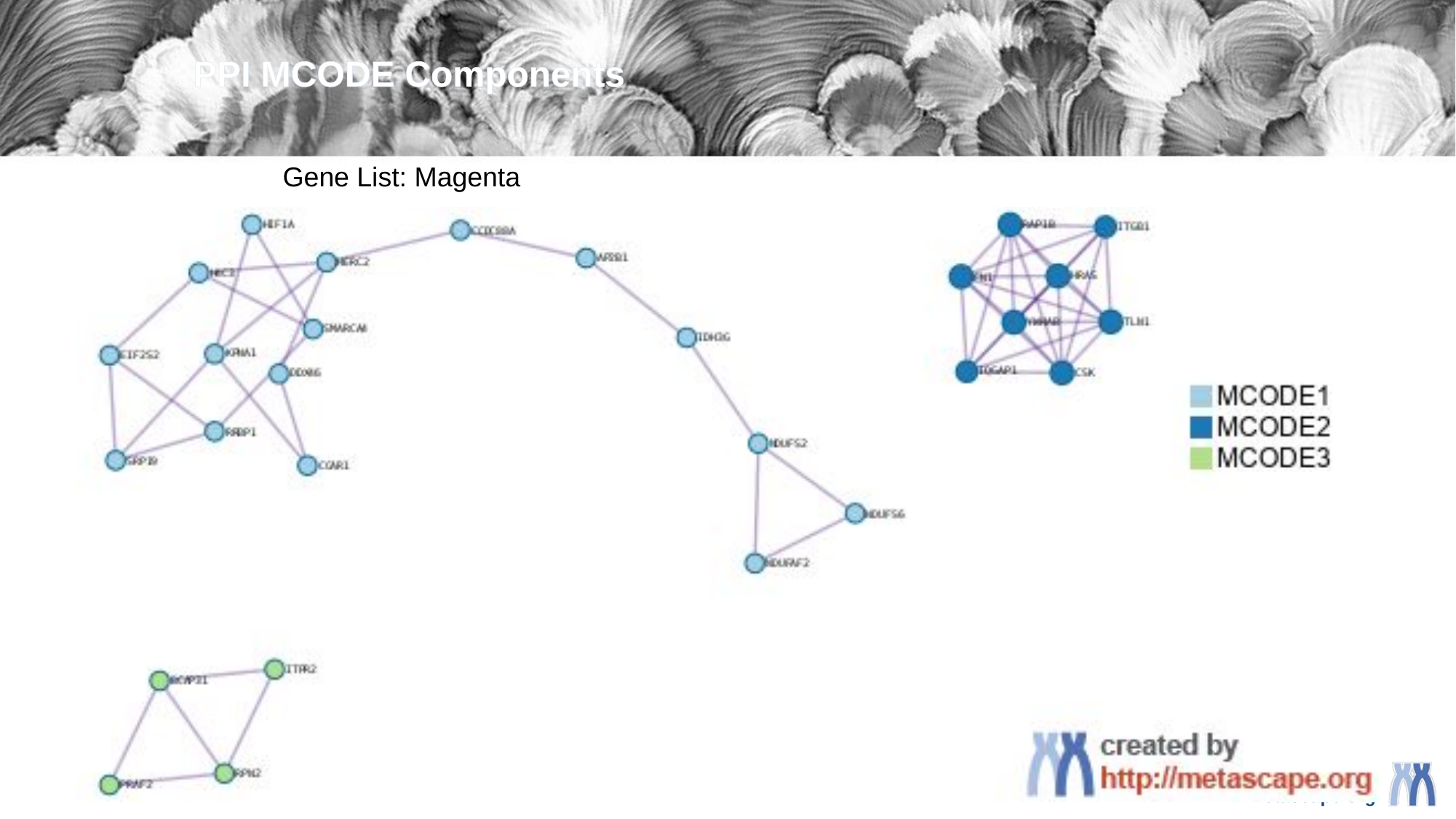

PPI MCODE Components
Gene List: Magenta

## Slide 25
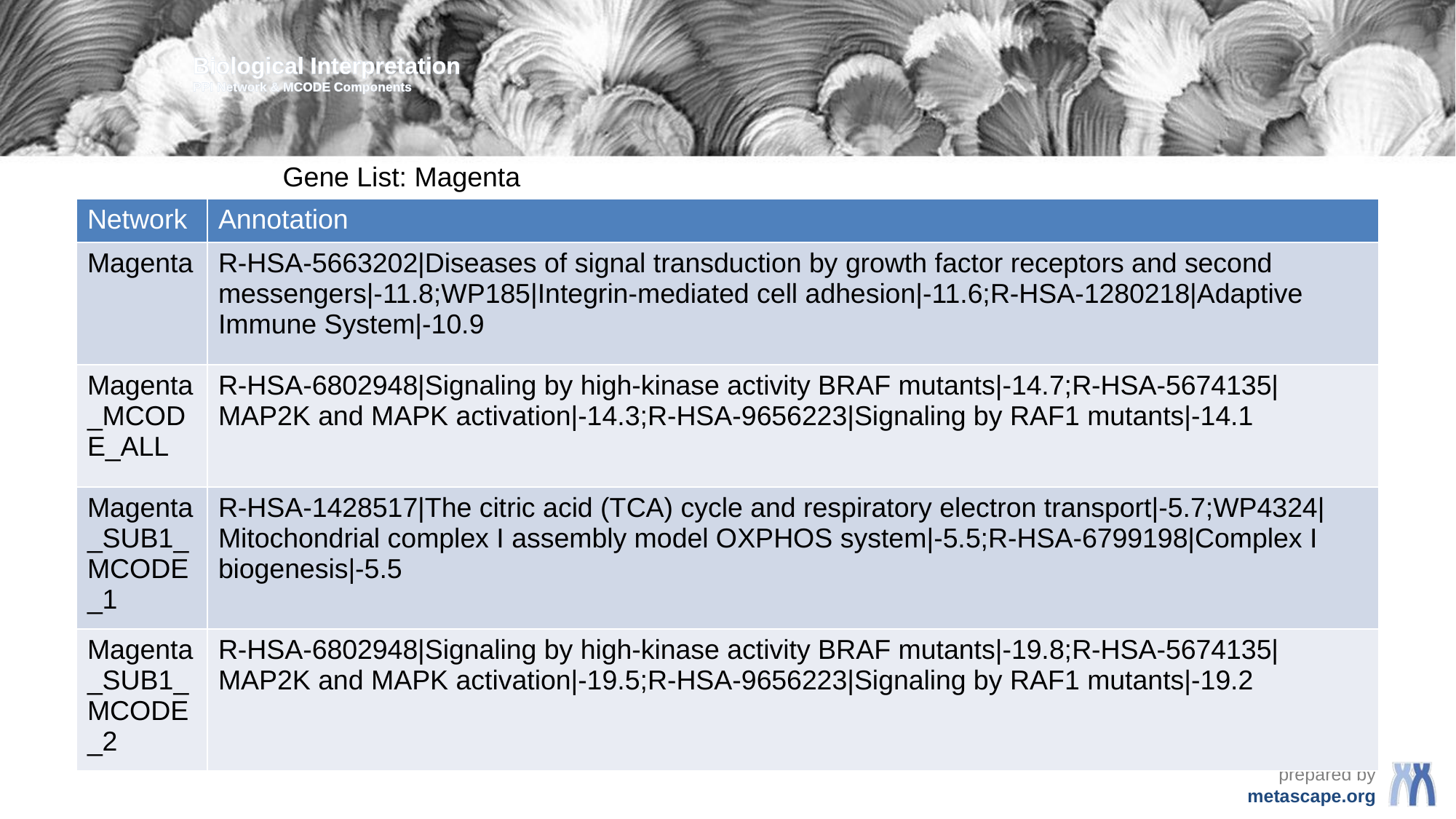

Biological Interpretation
PPI Network & MCODE Components
Gene List: Magenta
| Network | Annotation |
| --- | --- |
| Magenta | R-HSA-5663202|Diseases of signal transduction by growth factor receptors and second messengers|-11.8;WP185|Integrin-mediated cell adhesion|-11.6;R-HSA-1280218|Adaptive Immune System|-10.9 |
| Magenta\_MCODE\_ALL | R-HSA-6802948|Signaling by high-kinase activity BRAF mutants|-14.7;R-HSA-5674135|MAP2K and MAPK activation|-14.3;R-HSA-9656223|Signaling by RAF1 mutants|-14.1 |
| Magenta\_SUB1\_MCODE\_1 | R-HSA-1428517|The citric acid (TCA) cycle and respiratory electron transport|-5.7;WP4324|Mitochondrial complex I assembly model OXPHOS system|-5.5;R-HSA-6799198|Complex I biogenesis|-5.5 |
| Magenta\_SUB1\_MCODE\_2 | R-HSA-6802948|Signaling by high-kinase activity BRAF mutants|-19.8;R-HSA-5674135|MAP2K and MAPK activation|-19.5;R-HSA-9656223|Signaling by RAF1 mutants|-19.2 |

## Slide 26
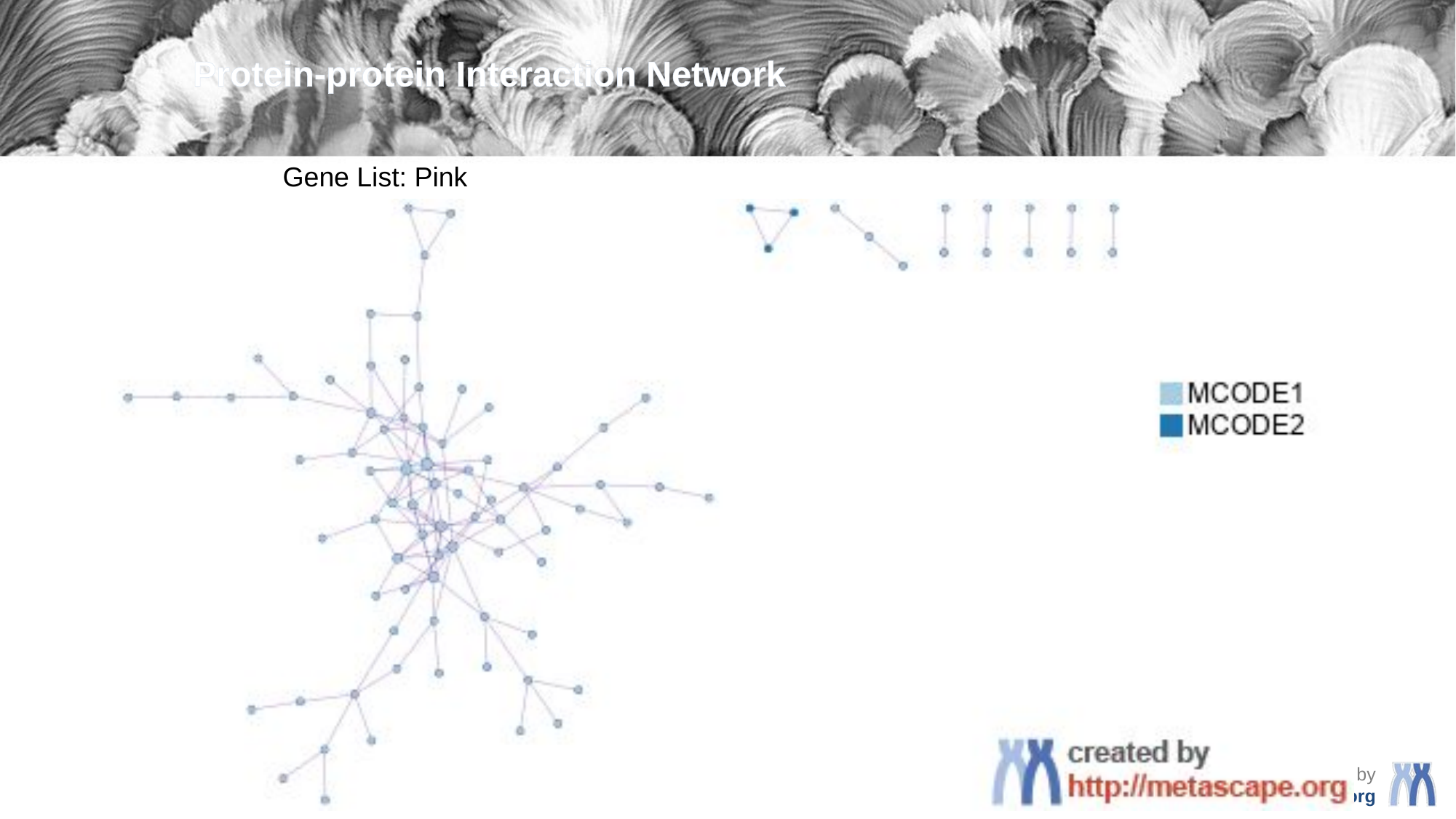

Protein-protein Interaction Network
Gene List: Pink

## Slide 27
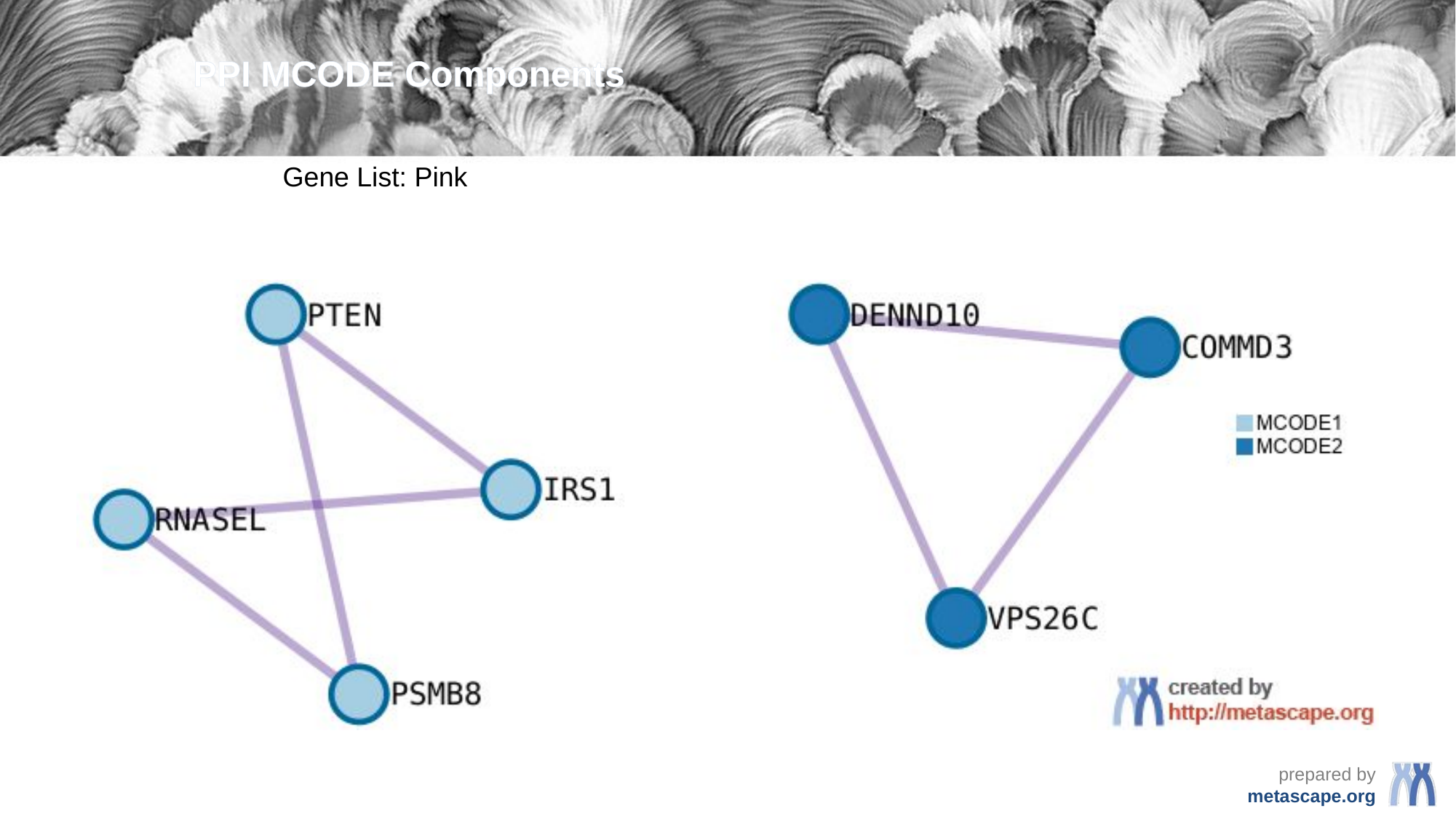

PPI MCODE Components
Gene List: Pink

## Slide 28
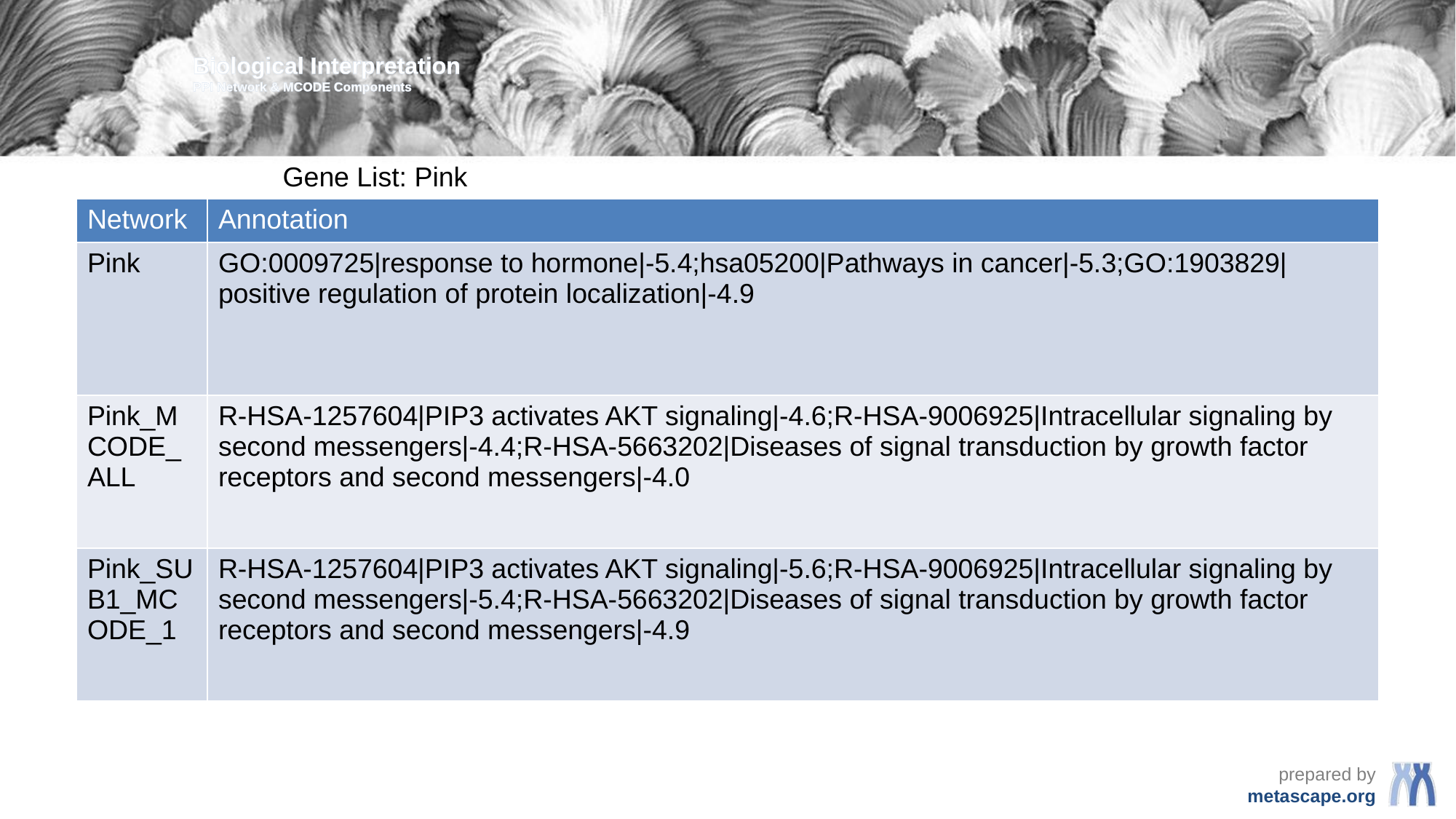

Biological Interpretation
PPI Network & MCODE Components
Gene List: Pink
| Network | Annotation |
| --- | --- |
| Pink | GO:0009725|response to hormone|-5.4;hsa05200|Pathways in cancer|-5.3;GO:1903829|positive regulation of protein localization|-4.9 |
| Pink\_MCODE\_ALL | R-HSA-1257604|PIP3 activates AKT signaling|-4.6;R-HSA-9006925|Intracellular signaling by second messengers|-4.4;R-HSA-5663202|Diseases of signal transduction by growth factor receptors and second messengers|-4.0 |
| Pink\_SUB1\_MCODE\_1 | R-HSA-1257604|PIP3 activates AKT signaling|-5.6;R-HSA-9006925|Intracellular signaling by second messengers|-5.4;R-HSA-5663202|Diseases of signal transduction by growth factor receptors and second messengers|-4.9 |

## Slide 29
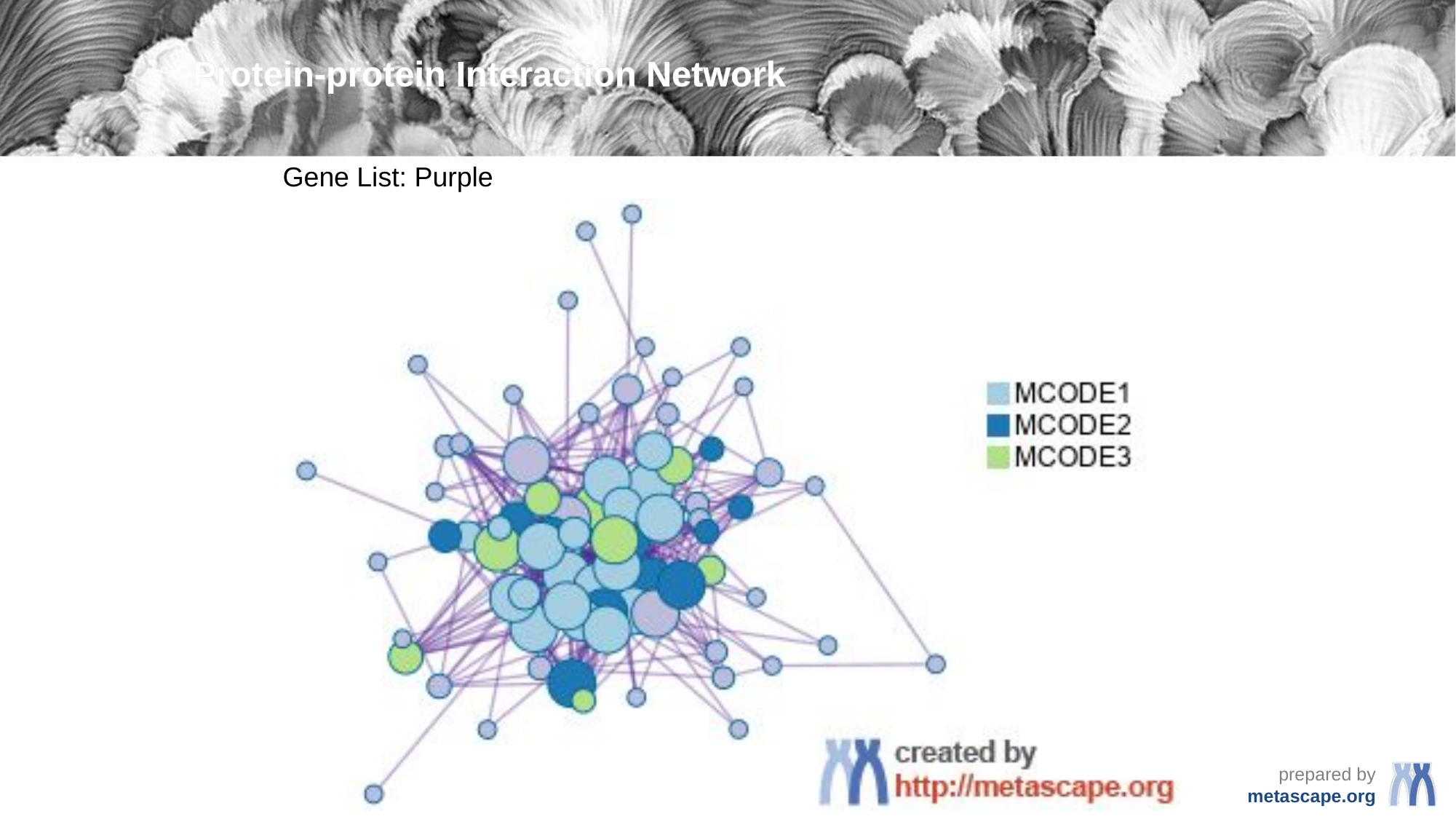

Protein-protein Interaction Network
Gene List: Purple

## Slide 30
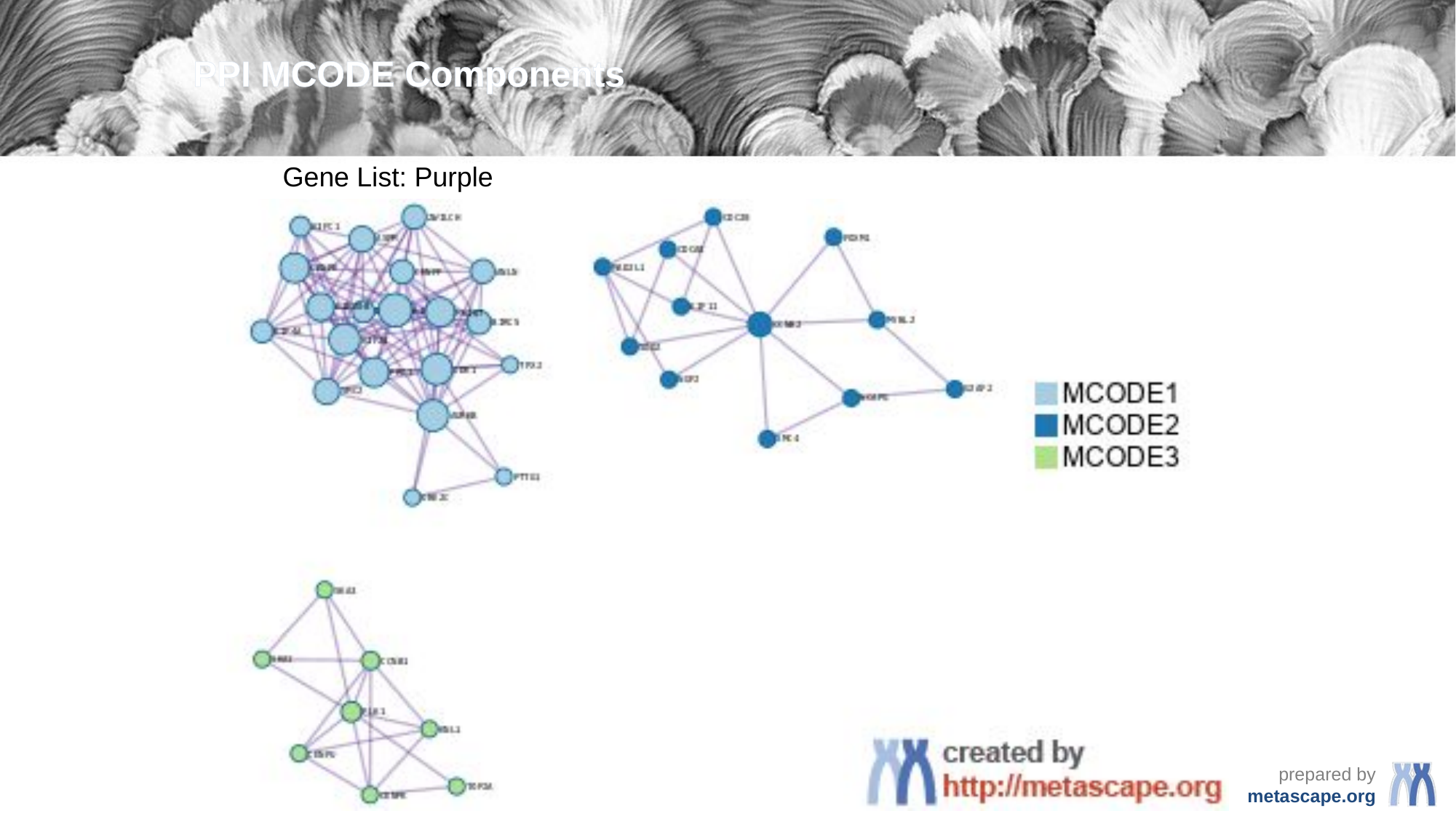

PPI MCODE Components
Gene List: Purple

## Slide 31
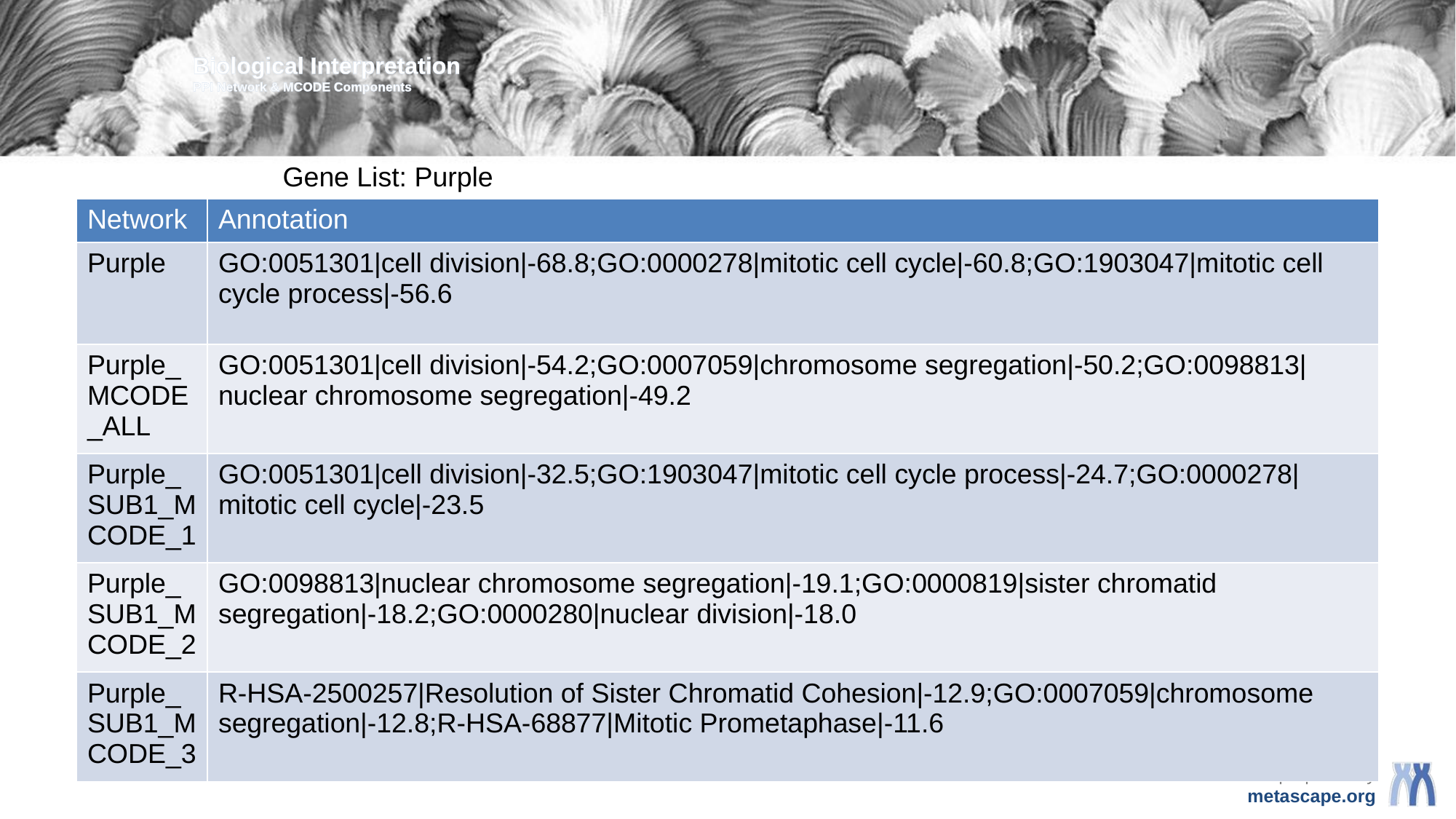

Biological Interpretation
PPI Network & MCODE Components
Gene List: Purple
| Network | Annotation |
| --- | --- |
| Purple | GO:0051301|cell division|-68.8;GO:0000278|mitotic cell cycle|-60.8;GO:1903047|mitotic cell cycle process|-56.6 |
| Purple\_MCODE\_ALL | GO:0051301|cell division|-54.2;GO:0007059|chromosome segregation|-50.2;GO:0098813|nuclear chromosome segregation|-49.2 |
| Purple\_SUB1\_MCODE\_1 | GO:0051301|cell division|-32.5;GO:1903047|mitotic cell cycle process|-24.7;GO:0000278|mitotic cell cycle|-23.5 |
| Purple\_SUB1\_MCODE\_2 | GO:0098813|nuclear chromosome segregation|-19.1;GO:0000819|sister chromatid segregation|-18.2;GO:0000280|nuclear division|-18.0 |
| Purple\_SUB1\_MCODE\_3 | R-HSA-2500257|Resolution of Sister Chromatid Cohesion|-12.9;GO:0007059|chromosome segregation|-12.8;R-HSA-68877|Mitotic Prometaphase|-11.6 |

## Slide 32
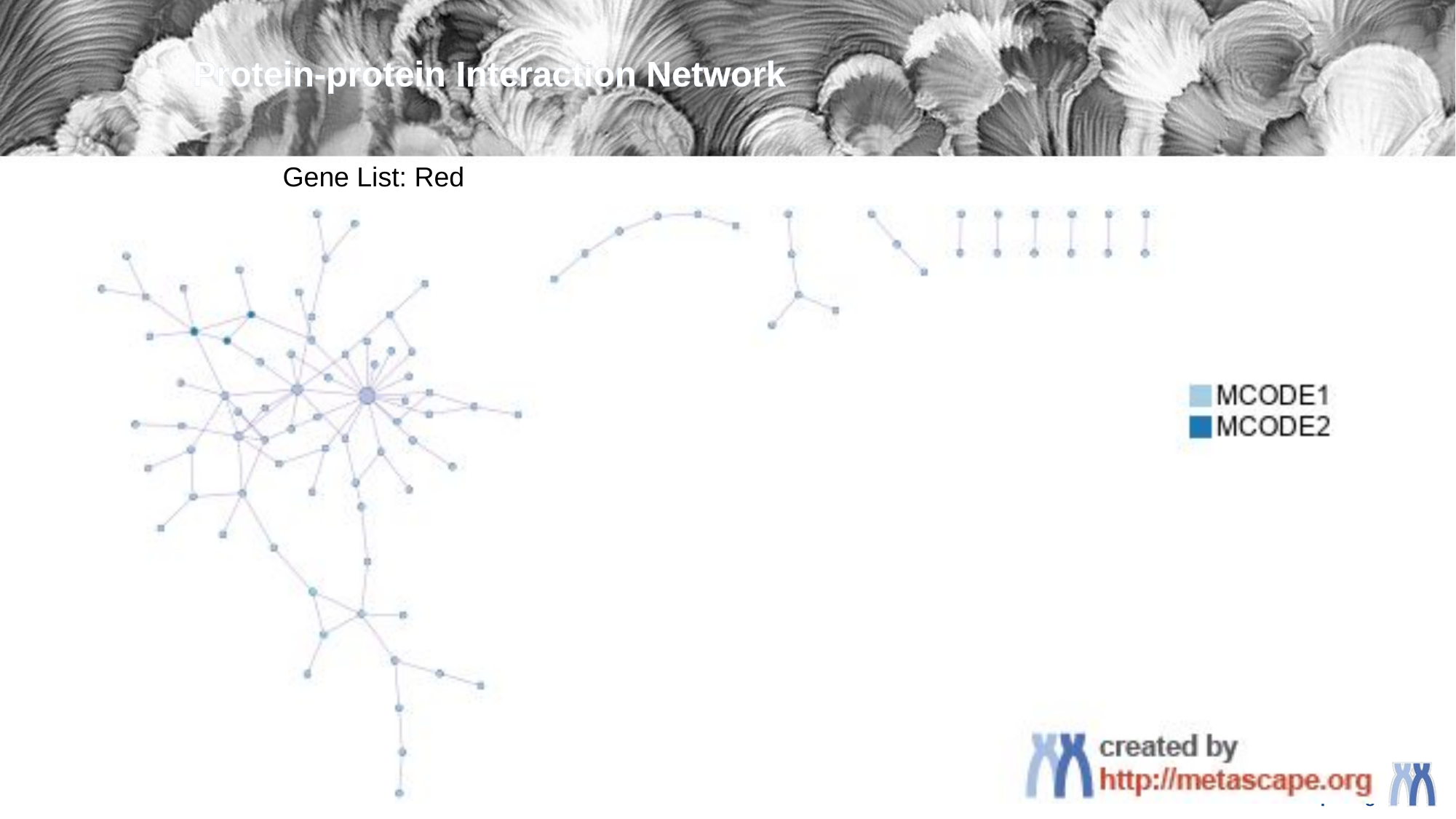

Protein-protein Interaction Network
Gene List: Red

## Slide 33
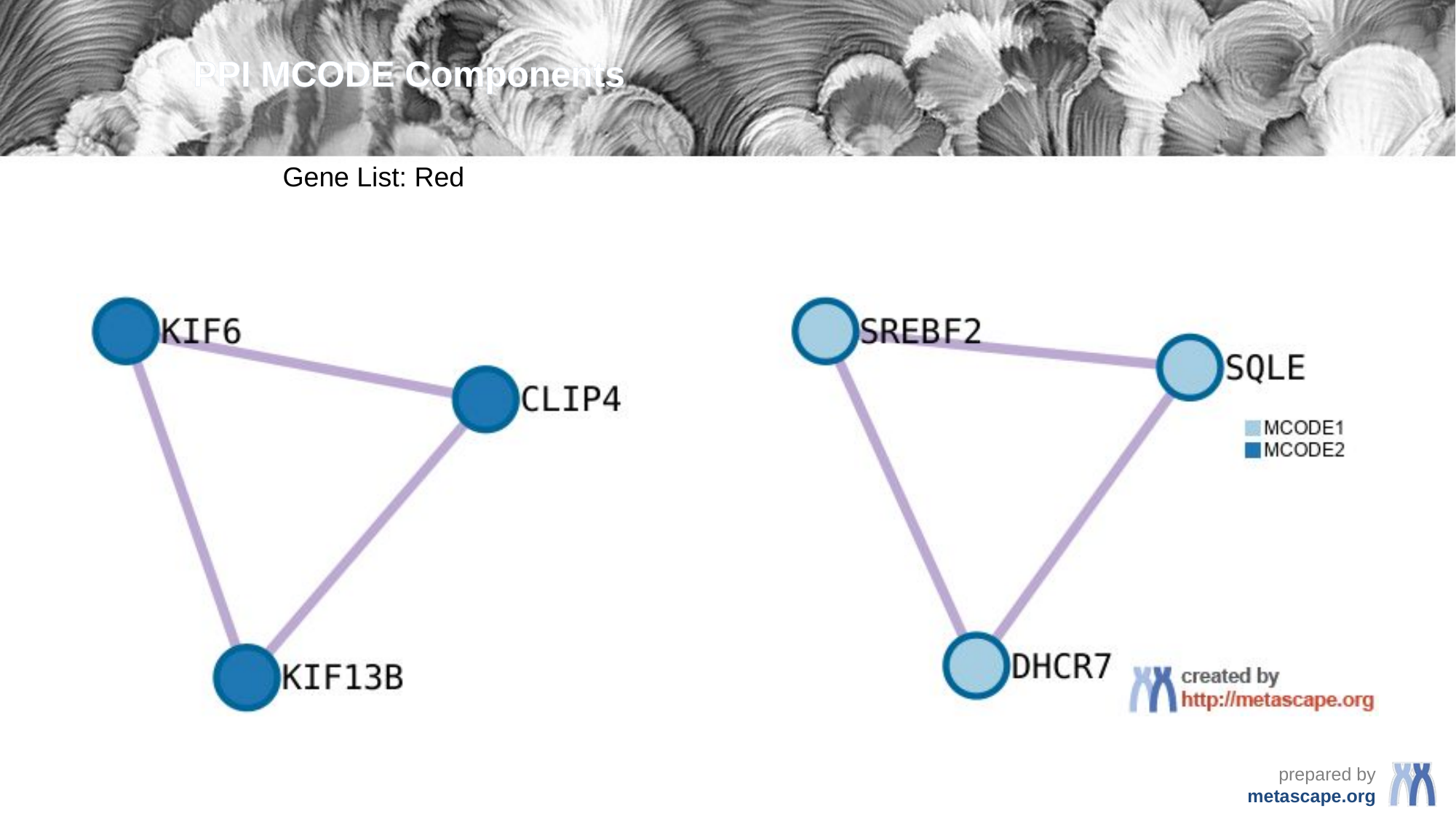

PPI MCODE Components
Gene List: Red

## Slide 34
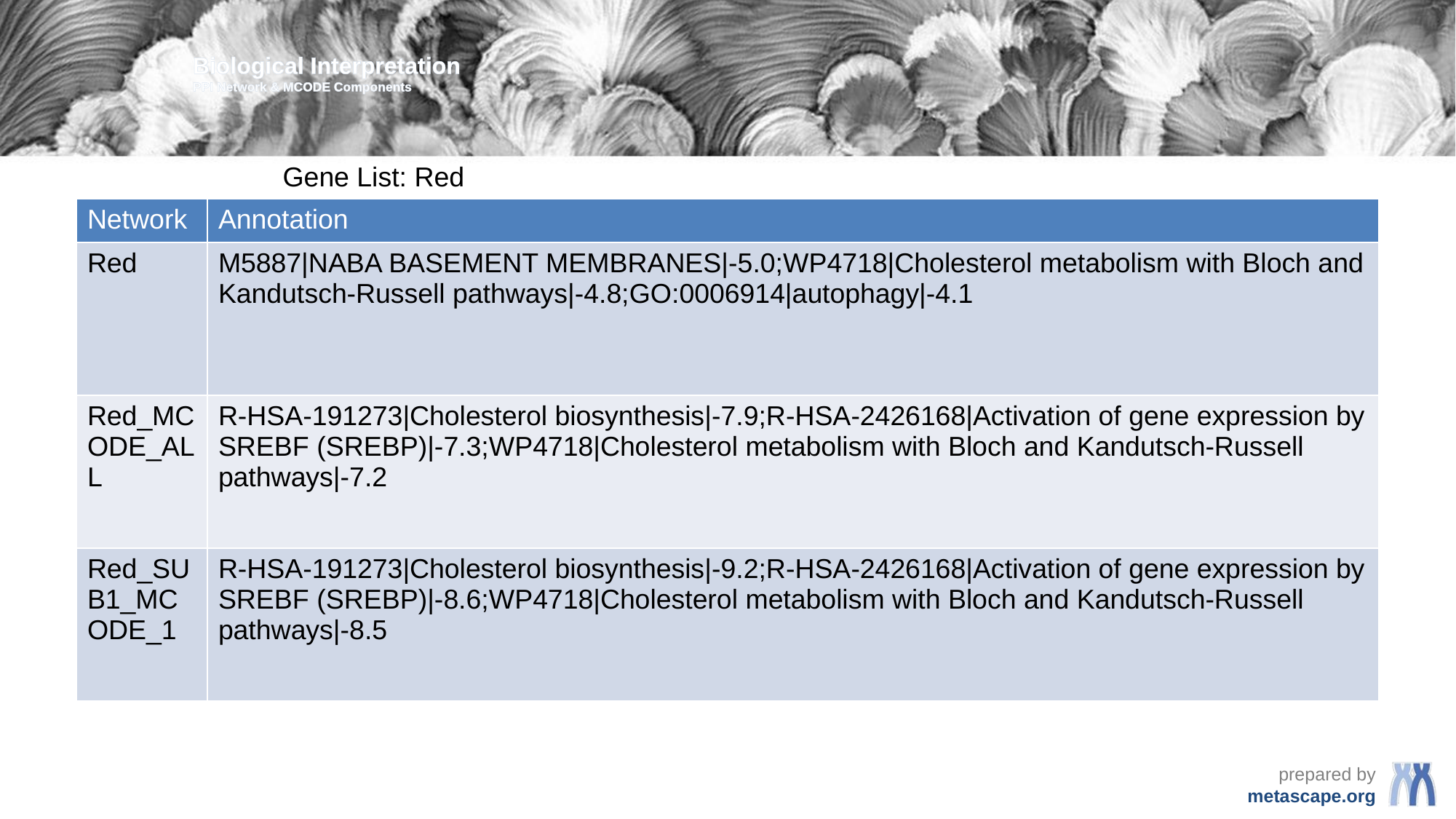

Biological Interpretation
PPI Network & MCODE Components
Gene List: Red
| Network | Annotation |
| --- | --- |
| Red | M5887|NABA BASEMENT MEMBRANES|-5.0;WP4718|Cholesterol metabolism with Bloch and Kandutsch-Russell pathways|-4.8;GO:0006914|autophagy|-4.1 |
| Red\_MCODE\_ALL | R-HSA-191273|Cholesterol biosynthesis|-7.9;R-HSA-2426168|Activation of gene expression by SREBF (SREBP)|-7.3;WP4718|Cholesterol metabolism with Bloch and Kandutsch-Russell pathways|-7.2 |
| Red\_SUB1\_MCODE\_1 | R-HSA-191273|Cholesterol biosynthesis|-9.2;R-HSA-2426168|Activation of gene expression by SREBF (SREBP)|-8.6;WP4718|Cholesterol metabolism with Bloch and Kandutsch-Russell pathways|-8.5 |

## Slide 35
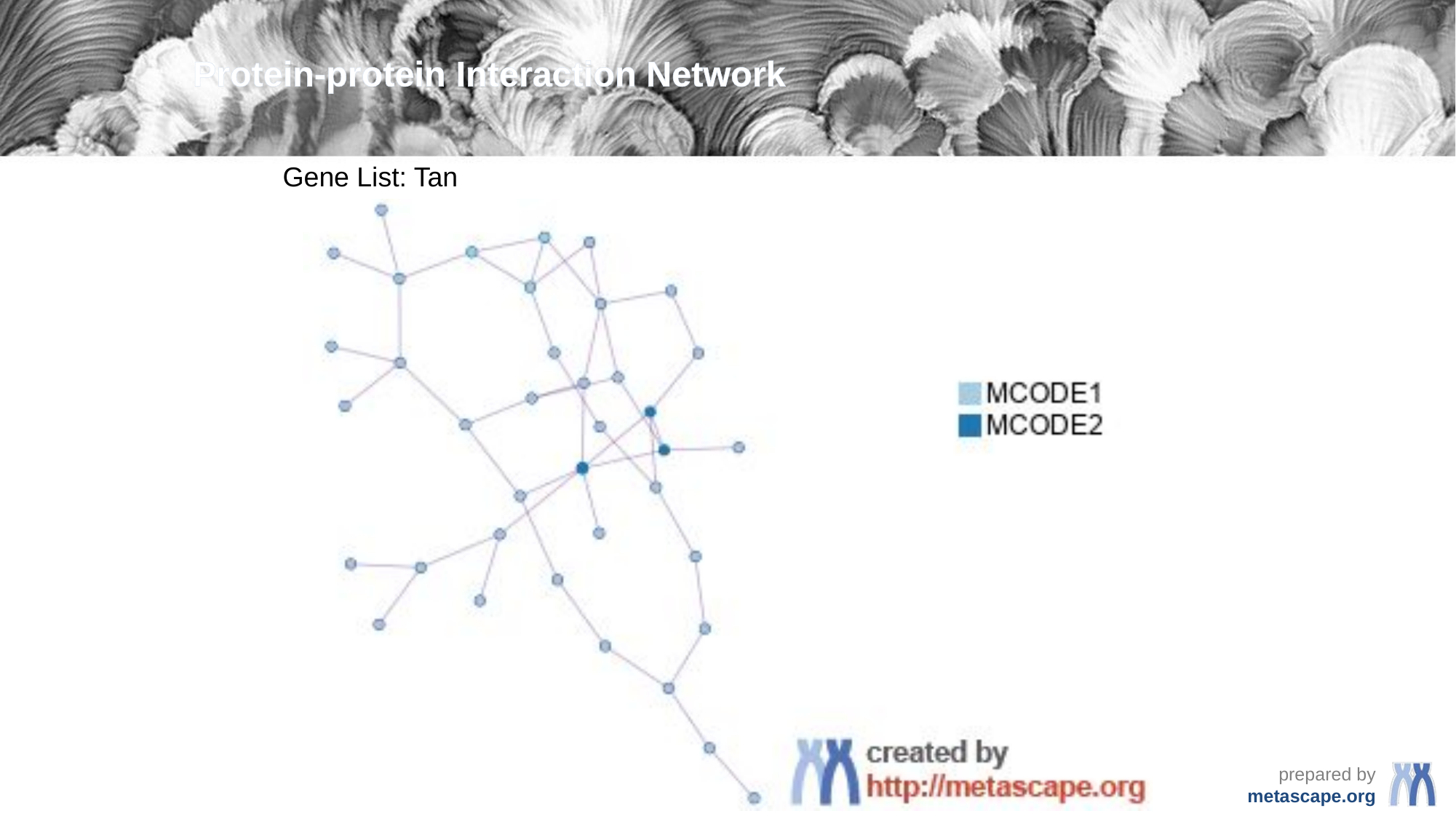

Protein-protein Interaction Network
Gene List: Tan

## Slide 36
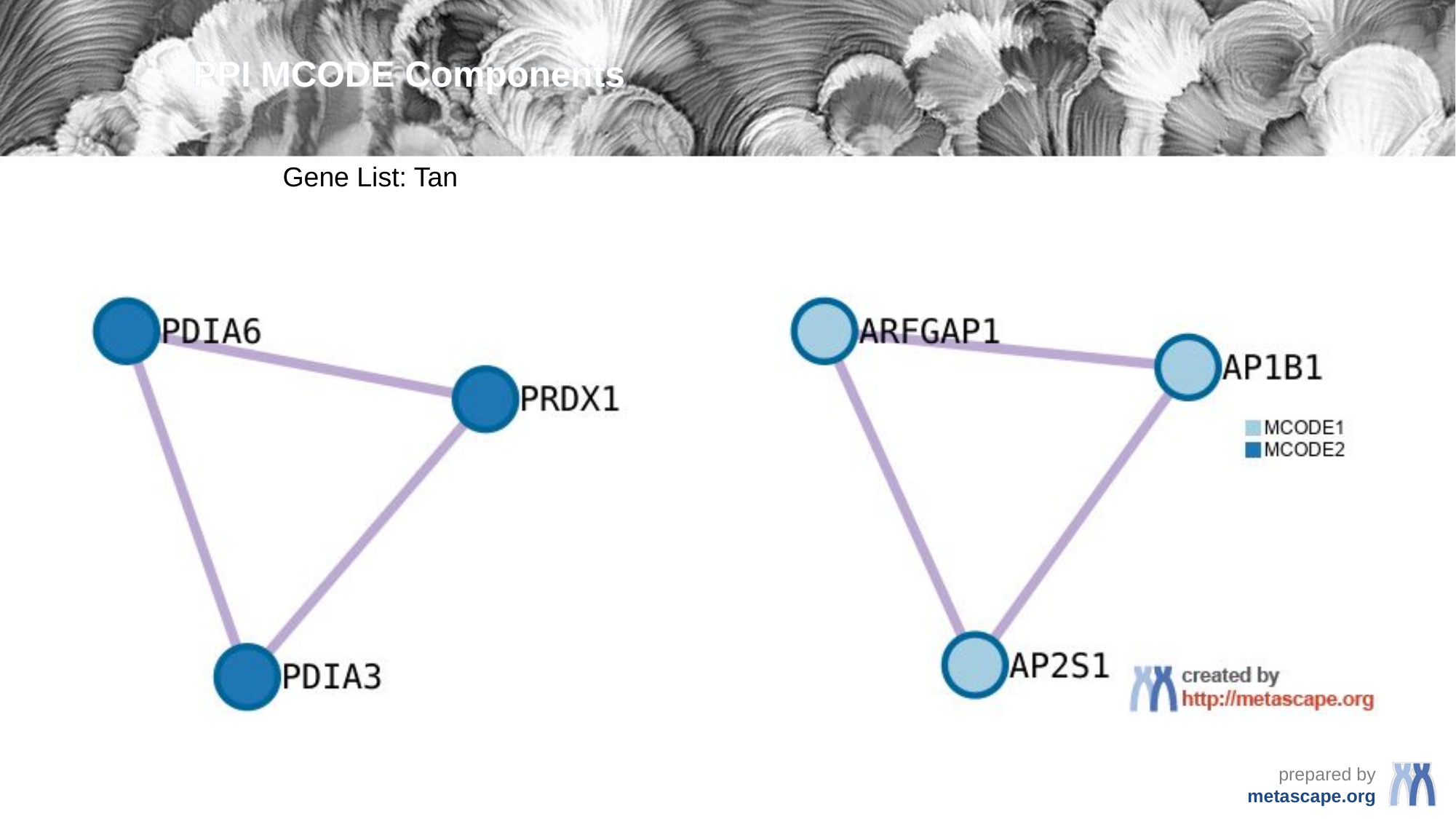

PPI MCODE Components
Gene List: Tan

## Slide 37
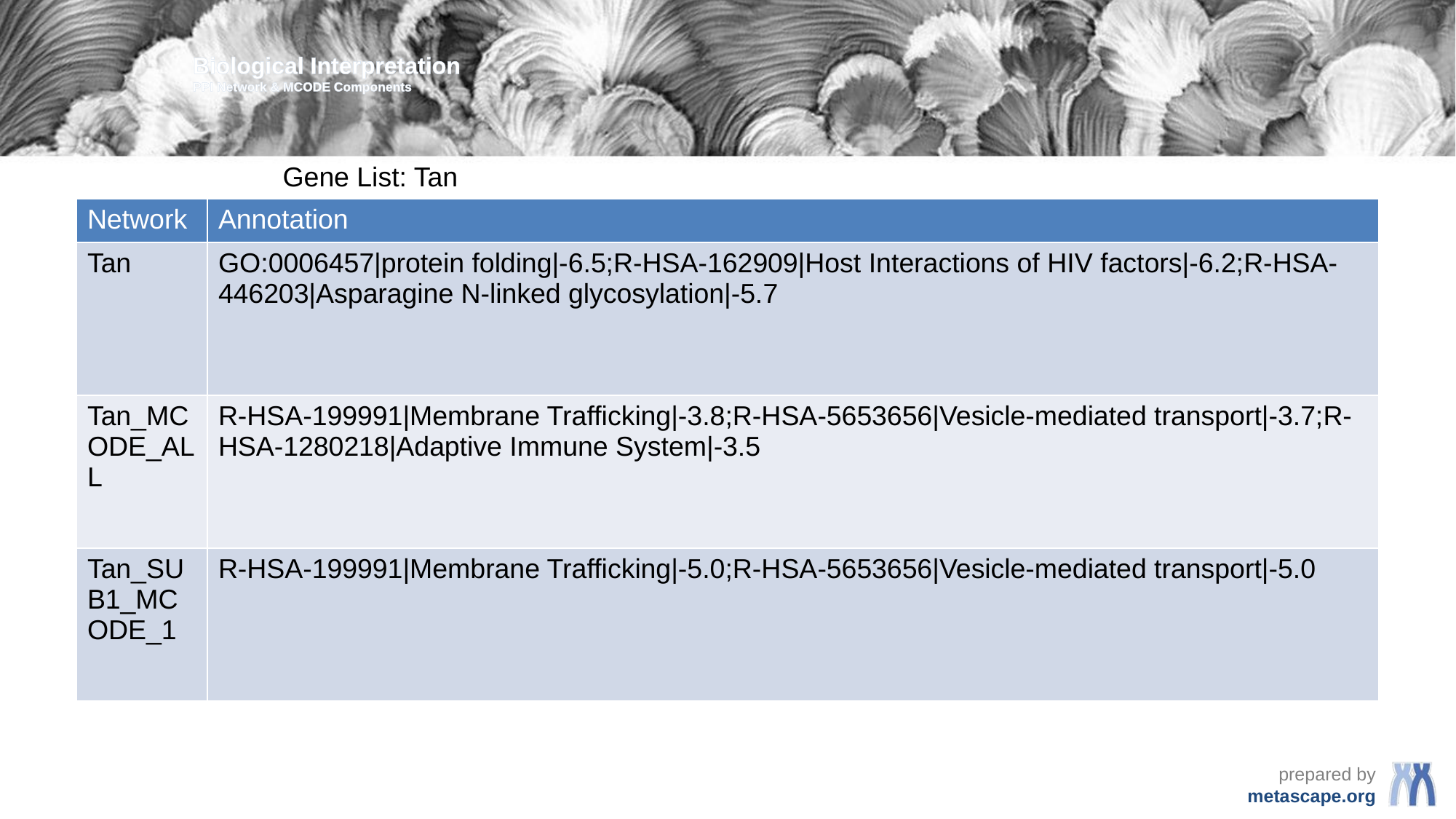

Biological Interpretation
PPI Network & MCODE Components
Gene List: Tan
| Network | Annotation |
| --- | --- |
| Tan | GO:0006457|protein folding|-6.5;R-HSA-162909|Host Interactions of HIV factors|-6.2;R-HSA-446203|Asparagine N-linked glycosylation|-5.7 |
| Tan\_MCODE\_ALL | R-HSA-199991|Membrane Trafficking|-3.8;R-HSA-5653656|Vesicle-mediated transport|-3.7;R-HSA-1280218|Adaptive Immune System|-3.5 |
| Tan\_SUB1\_MCODE\_1 | R-HSA-199991|Membrane Trafficking|-5.0;R-HSA-5653656|Vesicle-mediated transport|-5.0 |

## Slide 38
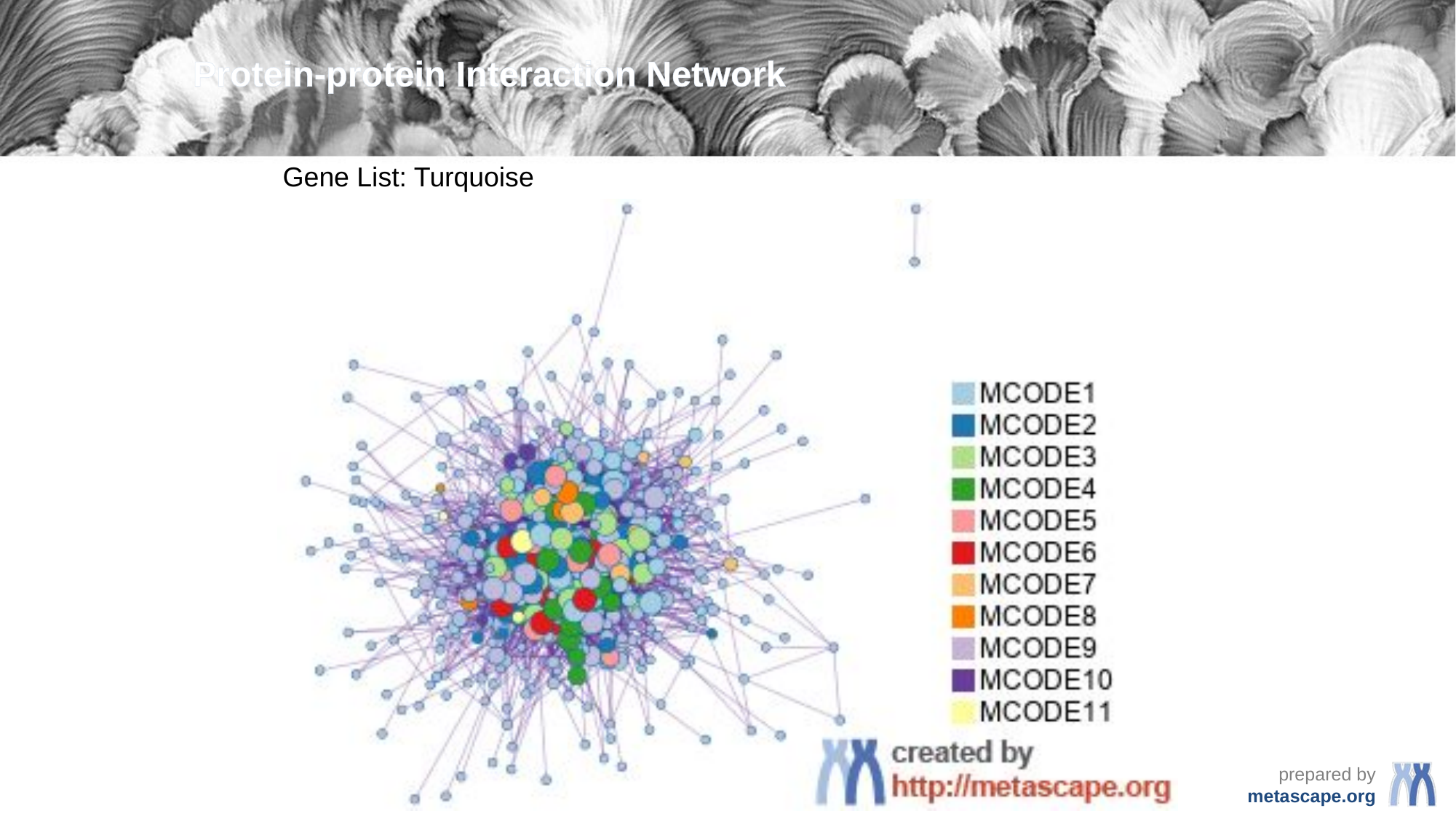

Protein-protein Interaction Network
Gene List: Turquoise

## Slide 39
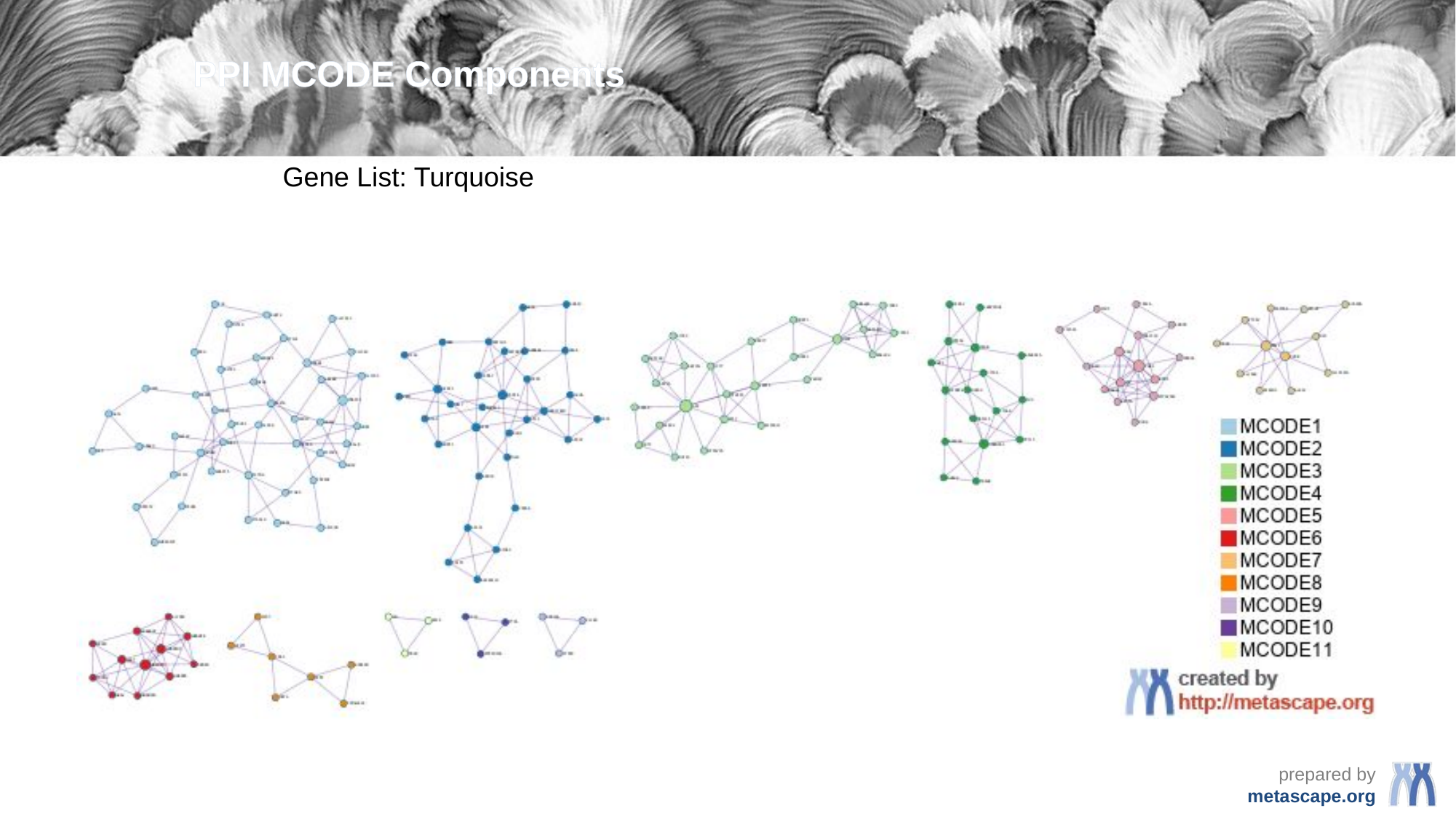

PPI MCODE Components
Gene List: Turquoise

## Slide 40
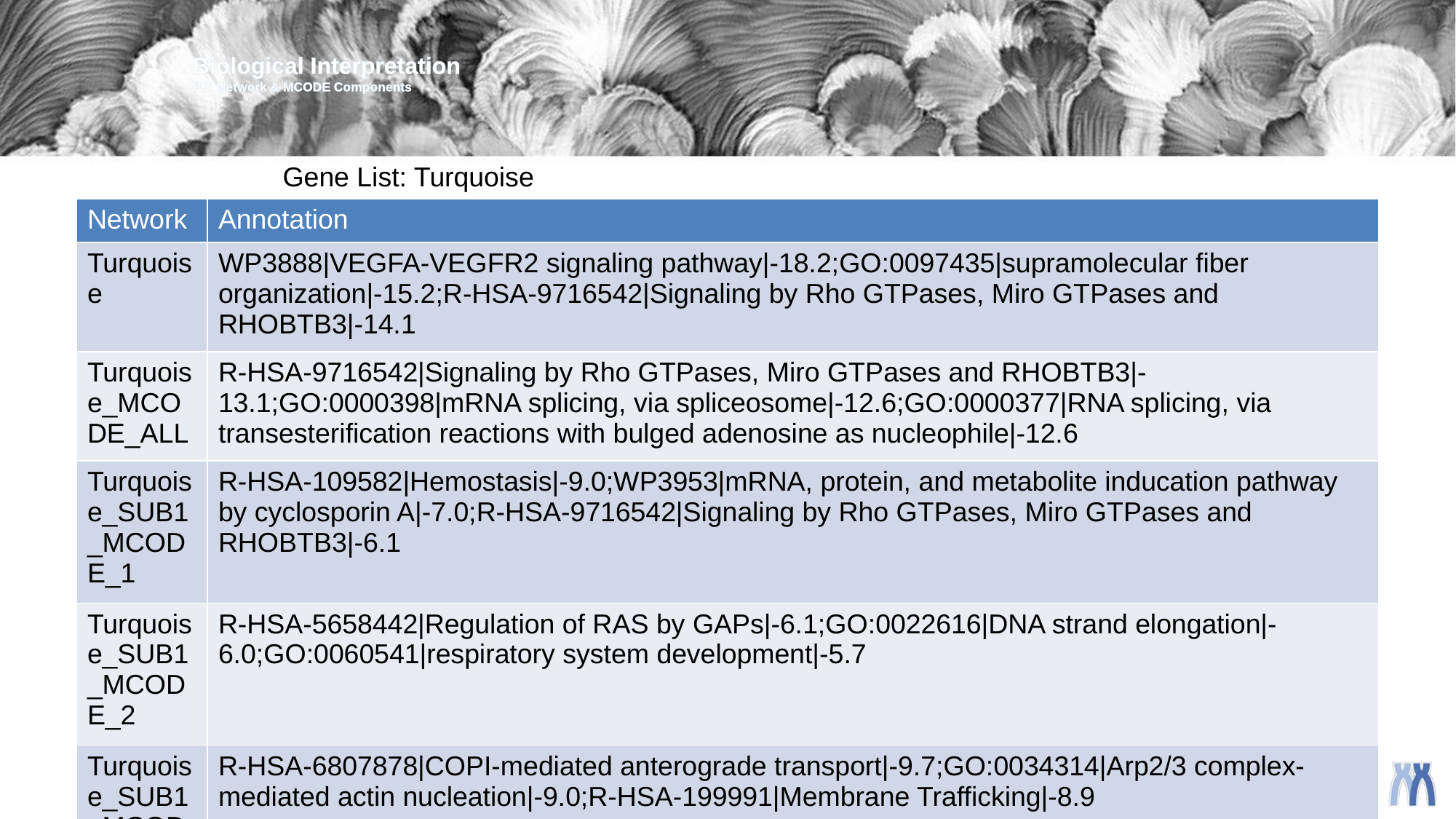

Biological Interpretation
PPI Network & MCODE Components
Gene List: Turquoise
| Network | Annotation |
| --- | --- |
| Turquoise | WP3888|VEGFA-VEGFR2 signaling pathway|-18.2;GO:0097435|supramolecular fiber organization|-15.2;R-HSA-9716542|Signaling by Rho GTPases, Miro GTPases and RHOBTB3|-14.1 |
| Turquoise\_MCODE\_ALL | R-HSA-9716542|Signaling by Rho GTPases, Miro GTPases and RHOBTB3|-13.1;GO:0000398|mRNA splicing, via spliceosome|-12.6;GO:0000377|RNA splicing, via transesterification reactions with bulged adenosine as nucleophile|-12.6 |
| Turquoise\_SUB1\_MCODE\_1 | R-HSA-109582|Hemostasis|-9.0;WP3953|mRNA, protein, and metabolite inducation pathway by cyclosporin A|-7.0;R-HSA-9716542|Signaling by Rho GTPases, Miro GTPases and RHOBTB3|-6.1 |
| Turquoise\_SUB1\_MCODE\_2 | R-HSA-5658442|Regulation of RAS by GAPs|-6.1;GO:0022616|DNA strand elongation|-6.0;GO:0060541|respiratory system development|-5.7 |
| Turquoise\_SUB1\_MCODE\_3 | R-HSA-6807878|COPI-mediated anterograde transport|-9.7;GO:0034314|Arp2/3 complex-mediated actin nucleation|-9.0;R-HSA-199991|Membrane Trafficking|-8.9 |
| Turquoise\_SUB1\_MCODE\_4 | R-HSA-72203|Processing of Capped Intron-Containing Pre-mRNA|-8.1;hsa03040|Spliceosome|-5.8;R-HSA-8953854|Metabolism of RNA|-5.8 |
| Turquoise\_SUB1\_MCODE\_5 | GO:0030433|ubiquitin-dependent ERAD pathway|-5.1;GO:0036503|ERAD pathway|-4.8;GO:0006259|DNA metabolic process|-4.6 |
| Turquoise\_SUB1\_MCODE\_6 | CORUM:1181|C complex spliceosome|-12.6;R-HSA-72163|mRNA Splicing - Major Pathway|-10.1;R-HSA-72172|mRNA Splicing|-10.0 |
| Turquoise\_SUB1\_MCODE\_7 | R-HSA-6798695|Neutrophil degranulation|-6.1;R-HSA-195258|RHO GTPase Effectors|-5.2;WP4540|Hippo signaling regulation pathways|-5.1 |
| Turquoise\_SUB1\_MCODE\_8 | GO:0006260|DNA replication|-9.6;GO:0090329|regulation of DNA-templated DNA replication|-9.4;GO:0006261|DNA-templated DNA replication|-7.9 |
| Turquoise\_SUB1\_MCODE\_9 | R-HSA-4086398|Ca2+ pathway|-8.1;hsa04720|Long-term potentiation|-8.0;hsa04924|Renin secretion|-7.9 |
| Turquoise\_SUB1\_MCODE\_10 | GO:0051345|positive regulation of hydrolase activity|-5.2 |

## Slide 41
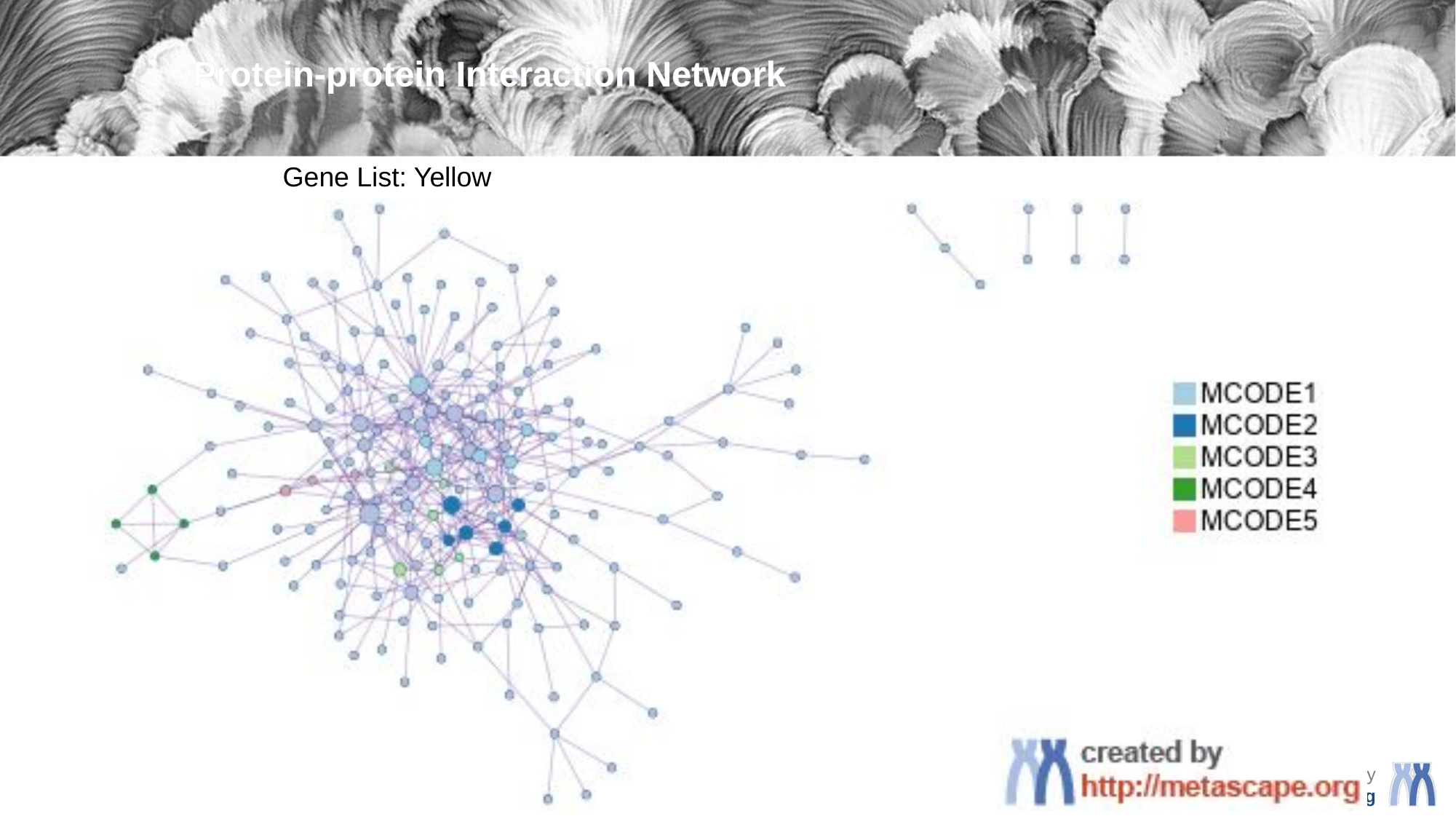

Protein-protein Interaction Network
Gene List: Yellow

## Slide 42
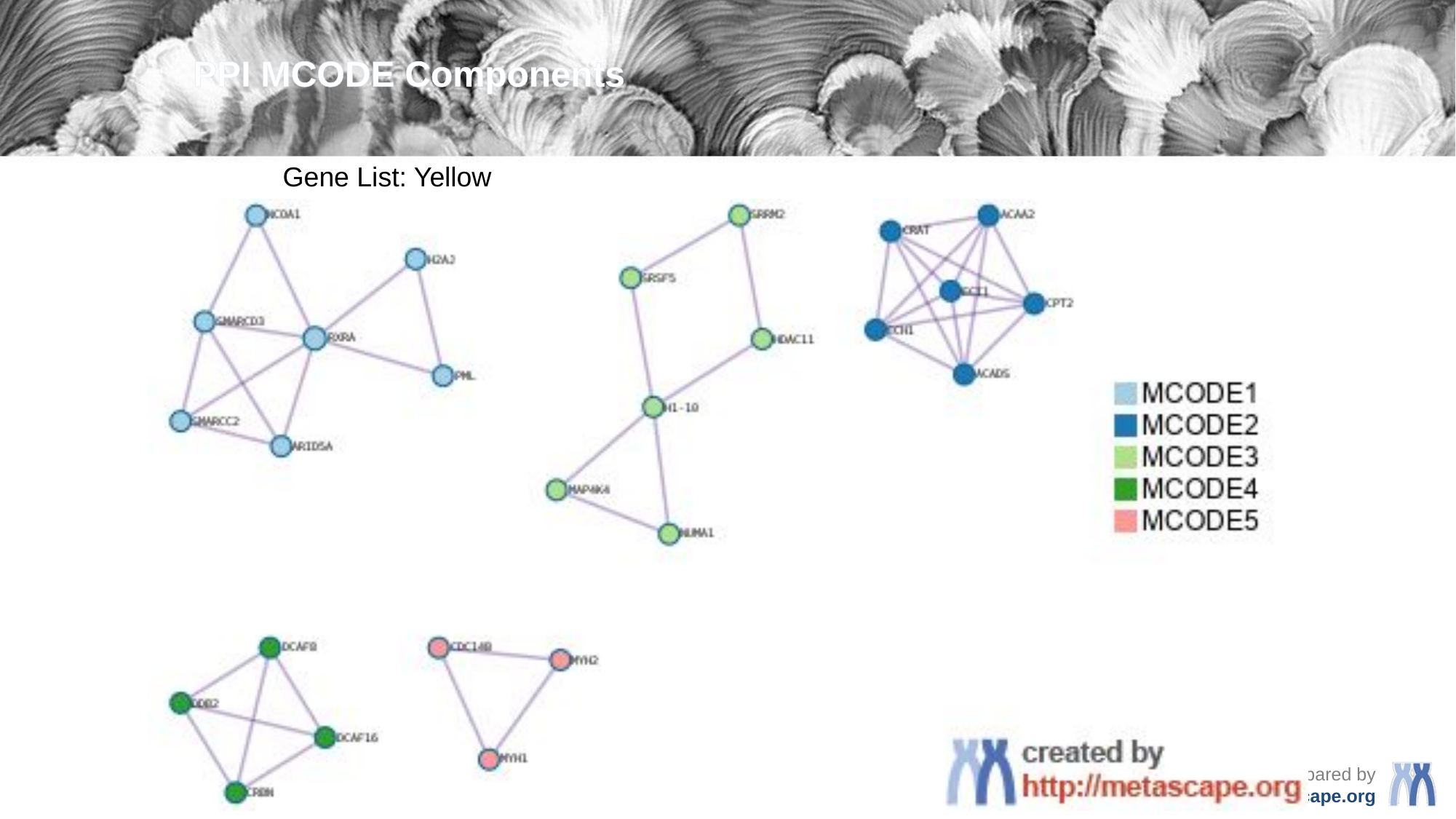

PPI MCODE Components
Gene List: Yellow

## Slide 43
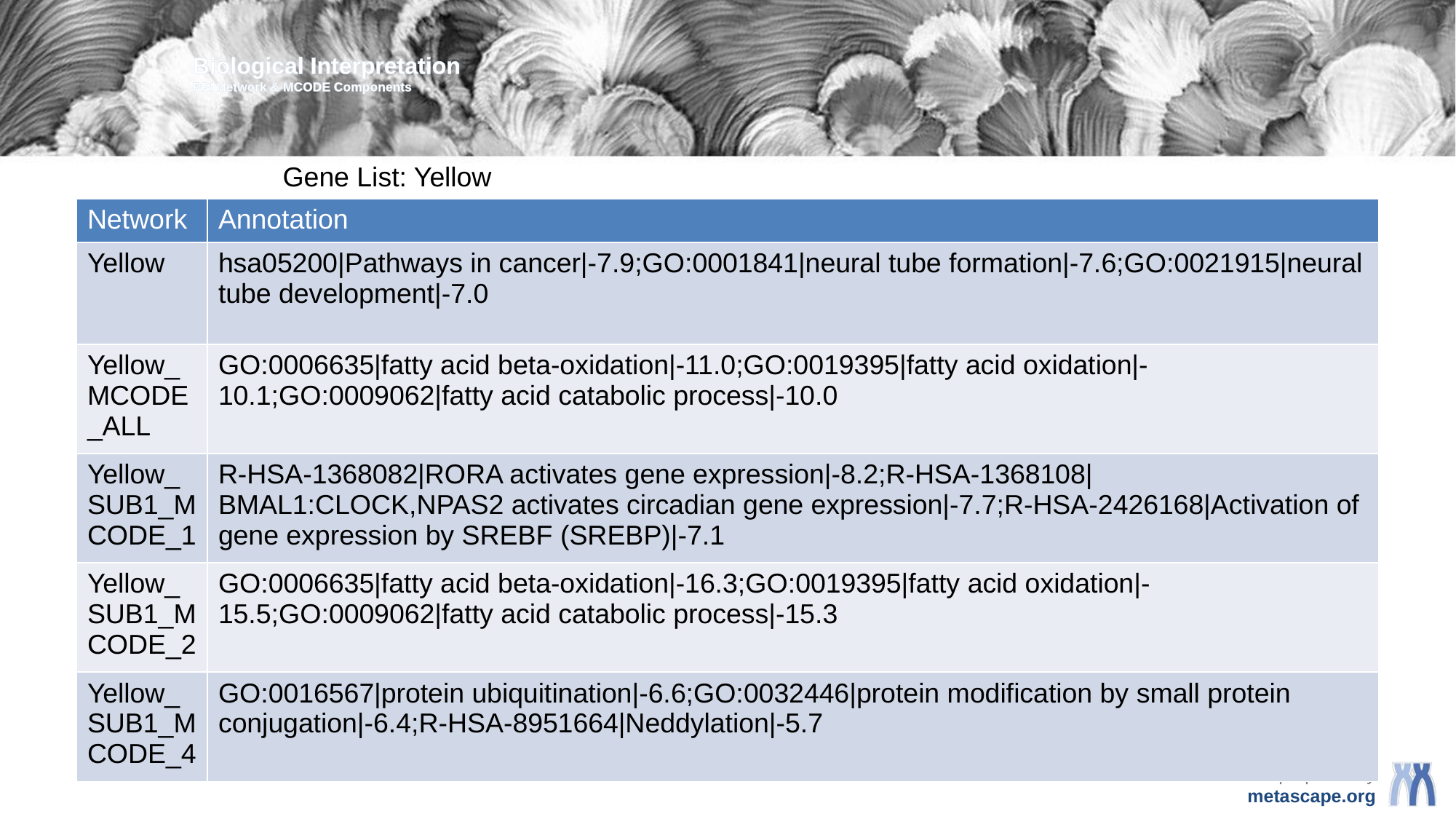

Biological Interpretation
PPI Network & MCODE Components
Gene List: Yellow
| Network | Annotation |
| --- | --- |
| Yellow | hsa05200|Pathways in cancer|-7.9;GO:0001841|neural tube formation|-7.6;GO:0021915|neural tube development|-7.0 |
| Yellow\_MCODE\_ALL | GO:0006635|fatty acid beta-oxidation|-11.0;GO:0019395|fatty acid oxidation|-10.1;GO:0009062|fatty acid catabolic process|-10.0 |
| Yellow\_SUB1\_MCODE\_1 | R-HSA-1368082|RORA activates gene expression|-8.2;R-HSA-1368108|BMAL1:CLOCK,NPAS2 activates circadian gene expression|-7.7;R-HSA-2426168|Activation of gene expression by SREBF (SREBP)|-7.1 |
| Yellow\_SUB1\_MCODE\_2 | GO:0006635|fatty acid beta-oxidation|-16.3;GO:0019395|fatty acid oxidation|-15.5;GO:0009062|fatty acid catabolic process|-15.3 |
| Yellow\_SUB1\_MCODE\_4 | GO:0016567|protein ubiquitination|-6.6;GO:0032446|protein modification by small protein conjugation|-6.4;R-HSA-8951664|Neddylation|-5.7 |

## Slide 44
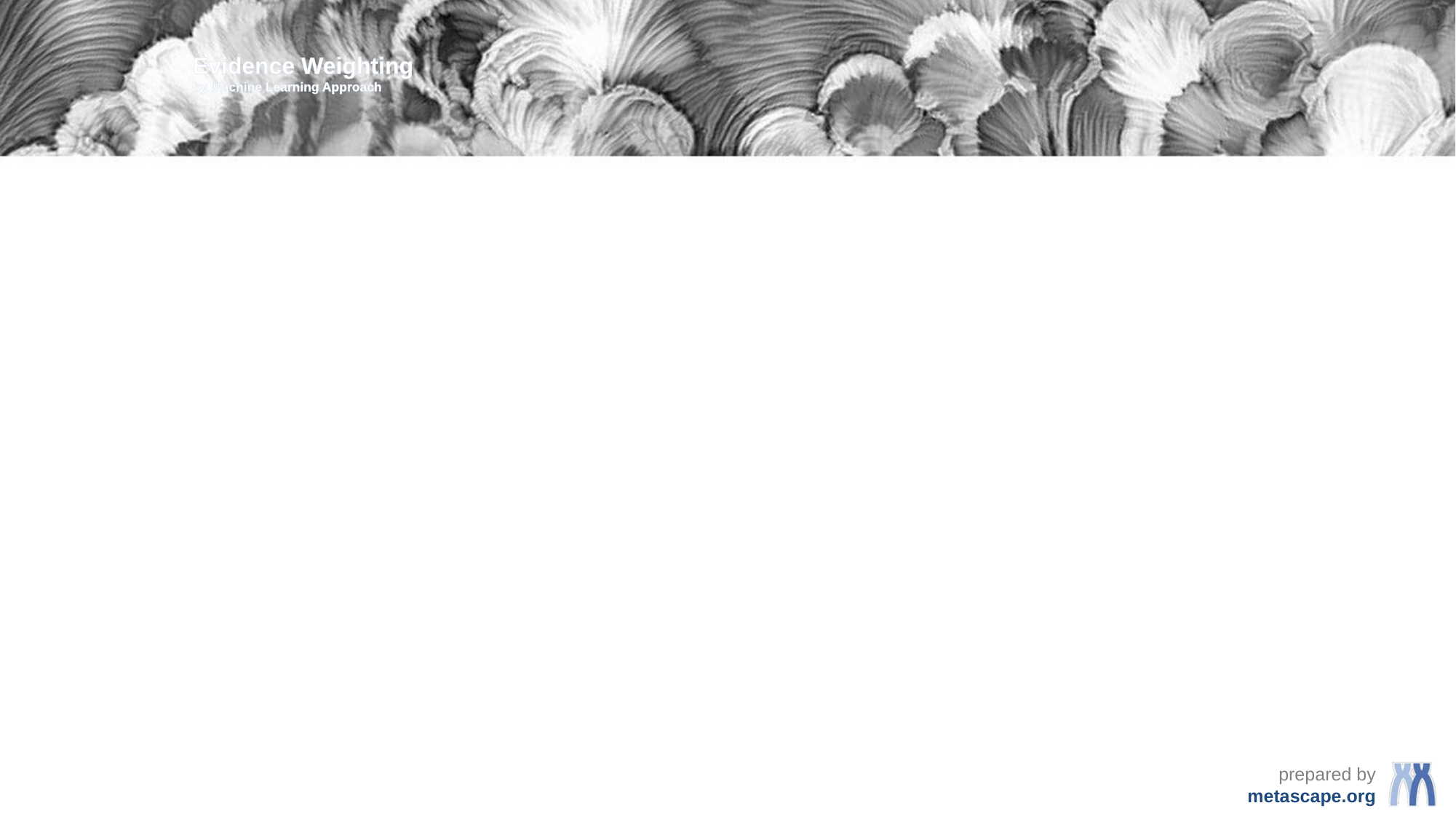

Evidence Weighting
by Machine Learning Approach

## Slide 45
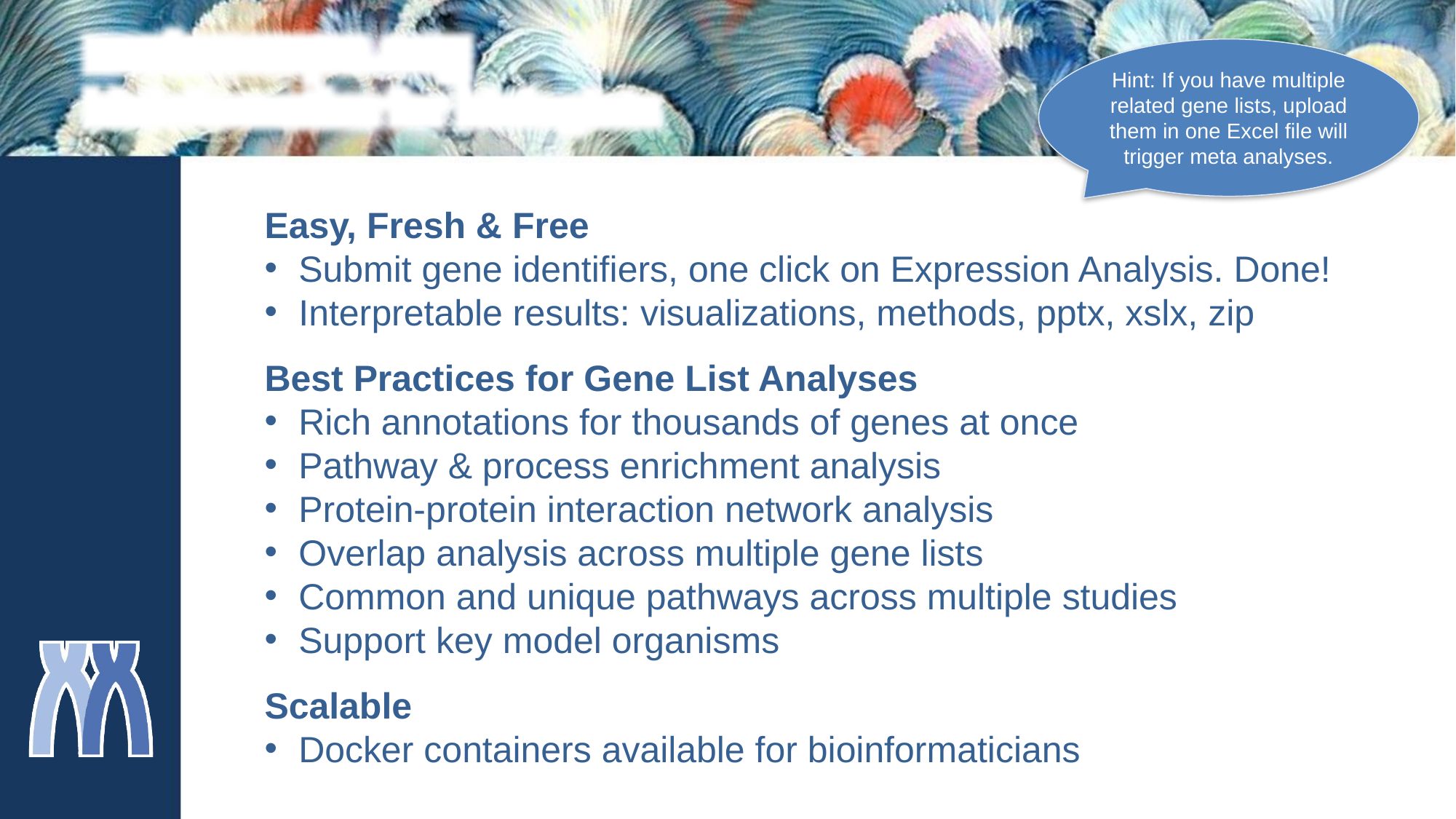

metascape.org
bioinformatics for biologists
Hint: If you have multiple related gene lists, upload them in one Excel file will trigger meta analyses.
Easy, Fresh & Free
Submit gene identifiers, one click on Expression Analysis. Done!
Interpretable results: visualizations, methods, pptx, xslx, zip
Best Practices for Gene List Analyses
Rich annotations for thousands of genes at once
Pathway & process enrichment analysis
Protein-protein interaction network analysis
Overlap analysis across multiple gene lists
Common and unique pathways across multiple studies
Support key model organisms
Scalable
Docker containers available for bioinformaticians
