# Supplementary material for: Fibroblasts as an in vitro model of circadian genetic and genomic studies
Source: Mamm Genome. 2024 Jul 3;35(3):432–44. doi: 10.1007/s00335-024-10050-7 (PMC11329553; doi:10.1007/s00335-024-10050-7)
Supplement: Supplementary file 4 — Supplementary file4 (ZIP 16237 kb) [file 335_2024_10050_MOESM4_ESM.zip › Enrichment_GO/ColorByCluster.pdf]

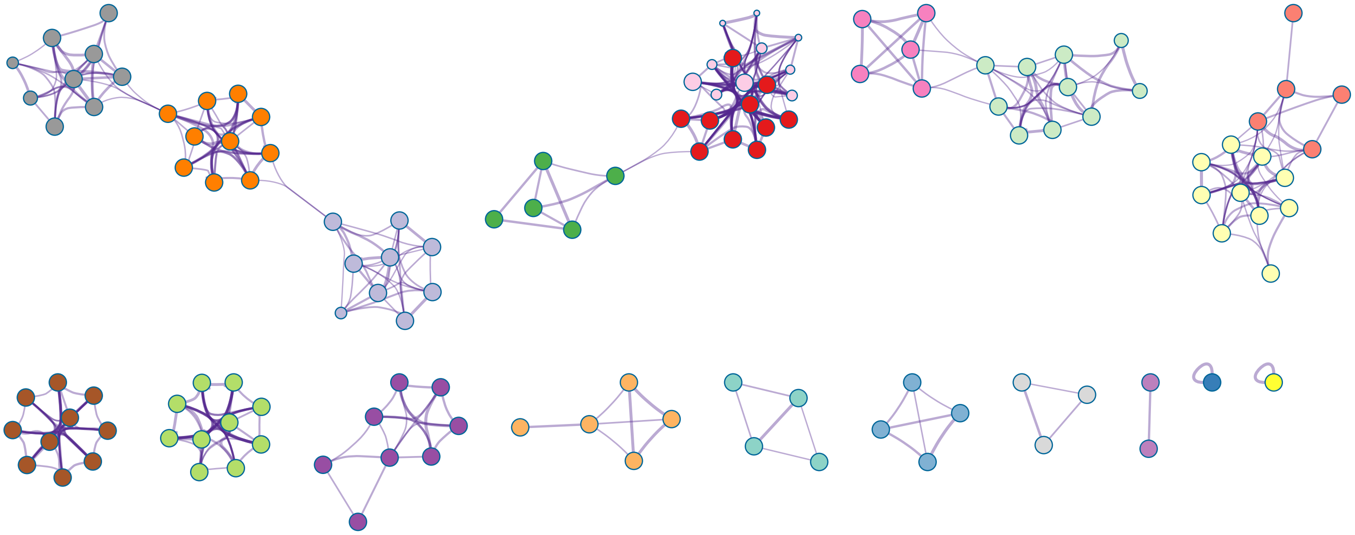

cell division
VEGFA-VEGFR2 signaling pathway
Signaling by Rho GTPases, Miro GTPases and RHOE
intracellular protein transport
Vesicle-mediated transport
Cellular responses to stress
response to hormone
tube morphogenesis
carbohydrate derivative biosynthetic process
Signaling by Receptor Tyrosine Kinases
negative regulation of cellular component organizatic
organelle localization
supramolecular fiber organization
Hemostasis
positive regulation of cell death
Organelle biogenesis and maintenance
positive regulation of cell cycle process
Cytokine Signaling in Immune system
ossification
heart development

created by
http://metascape.org
