# Supplementary material for: Fibroblasts as an in vitro model of circadian genetic and genomic studies
Source: Mamm Genome. 2024 Jul 3;35(3):432–44. doi: 10.1007/s00335-024-10050-7 (PMC11329553; doi:10.1007/s00335-024-10050-7)
Supplement: Supplementary file 4 — Supplementary file4 (ZIP 16237 kb) [file 335_2024_10050_MOESM4_ESM.zip › Enrichment_GO/ColorByCounts.pdf]

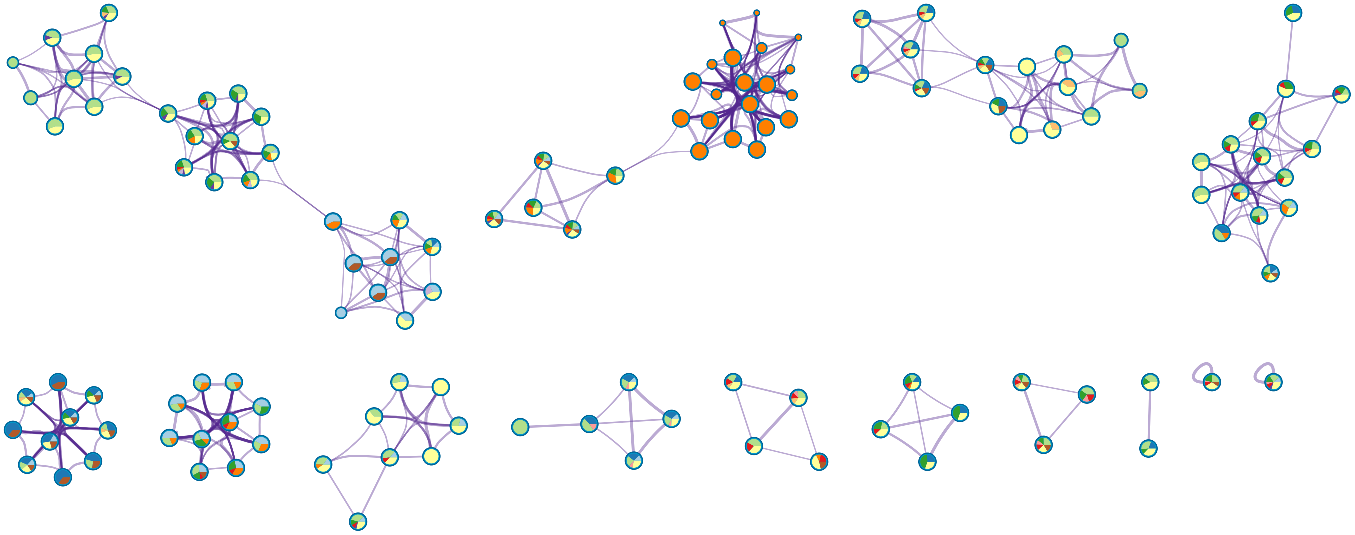

Black
Blue
Brown
Green
Greenyellow
Magenta
Pink
Purple
Red
Tan
Turquoise
Yellow

created by
http://metascape.org
