# Supplementary material for: Fibroblasts as an in vitro model of circadian genetic and genomic studies
Source: Mamm Genome. 2024 Jul 3;35(3):432–44. doi: 10.1007/s00335-024-10050-7 (PMC11329553; doi:10.1007/s00335-024-10050-7)
Supplement: Supplementary file 4 — Supplementary file4 (ZIP 16237 kb) [file 335_2024_10050_MOESM4_ESM.zip › AnalysisReport.html]

## Metascape Gene List Analysis Report

metascape.org1

### Heatmap Summary

Figure 1. Heatmap of enriched terms across input gene lists, colored by p-values.

|  |
| --- |
|  |
|  |
| Metascape only visualizes the top 20 clusters. Up to 100 enriched clusters can be viewed here. |
| The top-level Gene Ontology biological processes can be viewed here. |

The heatmap can be interactively viewed using JTreeView2 (.cdt, .gtr and .atr files can be found in the Zip package).

### Gene Lists

User-provided gene identifiers are first converted into their corresponding H. sapiens Entrez gene IDs using the latest version of the database (last updated on 2023-01-01). If multiple identifiers correspond to the same Entrez gene ID, they will be considered as a single Entrez gene ID in downstream analyses. Each gene list is assigned a unique color, which is used throughout the analysis. The gene lists are summarized in Table 1.

Table 1. Statistics of input gene lists.

| Name | Total | Unique | Color Code |
| --- | --- | --- | --- |
| Black | 207 | 207 |  |
| Blue | 396 | 396 |  |
| Brown | 381 | 381 |  |
| Green | 248 | 247 |  |
| Greenyellow | 77 | 77 |  |
| Magenta | 128 | 127 |  |
| Pink | 161 | 161 |  |
| Purple | 81 | 81 |  |
| Red | 216 | 215 |  |
| Tan | 70 | 70 |  |
| Turquoise | 505 | 503 |  |
| Yellow | 297 | 296 |  |

The overlaps between these lists are shown in a Circos3 plot (Figure 2.a). Another useful representation is to overlap genes based on their functions or shared pathways. The overlaps between gene lists can be significantly improved by considering overlaps between genes sharing the same enriched ontology term(s) (Figure 2.b). Only ontology terms that contain less than 100 genes are used to calculate functional overlaps to avoid linking genes using very general annotation. (We do not want to link all genes, only genes that belong to specific biological processes.)

Figure 2. Overlap between gene lists: (a) only at the gene level, where purple curves link identical genes; (b) including the shared term level, where blue curves link genes that belong to the same enriched ontology term. The inner circle represents gene lists, where hits are arranged along the arc. Genes that hit multiple lists are colored in dark orange, and genes unique to a list are shown in light orange. The publication-quality version of the figures is included in the Zip package as a .svg file under the Overlap\_circos folder (readable by popular web browsers and Adobe Illustrator).

|  |  |
| --- | --- |
|  |  |
|  |  |

### Gene Annotation

The following are the list of annotations retrieved from the latest version of the database (last updated on 2023-01-01) (Table 2).

Table 2. Gene annotations extracted

| Name | Type | Description |
| --- | --- | --- |
| Gene Symbol | Description | Primary HUGO gene symbol. |
| Description | Description | Short description. |
| Biological Process (GO) | Function/Location | Descriptions summarized based on gene ontology database, where up to three most informative GO terms are kept. |
| Kinase Class (UniProt) | Function/Location | Detailed kinase classes. |
| Protein Function (Protein Atlas) | Function/Location | Protein Function (Protein Atlas) |
| Subcellular Location (Protein Atlas) | Function/Location | Subcellular Location (Protein Atlas) |
| Drug (DrugBank) | Genotype/Phenotype/Disease | Drug information for the given gene as target. |
| Canonical Pathways | Ontology | Canonical Pathways |
| Hallmark Gene Sets | Ontology | Hallmark Gene Sets |

### Pathway and Process Enrichment Analysis

For each given gene list, pathway and process enrichment analysis have been carried out with the following ontology sources: KEGG Pathway, GO Biological Processes, Reactome Gene Sets, Canonical Pathways, CORUM, WikiPathways, and PANTHER Pathway. All genes in the genome have been used as the enrichment background. Terms with a p-value < 0.01, a minimum count of 3, and an enrichment factor > 1.5 (the enrichment factor is the ratio between the observed counts and the counts expected by chance) are collected and grouped into clusters based on their membership similarities. More specifically, p-values are calculated based on the cumulative hypergeometric distribution4, and q-values are calculated using the Benjamini-Hochberg procedure to account for multiple testings5. Kappa scores6 are used as the similarity metric when performing hierarchical clustering on the enriched terms, and sub-trees with a similarity of > 0.3 are considered a cluster. The most statistically significant term within a cluster is chosen to represent the cluster.When multiple gene lists are provided, all lists are merged into one list called "\_FINAL". A term may be found enriched in several individual gene lists and/or in the \_FINAL gene list, and the best p-value among them is chosen as the final p-value. The pathway/process clusters that are found to be of interest (either shared or unique based on specific list enrichment) are used to prioritize the genes that fall into those clusters (membership is presented as 1/0 binary columns in the Excel spreadsheet). Note that individual gene lists containing more than 3000 genes are ignored during the enrichment analysis to avoid superficial terms; this is because long gene lists are often not random and generally trigger too many terms that are not of direct relevance to the biology under study.

Table 3. Top 20 clusters with their representative enriched terms (one per cluster). "Count" is the number of genes in the user-provided lists with membership in the given ontology term. "%" is the percentage of all of the user-provided genes that are found in the given ontology term (only input genes with at least one ontology term annotation are included in the calculation). "Log10(P)" is the p-value in log base 10. "Log10(q)" is the multi-test adjusted p-value in log base 10. \_\_PATTERN\_\_ shows the color code used for the gene lists where the term is found statistically significant, i.e., multiple colors indicate a pathway/process that is shared across multiple lists.

| \_PATTERN\_ | GO | Category | Description | Count | % | Log10(P) | Log10(q) |
| --- | --- | --- | --- | --- | --- | --- | --- |
|  | GO:0051301 | GO Biological Processes | cell division | 50 | 61.73 | -67.55 | -63.20 |
|  | WP3888 | WikiPathways | VEGFA-VEGFR2 signaling pathway | 151 | 5.48 | -49.64 | -45.29 |
|  | R-HSA-9716542 | Reactome Gene Sets | Signaling by Rho GTPases, Miro GTPases and RHOBTB3 | 198 | 7.18 | -46.22 | -42.35 |
|  | GO:0006886 | GO Biological Processes | intracellular protein transport | 174 | 6.31 | -34.99 | -31.55 |
|  | R-HSA-5653656 | Reactome Gene Sets | Vesicle-mediated transport | 168 | 6.10 | -33.70 | -30.35 |
|  | R-HSA-2262752 | Reactome Gene Sets | Cellular responses to stress | 178 | 6.46 | -29.78 | -26.51 |
|  | GO:0009725 | GO Biological Processes | response to hormone | 174 | 6.31 | -28.22 | -25.04 |
|  | GO:0035239 | GO Biological Processes | tube morphogenesis | 155 | 5.62 | -27.19 | -24.08 |
|  | GO:1901137 | GO Biological Processes | carbohydrate derivative biosynthetic process | 141 | 5.12 | -26.26 | -23.23 |
|  | R-HSA-9006934 | Reactome Gene Sets | Signaling by Receptor Tyrosine Kinases | 130 | 4.72 | -25.86 | -22.85 |
|  | GO:0051129 | GO Biological Processes | negative regulation of cellular component organization | 156 | 5.66 | -25.31 | -22.36 |
|  | GO:0051640 | GO Biological Processes | organelle localization | 127 | 4.61 | -24.69 | -21.77 |
|  | GO:0097435 | GO Biological Processes | supramolecular fiber organization | 131 | 4.75 | -23.66 | -20.80 |
|  | GO:0010942 | GO Biological Processes | positive regulation of cell death | 140 | 5.08 | -23.44 | -20.61 |
|  | R-HSA-109582 | Reactome Gene Sets | Hemostasis | 140 | 5.08 | -23.44 | -20.61 |
|  | R-HSA-1852241 | Reactome Gene Sets | Organelle biogenesis and maintenance | 88 | 3.19 | -23.40 | -20.58 |
|  | GO:0090068 | GO Biological Processes | positive regulation of cell cycle process | 20 | 24.69 | -23.08 | -20.32 |
|  | R-HSA-1280215 | Reactome Gene Sets | Cytokine Signaling in Immune system | 155 | 5.62 | -23.05 | -20.25 |
|  | GO:0001503 | GO Biological Processes | ossification | 84 | 3.05 | -22.88 | -20.09 |
|  | GO:0007507 | GO Biological Processes | heart development | 130 | 4.72 | -22.86 | -20.08 |

To further capture the relationships between the terms, a subset of enriched terms has been selected and rendered as a network plot, where terms with a similarity > 0.3 are connected by edges. We select the terms with the best p-values from each of the 20 clusters, with the constraint that there are no more than 15 terms per cluster and no more than 250 terms in total. The network is visualized using Cytoscape7, where each node represents an enriched term and is colored first by its cluster ID (Figure 3.a) and then by its p-value (Figure 3.b). These networks can be interactively viewed in Cytoscape through the .cys files (contained in the Zip package, which also contains a publication-quality version as a PDF) or within a browser by clicking on the web icon. For clarity, term labels are only shown for one term per cluster, so it is recommended to use Cytoscape or a browser to visualize the network in order to inspect all node labels. We can also export the network into a PDF file within Cytoscape, and then edit the labels using Adobe Illustrator for publication purposes. To switch off all labels, delete the "Label" mapping under the "Style" tab within Cytoscape, and then export the network view.

Figure 3. Network of enriched terms: (a) colored by cluster ID, where nodes that share the same cluster ID are typically close to each other; (b) colored by p-value, where terms containing more genes tend to have a more significant p-value.

|  |  |
| --- | --- |
|  |  |
|  |  |

In the case of when multiple gene lists are provided, the nodes are represented as pie charts, where the size of a pie is proportional to the total number of hits that fall into that specific term. The pie charts are color-coded based on the gene list identities, where the size of a slice represents the percentage of genes under the term that originated from the corresponding gene list. This plot is particularly useful for visualizing whether the terms are shared by multiple lists or unique to a specific list, as well as for understanding how these terms associate with each other within the biological context of the meta study (Figure 4).

Figure 4. Network of enriched terms represented as pie charts, where pies are color-coded based on the identities of the gene lists.

|  |
| --- |
|  |
|  |

### Protein-protein Interaction Enrichment Analysis

For each given gene list, protein-protein interaction enrichment analysis has been carried out with the following databases: STRING8, BioGrid9, OmniPath10, InWeb\_IM11.Only physical interactions in STRING (physical score > 0.132) and BioGrid are used (details). The resultant network contains the subset of proteins that form physical interactions with at least one other member in the list. If the network contains between 3 and 500 proteins, the Molecular Complex Detection (MCODE) algorithm12 has been applied to identify densely connected network components. The MCODE networks identified for individual gene lists have been gathered and are shown in Figure 5.Pathway and process enrichment analysis has been applied to each MCODE component independently, and the three best-scoring terms by p-value have been retained as the functional description of the corresponding components, shown in the tables underneath corresponding network plots within Figure 5.

Figure 5. Protein-protein interaction network and MCODE components identified in the gene lists.

|  |  |  |
| --- | --- | --- |
|  |  |  |
|  |  |  |
| Black (Full Connection)  | GO | Description | Log10(P) | | --- | --- | --- | | R-HSA-5617833 | Cilium Assembly | -12.3 | | R-HSA-1852241 | Organelle biogenesis and maintenance | -12.0 | | GO:0006325 | chromatin organization | -12.0 | |  | Black (Keep MCODE Nodes Only)  | Color | MCODE | GO | Description | Log10(P) | | --- | --- | --- | --- | --- | |  | MCODE\_1 | R-HSA-1852241 | Organelle biogenesis and maintenance | -10.2 | |  | MCODE\_1 | R-HSA-5617833 | Cilium Assembly | -8.8 | |  | MCODE\_1 | R-HSA-380259 | Loss of Nlp from mitotic centrosomes | -8.5 | |  | MCODE\_2 | GO:0006368 | transcription elongation by RNA polymerase II | -7.1 | |  | MCODE\_2 | GO:0006354 | DNA-templated transcription elongation | -6.9 | |  | MCODE\_2 | R-HSA-75955 | RNA Polymerase II Transcription Elongation | -6.4 | |  | MCODE\_4 | GO:0006338 | chromatin remodeling | -5.7 | |  | MCODE\_4 | GO:0006325 | chromatin organization | -5.0 | |

|  |  |  |
| --- | --- | --- |
|  |  |  |
|  |  |  |
| Blue (Full Connection)  | GO | Description | Log10(P) | | --- | --- | --- | | GO:0009725 | response to hormone | -14.1 | | GO:0032870 | cellular response to hormone stimulus | -8.9 | | GO:0030097 | hemopoiesis | -8.7 | |  | Blue (Keep MCODE Nodes Only)  | Color | MCODE | GO | Description | Log10(P) | | --- | --- | --- | --- | --- | |  | MCODE\_1 | hsa05171 | Coronavirus disease - COVID-19 | -8.5 | |  | MCODE\_1 | R-HSA-909733 | Interferon alpha/beta signaling | -8.4 | |  | MCODE\_1 | GO:0045087 | innate immune response | -7.8 | |  | MCODE\_2 | hsa04014 | Ras signaling pathway | -12.7 | |  | MCODE\_2 | hsa04010 | MAPK signaling pathway | -12.1 | |  | MCODE\_2 | WP3932 | Focal adhesion: PI3K-Akt-mTOR-signaling pathway | -12.0 | |  | MCODE\_4 | M223 | PID BETA CATENIN NUC PATHWAY | -7.1 | |  | MCODE\_4 | GO:0198738 | cell-cell signaling by wnt | -5.5 | |  | MCODE\_4 | GO:0016055 | Wnt signaling pathway | -5.5 | |  | MCODE\_5 | R-HSA-432722 | Golgi Associated Vesicle Biogenesis | -8.2 | |  | MCODE\_5 | R-HSA-199992 | trans-Golgi Network Vesicle Budding | -7.9 | |  | MCODE\_5 | R-HSA-199991 | Membrane Trafficking | -5.0 | |  | MCODE\_6 | M181 | PID BMP PATHWAY | -8.6 | |  | MCODE\_6 | GO:0030509 | BMP signaling pathway | -7.6 | |  | MCODE\_6 | hsa04350 | TGF-beta signaling pathway | -7.5 | |  | MCODE\_9 | hsa02010 | ABC transporters | -8.5 | |  | MCODE\_9 | R-HSA-382556 | ABC-family proteins mediated transport | -7.4 | |  | MCODE\_9 | GO:0006869 | lipid transport | -5.8 | |

|  |  |  |
| --- | --- | --- |
|  |  |  |
|  |  |  |
| Brown (Full Connection)  | GO | Description | Log10(P) | | --- | --- | --- | | GO:0006886 | intracellular protein transport | -18.1 | | R-HSA-72163 | mRNA Splicing - Major Pathway | -17.4 | | hsa04141 | Protein processing in endoplasmic reticulum | -17.1 | |  | Brown (Keep MCODE Nodes Only)  | Color | MCODE | GO | Description | Log10(P) | | --- | --- | --- | --- | --- | |  | MCODE\_1 | R-HSA-72163 | mRNA Splicing - Major Pathway | -45.7 | |  | MCODE\_1 | R-HSA-72172 | mRNA Splicing | -45.3 | |  | MCODE\_1 | R-HSA-72203 | Processing of Capped Intron-Containing Pre-mRNA | -42.5 | |  | MCODE\_2 | R-HSA-1428517 | The citric acid (TCA) cycle and respiratory electron transport | -6.7 | |  | MCODE\_2 | R-HSA-1592230 | Mitochondrial biogenesis | -6.1 | |  | MCODE\_2 | GO:0051276 | chromosome organization | -5.9 | |  | MCODE\_3 | GO:0006457 | protein folding | -8.5 | |  | MCODE\_3 | GO:0061077 | chaperone-mediated protein folding | -7.1 | |  | MCODE\_3 | GO:0050821 | protein stabilization | -6.7 | |  | MCODE\_4 | GO:0018279 | protein N-linked glycosylation via asparagine | -6.4 | |  | MCODE\_4 | GO:1902600 | proton transmembrane transport | -6.1 | |  | MCODE\_4 | hsa05014 | Amyotrophic lateral sclerosis | -5.7 | |  | MCODE\_5 | R-HSA-6811434 | COPI-dependent Golgi-to-ER retrograde traffic | -20.8 | |  | MCODE\_5 | R-HSA-6807878 | COPI-mediated anterograde transport | -20.7 | |  | MCODE\_5 | R-HSA-8856688 | Golgi-to-ER retrograde transport | -19.6 | |  | MCODE\_6 | GO:0045047 | protein targeting to ER | -6.3 | |  | MCODE\_6 | R-HSA-381038 | XBP1(S) activates chaperone genes | -6.3 | |  | MCODE\_6 | R-HSA-381070 | IRE1alpha activates chaperones | -6.2 | |  | MCODE\_7 | GO:0033365 | protein localization to organelle | -5.8 | |  | MCODE\_7 | GO:0072594 | establishment of protein localization to organelle | -4.8 | |  | MCODE\_7 | GO:0051668 | localization within membrane | -4.2 | |

|  |  |  |
| --- | --- | --- |
|  |  |  |
|  |  |  |
| Green (Full Connection)  | GO | Description | Log10(P) | | --- | --- | --- | | WP3888 | VEGFA-VEGFR2 signaling pathway | -14.9 | | R-HSA-109582 | Hemostasis | -11.4 | | GO:0030036 | actin cytoskeleton organization | -10.3 | |  | Green (Keep MCODE Nodes Only)  | Color | MCODE | GO | Description | Log10(P) | | --- | --- | --- | --- | --- | |  | MCODE\_1 | R-HSA-446203 | Asparagine N-linked glycosylation | -11.9 | |  | MCODE\_1 | R-HSA-6807878 | COPI-mediated anterograde transport | -11.0 | |  | MCODE\_1 | R-HSA-199977 | ER to Golgi Anterograde Transport | -9.9 | |  | MCODE\_2 | GO:0016072 | rRNA metabolic process | -7.9 | |  | MCODE\_2 | R-HSA-8868773 | rRNA processing in the nucleus and cytosol | -6.3 | |  | MCODE\_2 | GO:0034660 | ncRNA metabolic process | -6.2 | |  | MCODE\_3 | R-HSA-389960 | Formation of tubulin folding intermediates by CCT/TriC | -17.3 | |  | MCODE\_3 | R-HSA-389958 | Cooperation of Prefoldin and TriC/CCT in actin and tubulin folding | -16.6 | |  | MCODE\_3 | R-HSA-190828 | Gap junction trafficking | -15.5 | |  | MCODE\_6 | GO:0031589 | cell-substrate adhesion | -6.7 | |  | MCODE\_6 | WP306 | Focal adhesion | -6.6 | |  | MCODE\_6 | hsa04510 | Focal adhesion | -6.5 | |  | MCODE\_7 | R-HSA-8934593 | Regulation of RUNX1 Expression and Activity | -9.8 | |  | MCODE\_7 | R-HSA-8878171 | Transcriptional regulation by RUNX1 | -6.3 | |

|  |
| --- |
|  |
|  |
| Greenyellow (Full Connection)  | GO | Description | Log10(P) | | --- | --- | --- | | WP2374 | Oncostatin M signaling pathway | -9.5 | | R-HSA-1280215 | Cytokine Signaling in Immune system | -8.6 | | GO:0071345 | cellular response to cytokine stimulus | -7.5 | |

|  |  |  |
| --- | --- | --- |
|  |  |  |
|  |  |  |
| Magenta (Full Connection)  | GO | Description | Log10(P) | | --- | --- | --- | | R-HSA-5663202 | Diseases of signal transduction by growth factor receptors and second messengers | -11.8 | | WP185 | Integrin-mediated cell adhesion | -11.6 | | R-HSA-1280218 | Adaptive Immune System | -10.9 | |  | Magenta (Keep MCODE Nodes Only)  | Color | MCODE | GO | Description | Log10(P) | | --- | --- | --- | --- | --- | |  | MCODE\_1 | R-HSA-1428517 | The citric acid (TCA) cycle and respiratory electron transport | -5.7 | |  | MCODE\_1 | WP4324 | Mitochondrial complex I assembly model OXPHOS system | -5.5 | |  | MCODE\_1 | R-HSA-6799198 | Complex I biogenesis | -5.5 | |  | MCODE\_2 | R-HSA-6802948 | Signaling by high-kinase activity BRAF mutants | -19.8 | |  | MCODE\_2 | R-HSA-5674135 | MAP2K and MAPK activation | -19.5 | |  | MCODE\_2 | R-HSA-9656223 | Signaling by RAF1 mutants | -19.2 | |

|  |  |  |
| --- | --- | --- |
|  |  |  |
|  |  |  |
| Pink (Full Connection)  | GO | Description | Log10(P) | | --- | --- | --- | | GO:0009725 | response to hormone | -5.4 | | hsa05200 | Pathways in cancer | -5.3 | | GO:1903829 | positive regulation of protein localization | -4.9 | |  | Pink (Keep MCODE Nodes Only)  | Color | MCODE | GO | Description | Log10(P) | | --- | --- | --- | --- | --- | |  | MCODE\_1 | R-HSA-1257604 | PIP3 activates AKT signaling | -5.6 | |  | MCODE\_1 | R-HSA-9006925 | Intracellular signaling by second messengers | -5.4 | |  | MCODE\_1 | R-HSA-5663202 | Diseases of signal transduction by growth factor receptors and second messengers | -4.9 | |

|  |  |  |
| --- | --- | --- |
|  |  |  |
|  |  |  |
| Purple (Full Connection)  | GO | Description | Log10(P) | | --- | --- | --- | | GO:0051301 | cell division | -68.8 | | GO:0000278 | mitotic cell cycle | -60.8 | | GO:1903047 | mitotic cell cycle process | -56.6 | |  | Purple (Keep MCODE Nodes Only)  | Color | MCODE | GO | Description | Log10(P) | | --- | --- | --- | --- | --- | |  | MCODE\_1 | GO:0051301 | cell division | -32.5 | |  | MCODE\_1 | GO:1903047 | mitotic cell cycle process | -24.7 | |  | MCODE\_1 | GO:0000278 | mitotic cell cycle | -23.5 | |  | MCODE\_2 | GO:0098813 | nuclear chromosome segregation | -19.1 | |  | MCODE\_2 | GO:0000819 | sister chromatid segregation | -18.2 | |  | MCODE\_2 | GO:0000280 | nuclear division | -18.0 | |  | MCODE\_3 | R-HSA-2500257 | Resolution of Sister Chromatid Cohesion | -12.9 | |  | MCODE\_3 | GO:0007059 | chromosome segregation | -12.8 | |  | MCODE\_3 | R-HSA-68877 | Mitotic Prometaphase | -11.6 | |

|  |  |  |
| --- | --- | --- |
|  |  |  |
|  |  |  |
| Red (Full Connection)  | GO | Description | Log10(P) | | --- | --- | --- | | M5887 | NABA BASEMENT MEMBRANES | -5.0 | | WP4718 | Cholesterol metabolism with Bloch and Kandutsch-Russell pathways | -4.8 | | GO:0006914 | autophagy | -4.1 | |  | Red (Keep MCODE Nodes Only)  | Color | MCODE | GO | Description | Log10(P) | | --- | --- | --- | --- | --- | |  | MCODE\_1 | R-HSA-191273 | Cholesterol biosynthesis | -9.2 | |  | MCODE\_1 | R-HSA-2426168 | Activation of gene expression by SREBF (SREBP) | -8.6 | |  | MCODE\_1 | WP4718 | Cholesterol metabolism with Bloch and Kandutsch-Russell pathways | -8.5 | |

|  |  |  |
| --- | --- | --- |
|  |  |  |
|  |  |  |
| Tan (Full Connection)  | GO | Description | Log10(P) | | --- | --- | --- | | GO:0006457 | protein folding | -6.5 | | R-HSA-162909 | Host Interactions of HIV factors | -6.2 | | R-HSA-446203 | Asparagine N-linked glycosylation | -5.7 | |  | Tan (Keep MCODE Nodes Only)  | Color | MCODE | GO | Description | Log10(P) | | --- | --- | --- | --- | --- | |  | MCODE\_1 | R-HSA-199991 | Membrane Trafficking | -5.0 | |  | MCODE\_1 | R-HSA-5653656 | Vesicle-mediated transport | -5.0 | |

|  |  |  |
| --- | --- | --- |
|  |  |  |
|  |  |  |
| Turquoise (Full Connection)  | GO | Description | Log10(P) | | --- | --- | --- | | WP3888 | VEGFA-VEGFR2 signaling pathway | -18.2 | | GO:0097435 | supramolecular fiber organization | -15.2 | | R-HSA-9716542 | Signaling by Rho GTPases, Miro GTPases and RHOBTB3 | -14.1 | |  | Turquoise (Keep MCODE Nodes Only)  | Color | MCODE | GO | Description | Log10(P) | | --- | --- | --- | --- | --- | |  | MCODE\_1 | R-HSA-109582 | Hemostasis | -9.0 | |  | MCODE\_1 | WP3953 | mRNA, protein, and metabolite inducation pathway by cyclosporin A | -7.0 | |  | MCODE\_1 | R-HSA-9716542 | Signaling by Rho GTPases, Miro GTPases and RHOBTB3 | -6.1 | |  | MCODE\_2 | R-HSA-5658442 | Regulation of RAS by GAPs | -6.1 | |  | MCODE\_2 | GO:0022616 | DNA strand elongation | -6.0 | |  | MCODE\_2 | GO:0060541 | respiratory system development | -5.7 | |  | MCODE\_3 | R-HSA-6807878 | COPI-mediated anterograde transport | -9.7 | |  | MCODE\_3 | GO:0034314 | Arp2/3 complex-mediated actin nucleation | -9.0 | |  | MCODE\_3 | R-HSA-199991 | Membrane Trafficking | -8.9 | |  | MCODE\_4 | R-HSA-72203 | Processing of Capped Intron-Containing Pre-mRNA | -8.1 | |  | MCODE\_4 | hsa03040 | Spliceosome | -5.8 | |  | MCODE\_4 | R-HSA-8953854 | Metabolism of RNA | -5.8 | |  | MCODE\_5 | GO:0030433 | ubiquitin-dependent ERAD pathway | -5.1 | |  | MCODE\_5 | GO:0036503 | ERAD pathway | -4.8 | |  | MCODE\_5 | GO:0006259 | DNA metabolic process | -4.6 | |  | MCODE\_6 | CORUM:1181 | C complex spliceosome | -12.6 | |  | MCODE\_6 | R-HSA-72163 | mRNA Splicing - Major Pathway | -10.1 | |  | MCODE\_6 | R-HSA-72172 | mRNA Splicing | -10.0 | |  | MCODE\_7 | R-HSA-6798695 | Neutrophil degranulation | -6.1 | |  | MCODE\_7 | R-HSA-195258 | RHO GTPase Effectors | -5.2 | |  | MCODE\_7 | WP4540 | Hippo signaling regulation pathways | -5.1 | |  | MCODE\_8 | GO:0006260 | DNA replication | -9.6 | |  | MCODE\_8 | GO:0090329 | regulation of DNA-templated DNA replication | -9.4 | |  | MCODE\_8 | GO:0006261 | DNA-templated DNA replication | -7.9 | |  | MCODE\_9 | R-HSA-4086398 | Ca2+ pathway | -8.1 | |  | MCODE\_9 | hsa04720 | Long-term potentiation | -8.0 | |  | MCODE\_9 | hsa04924 | Renin secretion | -7.9 | |  | MCODE\_10 | GO:0051345 | positive regulation of hydrolase activity | -5.2 | |

|  |  |  |
| --- | --- | --- |
|  |  |  |
|  |  |  |
| Yellow (Full Connection)  | GO | Description | Log10(P) | | --- | --- | --- | | hsa05200 | Pathways in cancer | -7.9 | | GO:0001841 | neural tube formation | -7.6 | | GO:0021915 | neural tube development | -7.0 | |  | Yellow (Keep MCODE Nodes Only)  | Color | MCODE | GO | Description | Log10(P) | | --- | --- | --- | --- | --- | |  | MCODE\_1 | R-HSA-1368082 | RORA activates gene expression | -8.2 | |  | MCODE\_1 | R-HSA-1368108 | BMAL1:CLOCK,NPAS2 activates circadian gene expression | -7.7 | |  | MCODE\_1 | R-HSA-2426168 | Activation of gene expression by SREBF (SREBP) | -7.1 | |  | MCODE\_2 | GO:0006635 | fatty acid beta-oxidation | -16.3 | |  | MCODE\_2 | GO:0019395 | fatty acid oxidation | -15.5 | |  | MCODE\_2 | GO:0009062 | fatty acid catabolic process | -15.3 | |  | MCODE\_4 | GO:0016567 | protein ubiquitination | -6.6 | |  | MCODE\_4 | GO:0032446 | protein modification by small protein conjugation | -6.4 | |  | MCODE\_4 | R-HSA-8951664 | Neddylation | -5.7 | |

### Quality Control and Association Analysis

Gene list enrichments are identified in the following ontology categories: COVID, TRRUST, Transcription\_Factor\_Targets, Cell\_Type\_Signatures, DisGeNET, PaGenBase. All genes in the genome have been used as the enrichment background. Terms with a p-value < 0.01, a minimum count of 3, and an enrichment factor > 1.5 (the enrichment factor is the ratio between the observed counts and the counts expected by chance) are collected and grouped into clusters based on their membership similarities. The top few enriched clusters (one term per cluster) are shown in the Figure 6-11. The algorithm used here is the same as that is used for pathway and process enrichment analysis.

Figure 6. Summary of enrichment analysis in COVID13.

|  |
| --- |
|  |
|  |
| | \_PATTERN\_ | GO | Description | Count | % | Log10(P) | Log10(q) | | --- | --- | --- | --- | --- | --- | --- | |  | COVID054 | RNA\_Xiong\_PBMC\_Up | 36 | 44.00 | -50.00 | -47.00 | |  | COVID007 | RNA\_Blanco-Melo\_A549\_Down | 23 | 28.00 | -27.00 | -24.00 | |  | COVID015 | RNA\_Blanco-Melo\_Calu-3\_Down | 23 | 28.00 | -27.00 | -24.00 | |  | COVID134 | Proteome\_Stukalov\_A549-ACE2\_24h\_Down | 19 | 23.00 | -20.00 | -17.00 | |  | COVID126 | Interactome\_Stukalov\_A549\_72h\_ORF7B | 29 | 7.60 | -17.00 | -14.00 | |  | COVID360 | RNA\_Zhang\_B-cells\_severe-only\_Up | 17 | 4.50 | -16.00 | -13.00 | |  | COVID039 | RNA\_Sun\_Calu-3\_24h\_Down | 30 | 6.00 | -14.00 | -12.00 | |  | COVID005 | RNA\_Appelberg\_Huh-7\_72h\_Down | 22 | 8.90 | -14.00 | -11.00 | |  | COVID037 | RNA\_Sun\_Calu-3\_12h\_Down | 21 | 8.50 | -13.00 | -10.00 | |  | COVID059 | Phosphoproteome\_Bouhaddou\_Vero\_E6\_24h\_Down | 13 | 16.00 | -12.00 | -9.30 | |  | COVID071 | Proteome\_Bouhaddou\_Vero\_E6\_24h\_Down | 9 | 11.00 | -11.00 | -8.90 | |  | COVID011 | RNA\_Blanco-Melo\_A549-ACE2-ruxolitinib\_Down | 21 | 5.30 | -11.00 | -8.80 | |  | COVID228 | Translatome\_Bojkova\_Caco-2\_24h\_Down | 18 | 4.70 | -11.00 | -8.70 | |  | COVID055 | Phosphoproteome\_Bouhaddou\_Vero\_E6\_0h\_Down | 12 | 15.00 | -11.00 | -8.50 | |  | COVID009 | RNA\_Blanco-Melo\_A549-ACE2\_Down | 16 | 6.50 | -11.00 | -8.30 | |  | COVID341 | RNA\_Wilk\_B-cells\_patient-C3\_Up | 11 | 4.50 | -10.00 | -7.80 | |  | COVID050 | RNA\_Wyler\_Calu-3\_24h\_Up | 22 | 5.60 | -10.00 | -7.70 | |  | COVID038 | RNA\_Sun\_Calu-3\_12h\_Up | 16 | 7.80 | -9.50 | -7.20 | |  | COVID235 | Phosphoproteome\_Klann\_Caco-2\_24h\_Up | 16 | 7.80 | -9.50 | -7.20 | |  | COVID049 | RNA\_Wyler\_Calu-3\_24h\_Down | 11 | 14.00 | -9.30 | -7.00 | |

Figure 7. Summary of enrichment analysis in TRRUST.

|  |
| --- |
|  |
|  |
| | \_PATTERN\_ | GO | Description | Count | % | Log10(P) | Log10(q) | | --- | --- | --- | --- | --- | --- | --- | |  | TRR00230 | Regulated by: E2F1 | 12 | 15.00 | -14.00 | -12.00 | |  | TRR01419 | Regulated by: TP53 | 9 | 11.00 | -9.00 | -6.80 | |  | TRR00233 | Regulated by: E2F4 | 5 | 6.20 | -8.20 | -6.10 | |  | TRR00232 | Regulated by: E2F3 | 4 | 4.90 | -7.50 | -5.50 | |  | TRR00645 | Regulated by: JUN | 13 | 2.60 | -5.20 | -3.60 | |  | TRR01256 | Regulated by: SP1 | 24 | 4.80 | -5.00 | -3.50 | |  | TRR00780 | Regulated by: MYC | 10 | 2.00 | -4.80 | -3.30 | |  | TRR01421 | Regulated by: TP63 | 4 | 1.40 | -4.60 | -3.20 | |  | TRR00484 | Regulated by: HIF1A | 9 | 1.80 | -4.40 | -3.00 | |  | TRR01071 | Regulated by: PTTG1 | 4 | 1.00 | -4.30 | -2.90 | |  | TRR01277 | Regulated by: STAT3 | 8 | 3.20 | -4.20 | -2.90 | |  | TRR00466 | Regulated by: HDAC1 | 8 | 1.60 | -4.20 | -2.90 | |  | TRR01072 | Regulated by: PURA | 3 | 0.79 | -4.20 | -2.80 | |  | TRR00342 | Regulated by: FOS | 7 | 1.40 | -4.00 | -2.70 | |  | TRR01275 | Regulated by: STAT1 | 4 | 5.20 | -4.00 | -2.70 | |  | TRR00366 | Regulated by: FOXO3 | 4 | 1.00 | -3.90 | -2.70 | |  | TRR01546 | Regulated by: YBX1 | 3 | 3.70 | -3.90 | -2.60 | |  | TRR01155 | Regulated by: RBMX | 3 | 0.76 | -3.80 | -2.50 | |  | TRR01548 | Regulated by: YY1 | 7 | 1.80 | -3.60 | -2.40 | |  | TRR00029 | Regulated by: ATM | 4 | 1.00 | -3.40 | -2.30 | |

Figure 8. Summary of enrichment analysis in Transcription Factor Targets14.

|  |
| --- |
|  |
|  |
| | \_PATTERN\_ | GO | Description | Count | % | Log10(P) | Log10(q) | | --- | --- | --- | --- | --- | --- | --- | |  | M30019 | HSD17B8 TARGET GENES | 63 | 78.00 | -88.00 | -85.00 | |  | M40770 | ATXN7L3 TARGET GENES | 29 | 5.80 | -14.00 | -11.00 | |  | M30131 | PSMB5 TARGET GENES | 30 | 6.00 | -14.00 | -11.00 | |  | M13493 | E4F1 Q6 | 23 | 6.00 | -11.00 | -8.90 | |  | M17925 | MGGAAGTG GABP B | 37 | 9.70 | -11.00 | -8.80 | |  | M30333 | ZNF507 TARGET GENES | 23 | 11.00 | -9.80 | -7.50 | |  | M4220 | E2F 03 | 22 | 4.40 | -9.60 | -7.30 | |  | M17736 | E2F1 Q3 01 | 22 | 4.40 | -9.60 | -7.30 | |  | M12443 | SRF C | 15 | 6.10 | -9.50 | -7.20 | |  | M171 | GCCATNTTG YY1 Q6 | 25 | 6.60 | -9.30 | -7.10 | |  | M9645 | GGGYGTGNY UNKNOWN | 37 | 7.40 | -9.30 | -7.00 | |  | M1905 | SGCGSSAAA E2F1DP2 01 | 18 | 3.60 | -9.20 | -7.00 | |  | M29943 | DLX6 TARGET GENES | 34 | 6.80 | -9.20 | -7.00 | |  | M3403 | GTGACGY E4F1 Q6 | 31 | 8.10 | -9.20 | -6.90 | |  | M10112 | RNGTGGGC UNKNOWN | 38 | 7.60 | -8.30 | -6.10 | |  | M8101 | NGFIC 01 | 20 | 4.00 | -7.90 | -5.80 | |  | M5768 | E2F1DP1RB 01 | 9 | 11.00 | -7.90 | -5.80 | |  | M40742 | GTF2A2 TARGET GENES | 25 | 6.60 | -7.80 | -5.70 | |  | M10526 | E2F4DP1 01 | 9 | 11.00 | -7.70 | -5.70 | |  | M15729 | E2F Q6 01 | 9 | 11.00 | -7.70 | -5.70 | |

Figure 9. Summary of enrichment analysis in Cell Type Signatures.

|  |
| --- |
|  |
|  |
| | \_PATTERN\_ | GO | Description | Count | % | Log10(P) | Log10(q) | | --- | --- | --- | --- | --- | --- | --- | |  | M39036 | FAN EMBRYONIC CTX NSC 2 | 65 | 80.00 | -100.00 | -96.00 | |  | M39041 | FAN EMBRYONIC CTX MICROGLIA 1 | 65 | 80.00 | -100.00 | -96.00 | |  | M39096 | ZHONG PFC C1 OPC | 70 | 86.00 | -100.00 | -96.00 | |  | M39059 | MANNO MIDBRAIN NEUROTYPES HPROGBP | 58 | 72.00 | -99.00 | -95.00 | |  | M39061 | MANNO MIDBRAIN NEUROTYPES HPROGFPM | 58 | 72.00 | -94.00 | -90.00 | |  | M39060 | MANNO MIDBRAIN NEUROTYPES HPROGFPL | 55 | 68.00 | -90.00 | -86.00 | |  | M39078 | ZHONG PFC MAJOR TYPES NPCS | 42 | 52.00 | -77.00 | -74.00 | |  | M39103 | ZHONG PFC C1 MICROGLIA | 45 | 56.00 | -72.00 | -68.00 | |  | M39165 | GAO LARGE INTESTINE ADULT CH MKI67HIGH CELLS | 36 | 44.00 | -68.00 | -65.00 | |  | M39208 | HAY BONE MARROW PRO B | 42 | 52.00 | -62.00 | -59.00 | |  | M39087 | ZHONG PFC C2 UNKNOWN NPC | 31 | 38.00 | -61.00 | -58.00 | |  | M41687 | TRAVAGLINI LUNG PROLIFERATING NK T CELL | 33 | 41.00 | -57.00 | -54.00 | |  | M39062 | MANNO MIDBRAIN NEUROTYPES HNPROG | 33 | 41.00 | -48.00 | -45.00 | |  | M39175 | MURARO PANCREAS MESENCHYMAL STROMAL CELL | 64 | 26.00 | -48.00 | -45.00 | |  | M39153 | GAO LARGE INTESTINE 24W C2 MKI67POS PROGENITOR | 26 | 32.00 | -43.00 | -40.00 | |  | M39081 | ZHONG PFC C8 ORG PROLIFERATING | 22 | 27.00 | -41.00 | -38.00 | |  | M41717 | FAN OVARY CL15 SMALL ANTRAL FOLLICLE GRANULOSA CELL | 64 | 17.00 | -37.00 | -34.00 | |  | M39083 | ZHONG PFC C3 UNKNOWN INP | 18 | 22.00 | -37.00 | -33.00 | |  | M39058 | MANNO MIDBRAIN NEUROTYPES HPROGM | 26 | 32.00 | -31.00 | -28.00 | |  | M40010 | BUSSLINGER GASTRIC ISTHMUS CELLS | 41 | 17.00 | -30.00 | -26.00 | |

Figure 10. Summary of enrichment analysis in DisGeNET15.

|  |
| --- |
|  |
|  |
| | \_PATTERN\_ | GO | Description | Count | % | Log10(P) | Log10(q) | | --- | --- | --- | --- | --- | --- | --- | |  | C0205696 | Anaplastic carcinoma | 27 | 5.40 | -15.00 | -12.00 | |  | C1328504 | Hormone refractory prostate cancer | 19 | 23.00 | -14.00 | -11.00 | |  | C0812413 | Malignant Pleural Mesothelioma | 33 | 6.60 | -13.00 | -10.00 | |  | C0175754 | Agenesis of corpus callosum | 17 | 21.00 | -12.00 | -9.70 | |  | C0025500 | Mesothelioma | 37 | 7.40 | -12.00 | -9.30 | |  | C0025286 | Meningioma | 39 | 7.80 | -11.00 | -9.00 | |  | C0278996 | Malignant Head and Neck Neoplasm | 43 | 8.50 | -11.00 | -8.80 | |  | C1136382 | Sclerocystic Ovaries | 10 | 12.00 | -11.00 | -8.70 | |  | C0007138 | Carcinoma, Transitional Cell | 16 | 20.00 | -11.00 | -8.60 | |  | C4551472 | Hypertrophic obstructive cardiomyopathy | 17 | 6.90 | -11.00 | -8.50 | |  | C3887461 | Head and Neck Carcinoma | 43 | 8.50 | -11.00 | -8.50 | |  | C0205698 | Undifferentiated carcinoma | 25 | 5.00 | -11.00 | -8.40 | |  | C0036857 | Severe intellectual disability | 20 | 9.70 | -11.00 | -8.30 | |  | C0025990 | Micrognathism | 36 | 7.20 | -11.00 | -8.20 | |  | C0205699 | Carcinomatosis | 20 | 4.00 | -10.00 | -7.90 | |  | C0521158 | Recurrent tumor | 16 | 20.00 | -10.00 | -7.70 | |  | C0205697 | Carcinoma, Spindle-Cell | 21 | 4.20 | -9.90 | -7.50 | |  | C0278876 | Adult Medulloblastoma | 40 | 8.00 | -9.70 | -7.30 | |  | C0030297 | Pancreatic Neoplasm | 40 | 8.00 | -9.60 | -7.30 | |  | C0007194 | Hypertrophic Cardiomyopathy | 23 | 9.30 | -9.50 | -7.30 | |

Figure 11. Summary of enrichment analysis in PaGenBase16.

|  |
| --- |
|  |
|  |
| | \_PATTERN\_ | GO | Description | Count | % | Log10(P) | Log10(q) | | --- | --- | --- | --- | --- | --- | --- | |  | PGB:00015 | Tissue-specific: Smooth Muscle | 21 | 4.20 | -16.00 | -14.00 | |  | PGB:00049 | Cell-specific: Adipocyte | 17 | 4.30 | -11.00 | -8.60 | |  | PGB:00067 | Cell-specific: Brain cell | 13 | 2.60 | -10.00 | -7.80 | |  | PGB:00060 | Tissue-specific: retinoblastoma | 7 | 8.60 | -8.10 | -6.00 | |  | PGB:00101 | Tissue-specific: Colorectal adenocarcinoma | 5 | 6.20 | -7.10 | -5.10 | |  | PGB:00031 | Cell-specific: HUVEC | 22 | 4.40 | -6.40 | -4.60 | |  | PGB:00137 | Cell-specific: LN18 | 3 | 3.90 | -5.60 | -3.90 | |  | PGB:00081 | Cell-specific: Bronchial Epithelial Cells | 12 | 2.40 | -5.50 | -3.80 | |  | PGB:00120 | Cell-specific: Cardiac Myocytes | 8 | 1.60 | -5.20 | -3.60 | |  | PGB:00014 | Cell-specific: DRG | 19 | 4.80 | -5.00 | -3.40 | |  | PGB:00131 | Cell-specific: B-lymphocyte | 6 | 1.50 | -4.60 | -3.20 | |  | PGB:00051 | Tissue-specific: Cerebellum | 8 | 2.70 | -4.10 | -2.80 | |  | PGB:00165 | Cell-specific: BDCA4+ Dentritic Cells | 5 | 1.30 | -3.10 | -2.10 | |  | PGB:00026 | Cell-specific: CD71+ EarlyErythroid | 6 | 2.40 | -2.90 | -1.90 | |  | PGB:00043 | Cell-specific: CD56+ NKCells | 5 | 1.30 | -2.80 | -1.90 | |  | PGB:00010 | Tissue-specific: adipose tissue | 8 | 2.00 | -2.70 | -1.80 | |  | PGB:00045 | Tissue-specific: placenta | 11 | 2.80 | -2.60 | -1.80 | |  | PGB:00016 | Tissue-specific: thymus | 4 | 4.90 | -2.60 | -1.70 | |  | PGB:00048 | Tissue-specific: bone marrow | 4 | 4.90 | -2.50 | -1.70 | |  | PGB:00044 | Tissue-specific: skeletal muscle | 12 | 3.00 | -2.30 | -1.50 | |

### Reference

1. Zhou et al., Metascape provides a biologist-oriented resource for the analysis of systems-level datasets. Nature Communications (2019) 10(1):1523.
2. Saldanha AJ. Java Treeview - extensible visualization of microarray data. Bioinformatics (2004) 20:3246-3248
3. Krzywinski M. et al. Circos: an information aesthetic for comparative genomics. Genome Res (2009) 19:1639-1645
4. Zar, J.H. Biostatistical Analysis 1999 4th edn., NJ Prentice Hall, pp. 523
5. Hochberg Y., Benjamini Y. More powerful procedures for multiple significance testing. Statistics in Medicine (1990) 9:811-818.
6. Cohen, J. A coefficient of agreement for nominal scales. Educ. Psychol. Meas. (1960) 20:27-46.
7. Shannon P. et al., Cytoscape: a software environment for integrated models of biomolecular interaction networks. Genome Res (2003) 11:2498-2504.
8. Szklarczyk D. et al. STRING v11: protein-protein association networks with increased coverage, supporting functional discovery in genome-wide experimental datasets. Nucleic Acids Res. (2019) 47:D607-613.
9. Stark C. et al. BioGRID: a general repository for interaction datasets. Nucleic Acids Res. (2006) 34:D535-539.
10. Turei D. et al. A scored human protein-protein interaction network to catalyze genomic interpretation. Nat. Methods. (2016) 13:966-967.
11. Li T. et al. A scored human protein-protein interaction network to catalyze genomic interpretation. Nat. Methods. (2017) 14:61-64.
12. Bader, G.D. et al. An automated method for finding molecular complexes in large protein interaction networks. BMC bioinformatics (2003) 4:2.
13. https://metascape.org/COVID.
14. Subramanian A, et al. Gene set enrichment analysis: A knowledge-based approach for interpreting genome-wide expression profiles. Proc Natl Acad Sci U S A 102, 15545-15550 (2005).
15. Pinero J, et al. DisGeNET: a comprehensive platform integrating information on human disease-associated genes and variants. Nucleic acids research 45, D833-D839 (2017).
16. Pan JB, et al. PaGenBase: a pattern gene database for the global and dynamic understanding of gene function. PLoS One 8, e80747 (2013).
