# Supplementary material for: Fibroblasts as an in vitro model of circadian genetic and genomic studies
Source: Mamm Genome. 2024 Jul 3;35(3):432–44. doi: 10.1007/s00335-024-10050-7 (PMC11329553; doi:10.1007/s00335-024-10050-7)
Supplement: Supplementary file 8 — Supplementary file8 (DOCX 7854 kb) [file 335_2024_10050_MOESM8_ESM.docx]

**Supplementary Materials**

**Supplementary Figure 1 Principal component analysis of RNA-seq temporal dataset**

**
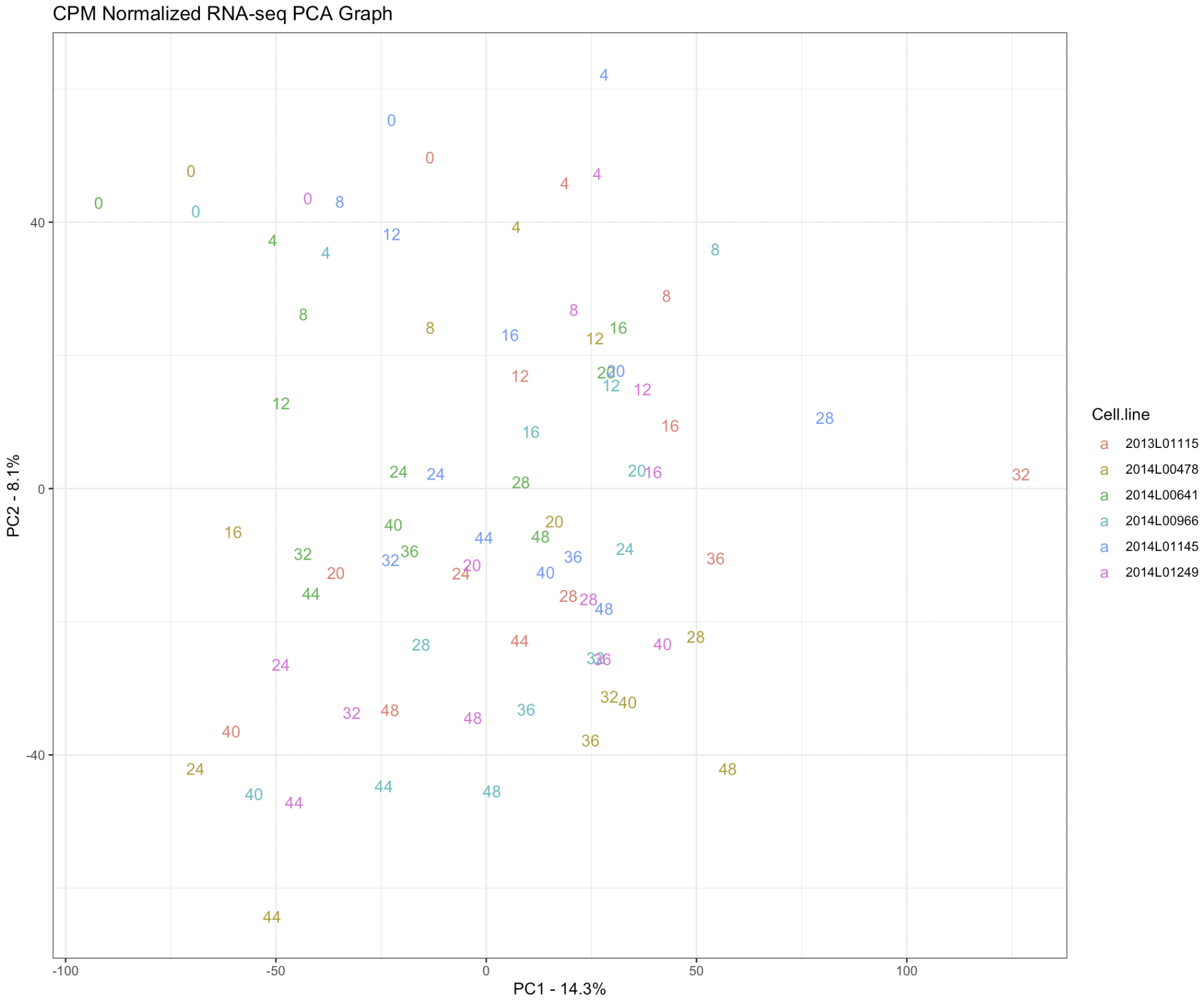
**

**Supplementary Figure 1 Description:** Principal component component representation of RNA-seq data after CPM(counts per million) normalization. Colors indicate each individual cell line used in this study. Numbers in the plot indicate the corresponding time point for that cell line.

**Supplementary Figure 2 Circadian-bioluminescence transduction experiment results**

**A
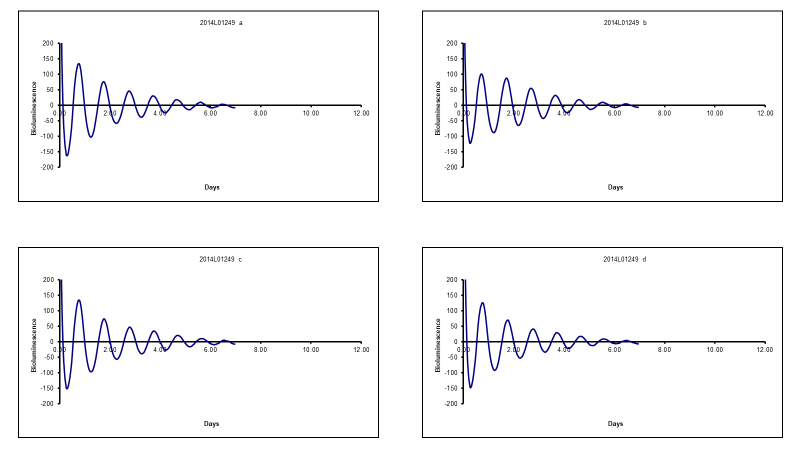
**

**B
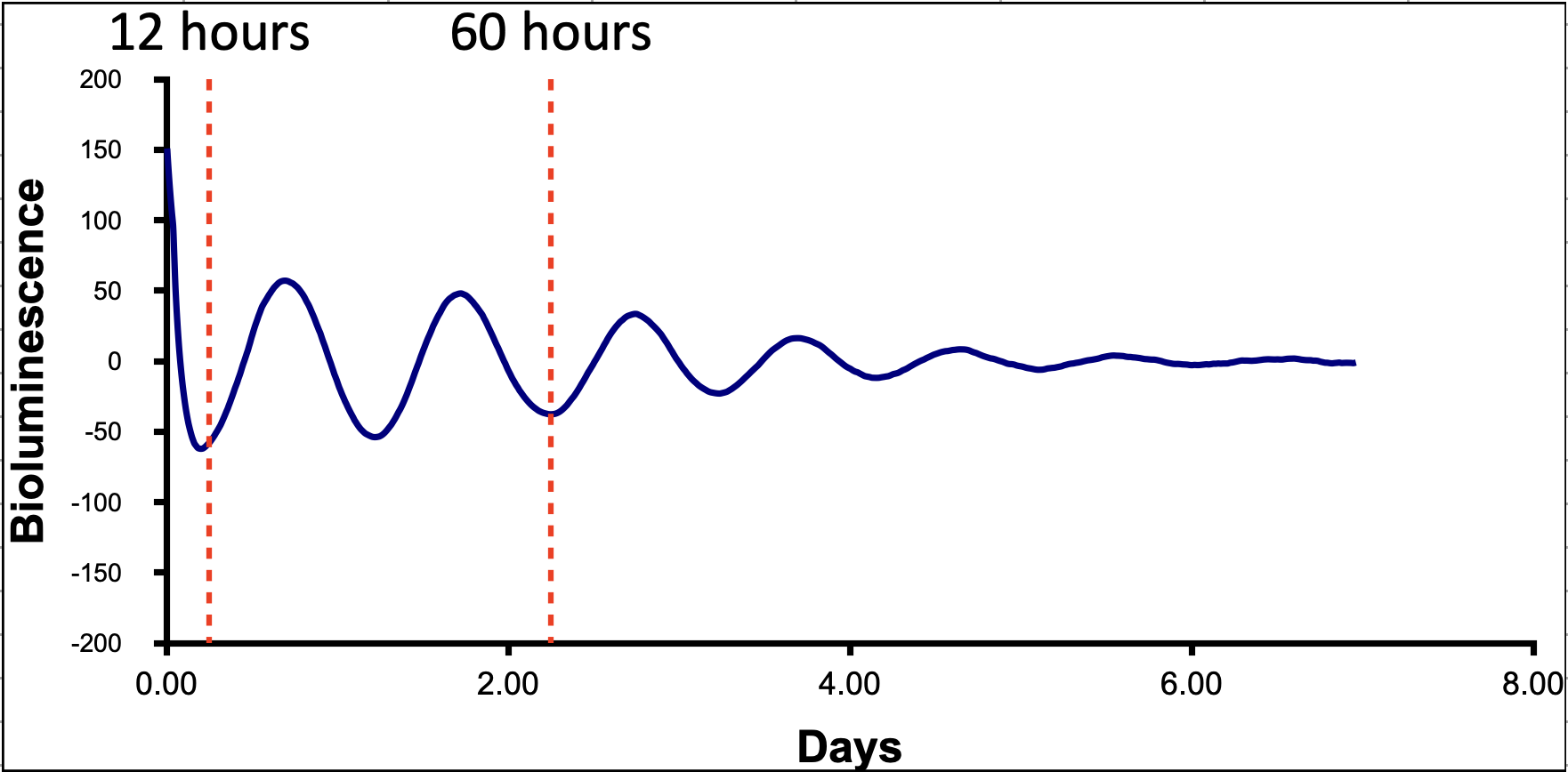
**

**Supplementary Figure 2 Description**: Example of circadian bioluminescence assay performed using transduced primary fibroblast cell lines with a Bmail1:luc construct, as described previously by Brown, et al. 2005 (The Period Length of Fibroblast Circadian

Gene Expression Varies Widely among Human Individuals). A. Bioluminescence measurements of cell cultures following synchronization with dexamethasone. B. Red lines indicate the time period during which RNA-seq and ATAC-seq data was collected from the cell lines.

**Supplementary Figure 3 WGCNA modules obtained from the RNA-seq temporal dataset
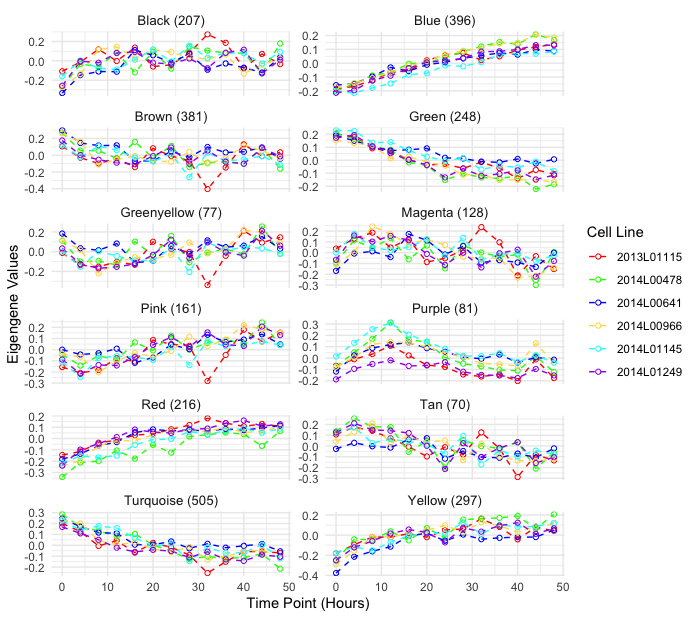
**

**Supplementary Figure 3 description**: WGCNA modules obtained from n=2,767 genes identified as having a significant effect of time in their expression through cubic splines modeling. Expression values are represented here as eigengene values. Names of the modules were assigned by WGCNA. The number of genes assigned per module is next to the module name.

**Supplementary figure 4 Mixed non-lin**
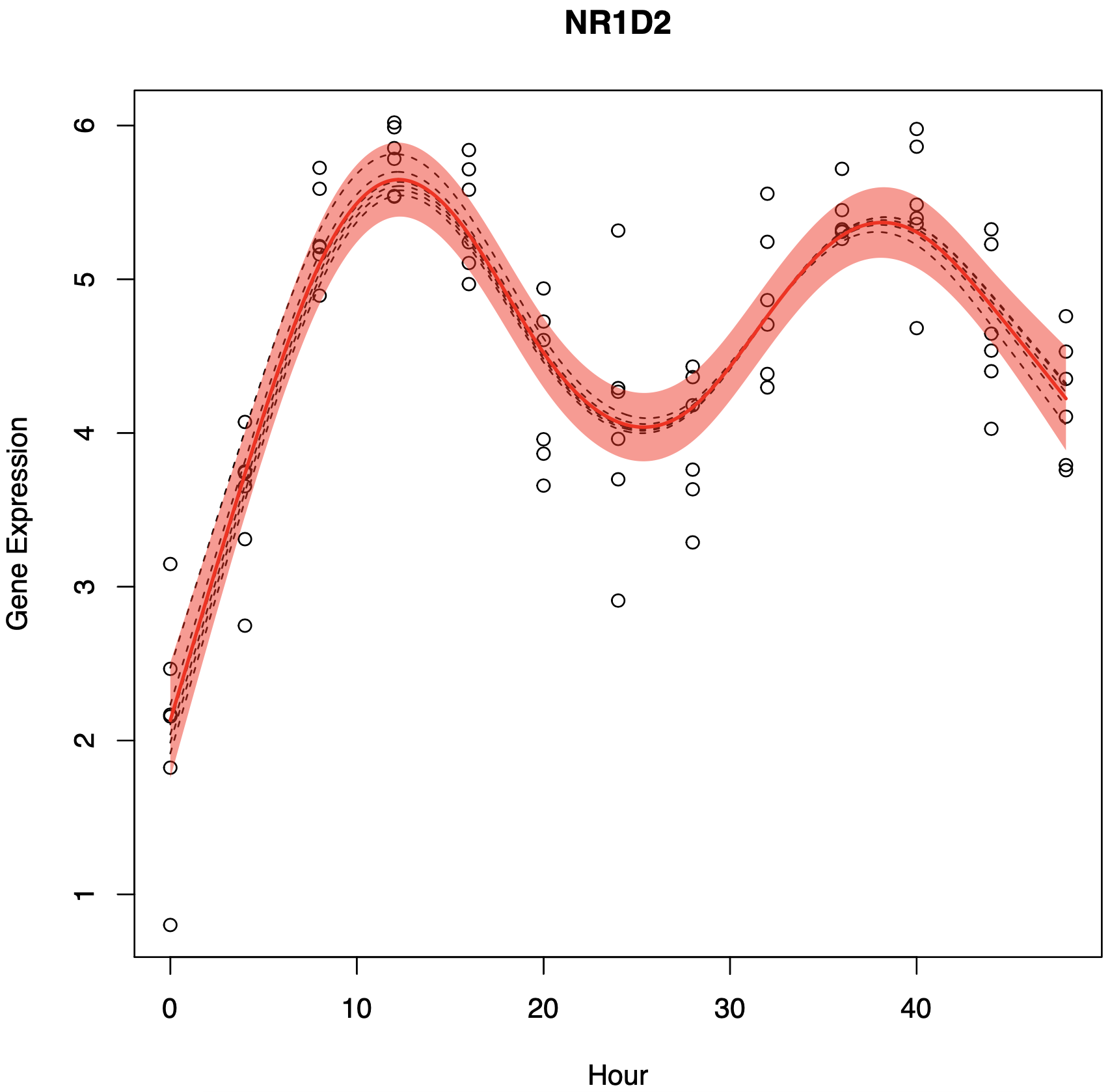
**ear modeling of circadian genes**

**A**


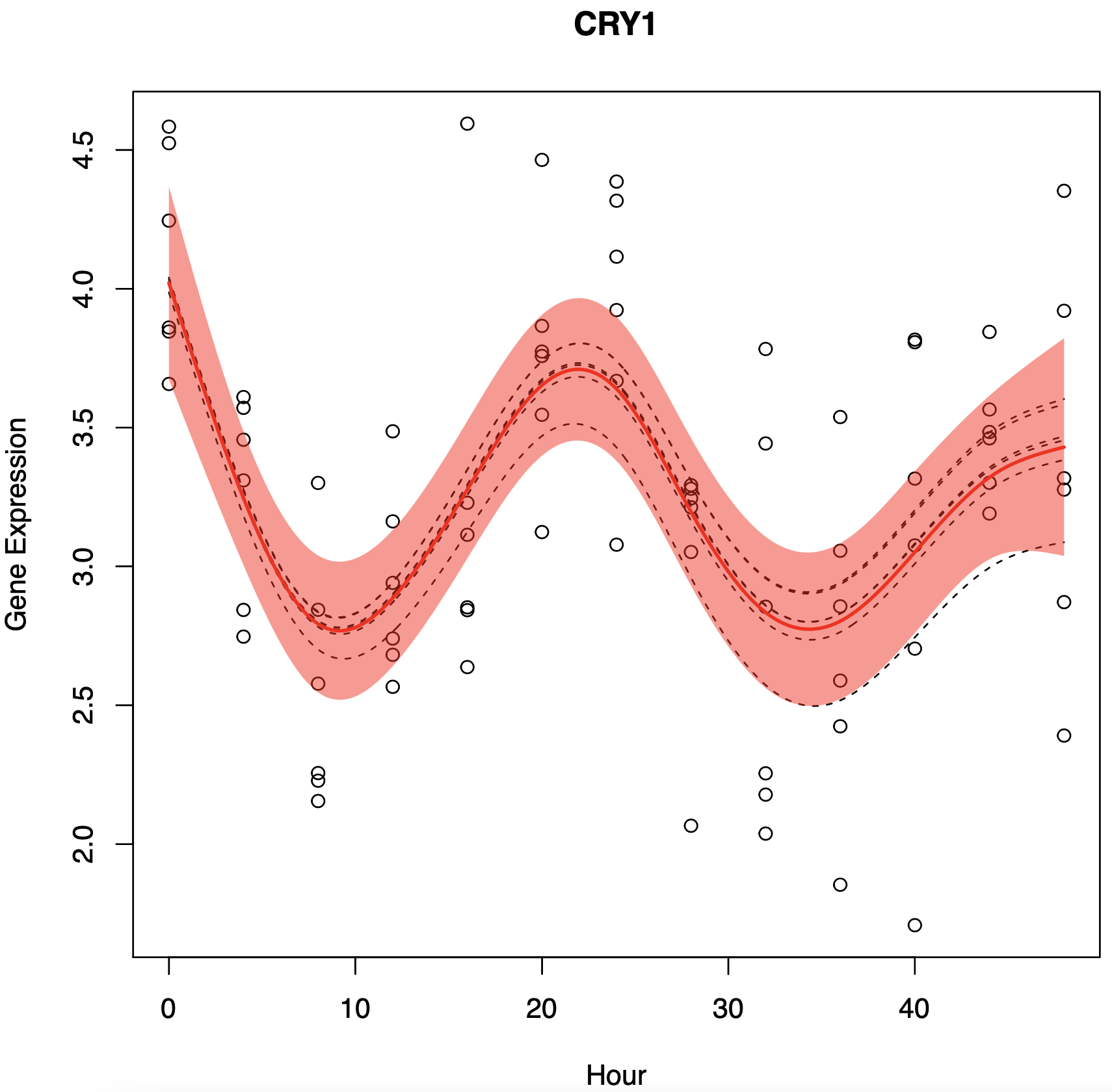

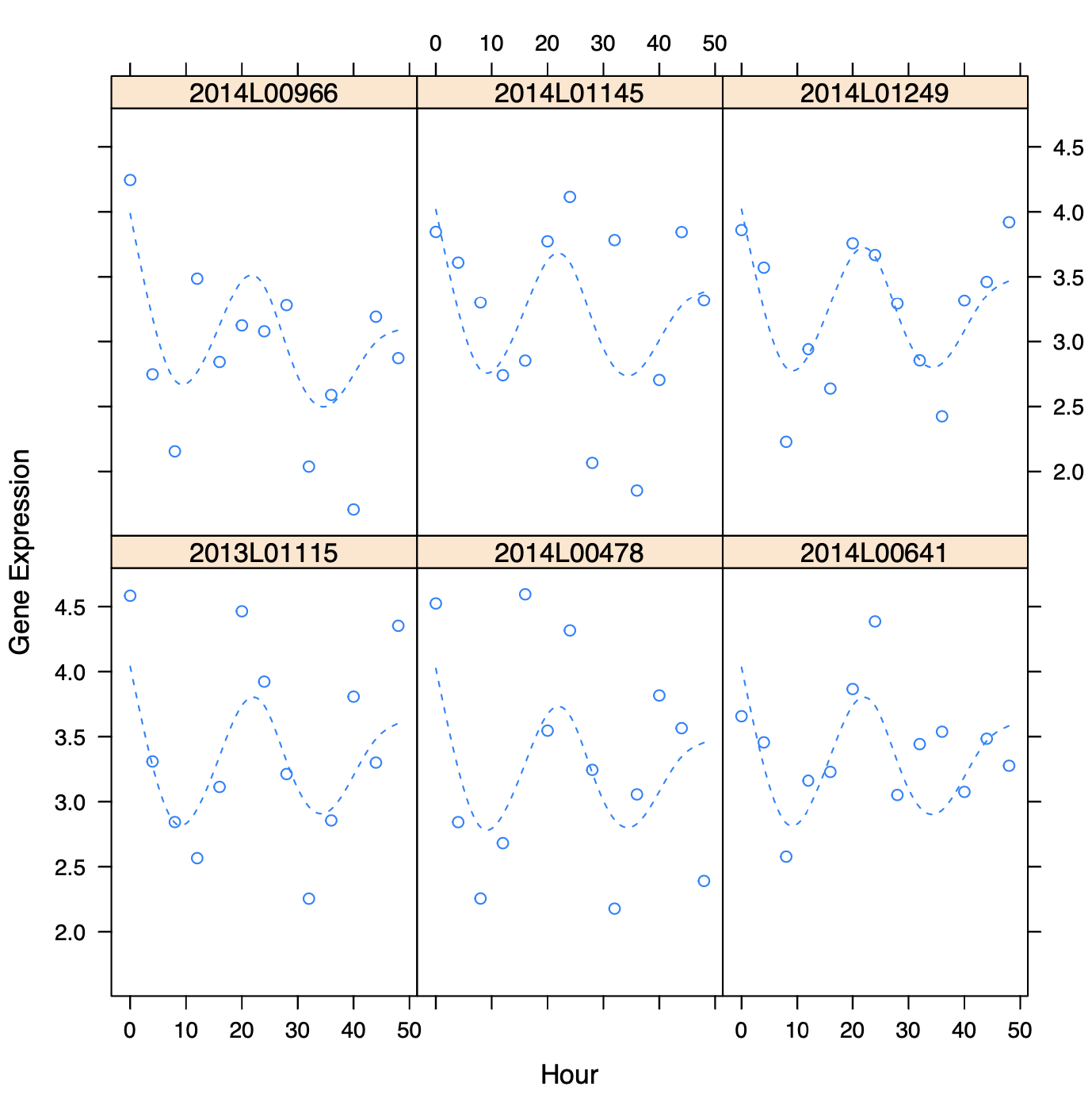

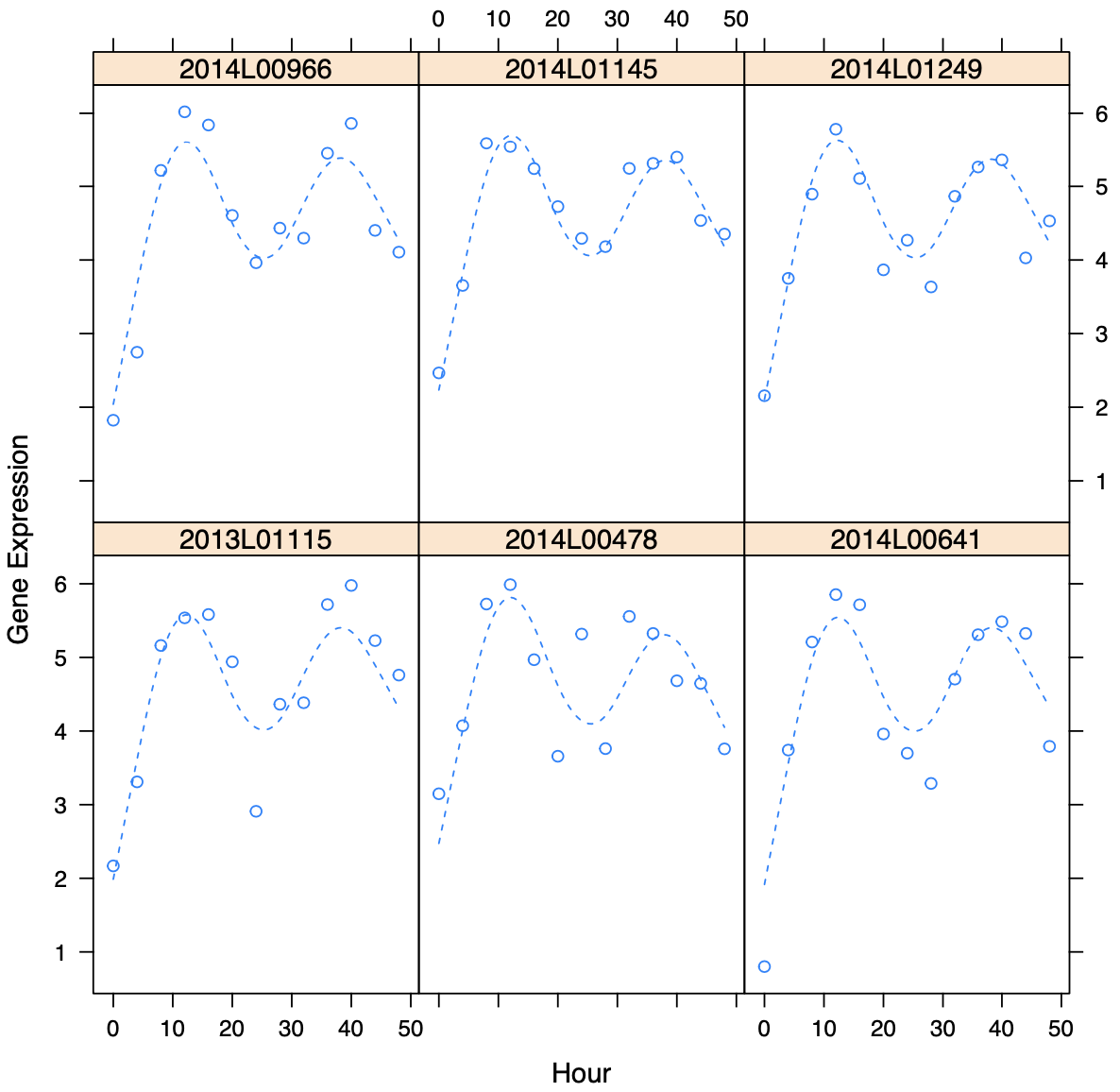


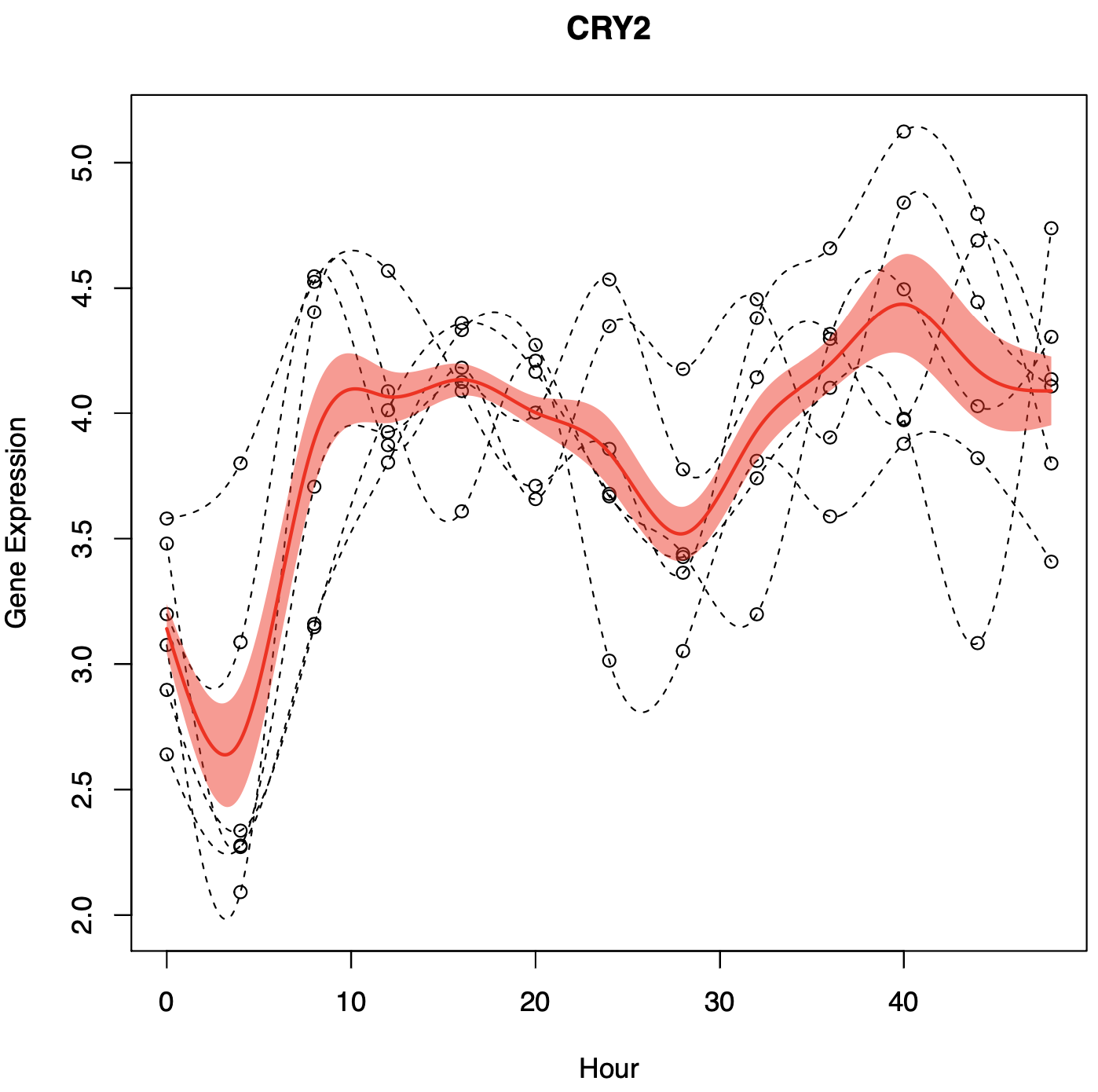

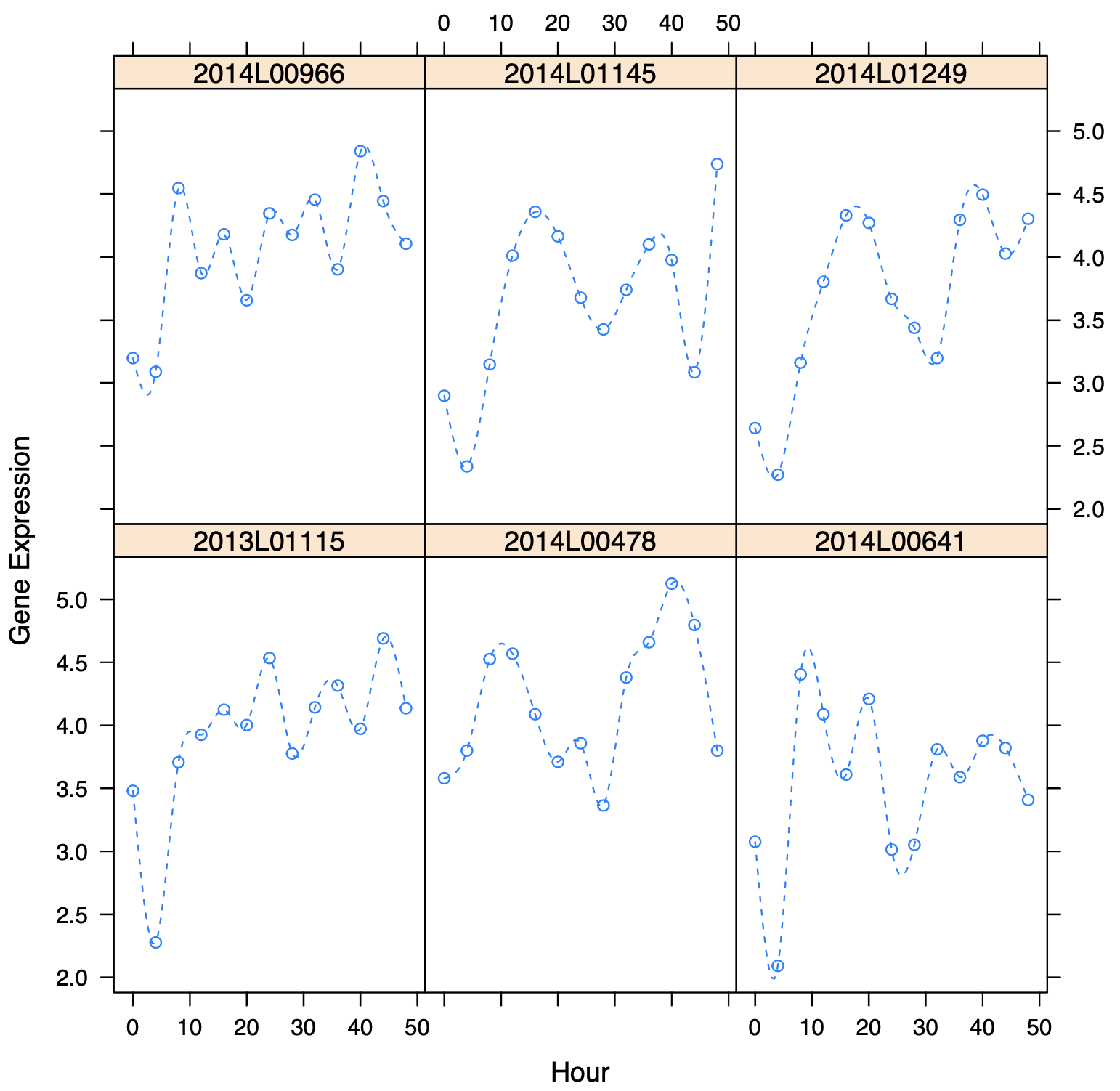


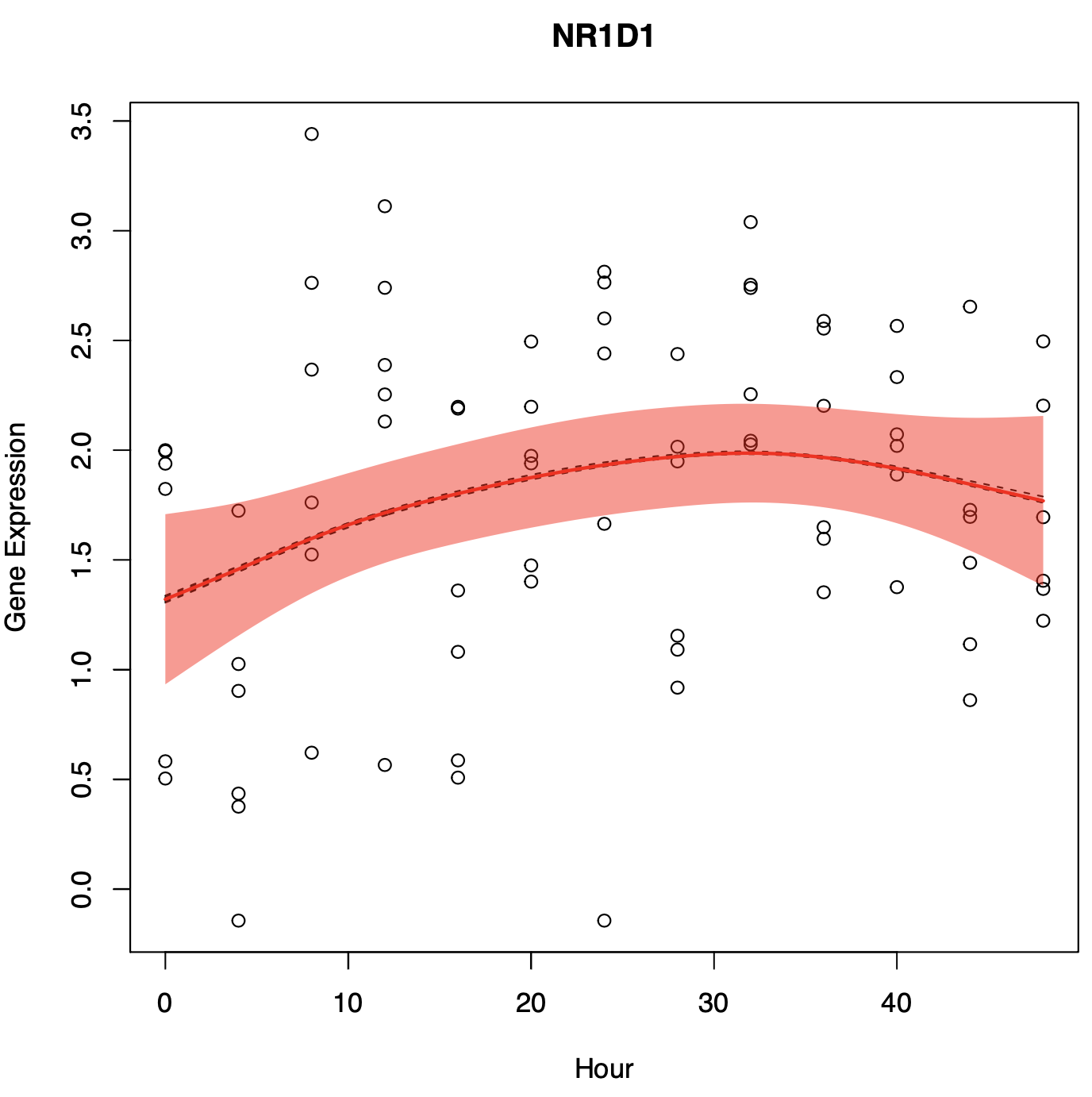

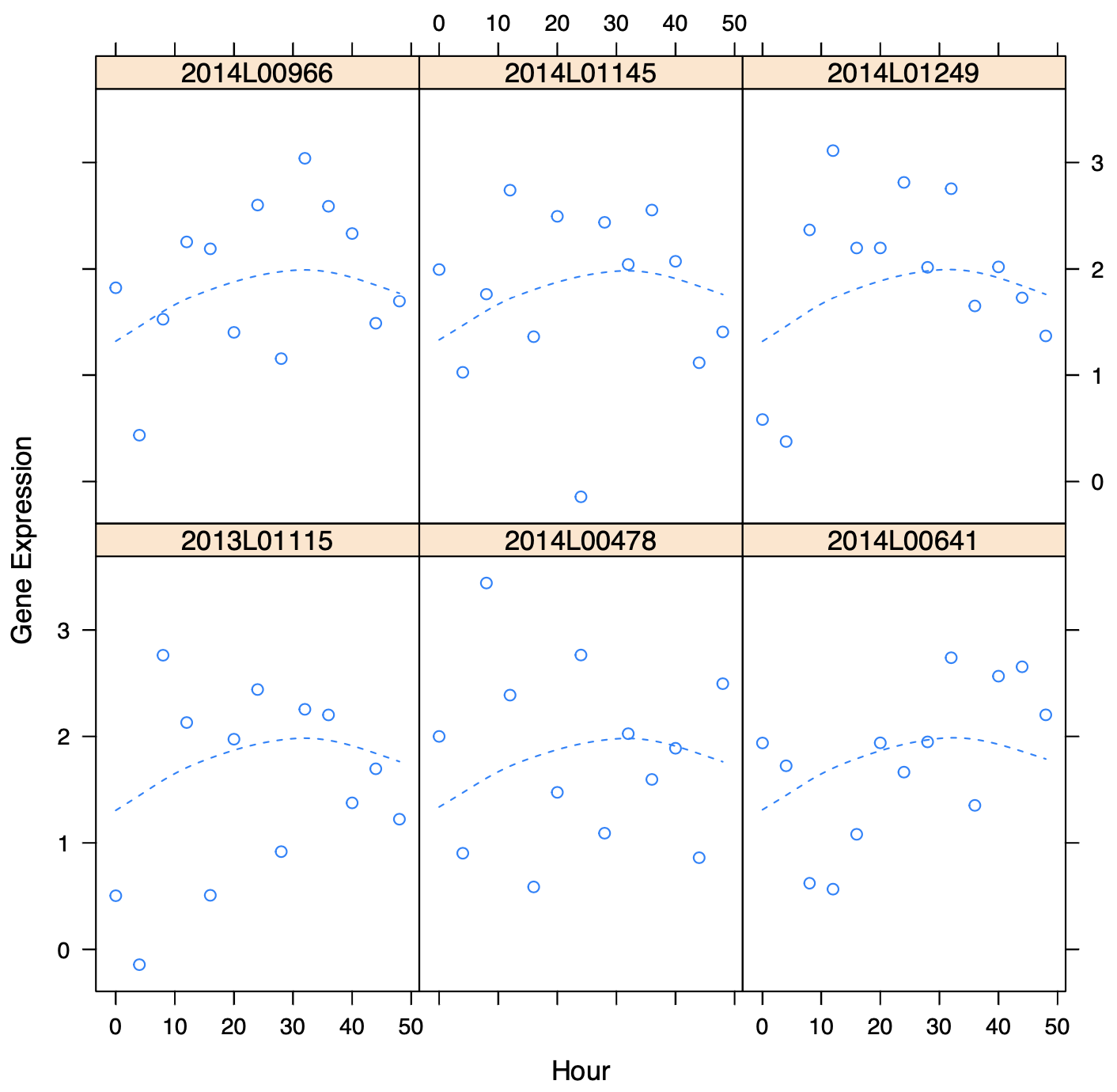


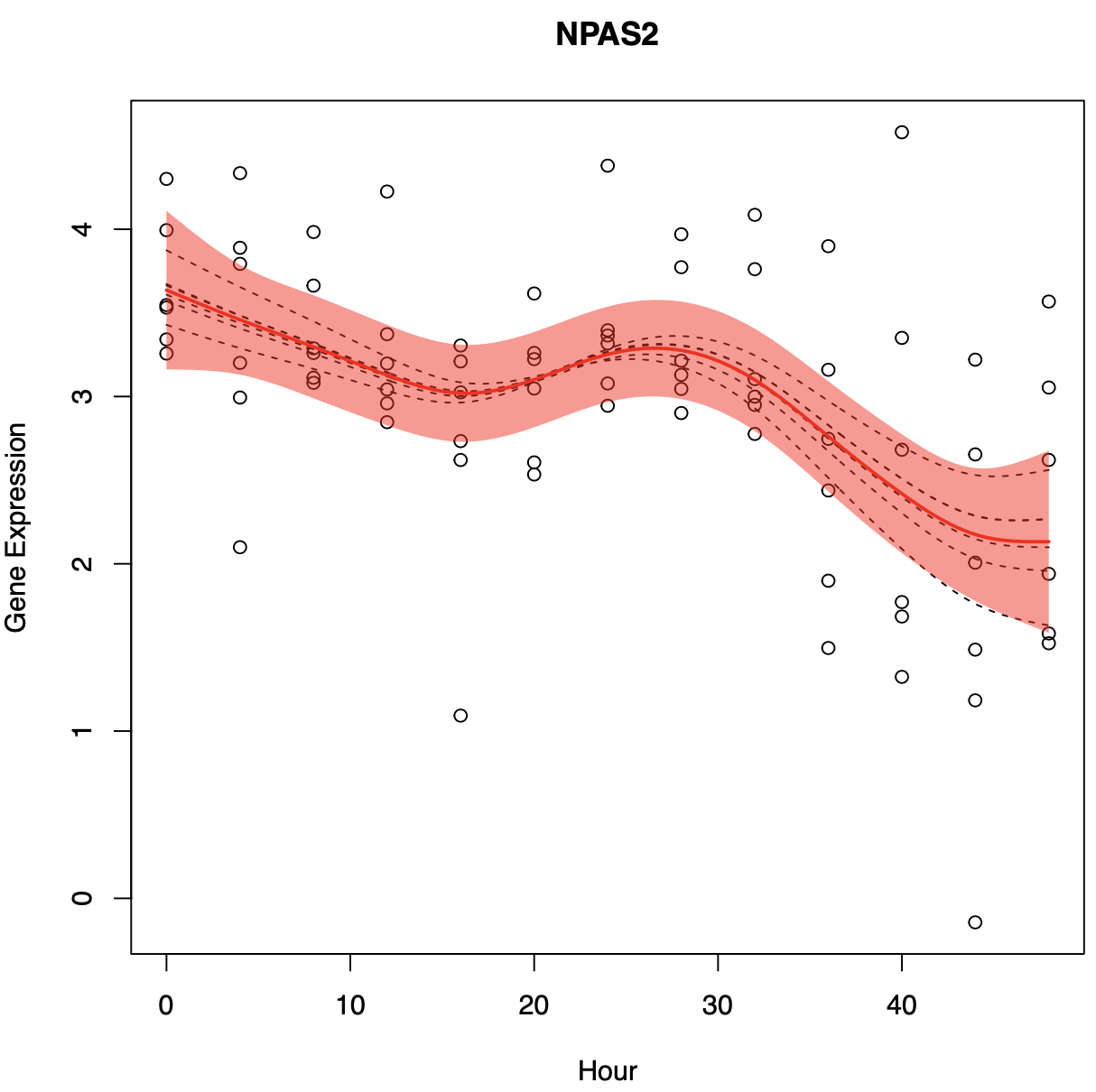

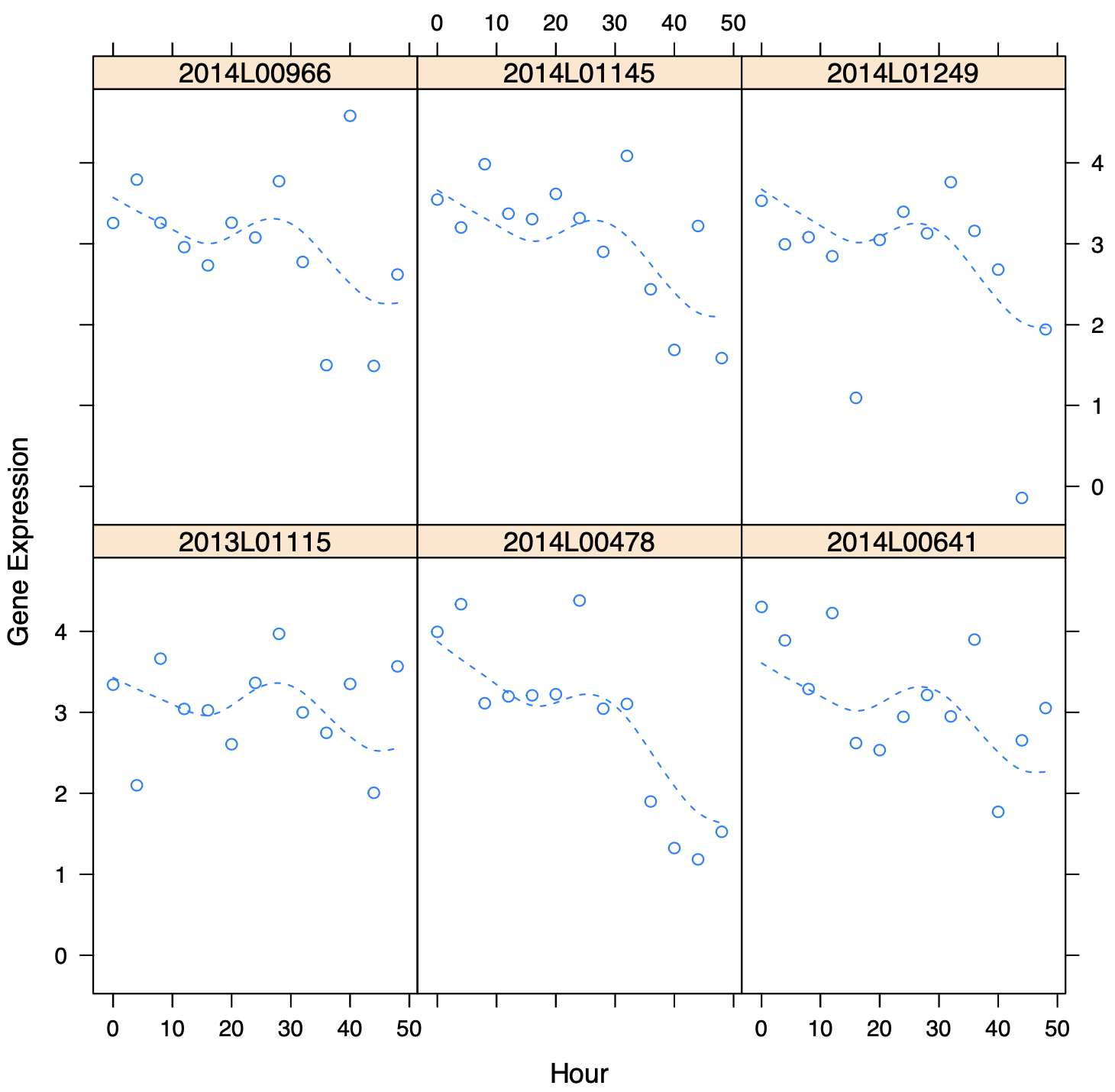


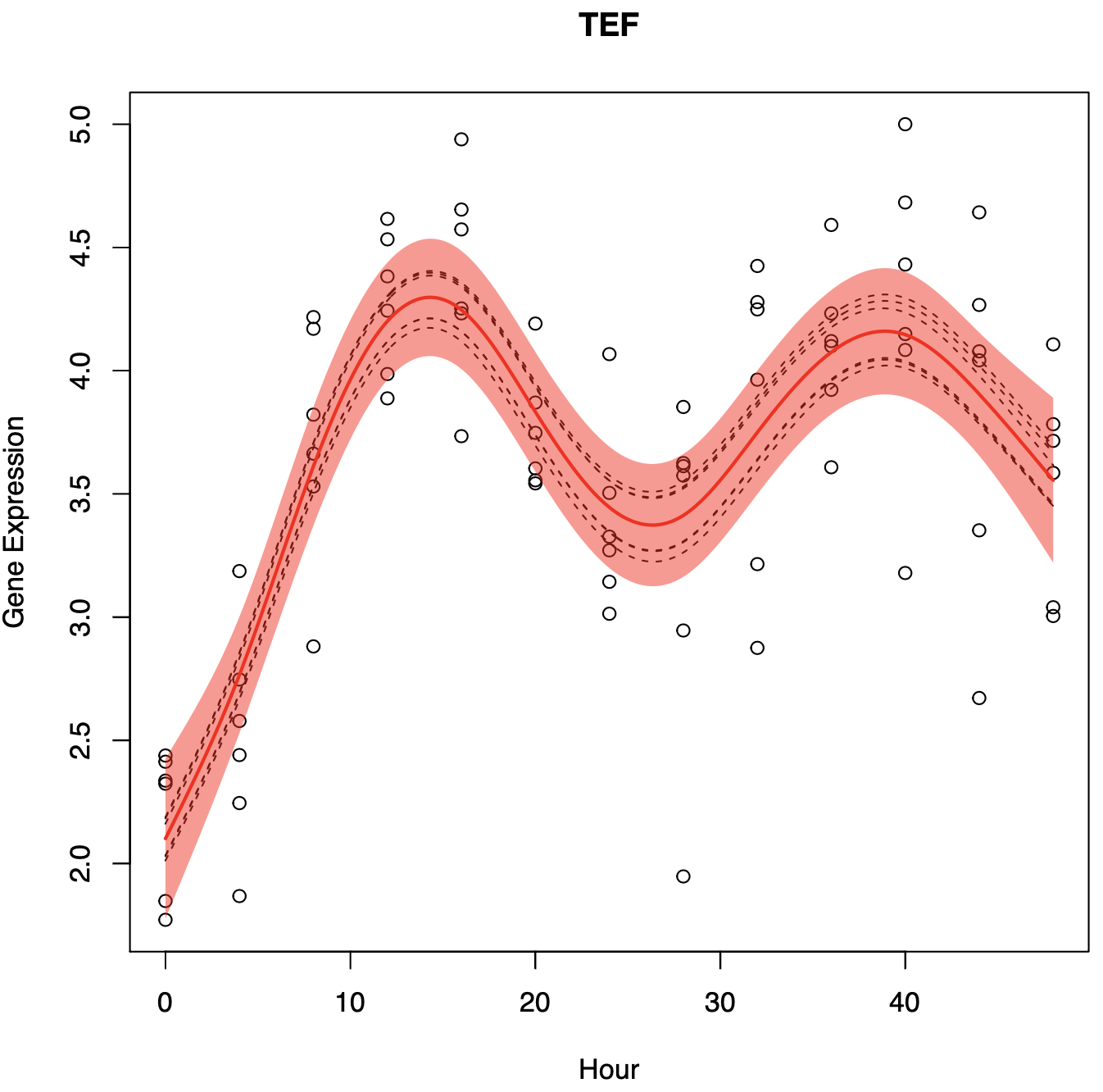

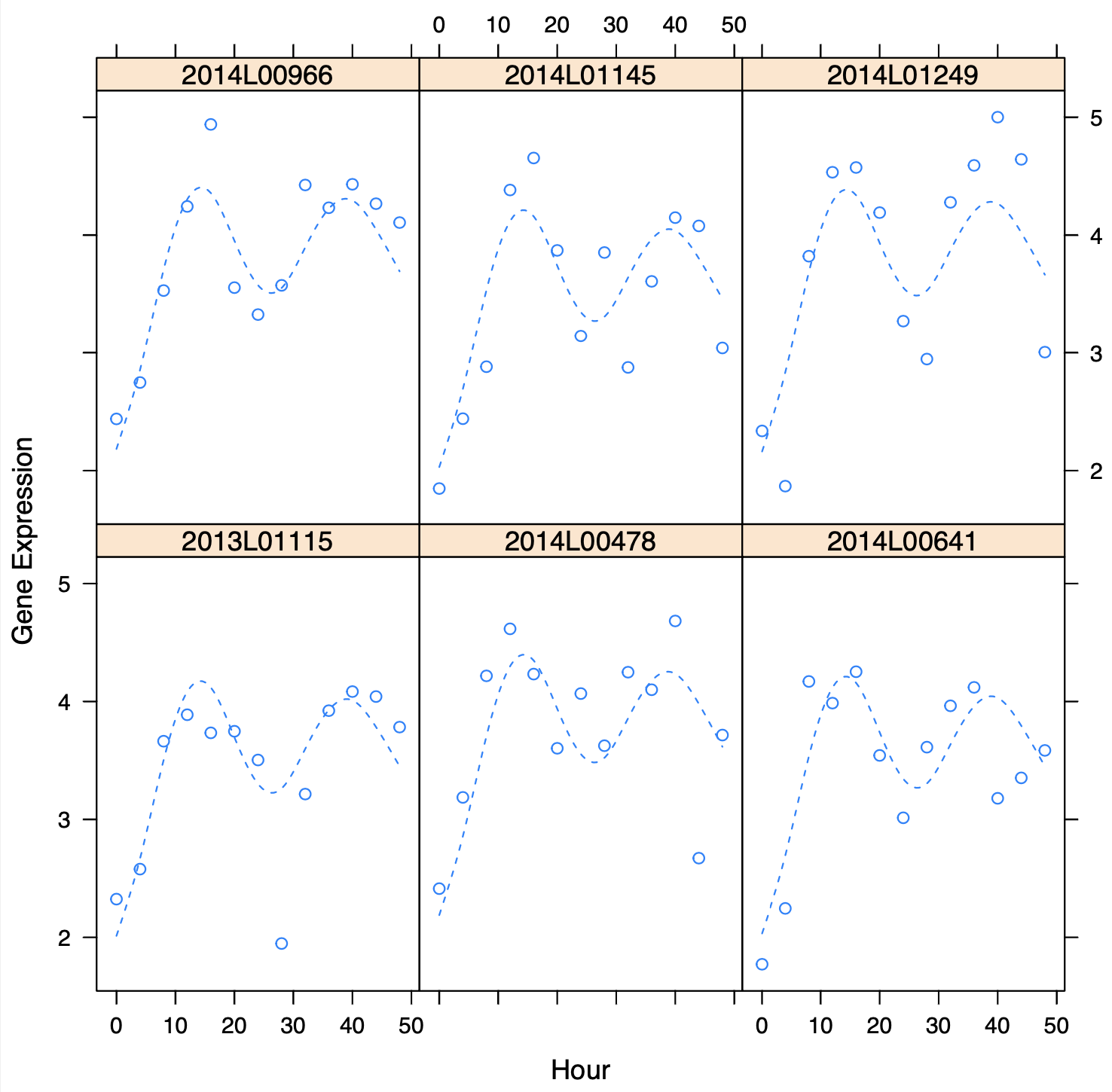


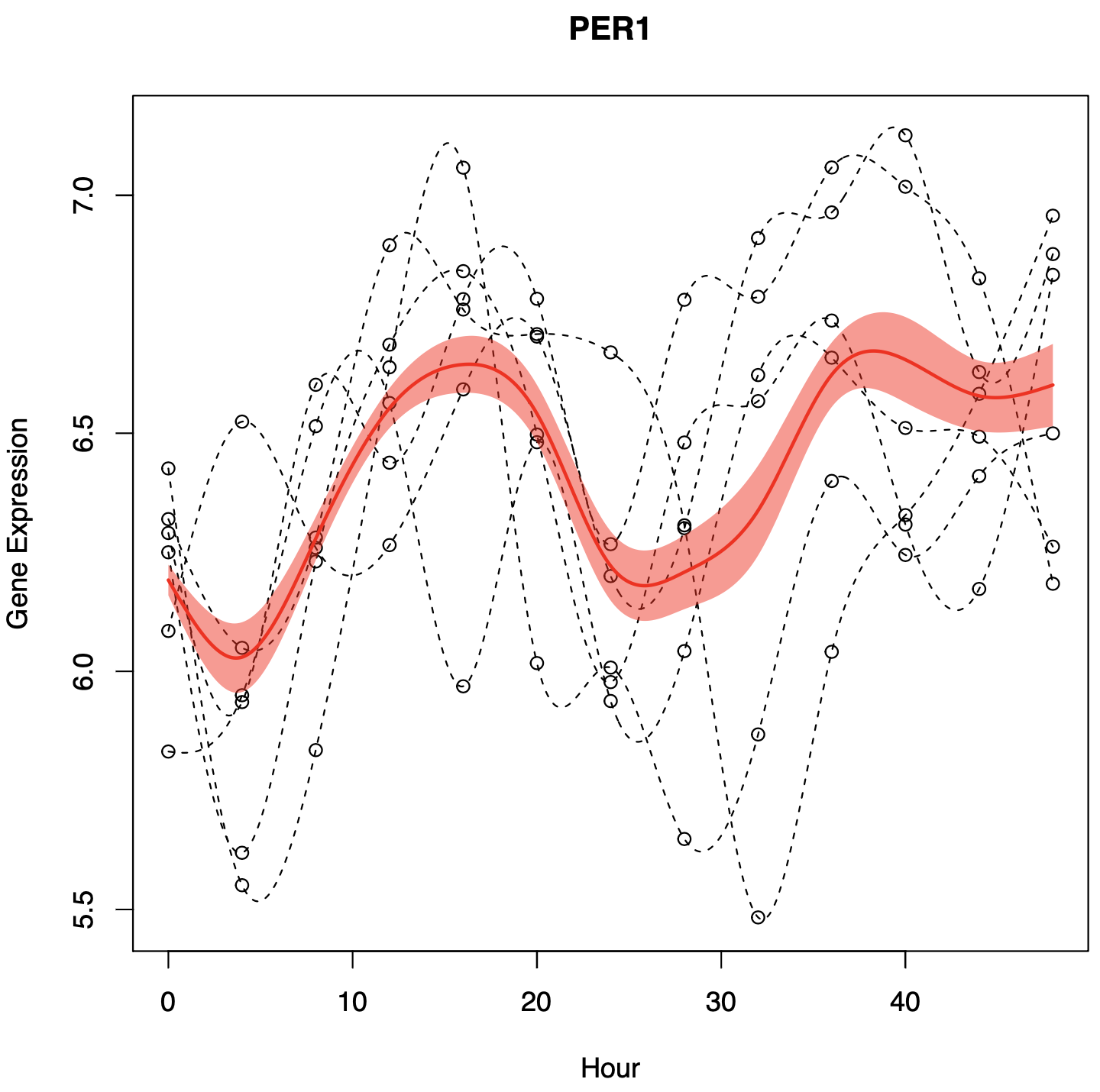

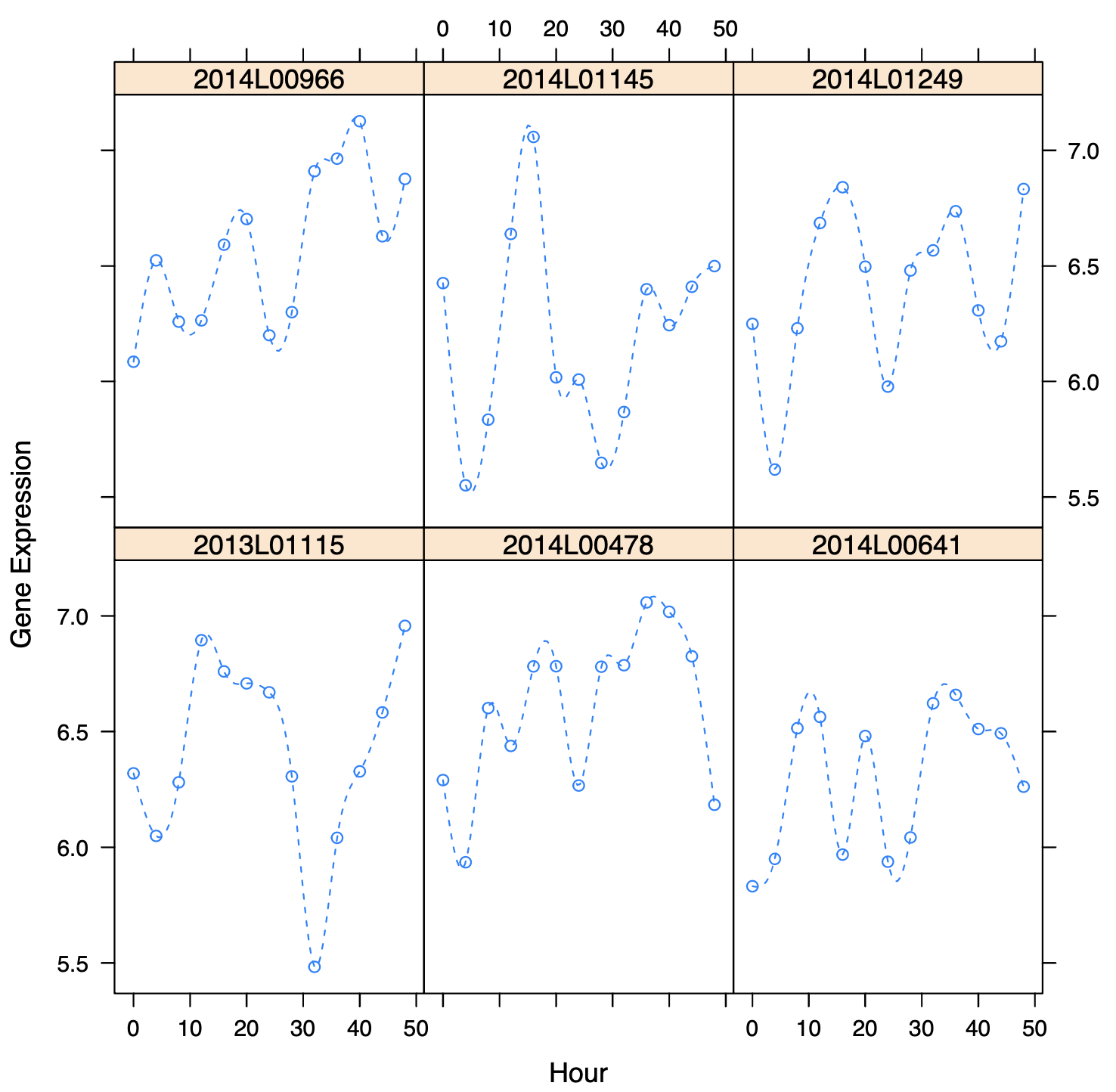


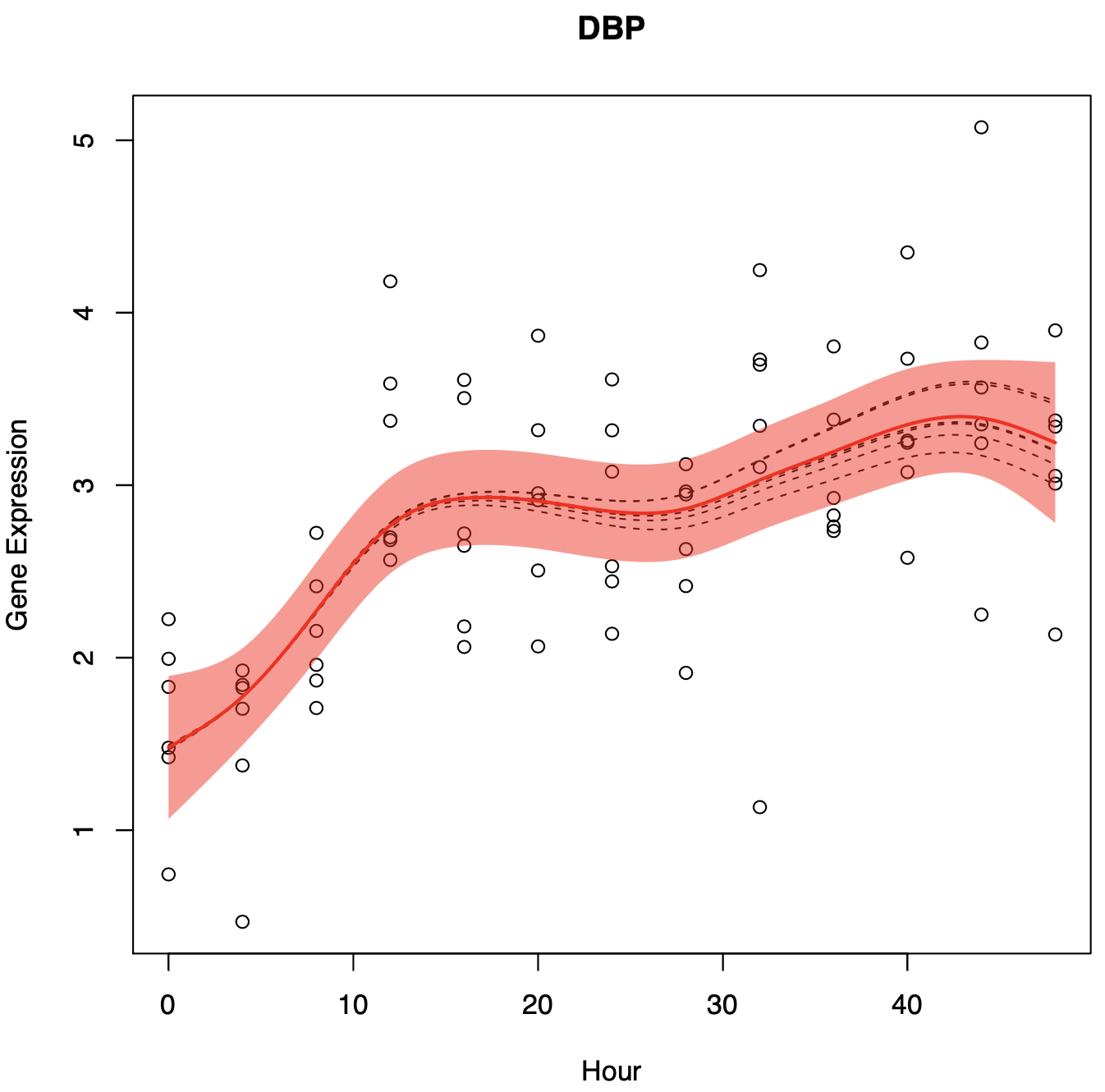

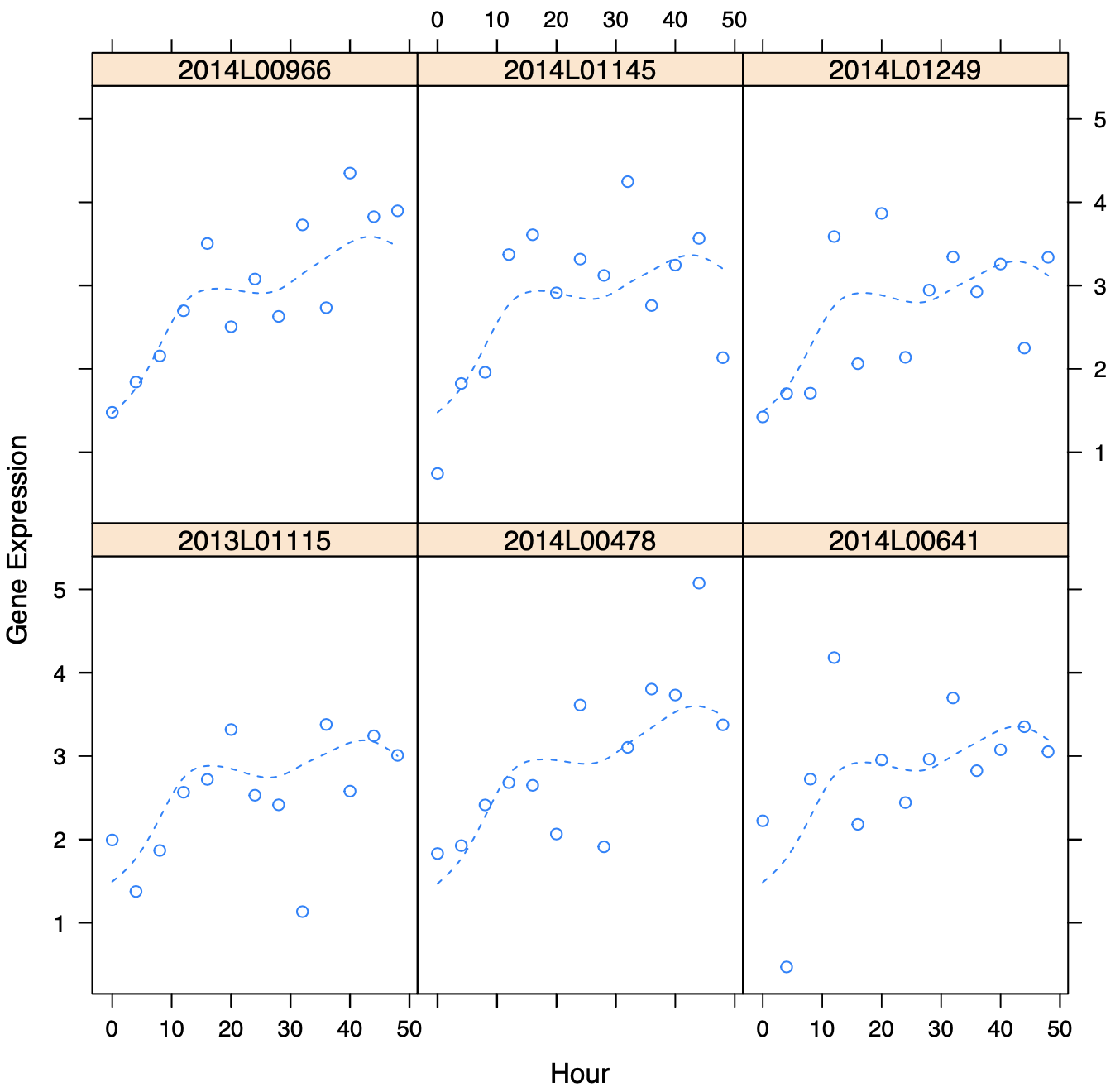


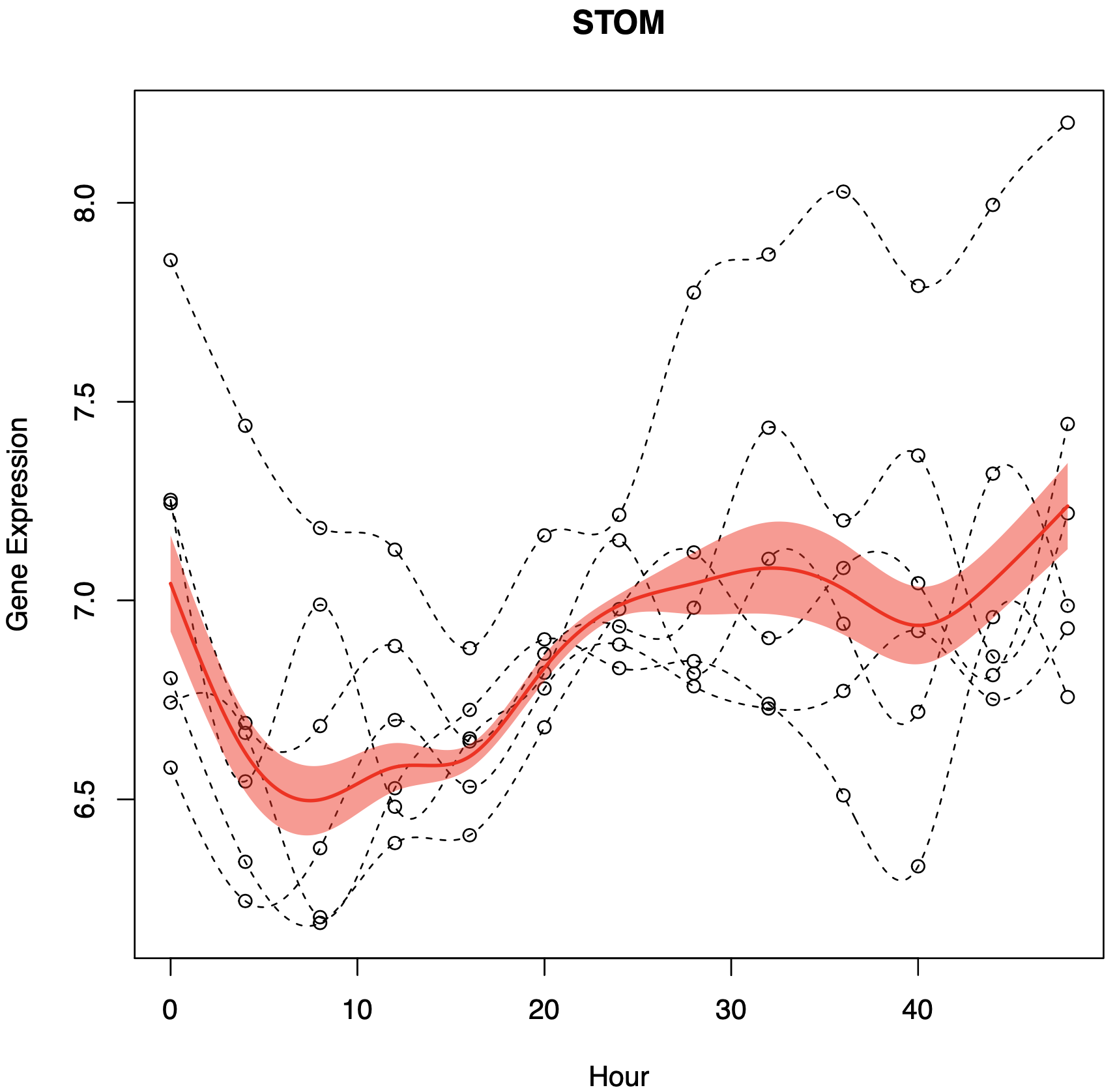

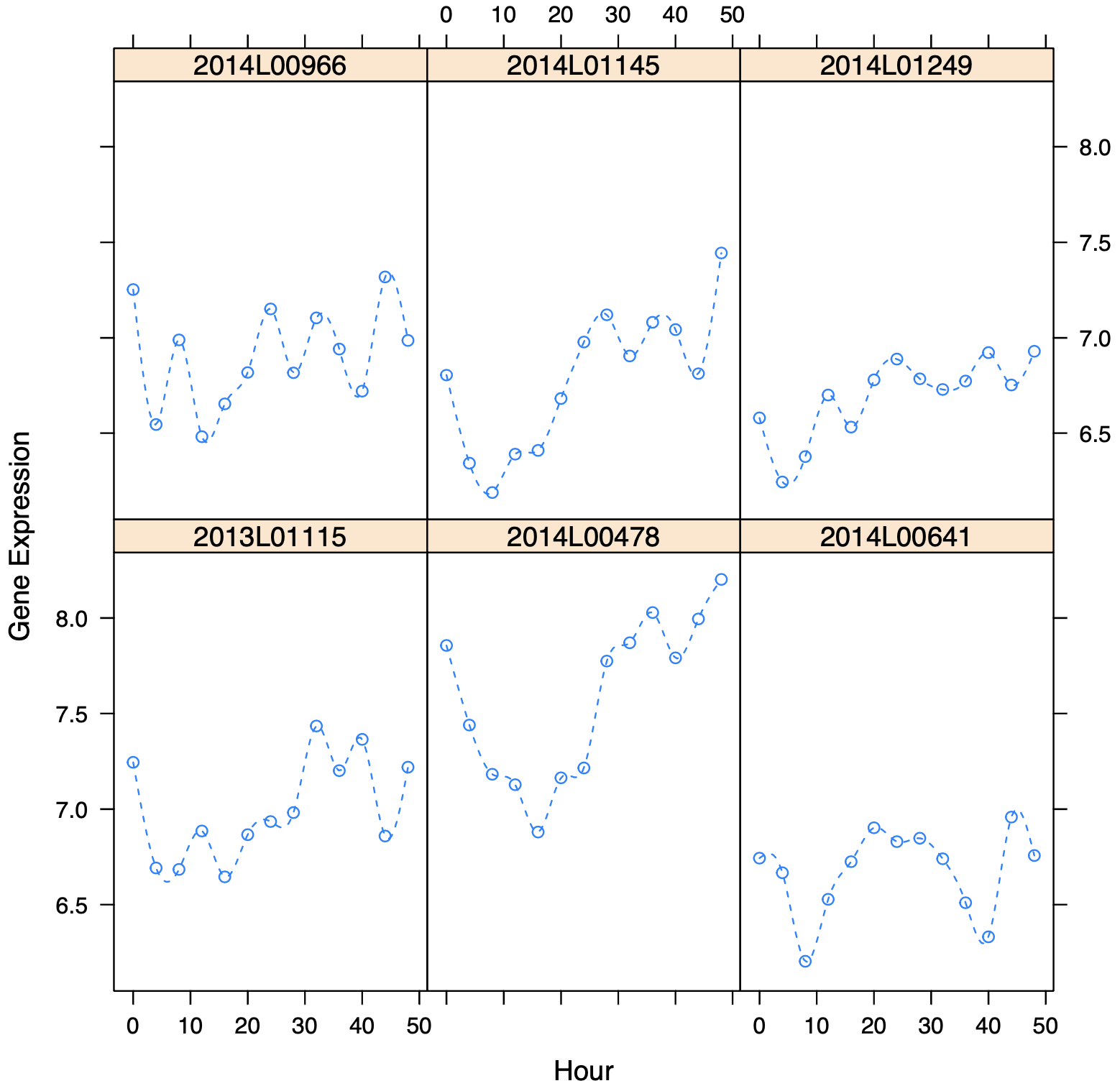


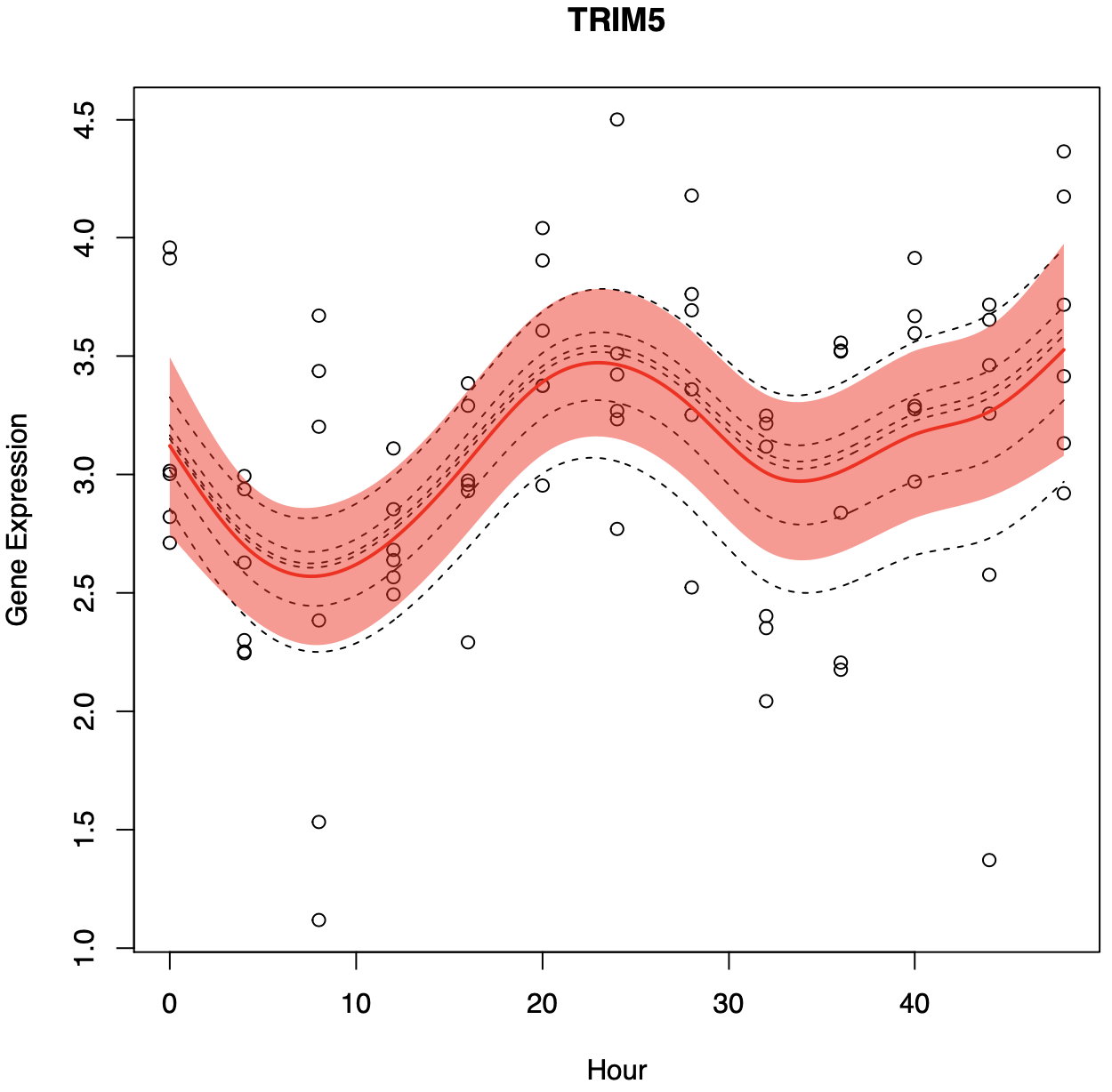

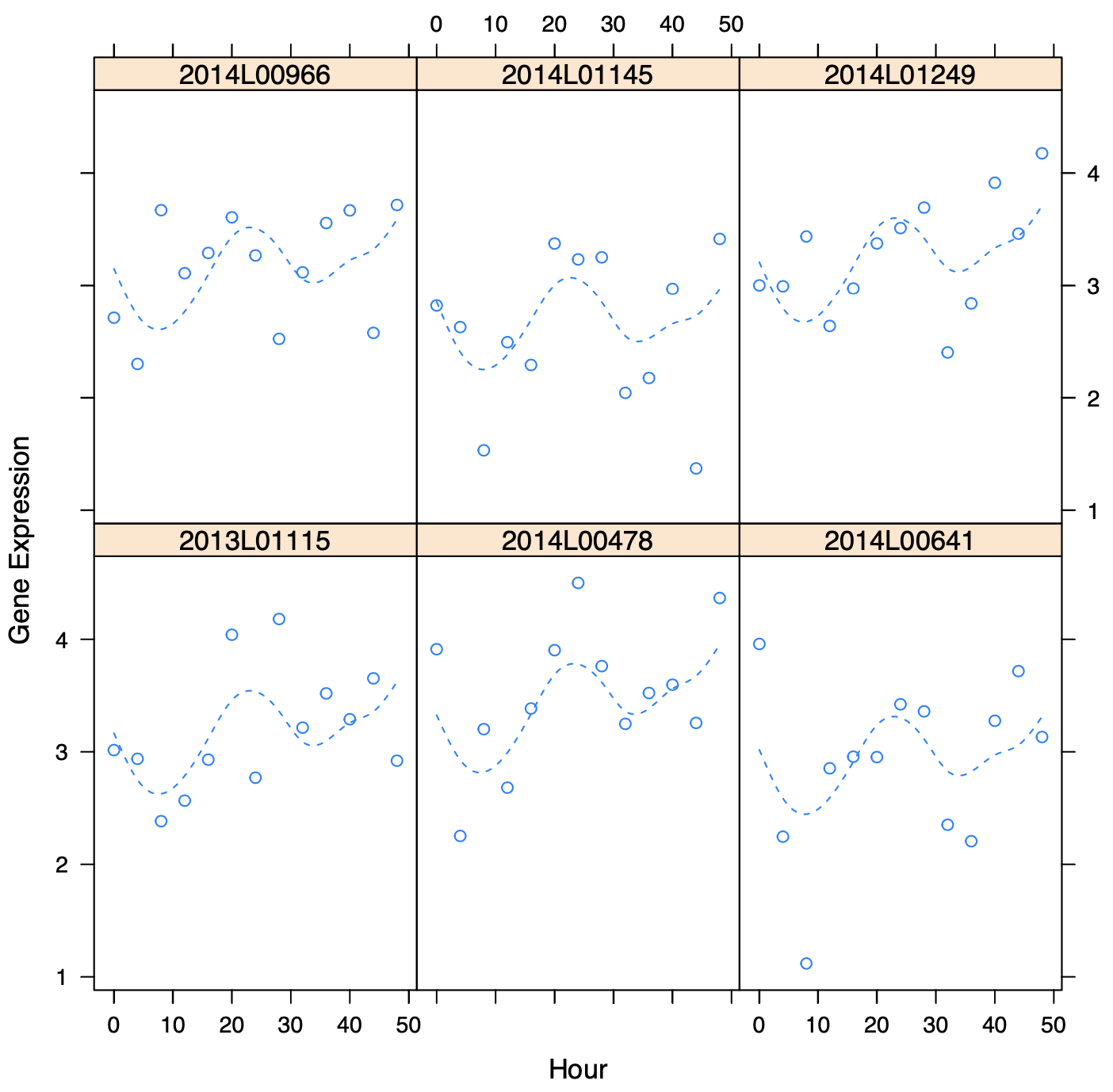


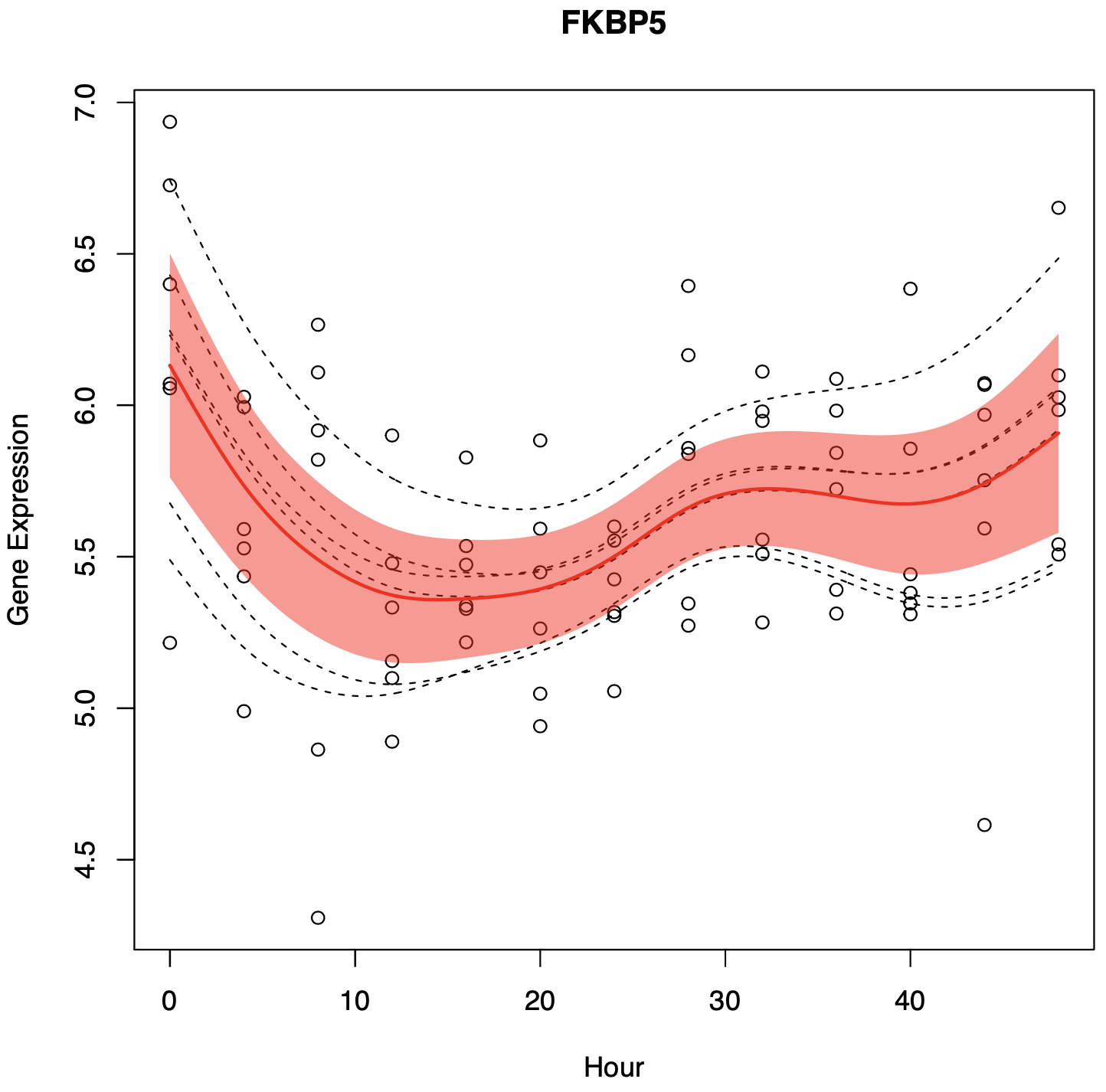

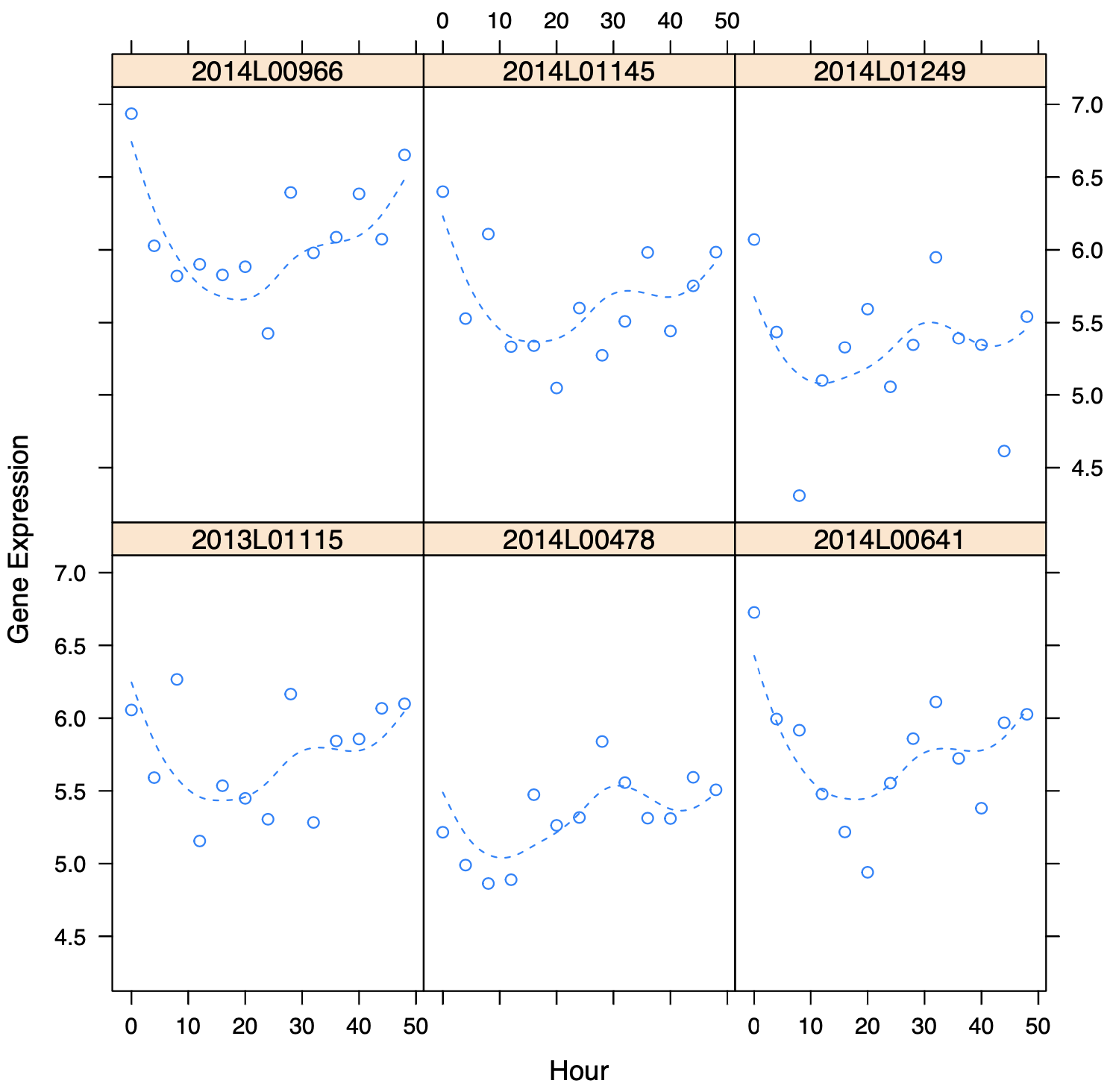


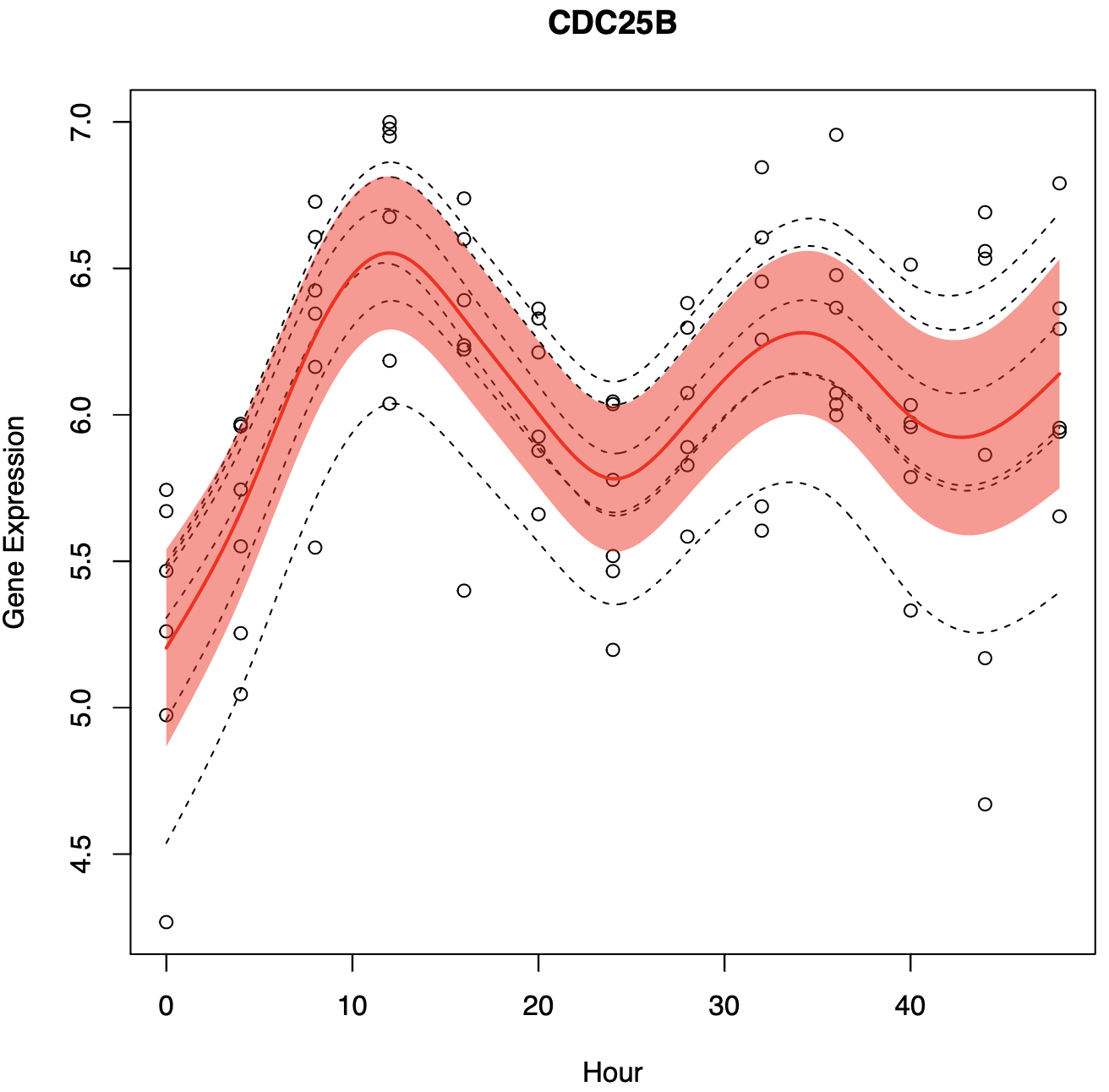

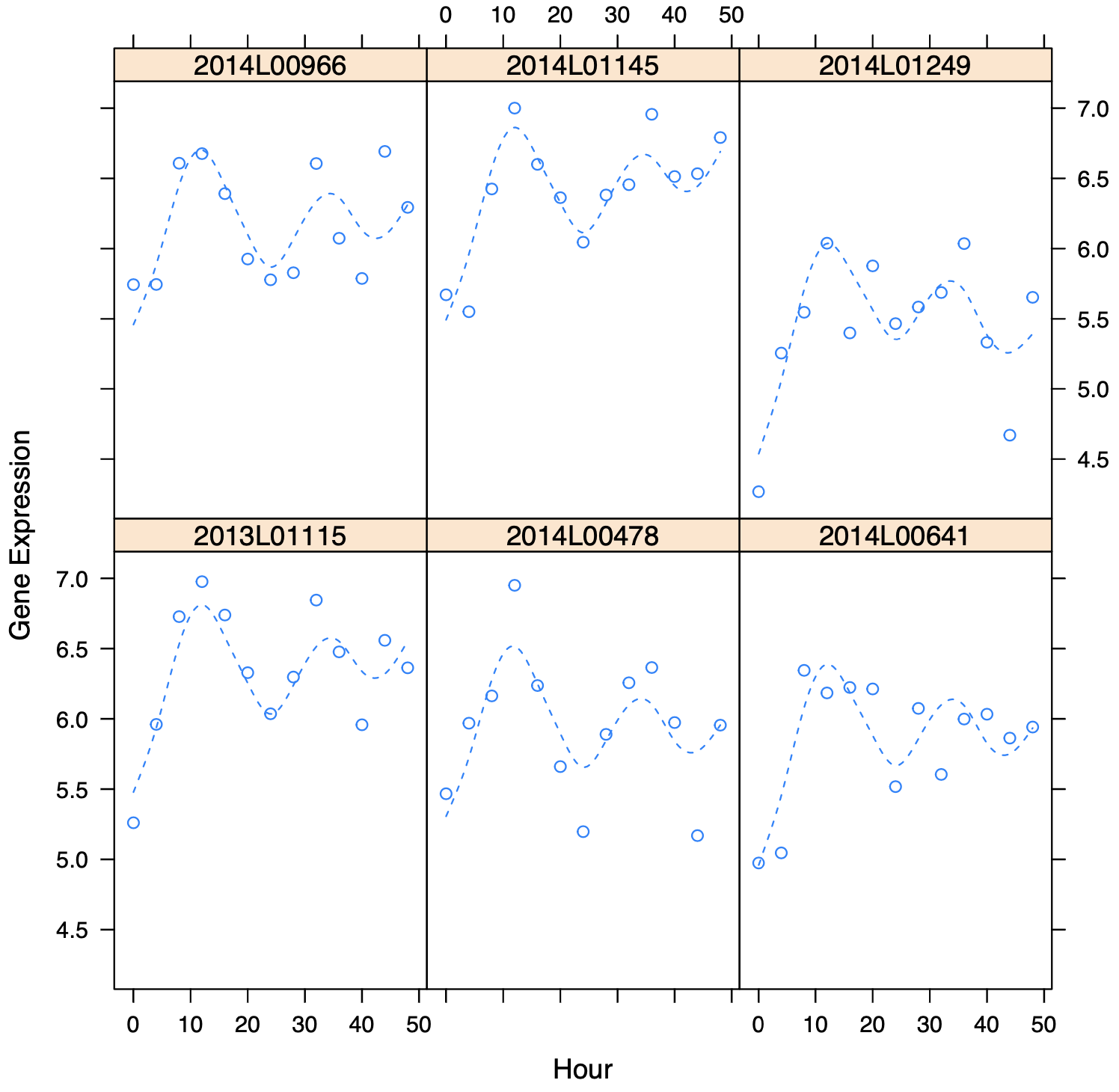


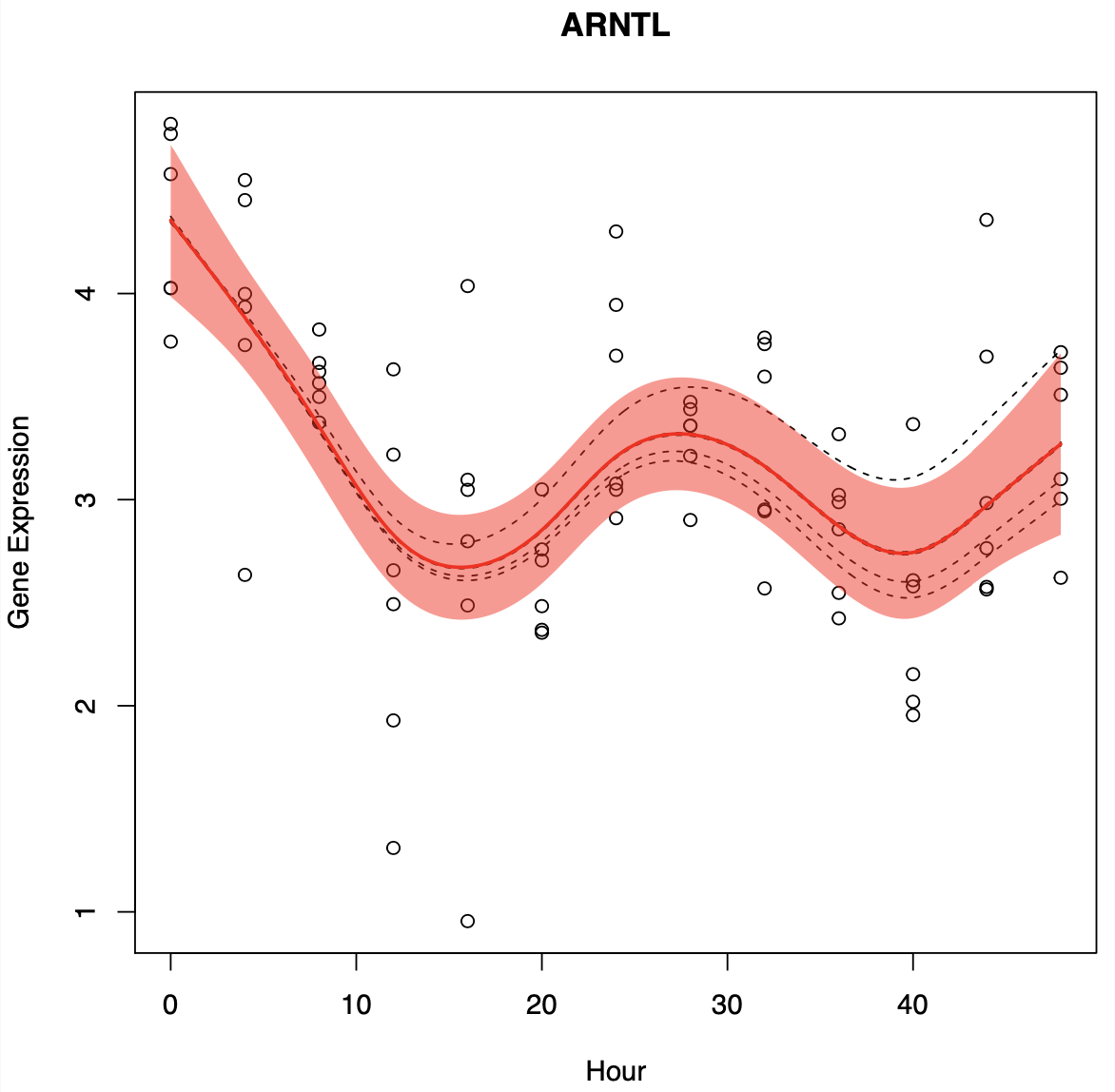

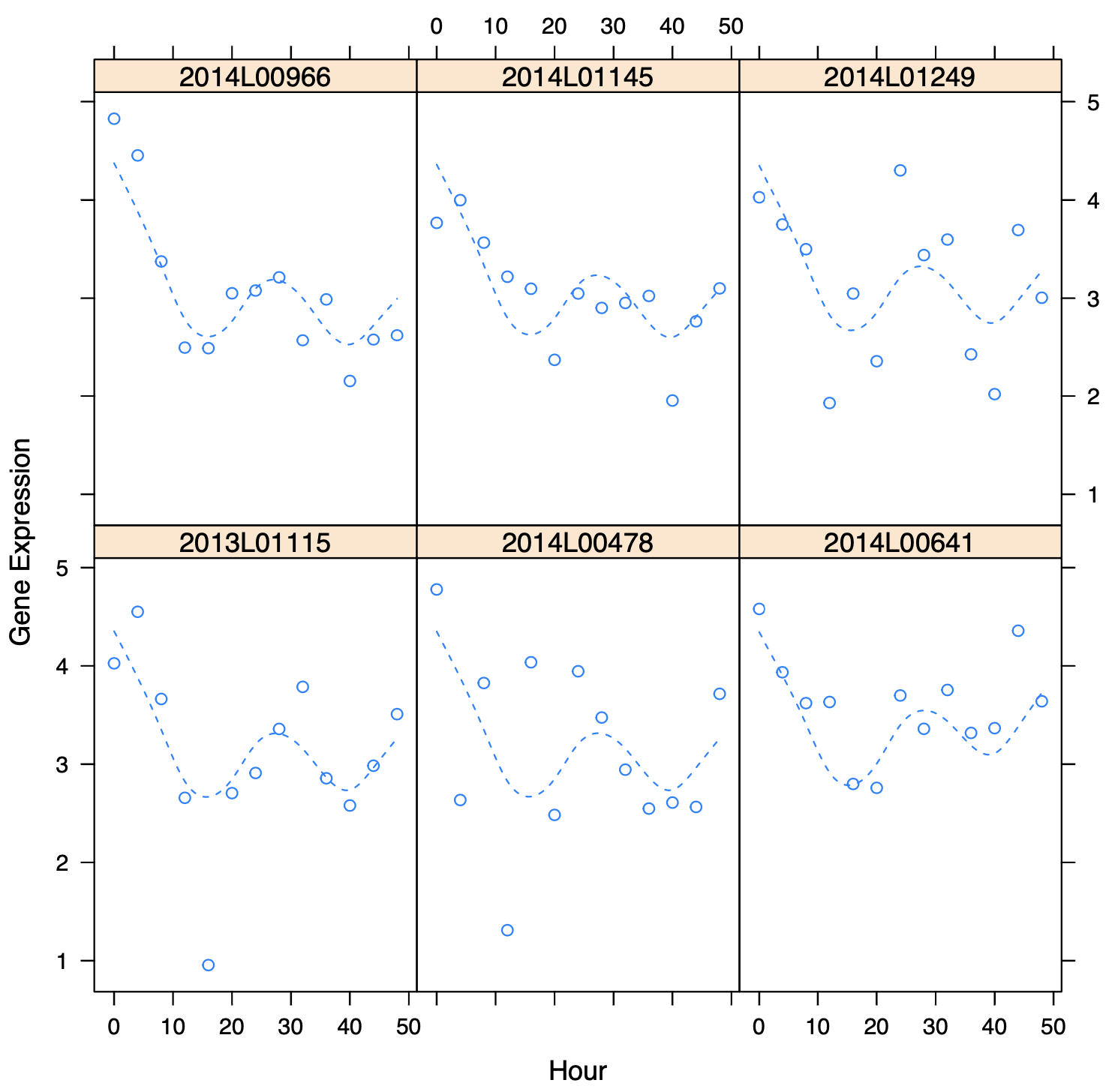


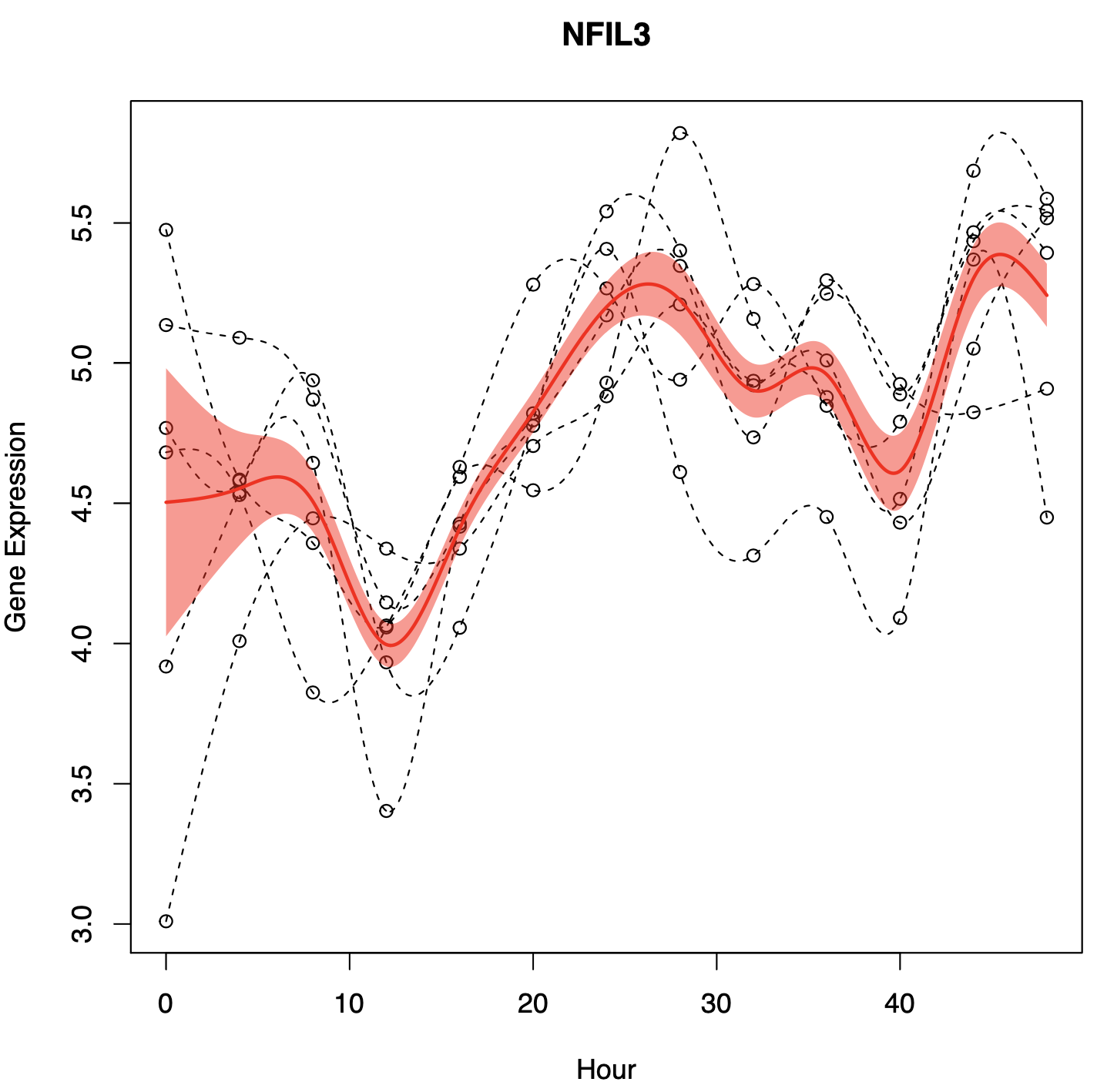

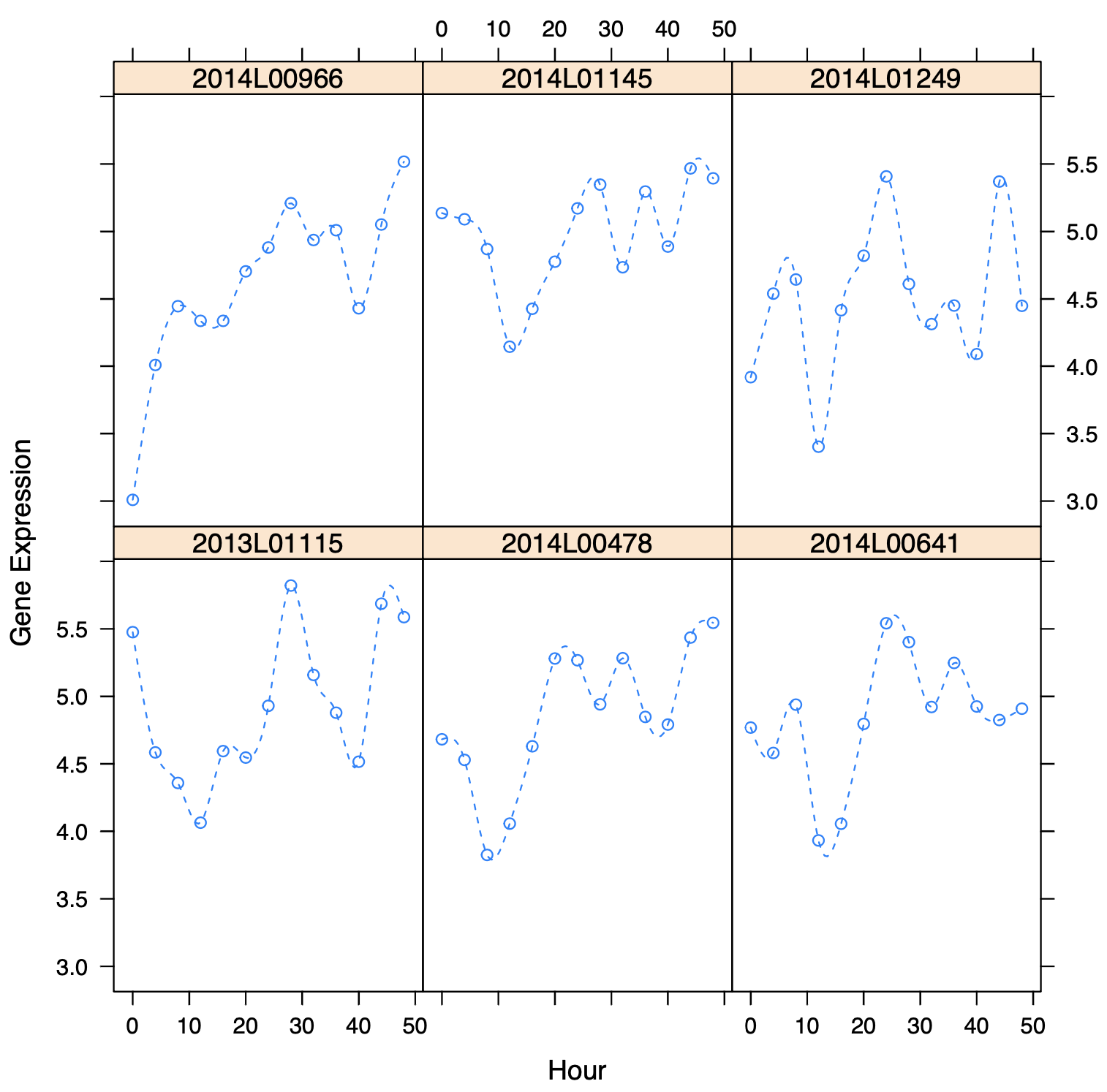


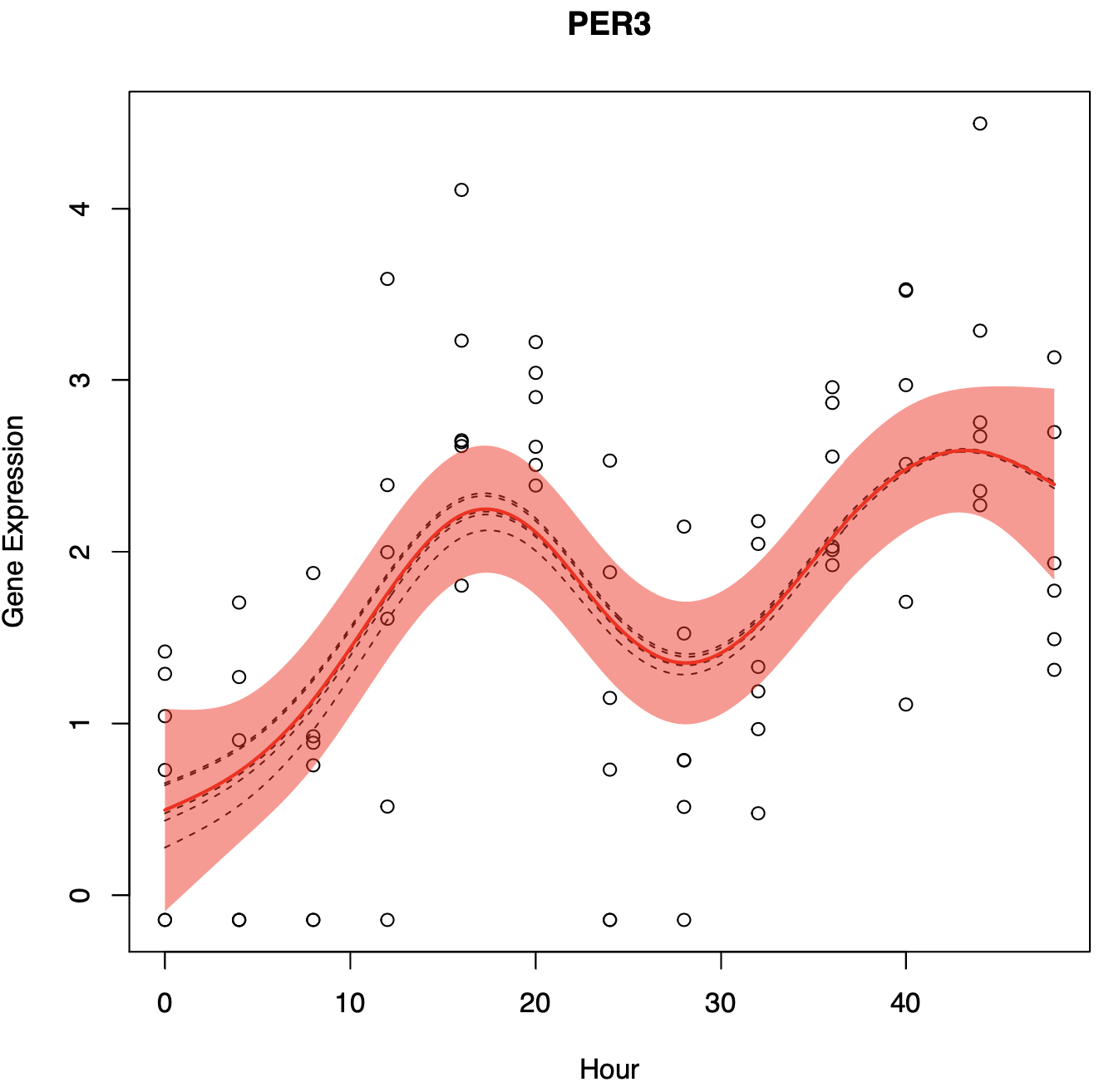

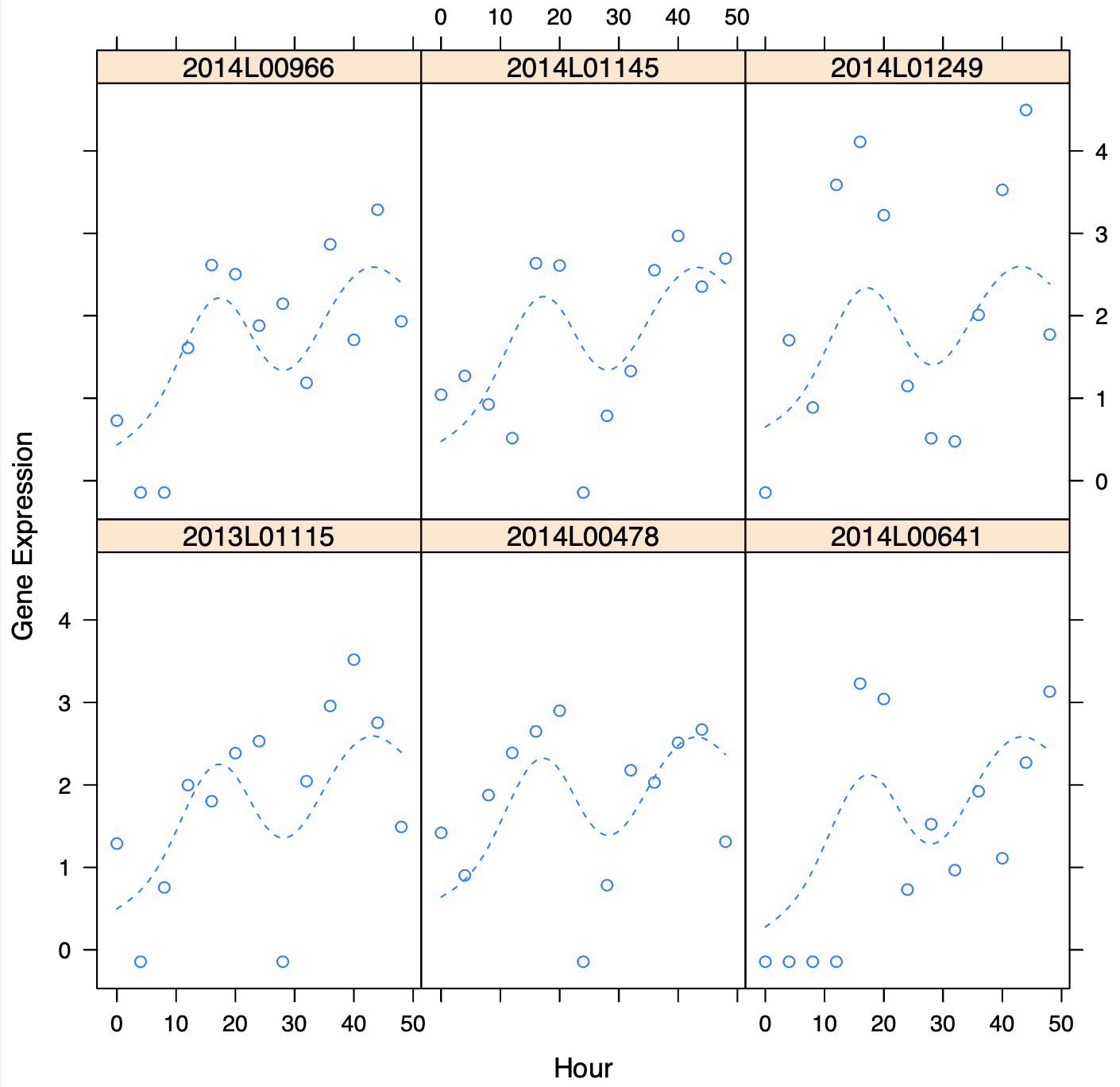


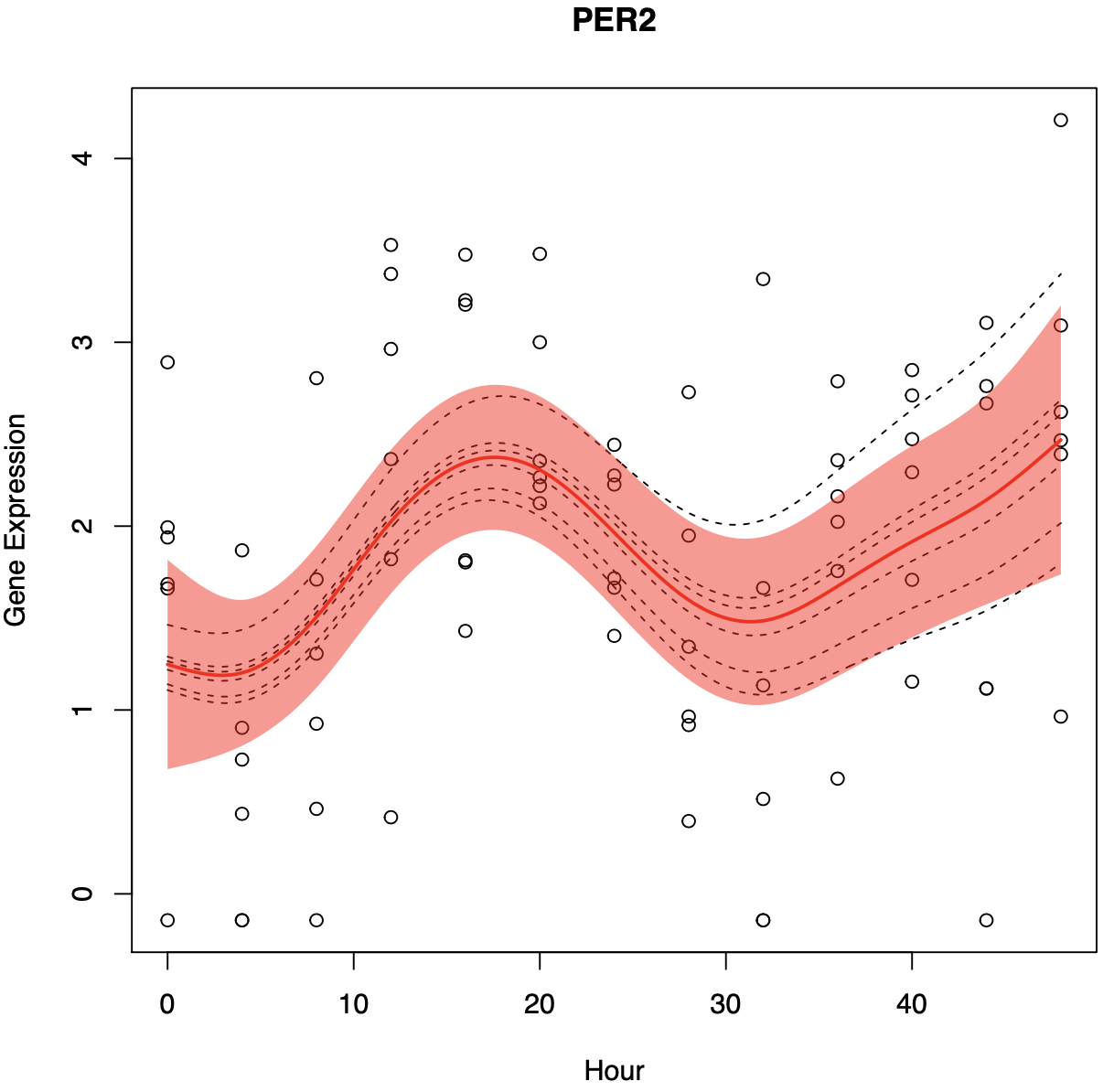

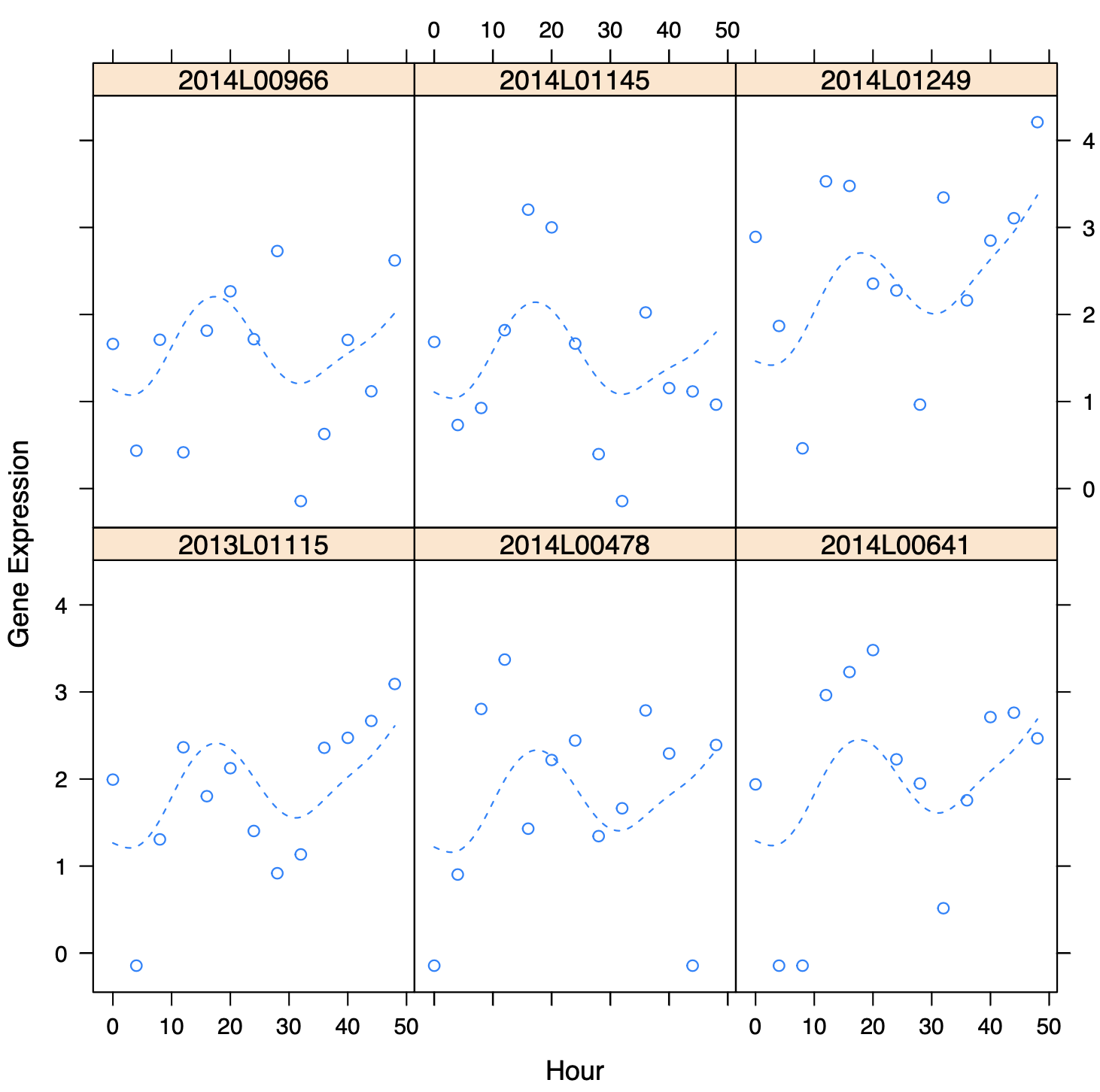


**Supplementary figure 4 description:** A, Smoothing-splines mixed-effect models of circadian gene expression across previously reported circadian genes in skin. Red line indicates the average fitted model across cell lines, with the red area representing a 95% confidence interval.

**Supplementary figure 5 Heat map of dynamic time clustering results for 9 circadian genes
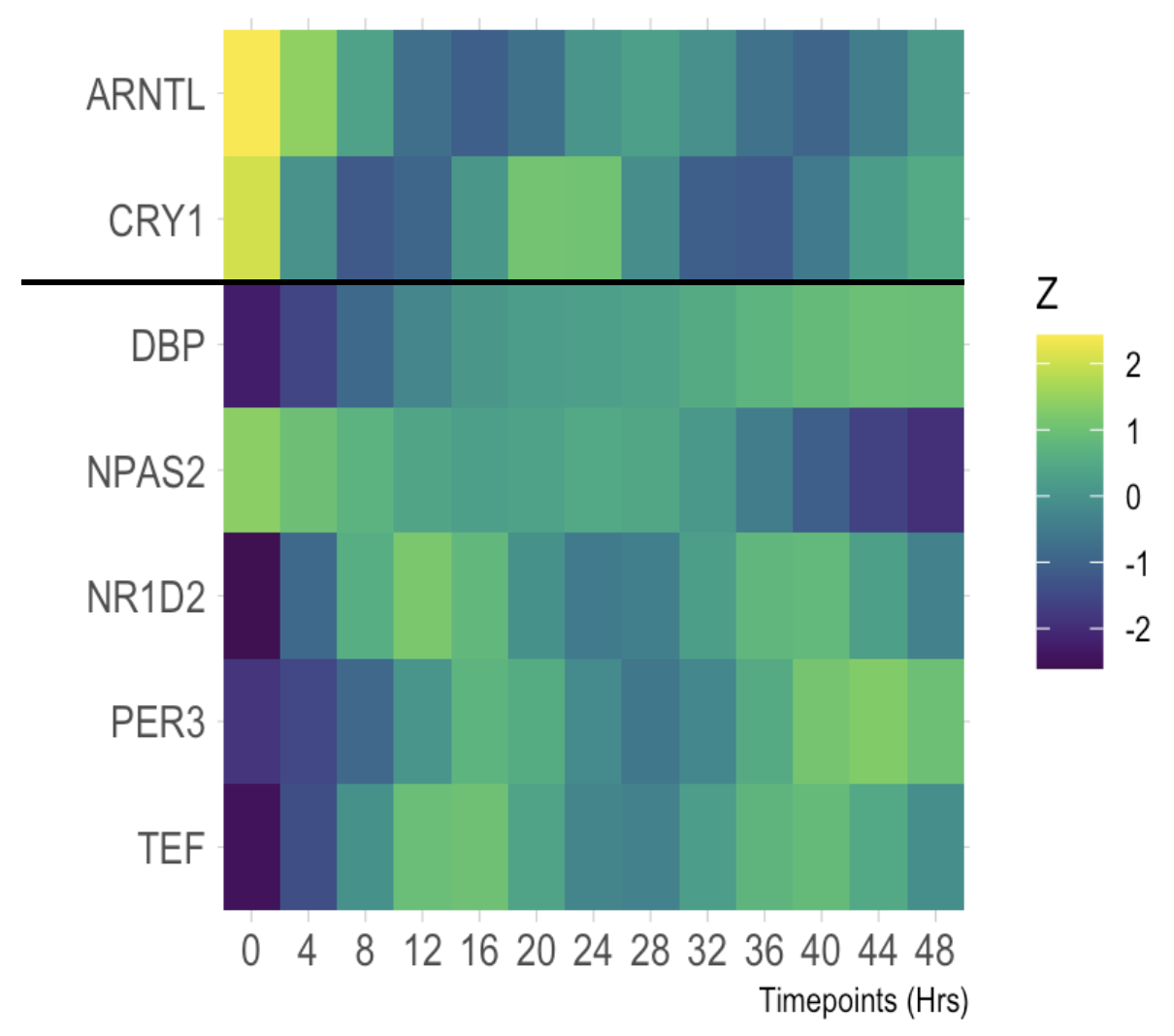
**

**Supplementary figure 5 description:** Dynamic clustering results for the 9 circadian core clock genes with consistent expression across cell lines. The black line separates the 2 clusters. Expression values are presented as Z-scores.

**Supplemental figure 6** Known interactions between circadian genes present identified in this dataset
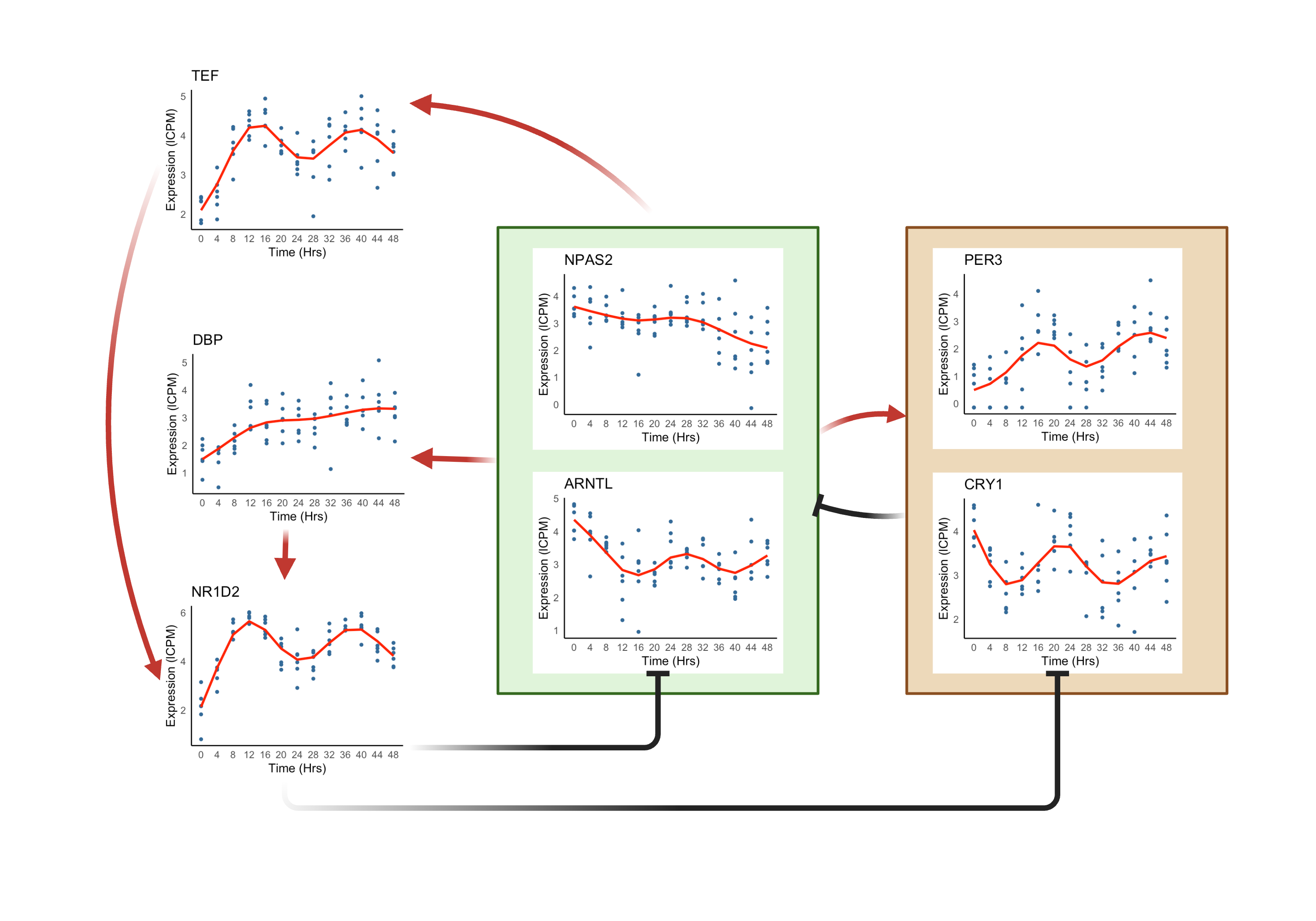


**Supplementary figure 6 description**: Known gene expression relationships of core circadian genes. Red arrows indicate a gene inducing in the expression of another gene. The black lines and bar indicates a gene repressing the expression of another gene.

**Supplementary Figure 7 Genomic annotation of the consensus peak regions and selected time significant regions**

1. All consensus regions


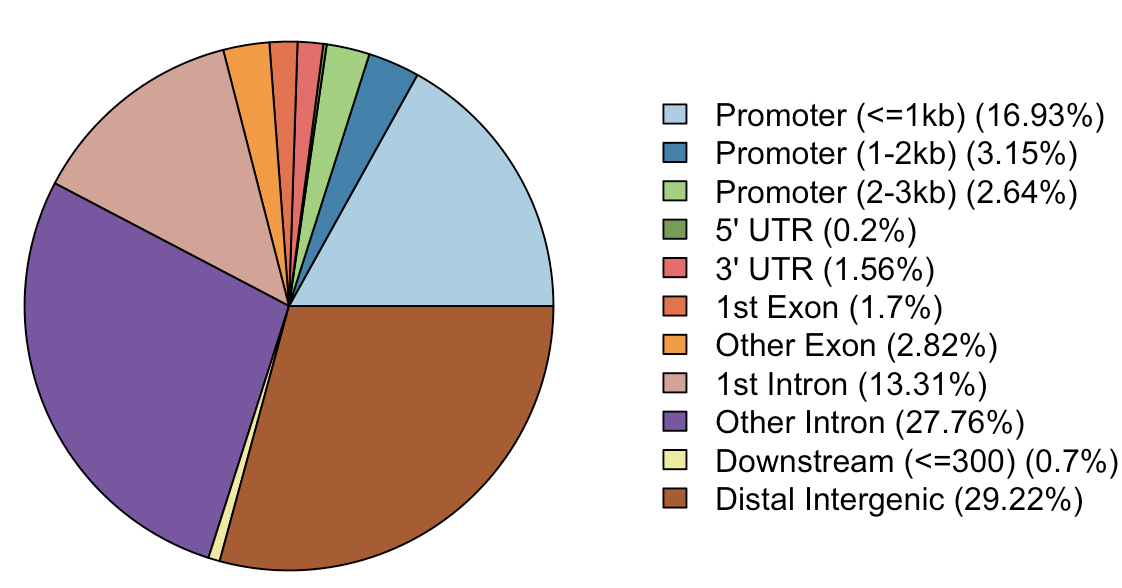


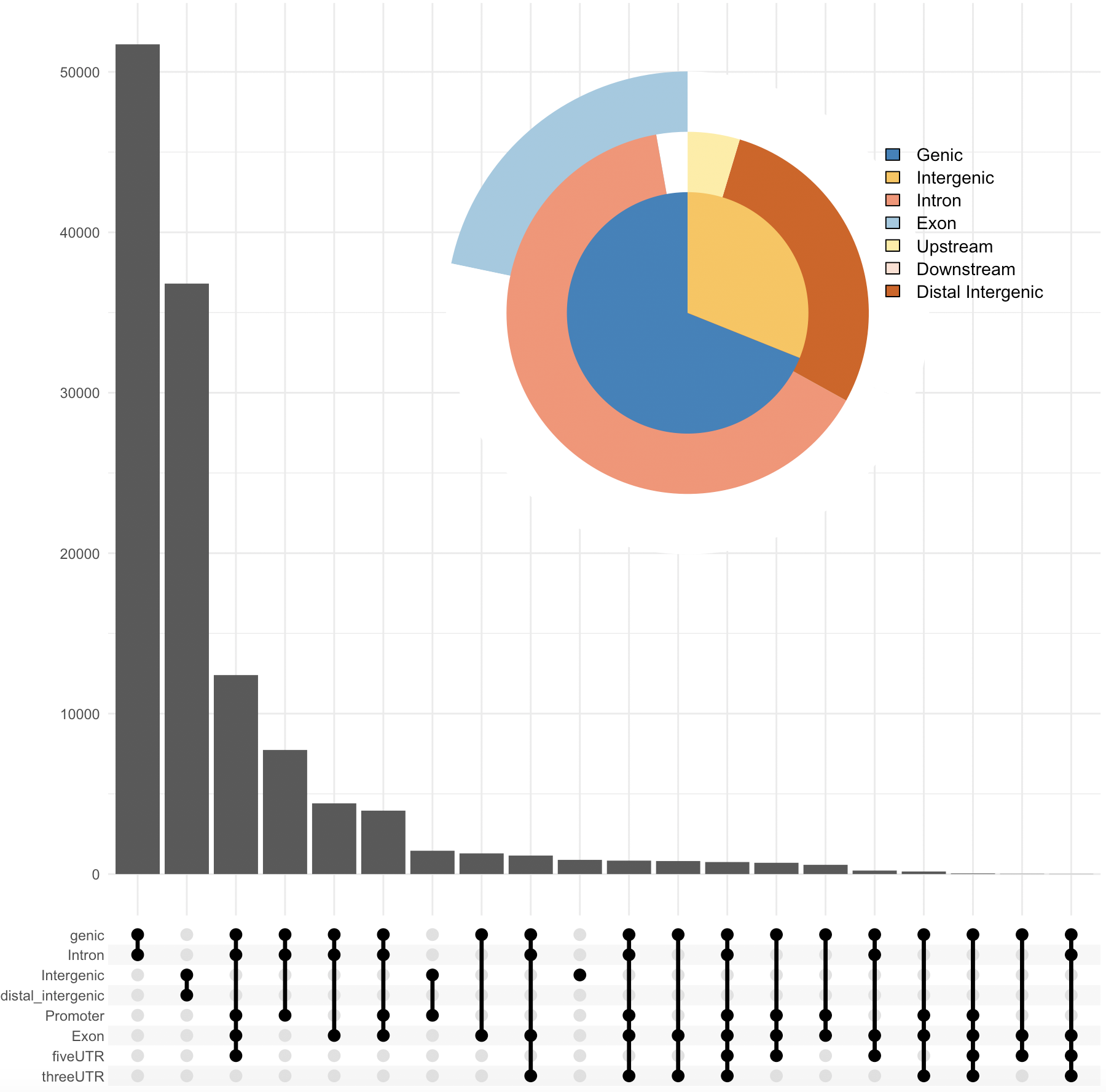


1. Time significant regions


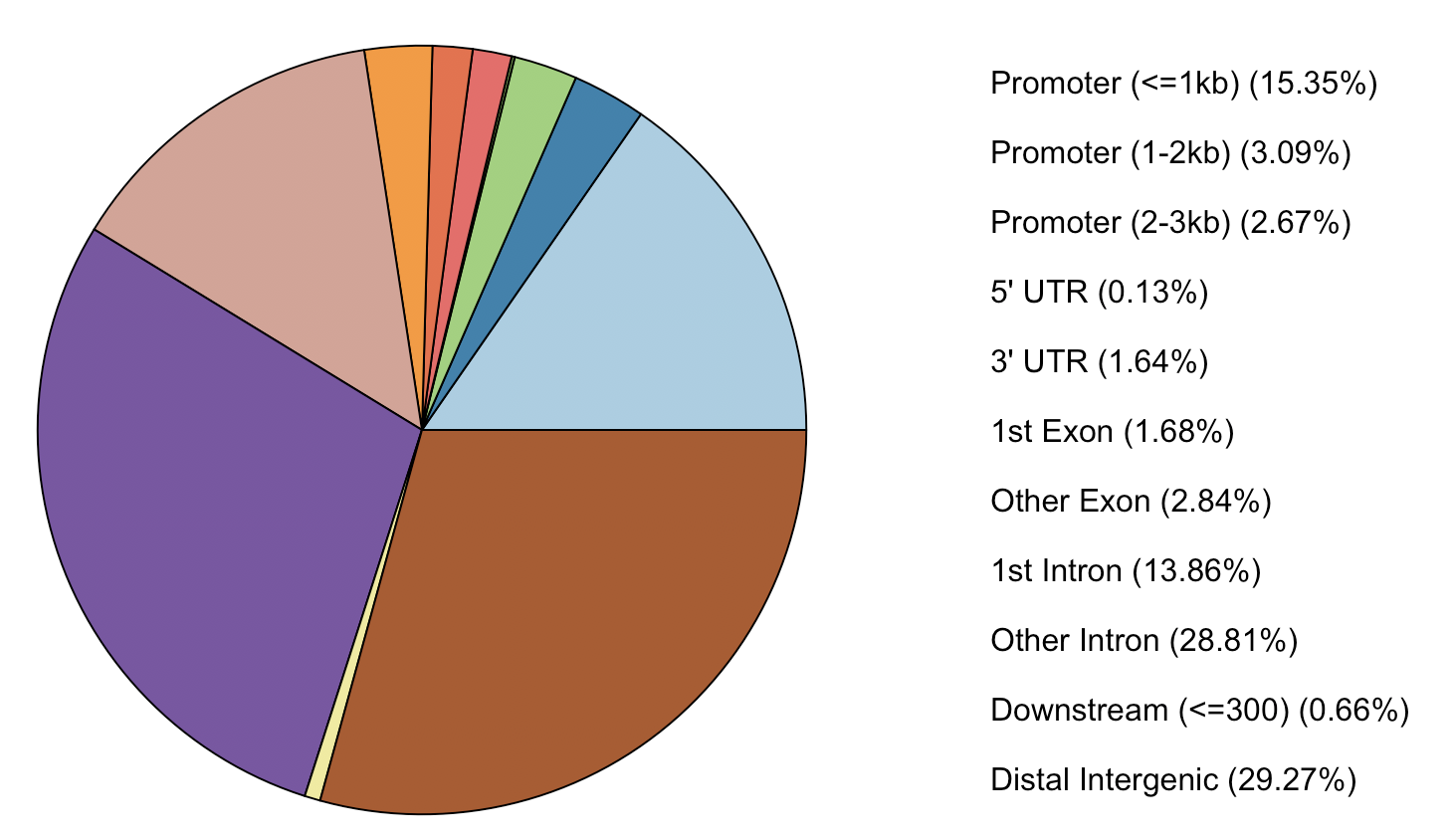


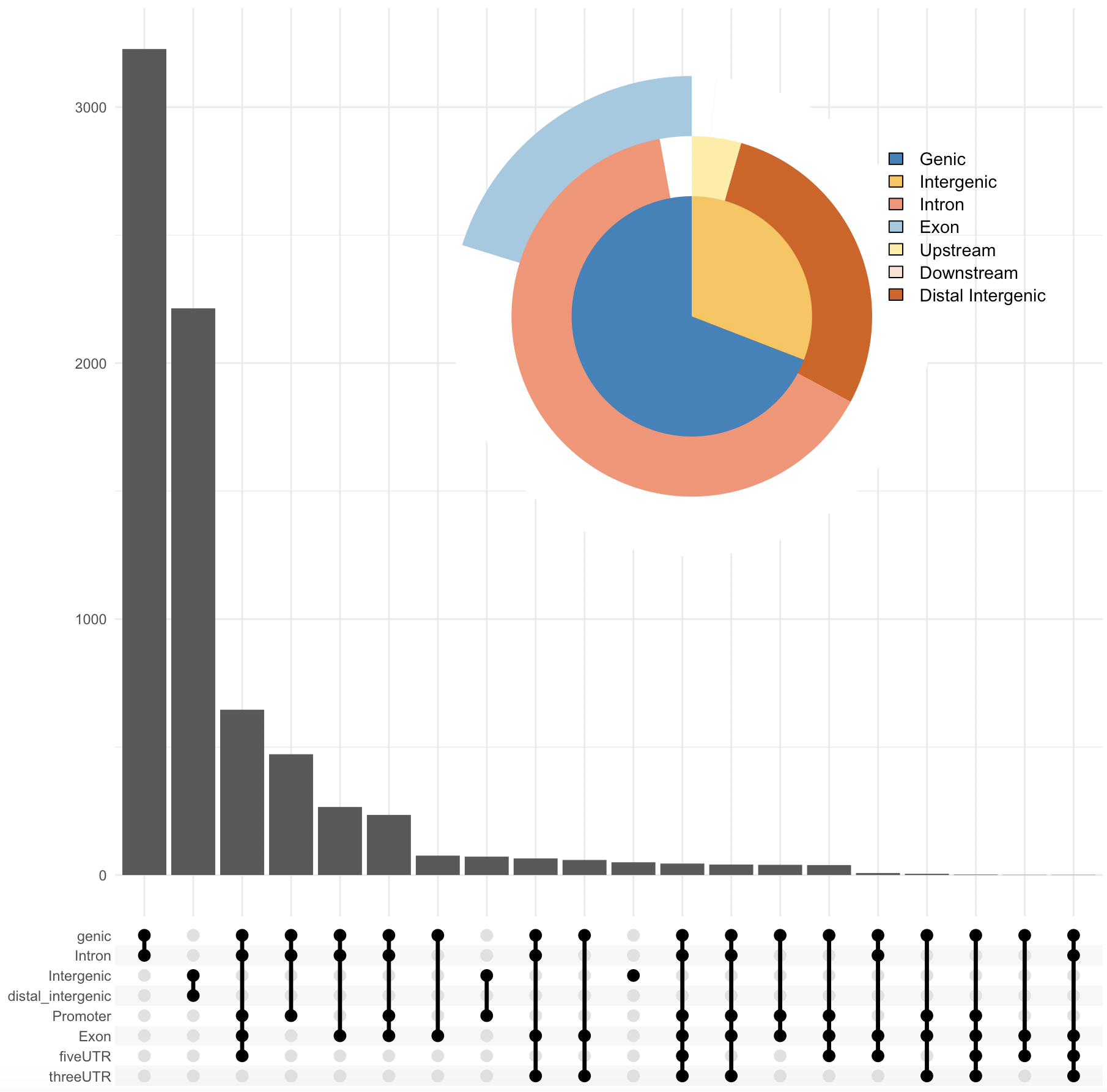


**Supplementary Figure 7 description:** Chipseeker annotations for peak regions. A. Genomic annotations for all n=126,057 consensus peak regions. B. Genomic annotations for the n=7,568 peaks that had a significant change over time in their accessibility.

**Supplemental Figure 8 Eigengene values for ATAC-seq modules obtained from WGCNA
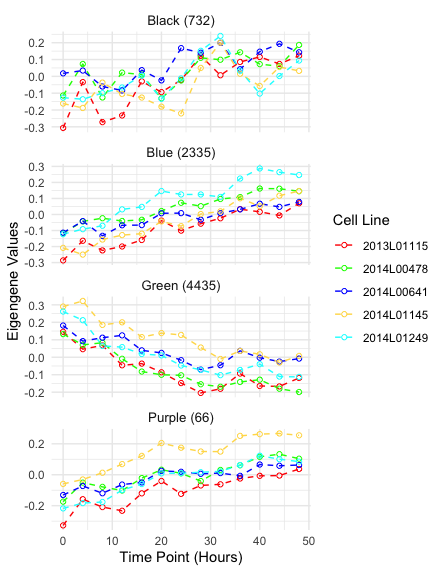
**

​​

**Supplemental Figure 8 Description:** Eigengene modules from WGCNA of the longitudinal chromatin accessibility patterns of ATAC-seq data collected every 4 hours for a 48 hour period. Each color represents a fibroblast cell culture from a different individual. The number of chromatin accessible regions assigned per module is indicated next to the module name.

**Supplementary figure 9 Schematic of synchronization and collection times
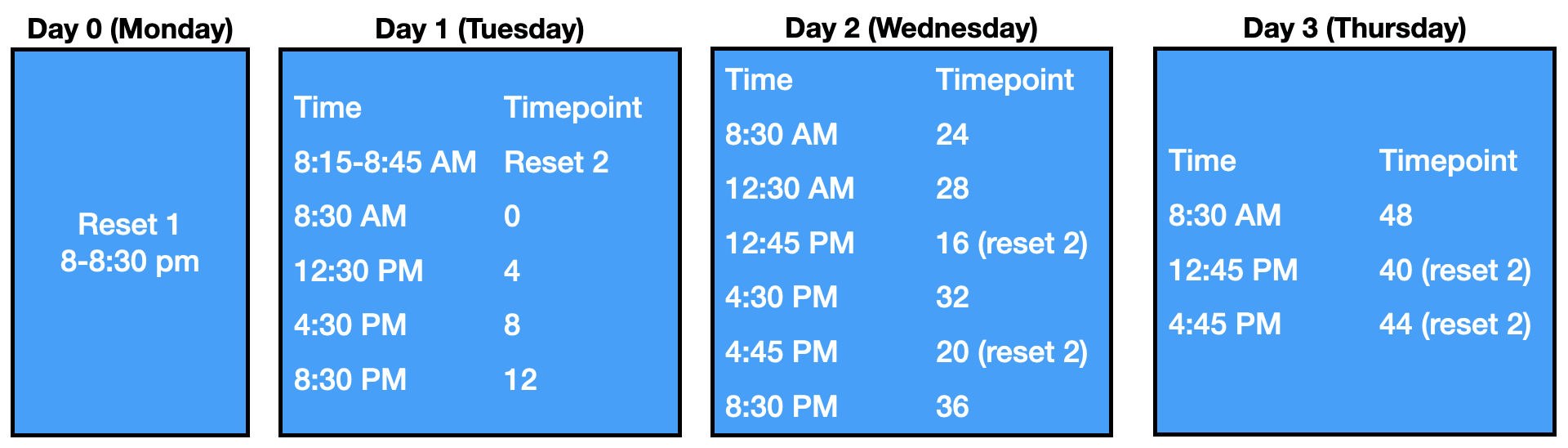
**

**Supplementary Figure 9 description:** Collection scheme for both RNA-seq and ATA-seq fibroblast cell culture samples. Cells were reset 12 hours before the first collection. In order to collect RNA or cells every 4 hours for 48 hours, cells were split into two batches, which were reset 12 hours apart.

**Supplemental Figure 10 Quality Control for ATAC-seq data**

**A**

**
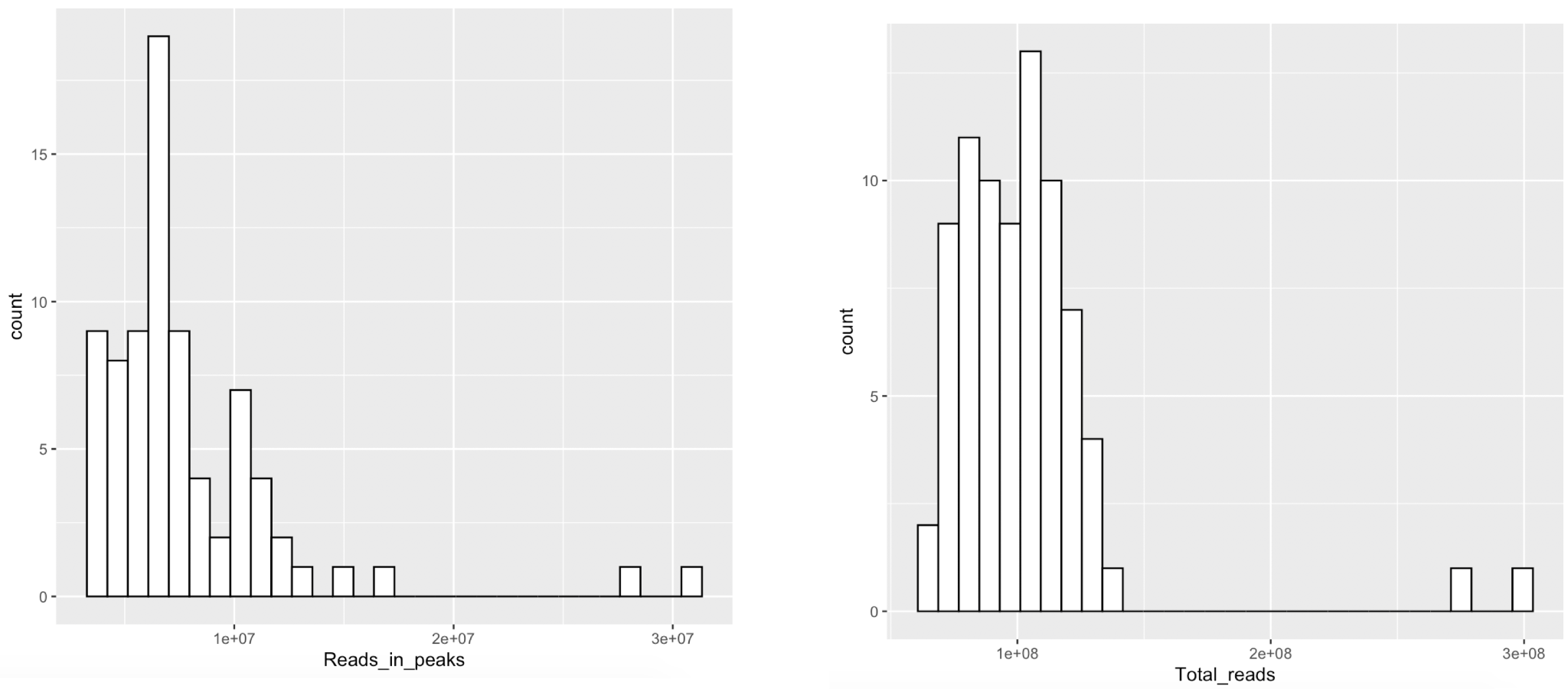
**

**B**

**
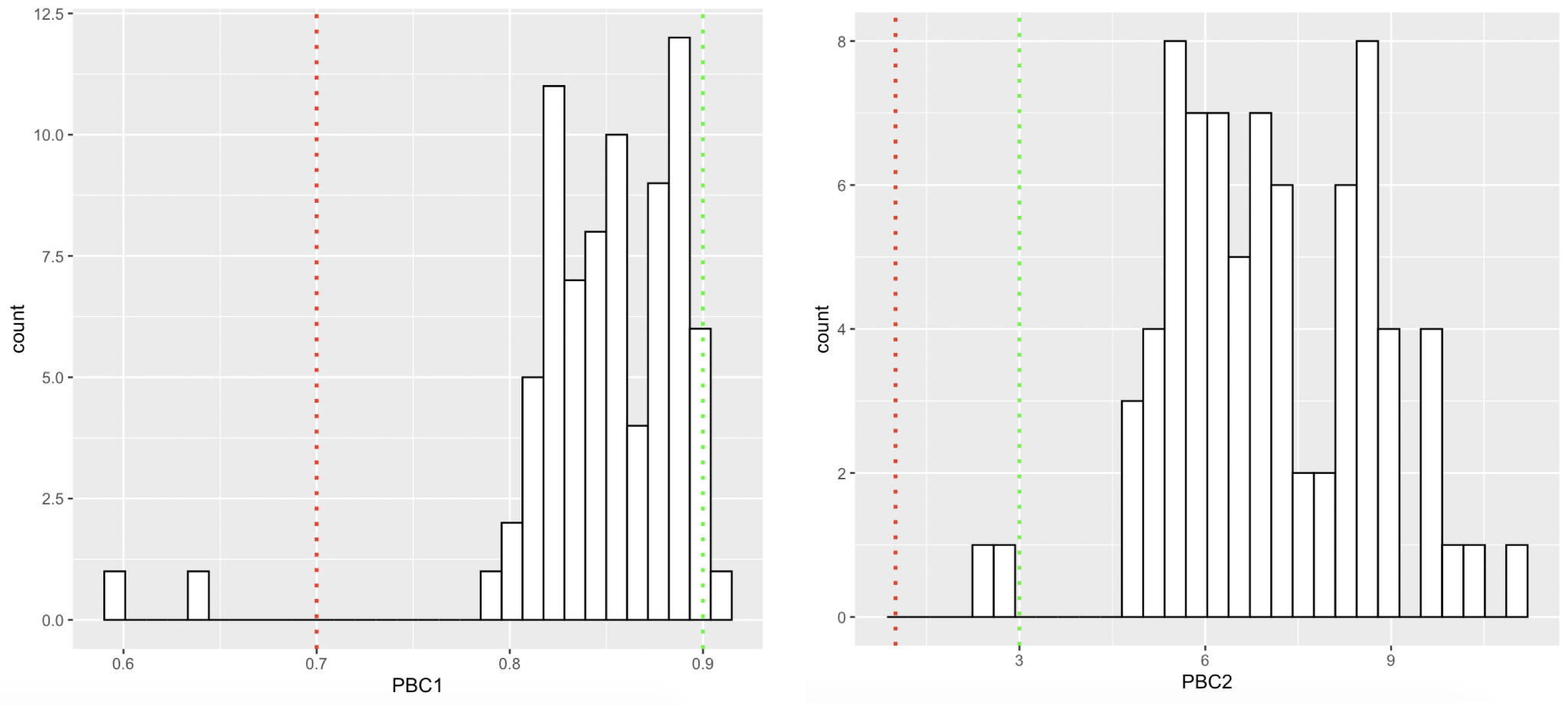
**

**C
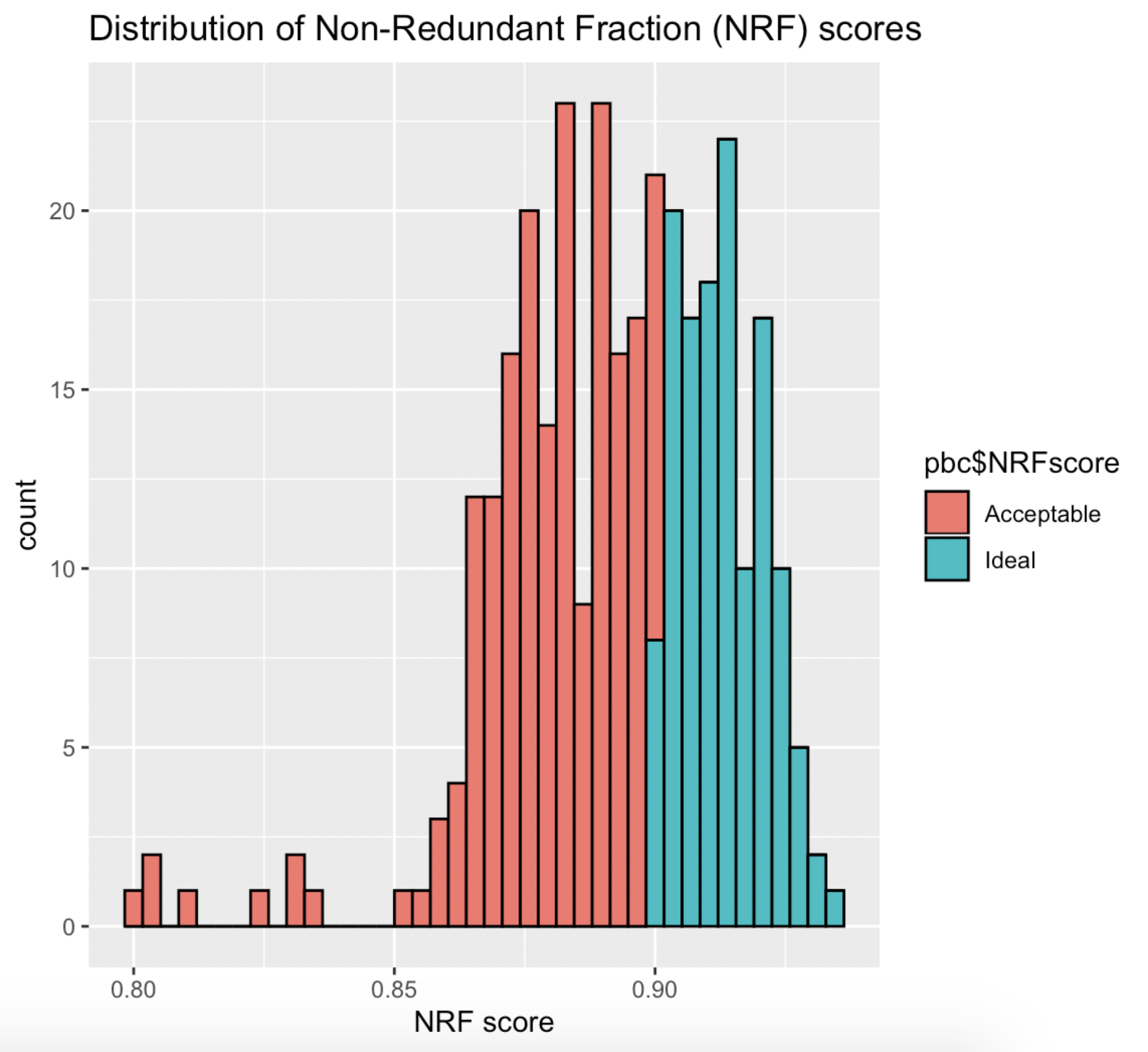
**

**Supplementary Figure 10 Description:** Quality control metrics, using the thresholds provided by the standard ENCODE Pipeline for the identification of open chromatin regions (OCRs) of the genome. A shows distribution of all reads present in the peaks identified in the ATAC-seq dataset, as well as the distribution of all the reads by sample. B shows library complexity metrics of PCR Bottlenecking Coefficients (PBC), measured as PBC1 = [# of positions with exactly 1 read mapped] / [# of positions with 1 or more reads mapped ] and PBC2 = [# of positions with exactly 1 read mapped] / [# of positions with 2 reads mapped ]. Red cutoff color indicates severe PCR bottlenecking threshold (<0.7 for PBC1, <1 for PBC2), green cutoff corresponds to no PCR bottleneck (>0.9 for PBC1, >3 for PBC2). C shows the distribution of Non-redundant Fraction(NRF) scores. Values within 0.7 and 0.9 are considered acceptable. The outlying samples across QC were from the 2014L00966 cell line, time points 20 and 0.
