# Supplementary material for: Fibroblasts as an in vitro model of circadian genetic and genomic studies
Source: Mamm Genome. 2024 Jul 3;35(3):432–44. doi: 10.1007/s00335-024-10050-7 (PMC11329553; doi:10.1007/s00335-024-10050-7)
Supplement: Supplementary file 12 — Supplementary file12 (DOCX 13 kb) [file 335_2024_10050_MOESM12_ESM.docx]

**[Supplementary](sps:id::sec17) Information**

Supplementary files:

All_Circadian_Genes_Heatmap_Clusters.zip: Zip file containing heat map plots of the 12 clusters obtained from the previously identified circadian genes in this dataset.

ATACQC_Scores.csv: CSV containing quality control metrics for the ATAC-seq data. First column is sample name, second column is the Fractions of reads in Peaks score, third column is the Non Redundant Fraction values, columns 4 and 5 are the PCR bottlenecking coefficients 1 and 2 respectively.

ATAC-seq_Sequencing_Depth.csv: CSV containing sequencing depth information for the ATAC-seq data samples. First column is sample name, second column is the sequencing depth as obtained by fastqc software in millions.

Full_Metascape_Output.zip: Zip file containing full Metascape gene ontology analysis for the WGCNA modules identified from the RNA-seq data.

GO_Results: Gene ontology results from Metascape. Last column indicates the WGCNA module.

MetaCycle_Results.zip: Zip file containing results from meta2d, JTK, ARSER, LS and RAIN analysis.

SLDSC_Enrichment_Results: Directory containing enrichment results from the Stratified linkage disequilibrium score analysis for ADHD, Bipolar disorder, Schizophrenia, Insomnia, MDD, Morningness and PTSD.

TSS QC Summary: PDF file including coverage curves of nucleosome-free and nucleosome signals for each ATAC-seq sample. Transcription Start Site (TSS) Enrichment Score is indicated in bold next to the sample name.

WGCNA_ATAC_Modules_ID.csv: CSV file containing WGCNA module membership results for the ATAC-seq data.

WGCNA_RNA_Modules_ID.csv: CSV file containing WGCNA module membership results for the RNA-seq data.
